# Electron-Donor–Acceptor Enabled Electrophilic C(sp^2^)‑C(sp^3^) Cross-Coupling

**DOI:** 10.1021/jacsau.6c01012

**Published:** 2026-08-14

**Authors:** Zheng Wang, Xiaodong Qiu, Burkhard König

**Affiliations:** Fakultät für Chemie und Pharmazie, 9147 Universität Regensburg, 93040 Regensburg, Germany

**Keywords:** EDA complex, metal-free, XAT, aminosilane, C(sp^2^)-C(sp^3^) coupling

## Abstract

The construction of C­(sp^2^)-C­(sp^3^) bonds is fundamental to pharmaceutical synthesis, yet metal-free approaches to electrophilic cross-coupling remain underdeveloped. Herein, we report a visible-light-driven, metal-free cross-coupling between heteroaryl nitriles and alkyl bromides enabled by a rationally designed aminosilane, (TMS)_3_SiNHPh. This reagent engages electron-deficient nitriles in an EDA complex via π–π interaction. Photoinduced electron transfer generates a silyl radical that triggers XAT from alkyl bromides, delivering alkyl radicals for subsequent decyanative coupling without the need for an external photocatalyst or transition-metal catalyst. The method accommodates a broad range of substrates, providing alkylated heteroarenes in moderate to good yields, and extends to Minisci-type C–H alkylations, underscoring the versatility of this platform. This work demonstrates that strategic EDA donor design can address a long-standing challenge in metal-free electrophilic coupling.

## Graphical abstract

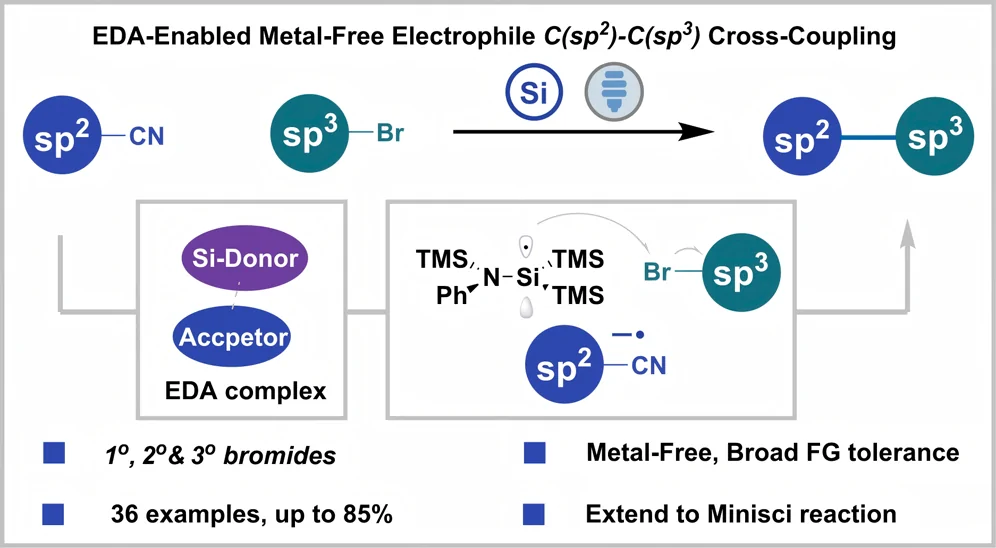

## Introduction

The construction of C­(sp^2^)-C­(sp^3^) bonds remains a linchpin in pharmaceutical discovery.^1–5^ Although transition metal-catalyzed cross-coupling has long served as the primary platform for assembling such motifs,^6–8^ electrochemical^9–11^ and photochemical approaches^4^ are also undergoing continuous development (Scheme 1A). Over the past decade, silane-mediated halogen atom transfer (XAT) has emerged as a powerful strategy for alkyl radical generation, particularly in combination with dual nickel-photoredox catalysis. This activation mode, as reported by the MacMillan group,^12–18^ has enabled a diverse array of cross-electrophile couplings, including alkyl–alkyl cross-coupling,^16^ trifluoromethylation^15^ and aryl-alkyl cross-coupling,^12,14^ highlighting the versatility of silane-based radical chemistry (Scheme 1B). Despite these advances, the development of metal-free electrophilic coupling reactions continues to pose challenges regarding sustainability and functional-group compatibility.

**Scheme 1:**
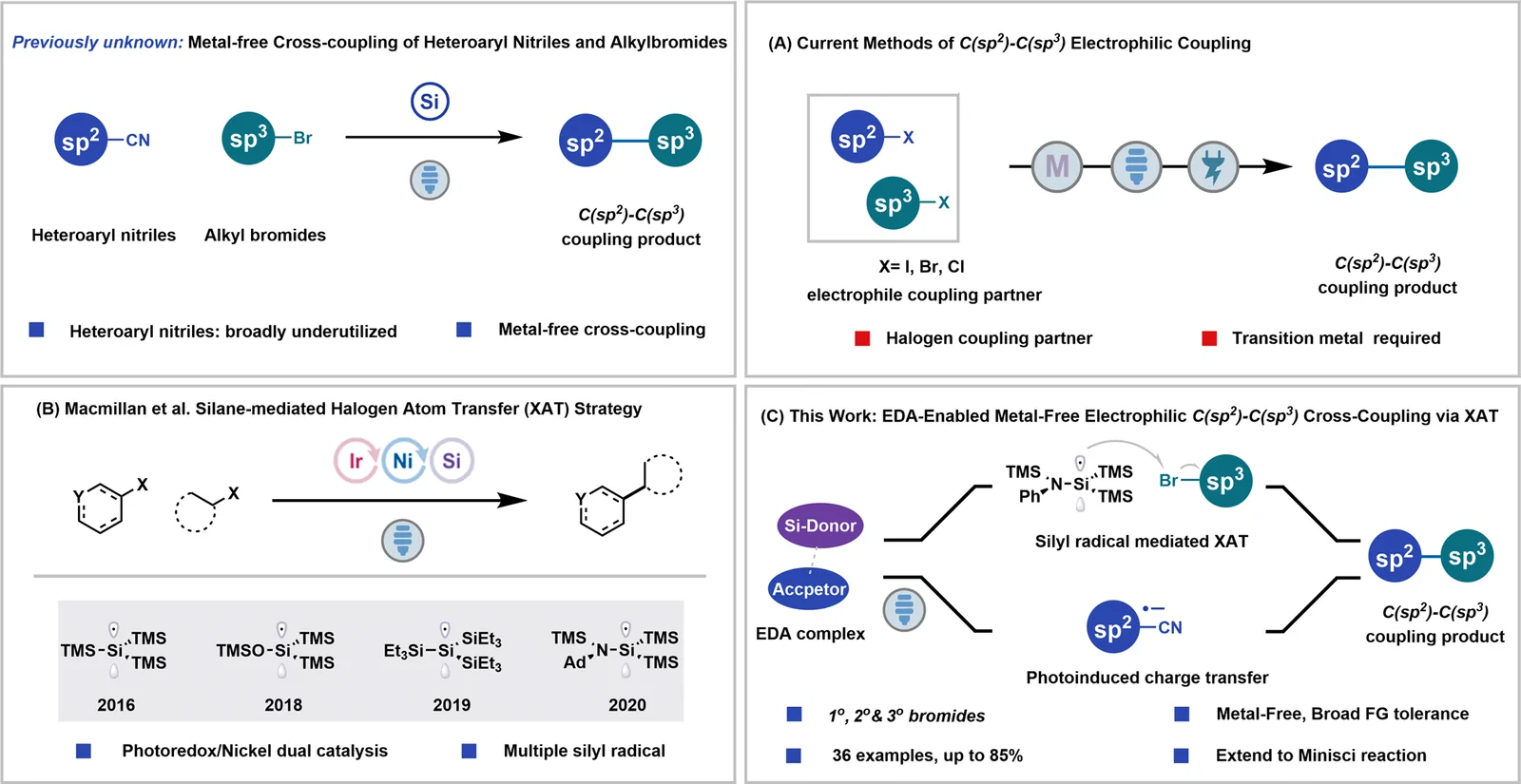
Electrophilic Cross-Coupling Reactions.

In parallel to these metal-based approaches, electron donor–acceptor (EDA) complexes have provided a photocatalyst-free access to single-electron transfer chemistry.^19^ Surprisingly, the strategic merger of EDA complexation and silyl radical generation for XAT remains unexplored, despite its potential for metal-free bond construction. Within this underexplored merger, heteroaryl nitriles represent attractive yet underutilized electrophilic partners owing to their strong electron-accepting ability.^20^ However, their robust C–CN bonds are notoriously resistant to activation under mild, metal-free conditions.

We aimed to investigate whether a single organosilane could bridge this gap by operating in a dual capacity, namely as an electron donor for EDA complex formation with heteroaryl nitriles and as a precursor to silyl radicals upon visible-light irradiation. Our group has longstanding interests in EDA photochemistry,^21,22^ and the MacMillan group demonstrated the power of silyl radicals in XAT processes in 2020.^14^ We envisioned that these two concepts might be unified in a single organosilane, such as (TMS)_3_SiNHPh. The aniline-derived donor was anticipated to engage electron-deficient nitriles in EDA complexation through π–π interactions, serving as an electron donor. Photoinduced electron transfer would then generate a silyl radical, triggering XAT from alkyl bromides and delivering the key alkyl radical intermediate.

Here we report a visible-light-driven, metal-free electrophilic cross-coupling between heteroaryl nitriles and alkyl bromides (Scheme 1C). The method utilized an EDA complex between (TMS)_3_SiNHPh and the nitrile substrate, generating a silyl radical that triggers XAT to produce an alkyl radical, which then undergoes decyanative coupling. Notably, the reaction proceeds without the need for an external photocatalyst or transition-metal catalyst, distinguishing it from established dual catalytic manifolds. This platform demonstrates broad compatibility with alkyl bromides and heteroaryl nitriles, delivering products in moderate to good yields and extending to Minisci-type C–H alkylations, thereby underscoring the versatility of this silane platform for metal-free radical generation and bond construction.

## Reaction Development

Evaluating aminosilane derivatives, substrate ratios, solvents, and additives, we found that optimal conditions enabled electrophilic cross-coupling using (TMS)_3_SiNHPh (**3**). Using a nitrile substrate (1.0 equiv), a bromide (1.6 equiv), and NEt_3_ (1.0 equiv) as an additive under 365 nm light irradiation gave the desired product in good yield (Table 1, **entry 1**, 62%). Control experiments were conducted to probe the key reaction parameters (Table 1). In the absence of light, no product formation was observed (**entry 2**), indicating the essential role of photoirradiation. Similarly, omission of the aminosilane resulted in only trace conversion (**entry 3**), and no improvement was observed when both the aminosilane and NEt_3_ were excluded (**entry 4**). The exclusion of NEt_3_ led to a diminished yield (54%, **entry 5**), suggesting its beneficial role in the transformation. Substitution of (TMS)_3_SiNHPh with tris­(trimethylsilyl)­silane (TTMSS) and AdNHSi­(TMS)_3_ was proved ineffective (**entries 6** and **7**), underscoring the unique reactivity of the (TMS)_3_SiNHPh. Although a naphthylamine-derived aminosilane exhibited comparable reactivity (Table S1), its use was limited by operational challenges arising from its viscous state. Consequently, the liquid (TMS)_3_SiNHPh was selected as the reagent of choice. Performing the reaction under air or at elevated temperature (55 °C) led to decreased yields (44% and 43%, **entries 8** and **9**, respectively).

**Table 1:**
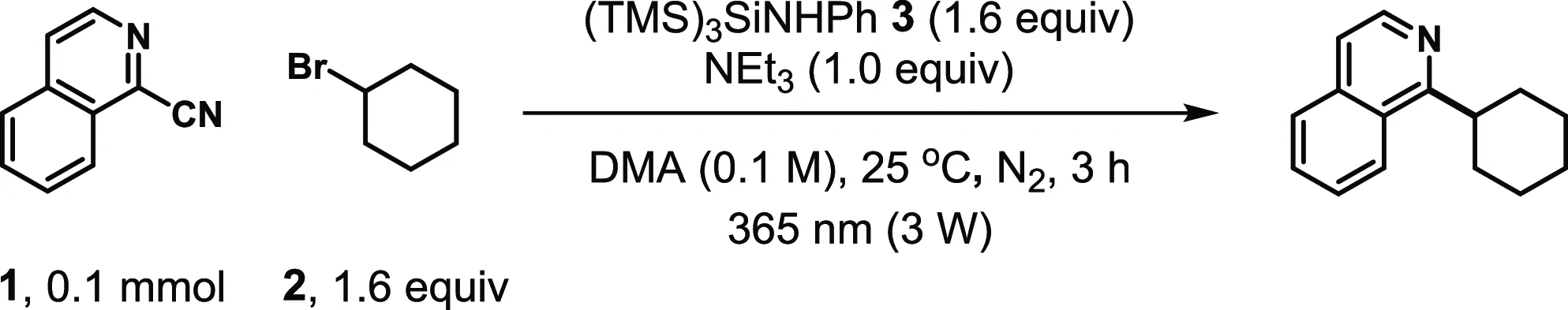
Optimization of Reaction Conditions and Control Experiments

entry^a^	conditions	yield (%)^b^
1	none	62(60)
2	no light	0
3	no aminosilane	<5
4	no aminosilane and NEt_3_	<5
5	no NEt_3_	54
6	TTMSS as Si regent	<5
7	AdNHSi(TMS)_3_ as Si reagent	39
8	open to air	44
9	at 55 °C	43

a Preliminary reactions were run with 1-isoquinolinecarbonitrile (0.1 mmol), alkyl bromide (1.6 equiv), (TMS)_3_SiNHPh (1.6 equiv), and NEt_3_ (1.0 equiv) in DMA (0.1 M) under N_2_ atomsphere at 25 ^o^C, irradiated with 365 nm (3W) LED for 3 h.

b Yields were determined by GC-FID with 0.1 mmol *n*-dodecane as standard. Yields in parentheses are isolated yields. Ad = adamantane.

## Substrate Scope

Subsequently, we turned our attention to evaluating the generality of this protocol (Scheme 2). We first examined the scope with respect to the alkyl bromide, which furnished the desired products **4** (**4-1**to**4-17**) in moderate to good yields. Notably, primary, secondary, and tertiary alkyl bromides were all found to be compatible under the standard reaction conditions. A broad range of functionalized alkyl bromides proved to be well tolerated, including those bearing alkyl cyclic rings (**4**–**1**), silyl ethers (**4**–**2**), phenyl groups (**4**–**3**), acetals (**4**–**4**), esters (**4**–**5**), nitriles (**4**–**6**), boron esters (**4**–**8**), benzyl moieties (**4**–**7** to **4**–**10**) and amides (**4**–**12**) (Scheme 2).

**Scheme 2:**
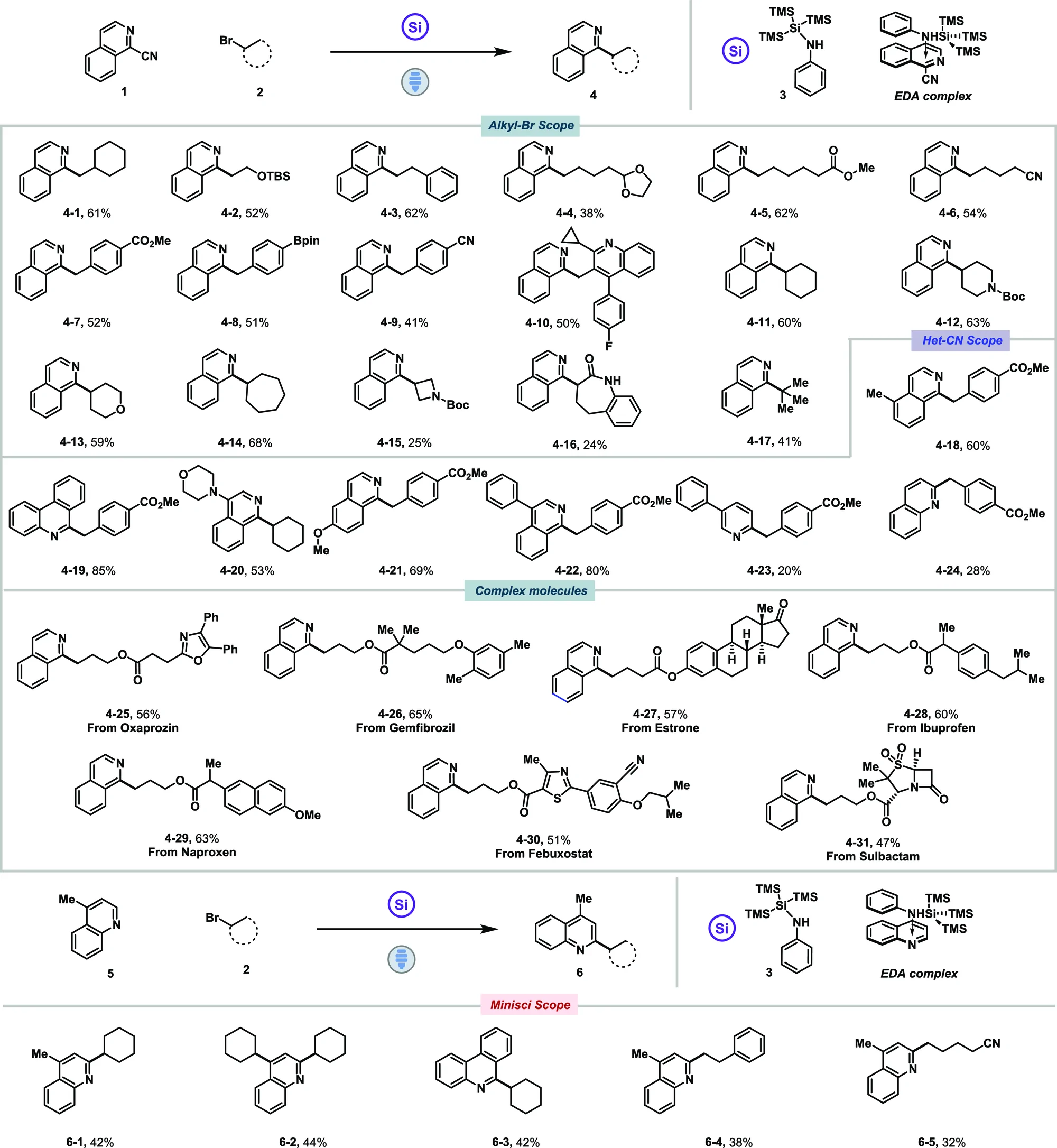
Electron-Donor-Acceptor Enabled Electrophilic C­(sp^2^)-C­(sp^3^) Cross-Coupling^a^

a General reaction conditions: **1** (0.2 mmol), **2** (0.32 mmol), **3** (0.32 mmol), NEt_3_ (0.2 mmol) in 2 mL DMA, N_2_, LEDs (λ_max_ = 365 nm, 3 W) at 25 °C for 3 h. Isolated yields were given. General reaction conditions for Minisci-type reaction: **5** (0.3 mmol), **2** (0.2 mmol), **3** (0.24 mmol), NPh_3_ (0.2 mmol) in 2 mL DMA, N_2_, LEDs (λ_max_ = 365 nm, 3 W) at 25 °C for 6 h. Isolated yields were given.

Following this, we next investigated a series of C­(sp^2^) nitriles as coupling partners. Our investigations revealed that both electron-rich and electron-neutral heteroaryl nitriles could be employed to provide the corresponding adducts in good yield (**4**–**18** to **4**–**24**). Notably, expanding the π system of the heteroaryl nitrile coupling partners led to a marked increase in reaction efficiency, with the corresponding products obtained in significantly improved yields (**4**–**19**, **4**–**22**). We hypothesize that this trend arises from the intrinsic red-shift of the heteroarene absorption upon increasing π-conjugation. Accordingly, the absorption of the corresponding EDA complex shifts to longer wavelengths, following the substrate absorption profile, and its formation is likely facilitated by π-interactions with the electron donor (Figure S18). In addition, pyridine- (**4**–**23**) and quinoline- (**4**–**24**) derived nitriles were also competent coupling partners, affording the corresponding C­(sp^2^)-C­(sp^3^) adducts, albeit in modest yields (Scheme 3).

**Scheme 3:**
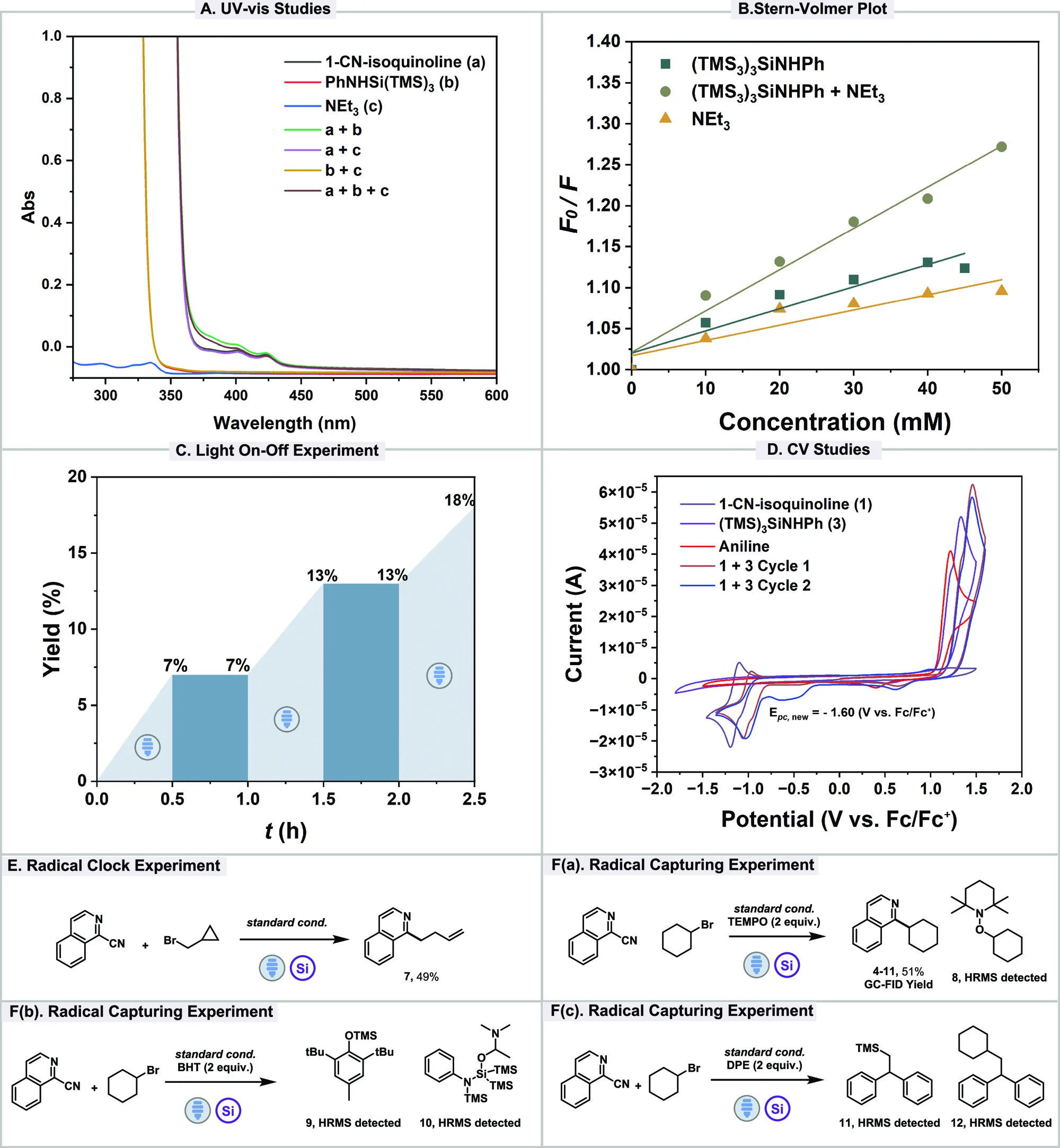
Experimental Studies on the Reaction Mechanism^a^

a Mechanism Studies: (A) UV–vis studies on the formation of a reactant complex. (B) Stern–Volmer plot. (C) On–Off experiment. (D) Cyclic voltammetry studies. (E) Radical clock experiment. (F) Radical capturing experiment.

To further demonstrate the applicability of this methodology for late-stage modification, we investigated the compatibility of our protocol with biologically relevant molecules. Moderate to good yields were obtained for coupling products derived from complex molecules such as Oxaprozin (**4**–**25**), Gemfibrozil (**4**–**26**), Estrone (**4**–**27**), Ibuprofen (**4**–**28**), Naproxen (**4**–**29**), Febuxostat (**4**–**30**), and Sulbactam (**4**–**31**). To highlight the synthetic utility of this strategy, the reaction was performed on a gram scale (Scheme 4), affording **4**–**10** in 51% isolated yield under the standard conditions. Notably, the protocol could be translated to flow chemistry, enabling the synthesis of **4**–**7** in 49% isolated yield.

**Scheme 4:**
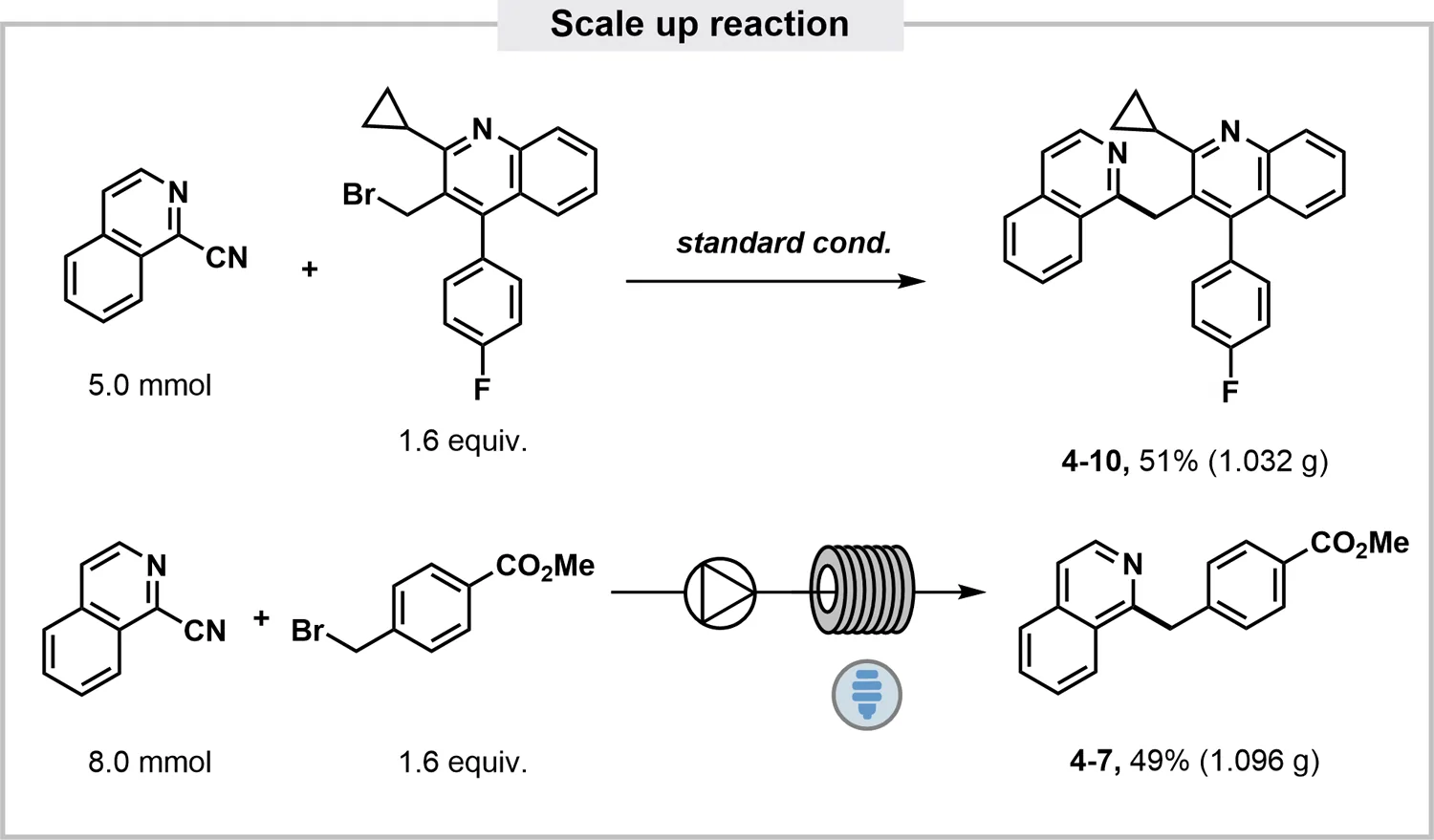
Scale up Reaction

Interestingly, our protocol was also found to be extendable to Minisci-type reactions. Importantly, these transformations proceeded smoothly under acid-free conditions, which are typically incompatible with classical Minisci-type protocols,^23^ affording the desired products in modest yields (**6**–**1** to **6**–**5**). UV–vis studies (Figure S4) indicate the formation of an EDA complex between NPh_3_ and the heteroarene substrate, suggesting that NPh_3_ primarily functions as an electron donor to generate the photoactive complex. The acid-free Minisci reactivity may be facilitated by Lewis acid activation^24^ from silicon-containing species generated during the XAT process, such as silyl bromide species, which could enhance the electrophilicity of the heteroarene substrate and promote the radical addition. However, the exact nature of the active species remains to be fully established.

## Mechanistic Studies

We next conducted a series of experiments to gain insight into the reaction mechanism (Scheme 3). UV–vis analysis revealed a distinct bathochromic shift only when 1-isoquinolinecarbonitrile was combined with (TMS)_3_SiNHPh, whereas no such shift was observed in the presence of NEt_3_ (Scheme 3A). These results are consistent with the formation of an EDA complex between the nitrile substrate and the aminosilane. Stern–Volmer fluorescence quenching studies showed that both NEt_3_ and (TMS)_3_SiNHPh independently quenched the excited state of the substrate, with a significantly enhanced quenching effect observed when both components were present, suggesting their cooperative involvement in the photoinduced process (Scheme 3B). Lifetime quenching studies revealed that the excited state of 1-CN-isoquinoline can be effectively quenched dynamically by (TMS)_3_SiNHPh and NEt_3_ (Figures S11–S12). Light-on/off experiments revealed that product formation occurs only under continuous irradiation, indicating the necessity of light for the progress of the reaction (Scheme 3C). Consequently, the quantum yield for the standard reaction was calculated to be ϕ = 1.8%, suggesting that the reaction does not proceed via a radical chain mechanism. Cyclic voltammetry (CV) studies showed that no additional reduction peaks were observed for the individual starting materials. In contrast, a new reductive signal (E_pc, new_ = −1.60 (V vs Fc/Fc^+^)) emerged when the mixture of starting materials was examined under identical conditions. Control experiments with aniline excluded its direct reduction as the origin of this feature. These results suggest that the new signal arises from a species formed in situ from the combination of the starting materials (Scheme 3D, see Supporting Information for details). Further mechanistic insight was obtained from radical clock and trapping experiments. Under the standard conditions, a cyclopropane-containing substrate afforded a ring-opened olefin product (Scheme 3E), consistent with the intermediacy of an alkyl radical. In addition, upon the addition of 2 equiv of TEMPO, BHT and 1,1-diphenyl ethylene (DPE), radical trapping adducts were detected by HRMS (Scheme 3F), further supporting the involvement of a radical pathway. Notably, in the presence of BHT as a radical scavenger, HRMS detected byproduct **10**, which results from trapping of the aza-Brook-rearranged silyl radical by DMA.

Based on these experimental results, two plausible mechanistic pathways are proposed (Scheme 5). In one pathway, the nitrile substrate and (TMS)_3_SiNHPh form an EDA complex, which upon photoexcitation undergoes intracomplex electron transfer to generate a silyl radical species.^14^ In an alternative pathway, direct photoexcitation of the nitrile substrate is followed by single-electron transfer involving NEt_3_ and (TMS)_3_SiNHPh, leading to the formation of both a silyl radical and an α-aminoalkyl radical. The resulting silyl radical is proposed to engage in XAT with the alkyl bromide to generate an alkyl radical. However, the α-aminoalkyl radical may also serve as a competent XAT species under the reaction conditions.^25–28^ The resulting alkyl radical subsequently undergoes cross-coupling with the nitrile-derived intermediate, ultimately delivering the desired C­(sp^2^)-C­(sp^3^) product.

**Scheme 5:**
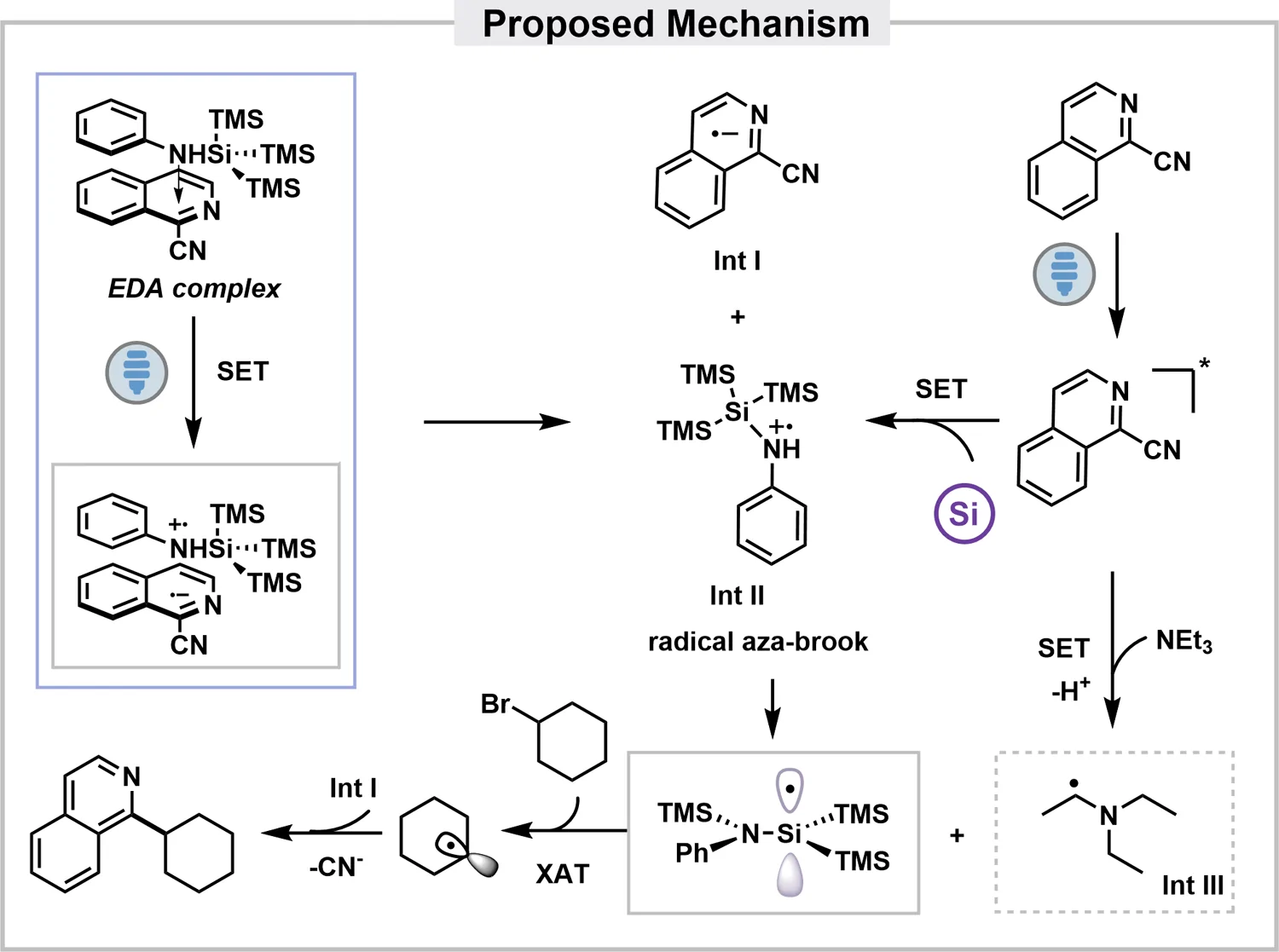
Plausible Mechanistic Pathways for the Silyl Radical-Mediated Cross-Coupling Reaction

## Conclusion

In summary, we have developed a visible-light-driven, metal-free electrophilic cross-coupling between heteroaryl nitriles and alkyl bromides. The transformation proceeds via an EDA complex between (TMS)_3_SiNHPh and the nitrile substrate, generating a silyl radical that engages in XAT to produce an alkyl radical, which subsequently undergoes decyanative coupling. Notably, the reaction proceeds without the need for an external photocatalyst or transition-metal catalyst. This protocol is compatible with a broad range of alkyl bromides and heteroaryl nitriles, delivers the desired products in moderate to good yields, and can be extended to Minisci-type C–H alkylations, demonstrating the versatility of this silane platform for metal-free radical generation and C–C bond formation.

## Supplementary Material



## Notes

Experimental procedures, materials and methods, characterization data, NMR spectra, photophysical studies, and mechanistic investigations (PDF)
