# Supplementary material for: Electron-Donor–Acceptor Enabled Electrophilic C(sp2)‑C(sp3) Cross-Coupling
Source: JACS Au. 2026 Aug 14;6(9):5344–50. doi: 10.1021/jacsau.6c01012 (PMC13625544; doi:10.1021/jacsau.6c01012)
Supplement: Supplementary file 1 [file au6c01012_si_001.pdf]

Supplementary Information

Electron-Donor-Acceptor Enabled Electrophilic $C(sp^2)$-$C(sp^3)$ Cross-Coupling

Zheng Wang,[†1] Xiaodong Qiu,[†1], Burkhard König*[1]

[1]Fakultät für Chemie und Pharmazie, Universität Regensburg, 93040 Regensburg, Germany

The correspondence may be addressed to: burkhard.koenig@chemie.uni-regensburg.de (B.K)

[†] These authors contributed equally to this work.

Primary research data of this manuscript are available at:

https://www.radar-service.eu/radar/de/dataset/5f02hwyah2te08na

Table of Content

1. General Information 3
2. Preparation of Starting materials 5
   2.1 Synthesis and characterization of silane reagents 5
   2.2 Synthesis and characterization of other starting materials 7
3. Reaction Optimization and Control Experiments 12
   3.1 Procedures for the optimization of the reaction conditions 12
   3.2 Optimization of Coupling reaction 12
   3.3 Optimization of Minisci type reaction 22
4. Mechanistic Studies 26
   4.1 UV-vis Absorption Studies 26
   4.2 Radical Clock Experiment 28
   4.3 Radical Capturing Experiment 29
      4.3.1 Using TEMPO 29
      4.3.2 Using BHT 30
      4.3.3 Using 1,1-diphenyl ethylene 32
   4.4 Fluorescence Quenching Experiments 34
   4.5 Excited State Lifetime Studies 36
   4.6 Cyclic Voltammetry Studies 38
   4.7 Light On-Off Experiment 41
   4.8 π-π Interaction Studies 42
   4.9 Quantum yield measurements 43
5. Synthesis and characterization of products 44
   5.1 characterization of products 45
   5.2 Scale-up reaction 59
      5.2.1 Gram-scale reaction 59
      5.2.2 Gram-scale reaction with flow reactor 60
   5.3 Failed substrate scope 61
   5.4 Mass blance analysis 62
6. NMR Data 63
7. References 113

1. General Information

All reagents were purchased from Alfa-Aesar, TCI, Sigma-Aldrich, ABCR, Acros or Fluorochem, and were checked for purity by GC-MS and/or ^{1}H NMR spectroscopy and used as received. Unless otherwise noted, all manipulations were performed using standard Schlenk manifold technique. Extradry anhydrous N, N-dimethylformamide，N, N-dimethylacetamide and dimethyl sulfoxide were purchased from Acros. All final products NMR spectra were recorded at ambient temperature using Bruker Avance 400 NMR (^{1}H, 400 MHz; ^{13}C{^{1}H}, 101 MHz; ^{19}F{^{1}H}, 377 MHz) spectrometers. ^{1}H NMR chemical shifts are reported relative to TMS and were referenced via residual proton resonances of the corresponding deuterated solvent ($CDCl_3$: 7.26 ppm, CD_3CN: 1.94 ppm, DMSO-d_6: 2.50 ppm, THF-d_8: 1.72, 3.58 ppm, D_2O: 4.79 ppm) whereas ^{13}C{^{1}H} NMR spectra are reported relative to TMS and were referenced via the carbon signals of the deuterated solvent ($CDCl_3$: 77.16 ppm, CD_3CN: 1.32, 118.26 ppm, DMSO-d_6: 39.52 ppm, THF-d_8: 67.21, 25.31 ppm). ^{19}F NMR chemical shifts are reported relative to $CFCl_3$ as external standard. All ^{13}C and ^{19}F NMR spectra were broad-band ^{1}H decoupled. Coupling constants J are given in Hertz (Hz). Abbreviations used for signal multiplicity: ^{1}H-NMR: b = broad, s = singlet, d = doublet, t = triplet, q = quartet, p = quintet, and m = multiplet.

High-resolution mass spectra (HRMS) were obtained from the central analytic mass spectrometry facilities of the Faculty of Chemistry and Pharmacy, Regensburg University, and are reported according to the 2013 IUPAC recommendations. All mass spectra were recorded on a Finnigan MAT 95, Thermo Quest Finnigan TSQ 7000, Finnigan MATSSQ 710 A or Agilent Q TOF 6540 UHD instrument. Gas chromatography (GC) measurements were performed on an Agilent GC 7890. Data acquisition and evaluation was done with Agilent ChemStation Rev.C.01.04. Analytical TLC was performed on silica gel-coated alumina plates (MN TLC sheets ALUGRAM ® Xtra SIL G /UV254). Visualization was done by UV light (254 or 366 nm). If necessary, potassium permanganate was used for chemical staining. Purification by column chromatography was performed with silica gel 60 M (40-63 µm, 230-440 mesh, Merck) on a Biotage® Isolera TM Spektra One device. All photochemical reactions for the substrate scopes were performed with OSRAM Oslon SSL 80 royal LEDs (λ = 365 nm ± 15 nm, 3.0 W). The samples were irradiated with an LED through the bottom side and cooled from the side using custom-made aluminum water-cooling blocks connected to a thermostat. The reaction mixture and the LED cooling block were temperature-controlled. Gram-scale reactions were performed in a glass tube photochemical reactor setup irradiated from the outside. UV-Vis and fluorescence measurements were performed with a Varian Cary 100 UV/Vis spectrophotometer and FluoroMax 4 spectrofluorometer, respectively.

Millimolar photoreactor equipment (on the left is a light-stirring device, and on the right is its temperature control equipment)

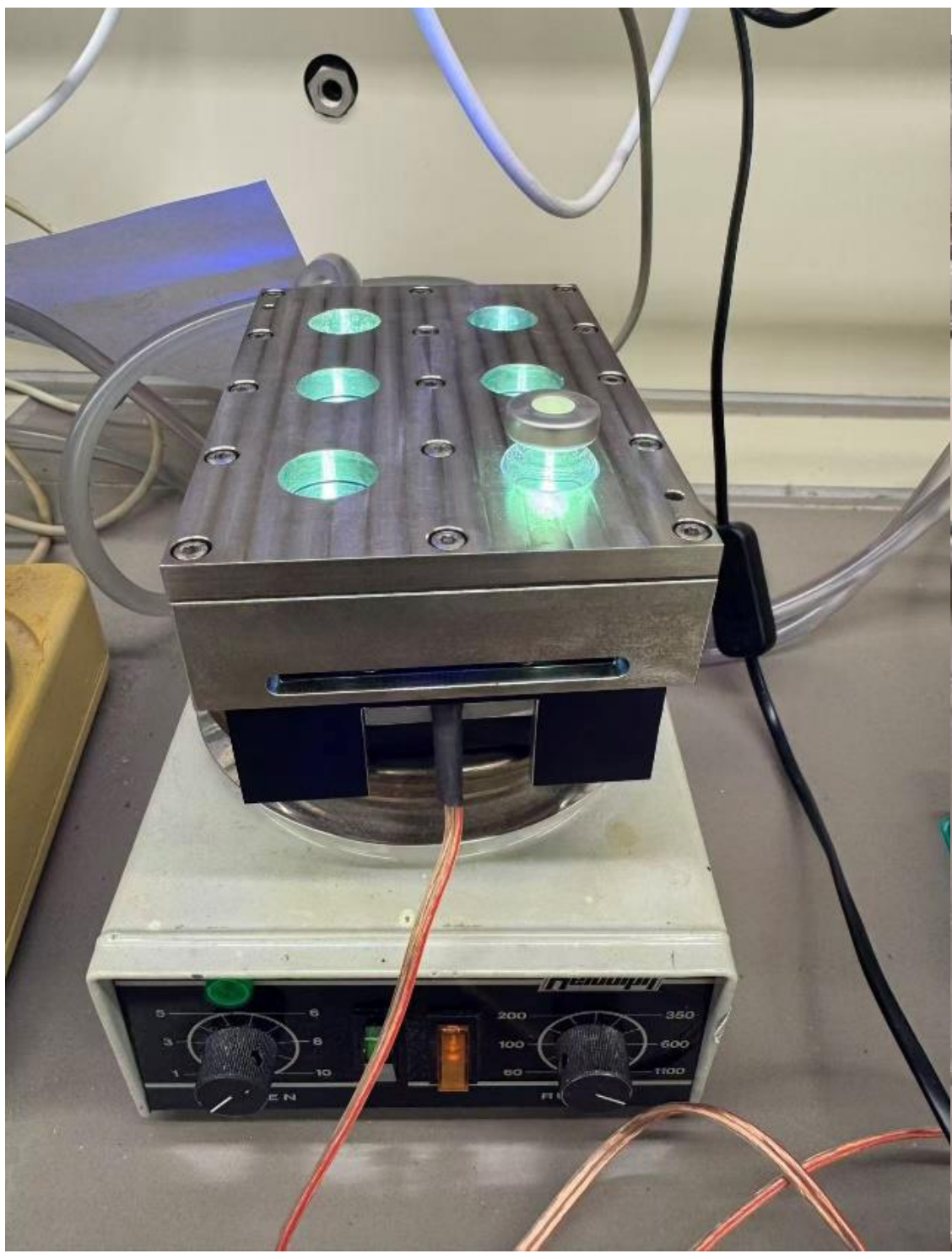

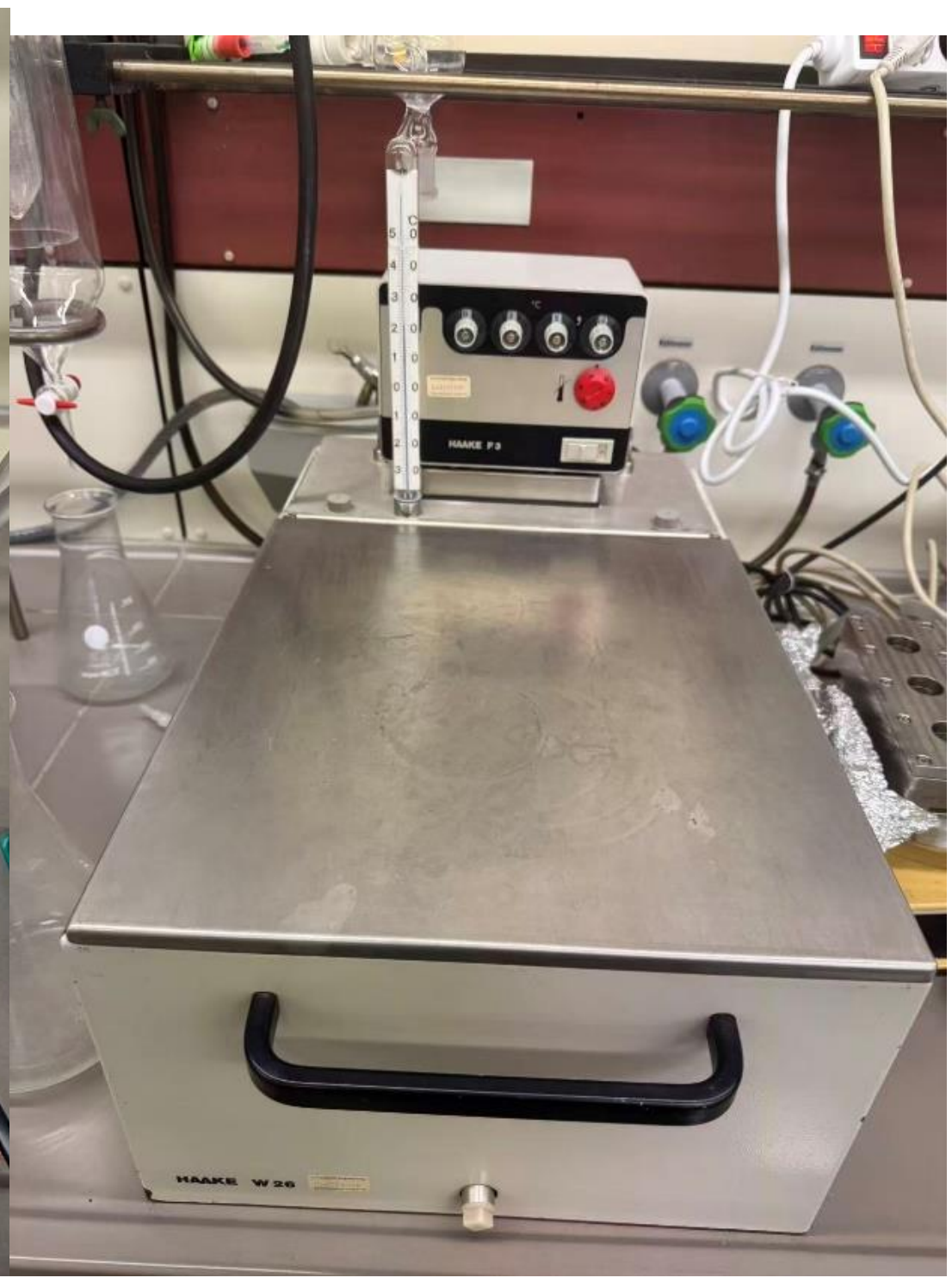

Gram scale equipment (on the left is a light-stirring device, and on the right is a flow photoreactor equipment)

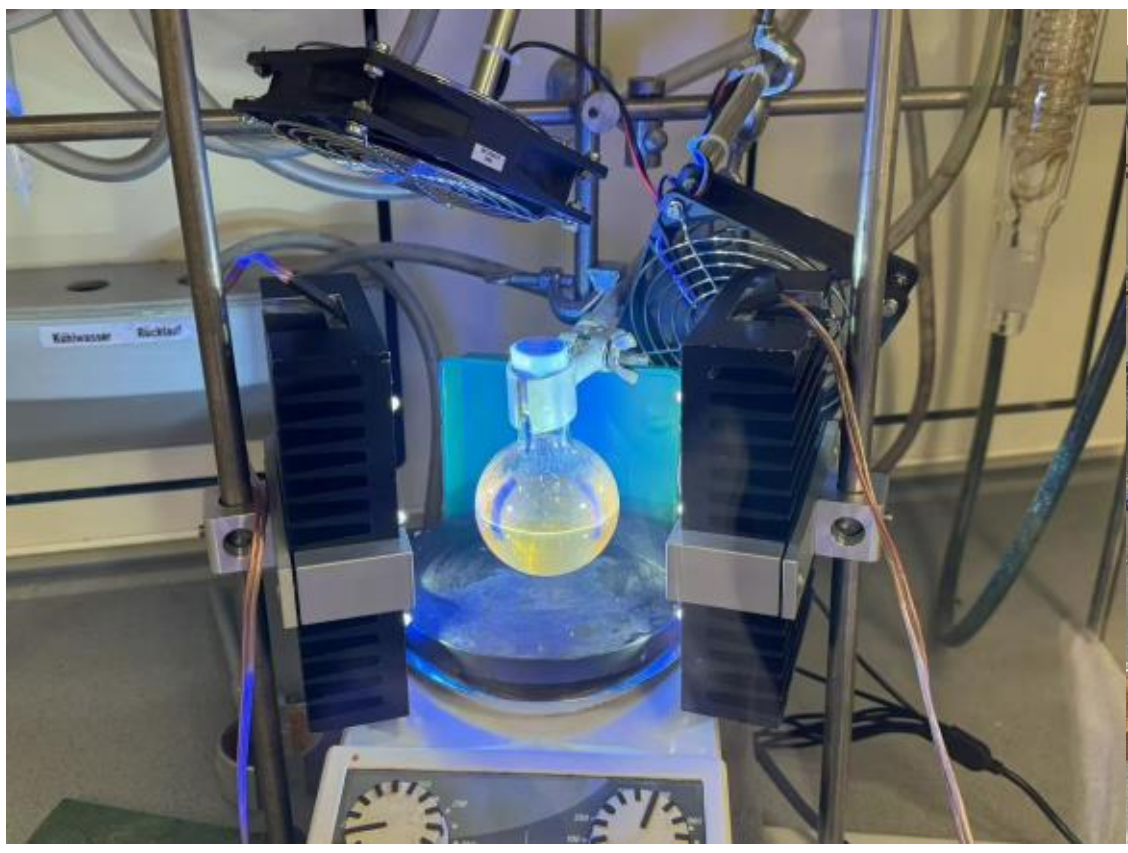

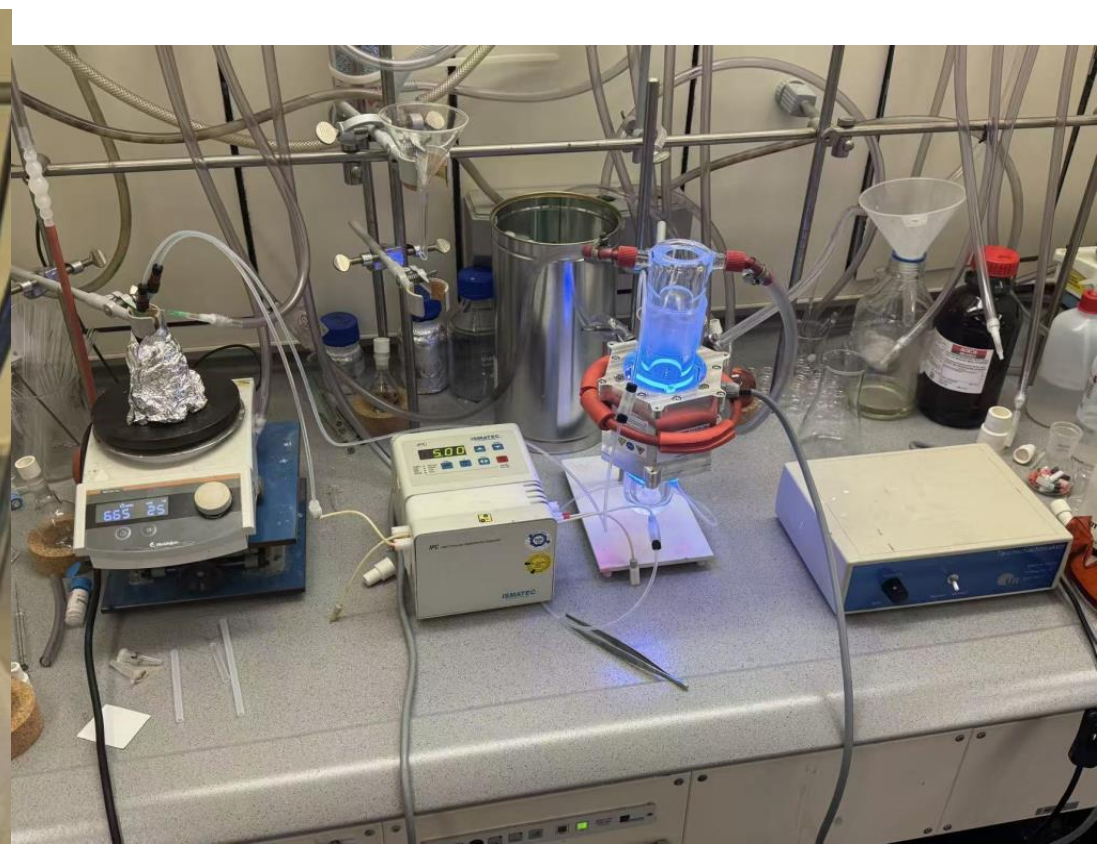

2. Preparation of Starting materials

2.1 Synthesis and characterization of silane reagents

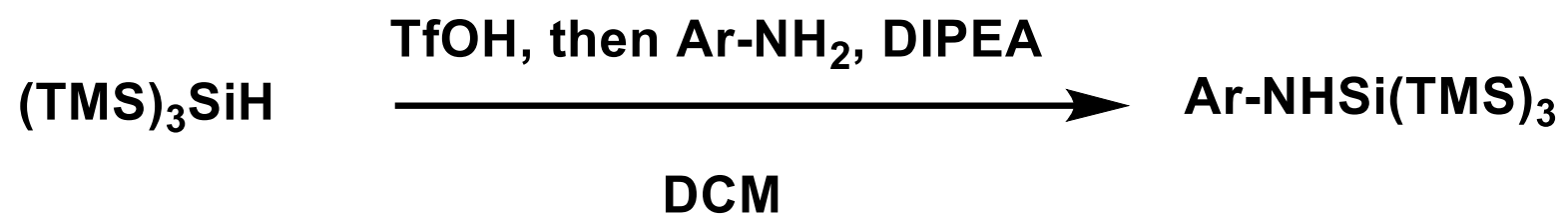

General Procedure

To an oven-dried 50 mL vial containing anhydrous DCM (6mL, 0.33 M) was added trifluoromethanesulfonic acid (0.186 mL, 2.1 mmol, 1.05 equiv.) via syringe. Dropwise addition of tris(trimethylsilyl)silane (0.6 mL, 2 mmol, 1.0 equiv.) at 0℃ was accompanied by evolution of hydrogen gas. The reaction mixture was stirred at room temperature for 1 h, after which the desired amine (2.1 mmol, 1.05 equiv.) and N, N-diisopropylethylamine were added at 0℃. The resulting solution was stirred for 12 h at room temperature. The solvent was removed under reduced pressure, and the resulting white solid was resuspended in 20 mL of hexane. The mixture was filtered with neutral alumina and concentrated in vacuo.

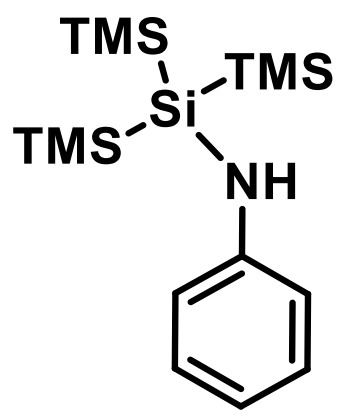

1,1,1,3,3,3-hexamethyl-N-phenyl-2-(trimethylsilyl)trisilan-2-amine ((TMS)$_3$SiNHPh, 3): The title compound was prepared according to the general procedure using aniline (1.4 mL, 15.75 mmol, 1.05 equiv.) and N, N-diisopropylethylamine (3.9 mL, 22.5 mmol, 1.5 equiv.). Removal of remaining starting material by filtration and vacuum afforded the desired product as a colorless oil (3.96 g, 11.6 mmol, 78% yield).

^{1}H NMR (400 MHz, CDCl$_3$) δ 7.15 – 7.07 (m, 2H), 6.70 – 6.61 (m, 3H), 2.91 (s, 1H), 0.22 (s, 27H).

^{13}C NMR (101 MHz, CDCl$_3$) δ 149.7, 129.0, 117.0, 116.3, 0.7.

HRMS (**EI**): *m/z* Calculated for $C_{15}H_{33}NSi_4$ ([M]$^+$): 339.1690; found: 339.1681;

Cyclic voltammetry was performed using a glassy carbon working electrode, a platinum wire counter electrode, and a silver wire pseudo-reference electrode in 0.1 M TBABF$_4$ (in DMA) with 10 mM substrate. Ferrocene was used as an internal standard, and potentials were referenced to SCE by taking **E** (vs. SCE) = **E** (vs. Fc/Fc$^+$) + 0.38 V. The cyclic voltammogram of (TMS$_3$)$_3$SiNHPh was recorded (initial potential = 0 V, Upper vertex potential = +1.5 V (with Fc), Lower vertex potential = - 2.0 V, stopping potential = 0 V) with 50 mV/s scan rate.

E_{pa}(vs SCE) = + 0.88 (V vs SCE in DMA)

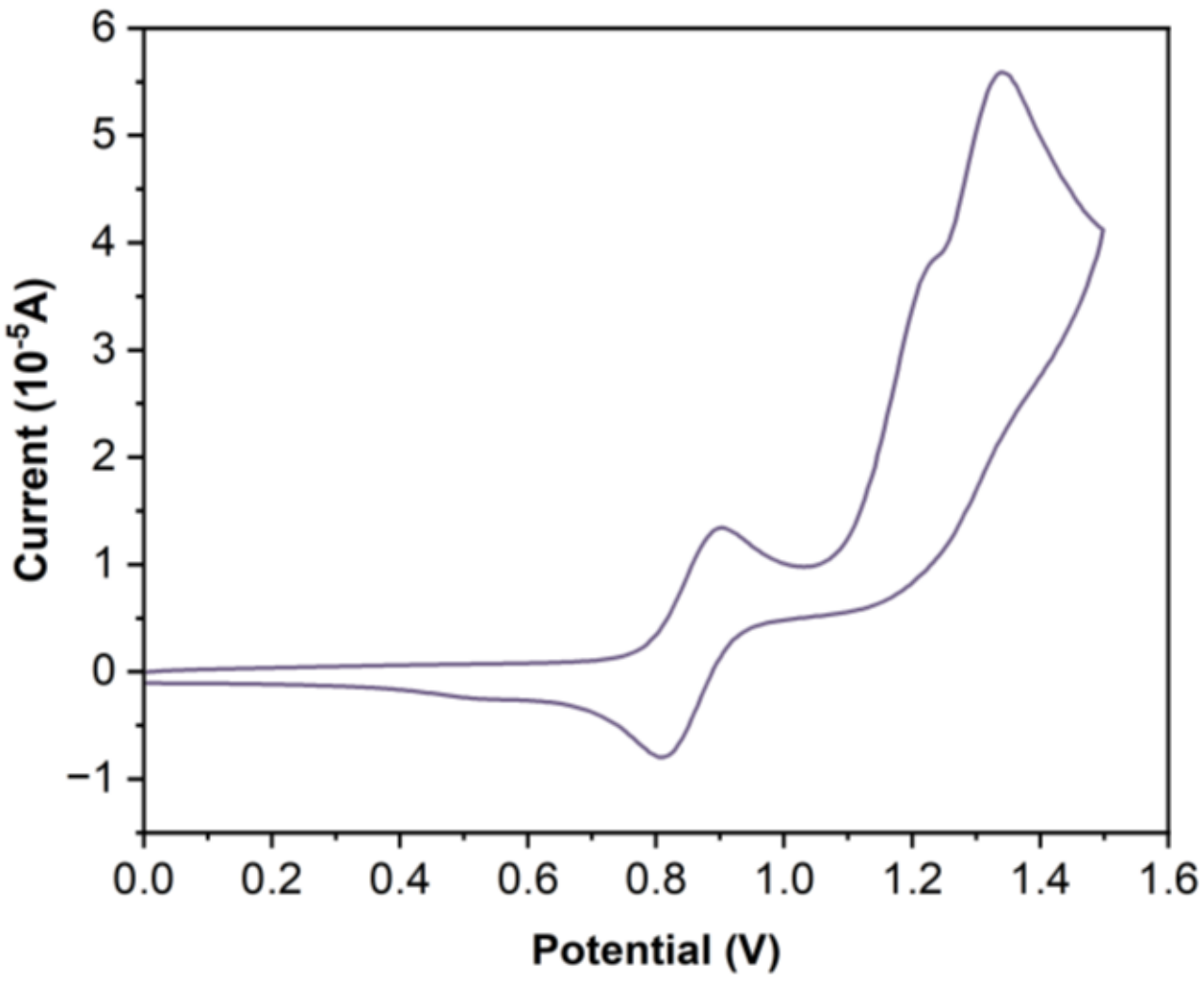

Figure S1. Cyclic voltammogram of compound 3.

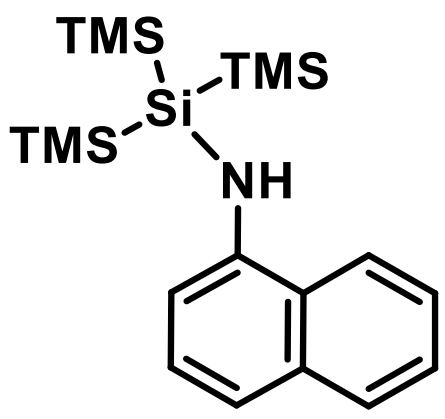

1,1,1,3,3,3-hexamethyl-N-(naphthalen-1-yl)-2-(trimethylsilyl)trisilan-2-amine (TMS$_3$SiNHNaph, S1): The title compound was prepared according to the general procedure using 1-Naphthylamine (0.63g, 4.4 mmol, 1.05 equiv.) and N,N-diisopropylethylamine (1.1 mL, 6.3 mmol, 1.5 equiv.). Removal of the remaining starting material by filtration under vacuum afforded the desired product as a colorless oil (1.22 g, 3.1 mmol, 75% yield).

^{1}H NMR (400 MHz, CDCl$_3$) δ 7.69 (d, *J* = 8.1 Hz, 1H), 7.65 – 7.57 (m, 2H), 7.37 (ddd, *J* = 8.2, 6.8, 1.3 Hz, 1H), 7.20 (ddd, *J* = 8.0, 6.8, 1.2 Hz, 1H), 7.01 (d, *J* = 2.5 Hz, 1H), 6.94 (dd, *J* = 8.7, 2.5 Hz, 1H), 3.15 (s, 1H), 0.29 (s, 27H).
^{13}C NMR (101 MHz, CDCl$_3$) δ 147.6, 135.2, 128.7, 127.8, 127.4, 126.3, 125.5, 121.8, 120.5, 108.8, 0.8.
HRMS (**EI**): *m/z* Calculated for $C_{19}H_{35}NSi_4$ ([M]$^+$): 389.1847; found: 389.1841:

2.2 Synthesis and characterization of other starting materials

Starting materials

Unless otherwise noted, all the bromides were purchased from commercial sources and used without further purification. Substrates (not commercially available) were prepared in accordance with methods described in the references.

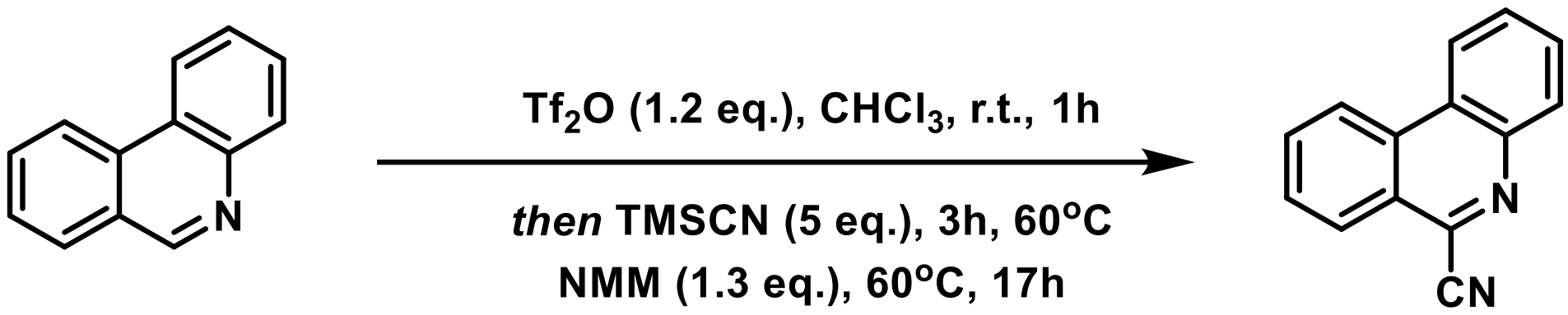

phenanthridine-6-carbonitrile (S2)[1]: Triflic anhydride (0.4 mL, 2.4 mmol, 1.2 eq.) was added dropwise to a solution of starting material (358.6 mg, 2 mmol, 1.0 eq.) in anhydrous $CHCl_3$ (0.1 M) in a sealed vial at room temperature under N_2. The resulting solution was stirred for one hour, then trimethylsilyl cyanide (1.25 mL, 10 mmol, 5.0 eq.) was added. The seal was quickly swapped for a screw cap, and the mixture was stirred at 60 °C for 3 h. The reaction was removed from the heating source, and N-methylmorpholine (0.3 mL, 1.3 eq.) was added quickly before the vial was recapped. The mixture was then stirred at 60 °C for a further 17 h, before cooling to room temperature, and quenching with sat. $NaHCO_3$ solution. The phases were separated, and the organic phase was extracted twice with CH_2Cl_2. The combined organic phase was dried ($MgSO_4$ or Na_2SO_4 as appropriate) and concentrated in vacuo. The crude mixture was purified via flash column chromatography (PE: EA=10:1) as a white solid (90%, 367.5 mg).

^{1}H NMR (400 MHz, $CDCl_3$) δ 8.67 (d, J = 8.4 Hz, 1H), 8.64 – 8.56 (m, 1H), 8.44 (d, J = 8.2 Hz, 1H), 8.25 (ddt, J = 7.6, 5.4, 3.1 Hz, 1H), 7.98 (ddd, J = 8.4, 7.0, 1.3 Hz, 1H), 7.89 – 7.73 (m, 3H).
^{13}C NMR (101 MHz, $CDCl_3$) δ 143.8, 135.9, 132.9, 132.4, 131.2, 130.1, 129.8, 129.0, 126.8, 125.6, 124.9, 122.5, 122.4, 116.0.
LRMS (EI): *m/z* Calculated for $C_{14}H_8N_2$ ($[M]^+$): 204.1; found: 204.1.

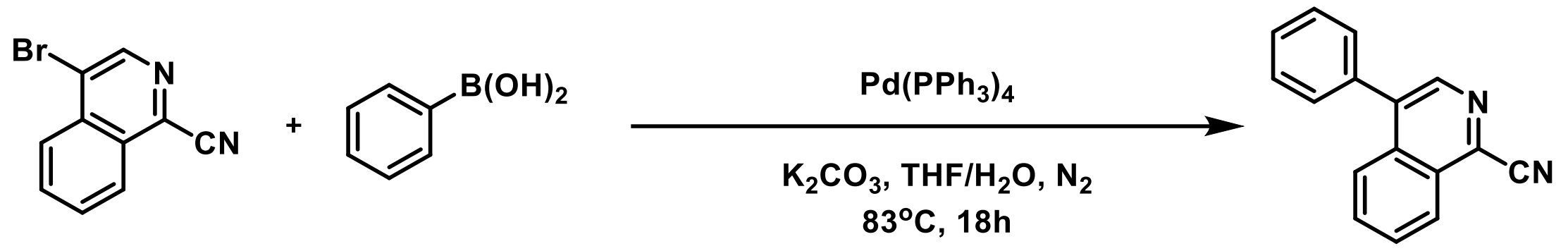

4-phenylisoquinoline-1-carbonitrile (S3): A reaction flask was charged with a mixture of 4-bromoisoquinoline-1-nitrile (466.1 mg, 2.0 mmol), phenylboronic acid (365.8 mg, 3.0 mmol), K_2CO_3 (552.8 mg, 4 mmol), $Pd(PPh_3)_4$ (150.2 mg, 0.13 mmol), THF (2.4 mL), and water (1 mL). The mixture was stirred at 83 °C under a dinitrogen atmosphere for 18 h. Then the mixture was allowed to cool to room temperature, and the product was extracted with DCM (30 mL × 3). The combined organic layers were dried over anhydrous Na_2SO_4, and the solvent was removed under reduced pressure to give a crude product. The crude product was then purified by silica gel chromatography (PE:EA = 5:1) to afford 4-phenylisoquinoline-1-carbonitrile as a white solid (466 mg, 97%).

^{1}H NMR (400 MHz, $CDCl_3$) δ 8.60 (s, 1H), 8.46 – 8.38 (m, 1H), 8.06 – 7.99 (m, 1H), 7.81 (ddt, *J* = 8.2, 6.9, 5.3 Hz, 2H), 7.64 – 7.46 (m, 5H).
^{13}C NMR (101 MHz, $CDCl_3$) δ 143.3, 137.6, 135.6, 134.5, 133.9, 131.9, 130.1, 129.8, 129.4, 129.0, 125.9, 125.7, 116.2.
HRMS (**EI**): *m/z* Calculated for $C_{16}H_{10}N_2$ ($[M]^+$): 230.0844; found: 230.0831:

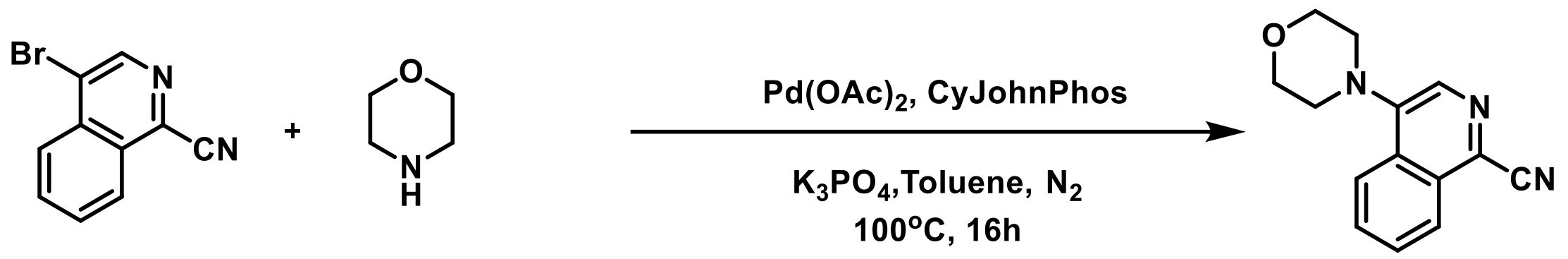

4-morpholinoisoquinoline-1-carbonitrile (S4): A reaction flask was charged with a mixture of 4-bromoisoquinoline-1-nitrile (1.17 g, 5.0 mmol), K_3PO_4 (1.7 g, 8 mmol), $Pd(OAc)_2$ (33.7 mg, 0.15 mmol), CyJohnPhos (105.1 mg, 0.3 mmol), morpholine (0.61 mL, 7 mmol), Toluene (10 mL), The mixture was stirred at 100 °C under nitrogen atmosphere for 16 h. Then the mixture was allowed to cool to room temperature, and the product was extracted with DCM (30 mL × 3). The combined organic layers were dried over anhydrous Na_2SO_4, and the solvent was removed under reduced pressure to give a crude product. The crude product was then purified by silica gel chromatography (PE:EA = 1:1) to afford 4-phenylisoquinoline-1-carbonitrile as a yellow solid (647.9 mg, 54%).

^{1}H NMR (400 MHz, $CDCl_3$) δ 8.33 – 8.28 (m, 1H), 8.26 (d, *J* = 1.3 Hz, 1H), 8.18 – 8.10 (m, 1H), 7.83 – 7.72 (m, 2H), 4.04 – 3.94 (m, 4H), 3.32 – 3.25 (m, 4H).
^{13}C NMR (101 MHz, $CDCl_3$) δ 146.6, 133.1, 130.8, 130.7, 130.2, 129.7, 128.8, 126.0, 123.5, 116.4, 67.0, 52.9.
HRMS (**EI**): *m/z* Calculated for $C_{14}H_{13}N_3O$ ($[M]^+$): 239.1059; found: 239.1051:

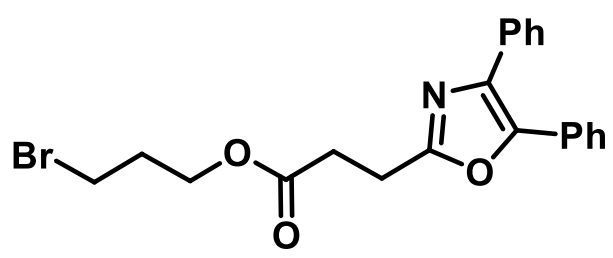

S5

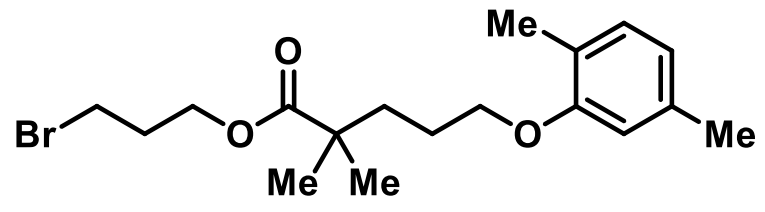

S6

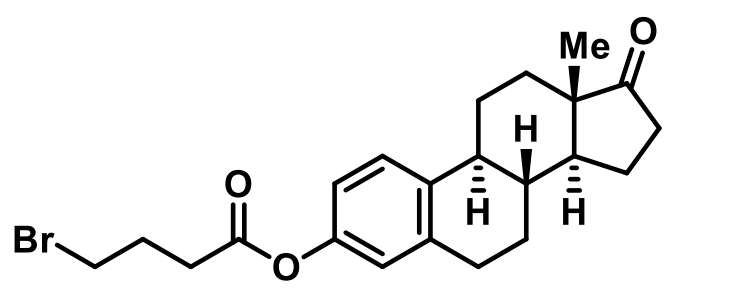

S7

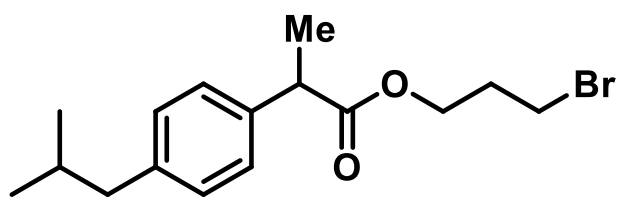

S8

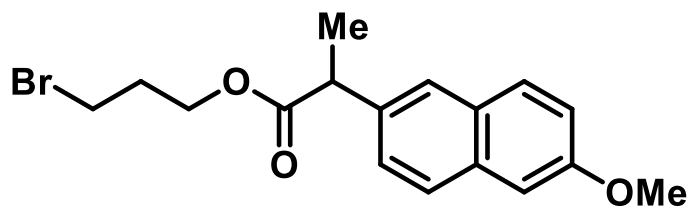

S9

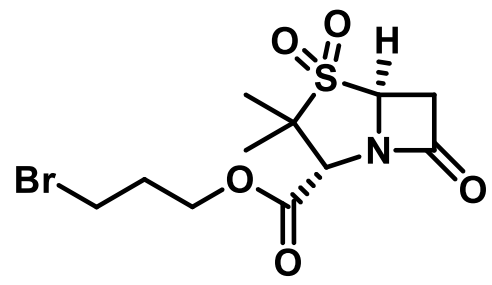

S10

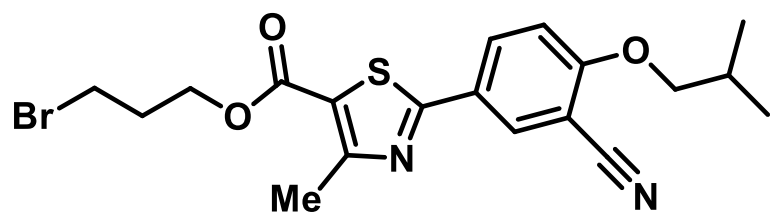

S11

The substrates (**S5-S11**) were prepared according to the general procedure**:**

To a solution of acids (1.0 equiv.), Dicyclohexylcarbodiimide (DCC, 1.2 equiv.) and N, N-dimethyl-4-aminopyridine (DMAP, 20 mol%) in CH_2Cl_2 (DCM, 0.2 M) were added to alcohols or phenols/alcohols (1.2 equiv.). The reaction mixture was stirred for 24 h at room temperature, then filtered and washed with DCM. The combined organic layer was dried over anhydrous Na_2SO_4 and concentrated under reduced pressure. The residue was purified with flash column chromatography on silica gel to give the desired bromides.

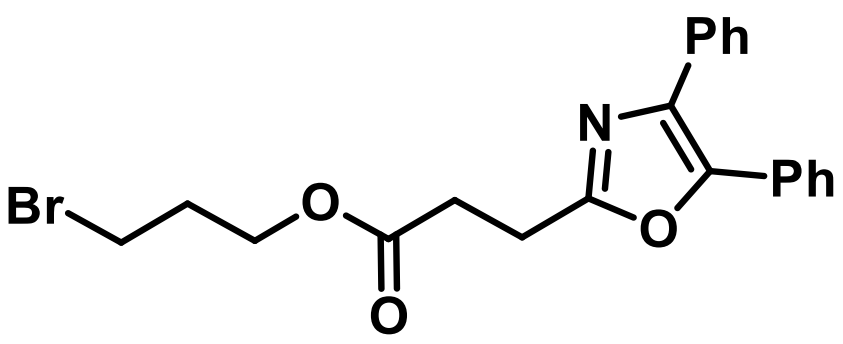

3-bromopropyl 3-(4,5-diphenyloxazol-2-yl)propanoate (S5)[2]**:** Following the general procedure on a 3 mmol scale, substrate **S5** was obtained as an orange oil (1.10 g, 88% yield).

^{1}H NMR (400 MHz, CDCl$_3$) δ 7.66 – 7.61 (m, 2H), 7.60 – 7.55 (m, 2H), 7.41 – 7.29 (m, 6H), 4.28 (t, *J* = 6.0 Hz, 2H), 3.43 (t, *J* = 6.5 Hz, 2H), 3.20 (t, *J* = 7.4 Hz, 2H), 2.94 (t, *J* = 7.4 Hz, 2H), 2.18 (p, *J* = 6.3 Hz, 2H).

^{13}C NMR (101 MHz, CDCl$_3$) δ 172.0, 161.8, 145.6, 135.1, 132.4, 129.0, 128.8, 128.71, 128.67, 128.3, 128.0, 126.6, 62.6, 31.8, 31.1, 29.5, 23.6.

LRMS (**EI**): *m/z* Calculated for $C_{21}H_{20}BrNO_3$ ($[M]^+$): 413.1; found: 413.2.

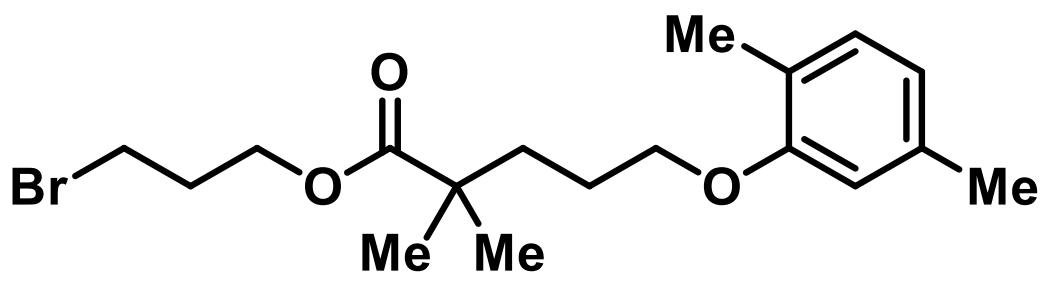

3-bromopropyl 5-(2,5-dimethylphenoxy)-2,2-dimethylpentanoate (S6)[3]: Following the general procedure on a 3 mmol scale, substrate **S6** was obtained as a colorless oil (836 mg, 75% yield).
^{1}H NMR (400 MHz, CDCl$_3$) δ 7.04 – 6.98 (m, 1H), 6.67 (d, *J* = 7.7 Hz, 1H), 6.63 – 6.55 (m, 1H), 4.21 (t, *J* = 6.0 Hz, 2H), 3.99 – 3.87 (m, 2H), 3.46 (t, *J* = 6.5 Hz, 2H), 2.32 (s, 3H), 2.23 – 2.13 (m, 5H), 1.87 – 1.68 (m, 4H), 1.24 (s, 6H).
^{13}C NMR (101 MHz, CDCl$_3$) δ 177.7, 157.0, 136.6, 130.4, 123.6, 120.8, 112.1, 68.0, 62.2, 42.3, 37.3, 31.8, 29.5, 25.31, 25.28, 21.5, 15.9.
LRMS (**EI**): *m/z* Calculated for $C_{18}H_{27}BrO_3$ ([M]$^+$): 370.1; found: 370.1.

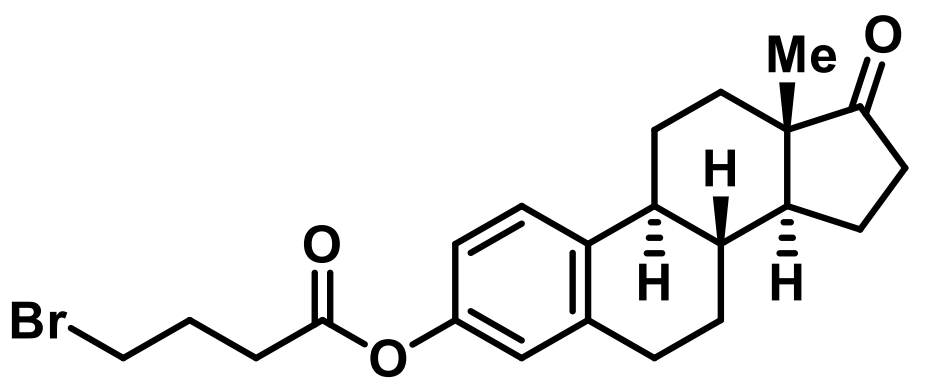

(8R,9S,13S,14S)-13-methyl-17-oxo-7,8,9,11,12,13,14,15,16,17-decahydro-6H-cyclopenta[a]phenanthren-3-yl 4-bromobutanoate (S7)[4]: Following the general procedure on a 3 mmol scale, substrate **S7** was obtained as a white solid (1.163 g, 92% yield).
^{1}H NMR (400 MHz, CDCl$_3$) δ 7.28 (d, *J* = 8.6 Hz, 1H), 6.91 – 6.83 (m, 1H), 6.81 (d, *J* = 2.5 Hz, 1H), 3.54 (t, *J* = 6.4 Hz, 2H), 2.94 – 2.87 (m, 2H), 2.75 (t, *J* = 7.2 Hz, 2H), 2.50 (dd, *J* = 18.9, 8.6 Hz, 1H), 2.44 – 2.37 (m, 1H), 2.28 (p, *J* = 6.7 Hz, 3H), 2.21 – 2.10 (m, 1H), 2.09 – 1.98 (m, 2H), 1.99 – 1.85 (m, 1H), 1.70 – 1.58 (m, 2H), 1.58 – 1.37 (m, 4H), 0.91 (s, 3H).
^{13}C NMR (101 MHz, CDCl$_3$) δ 220.8, 171.5, 148.5, 138.2, 137.6, 126.5, 121.6, 118.8, 50.5, 48.1, 44.3, 38.1, 36.0, 32.7, 31.7, 29.5, 27.8, 26.4, 25.9, 21.7, 13.9.
LRMS (**EI**): *m/z* Calculated for $C_{22}H_{27}BrO_3$ ([M]$^+$): 418.1; found: 418.2:

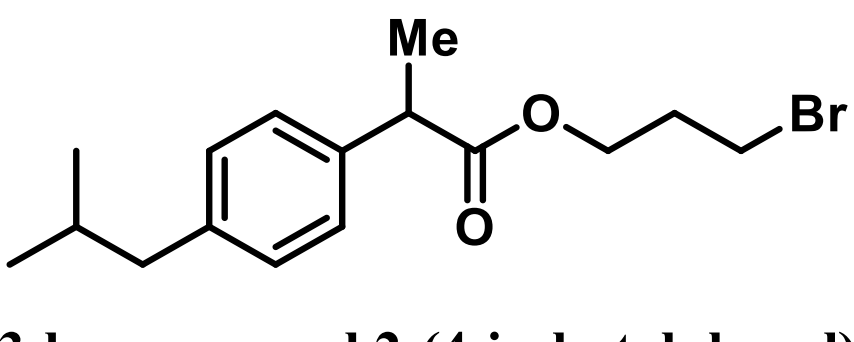

3-bromopropyl 2-(4-isobutylphenyl)propanoate (S8)[5]: Following the general procedure on a 1.5 mmol scale, substrate **S8** was obtained as a colorless oil (420.0 mg, 91% yield).
^{1}H NMR (400 MHz, CDCl$_3$) δ 7.22 – 7.16 (m, 2H), 7.13 – 7.06 (m, 2H), 4.28 – 4.12 (m, 2H), 3.69 (q, *J* = 7.2 Hz, 1H), 3.27 (dd, *J* = 6.9, 6.3 Hz, 2H), 2.45 (d, *J* = 7.2 Hz, 2H), 2.10 (p, *J* = 6.3 Hz, 2H), 1.84 (dq, *J* = 13.6, 6.8 Hz, 1H), 1.49 (d, *J* = 7.2 Hz, 3H), 0.90 (d, *J* = 6.6 Hz, 6H).
^{13}C NMR (101 MHz, CDCl$_3$) δ 174.7, 140.8, 137.8, 129.5, 127.2, 62.4, 45.24, 45.15, 31.8, 30.3, 29.4, 22.5, 18.4.
LRMS (**EI**): *m/z* Calculated for $C_{16}H_{23}BrO_2$ ([M]$^+$): 326.1; found: 326.1.

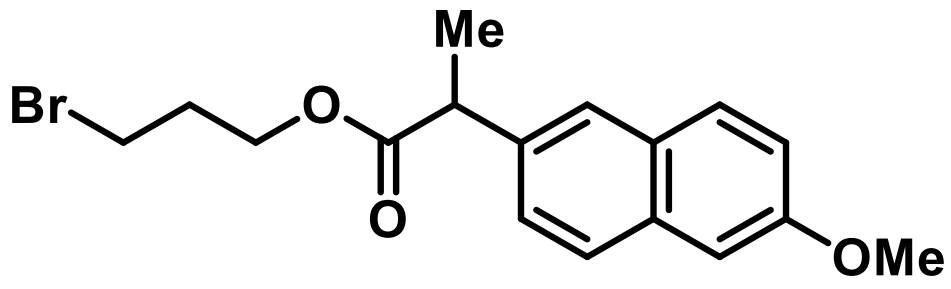

3-bromopropyl 2-(6-methoxynaphthalen-2-yl)propanoate (S9)[3]: Following the general procedure on a 2 mmol scale, substrate **S9** was obtained as a white solid (547.8 mg, 78% yield).

^{1}H NMR (400 MHz, CDCl$_3$) δ 7.71 (d, *J* = 8.6 Hz, 2H), 7.66 (s, 1H), 7.39 (dd, *J* = 8.5, 1.8 Hz, 1H), 7.15 (dd, *J* = 8.8, 2.5 Hz, 1H), 7.12 (d, *J* = 2.5 Hz, 1H), 4.29 – 4.15 (m, 2H), 3.92 (s, 3H), 3.86 (q, *J* = 7.1 Hz, 1H), 3.29 (td, *J* = 6.6, 1.8 Hz, 2H), 2.10 (p, *J* = 6.3 Hz, 2H), 1.58 (d, *J* = 7.1 Hz, 3H).

^{13}C NMR (101 MHz, CDCl$_3$) 174.6, 157.8, 135.7, 133.8, 129.4, 129.1, 127.3, 126.3, 126.0, 119.2, 105.7, 62.5, 55.5, 45.6, 31.7, 29.4, 18.6.

LRMS (**EI**): *m/z* Calculated for $C_{17}H_{19}BrO_3$ ([M]$^+$): 350.1; found: 350.1.

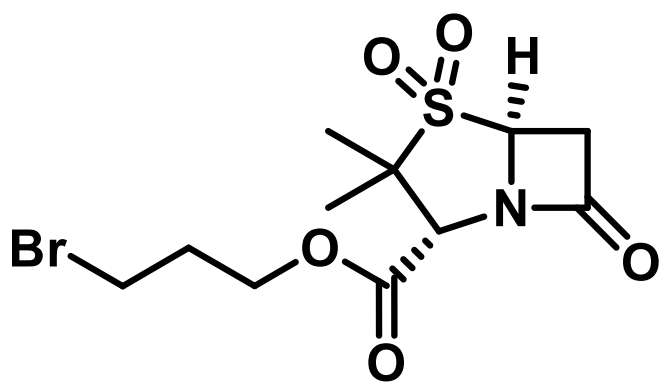

3-bromopropyl (2R,5S)-3,3-dimethyl-7-oxo-4-thia-1-azabicyclo[3.2.0]heptane-2-carboxylate 4,4-dioxide (S10): Following the general procedure on a 2 mmol scale, substrate **S10** was obtained as a white solid (603.5 mg, 85% yield).

^{1}H NMR (400 MHz, CDCl$_3$) δ 4.61 (dd, *J* = 4.2, 2.2 Hz, 1H), 4.51 – 4.28 (m, 3H), 3.67 – 3.34 (m, 4H), 2.34 – 2.13 (m, 2H), 1.60 (s, 3H), 1.42 (s, 3H).

^{13}C NMR (101 MHz, CDCl$_3$) δ 170.8, 167.0, 64.2, 63.3, 62.8, 61.2, 38.5, 31.1, 28.9, 20.5, 18.8.

HRMS (**ESI**): *m/z* Calculated for $C_{11}H_{16}BrNO_5S$ ([M+H]$^+$): 354.0005; found: 354.0006.

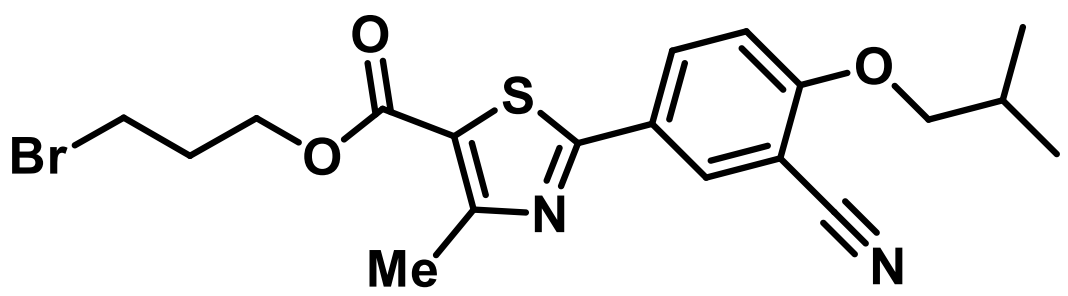

3-bromopropyl 2-(3-cyano-4-isobutoxyphenyl)-4-methylthiazole-5-carboxylate (S11)[3]: Following the general procedure on a 2 mmol scale, substrate **S11** was obtained as a white solid (678.8 mg, 78% yield).

^{1}H NMR (400 MHz, CDCl$_3$) δ 8.18 (d, *J* = 2.3 Hz, 1H), 8.10 (dd, *J* = 8.9, 2.3 Hz, 1H), 7.01 (d, *J* = 8.9 Hz, 1H), 4.45 (t, *J* = 6.0 Hz, 2H), 3.90 (d, *J* = 6.4 Hz, 2H), 3.54 (t, *J* = 6.5 Hz, 2H), 2.77 (s, 3H), 2.31 (p, *J* = 6.3 Hz, 2H), 2.19 (dt, *J* = 13.1, 6.6 Hz, 1H), 1.09 (d, *J* = 6.7 Hz, 6H).

^{13}C NMR (101 MHz, CDCl$_3$) δ 167.6, 162.7, 161.9, 161.7, 132.7, 132.3, 126.0, 121.4, 115.5, 112.8, 103.2, 75.9, 63.1, 31.8, 29.3, 28.3, 19.2, 17.7.

HRMS (**ESI**): *m/z* Calculated for $C_{19}H_{21}BrN_2O_3S$ ([M+H]$^+$):437.0529; found: 437.0513.

3. Reaction Optimization and Control Experiments

3.1 Procedures for the optimization of the reaction conditions

Unless specified otherwise, a mixture of an electron acceptor substrate, e.g., 1-isoquinolinecarbonitrile (0.1 mmol), electron donor reagent $(TMS)_3SiNHPh$ (0.16 mmol), alkyl bromide, eg., bromocyclohexane (0.16 mmol) and additive, eg., NEt_3 (0.1 mmol) was added to an oven-dried 5 mL snap vial equipped with a magnetic stirring bar. The capped vial was evacuated and backfilled with N_2 three times, and 1 mL of solvent (DMA or other organic solvents) was added to the vial by syringe. Then, the cap was sealed with parafilm. The reaction mixture was stirred vigorously and irradiated with a 365 nm (3.0 w or 500 mw) LED (for the screening of the light sources, 700 mW of 340nm, 365 nm, 385 nm, 300-395 nm, 400-405 nm, 451 nm LEDs were used) at 25 °C (for the control experiments, reactions were also conducted under 55°C and 5°C) for 3 h. Then diluted with ethyl acetate (EA, 2 mL) and filtered through a plug of celite (Ø 3 mm × 8 mm). After addition of n-dodecane (0.1 mmol) as an internal standard for calibration, the product yield was determined by GC-FID.

3.2 Optimization of Coupling reaction

Table S1. Aminosilane Screening

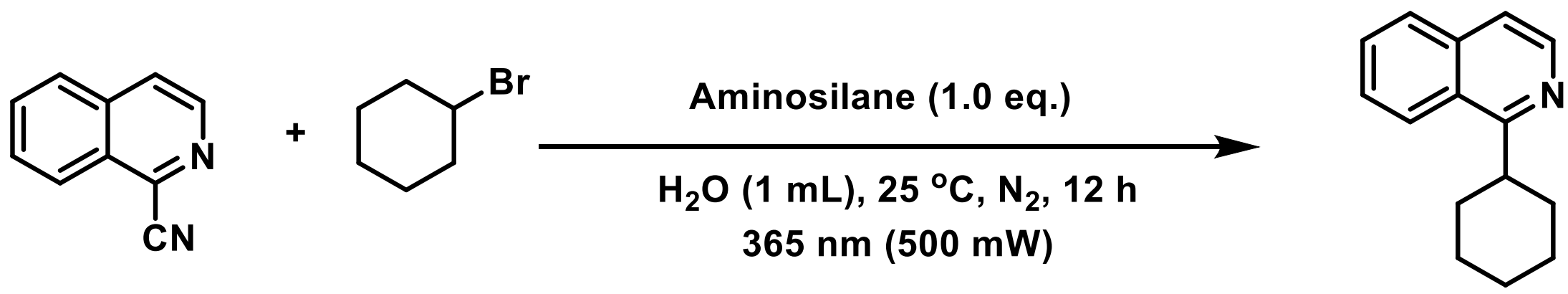

1, 0.125 mmol **2, 0.1 mmol**

Entry	Aminosilane	Yield[a]
1	$(TMS)_3SiNHPh$ (3)	17
2	$(TMS)_3SiNHNaph$ (S1)	15

Standard reaction conditions: **1** (0.125 mmol), **2** (0.1 mmol), aminosilane (1.0 eq.) in the presence of 1 mL H_2O under 365 nm LED (500 mW) light irradiation at 25 °C under N_2 atmosphere for 12 h. [a]Yields are determined by ^1H NMR with CH_2Br_2 as inert standard.

Table S2. Solvent Screening

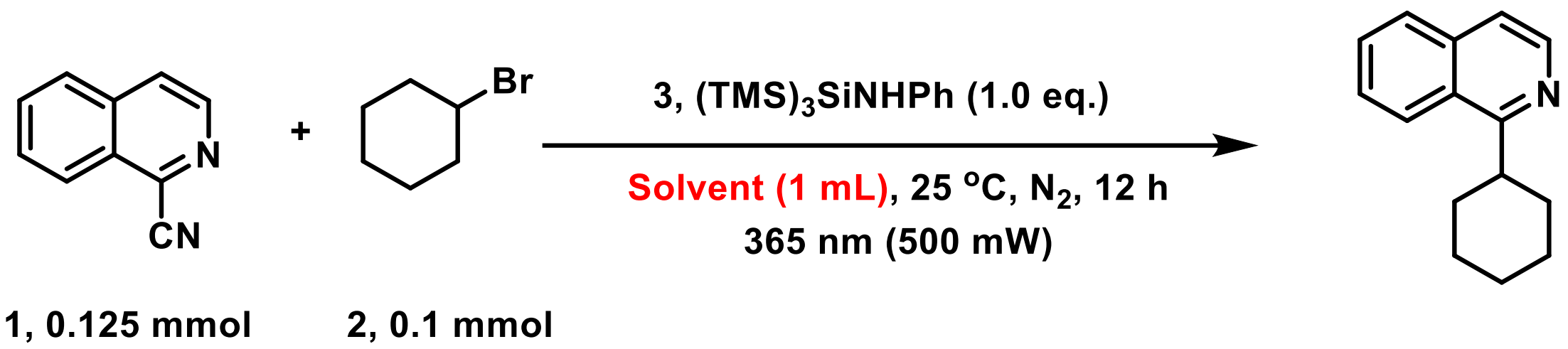

Entry	Solvent	Yield[a] (%)
1	H_2O	17
2	DMF	38
3	DMSO	10
4	DCM	4
5	MeCN	24
6	THF	trace
7	Acetone	26
8	MeOH	18
9	Toulune	16
10	DMA	17
11	$CHCl_3$	trace
12	MTBE	33
13	2-propanol	23

Standard reaction conditions: **1** (0.125 mmol), **2** (0.1 mmol), $(TMS)_3SiNHPh$ (1.0 eq.) in the presence of 1 mL Solvent under 365 nm LED (500 mW) light irradiation at 25 °C under N_2 atmosphere for 12 h. [a]Yields are determined by GC-FID with 0.1 mmol n-dodecane as standard.

Table S3. Additive Screening

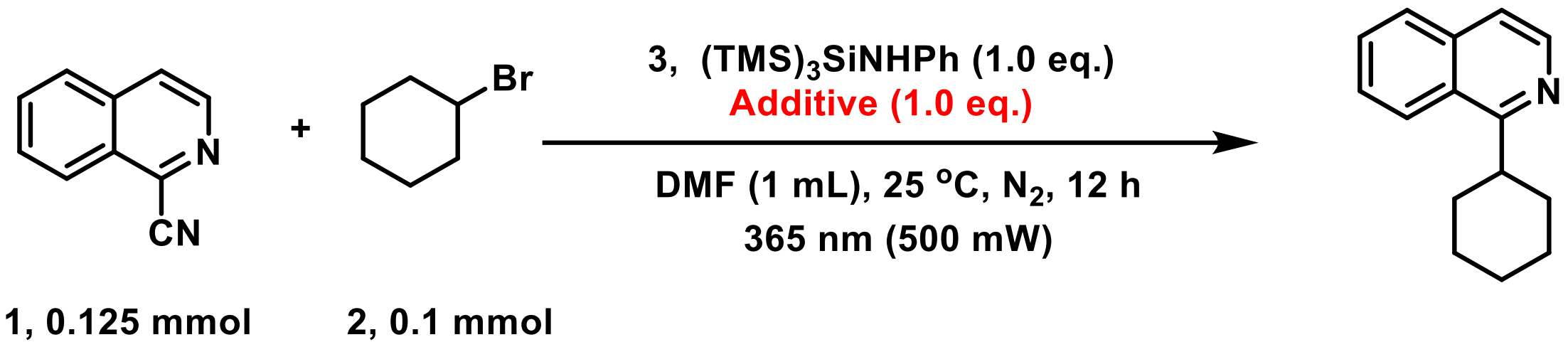

Entry	Additive	Yield[a] (%)
1	none	38
2	DIPEA	50
3	Cs_2CO_3	15
4	Pyridine	29
5	DABCO	32
6	NEt_3	47
7	Quinuclidine	52
8	BTMG	25

Standard reaction conditions: **1** (0.125 mmol), **2** (0.1 mmol), $(TMS)_3SiNHPh$ (1.0 eq.) and Additive (1.0 eq.) in the presence of 1 mL DMF under 365 nm LED (500 mW) light irradiation at 25 °C under N_2 atmosphere for 12 h. [a]Yields are determined by GC-FID with 0.1 mmol n-dodecane as standard.

Table S4. LED Screening

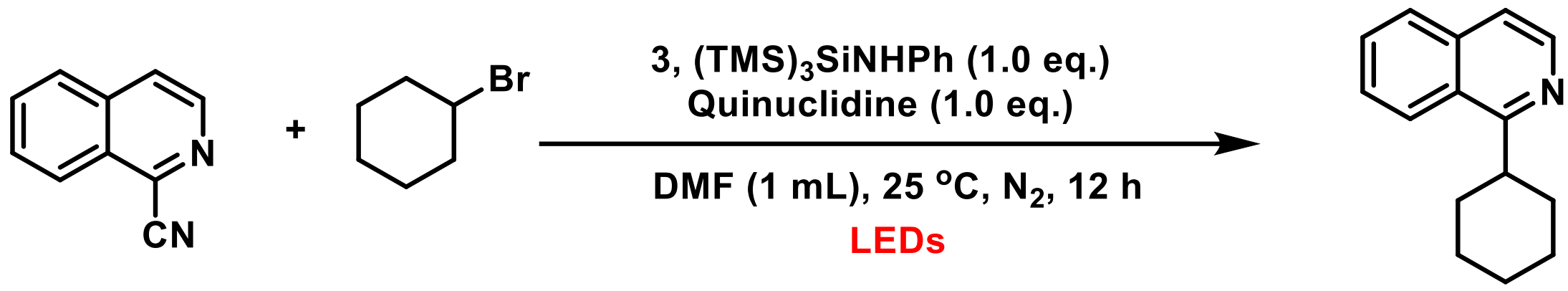

1, 0.125 mmol **2, 0.1 mmol**

Entry	LEDs	Yield[a] (%)
1	340 nm	38
2	365 nm	52
3	385 nm	39
4	390-395 nm	47
5	400-405 nm	24
6	451 nm	21
7	365 nm (3W)	56

Standard reaction conditions: **1** (0.125 mmol), **2** (0.1 mmol), $(TMS)_3SiNHPh$ (1.0 eq.) and Quinuclidine (1.0 eq.) in the presence of 1 mL DMF under different LEDs (500 mW) light irradiation at 25 °C under N_2 atmosphere for 12 h. [a]Yields are determined by GC-FID with 0.1 mmol n-dodecane as standard.

Table S5. Concentration Screening

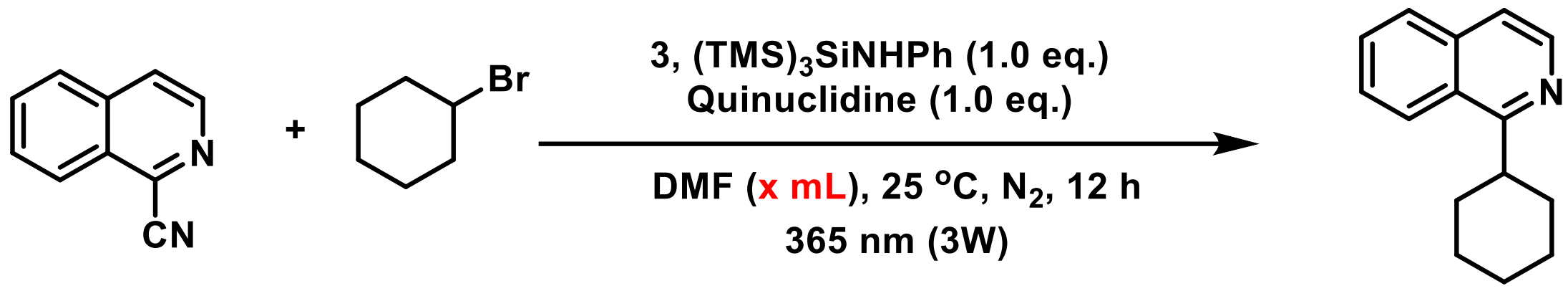

1, 0.125 mmol **2, 0.1 mmol**

Entry	DMF (x mL)	Yield[a] (%)
1	0.1	43
2	0.2	42
3	0.5	41
4	1.0	56
5	1.5	51
6	2.0	42

Standard reaction conditions: **1** (0.125 mmol), **2** (0.1 mmol), $(TMS)_3SiNHPh$ (1.0 eq.) and Quinuclidine (1.0 eq.) in the presence of x mL DMF under 365 nm LEDs light irradiation at 25 °C under N_2 atmosphere for 12 h. [a]Yields are determined by GC-FID with 0.1 mmol n-dodecane as standard.

Table S6. Temperature Screening

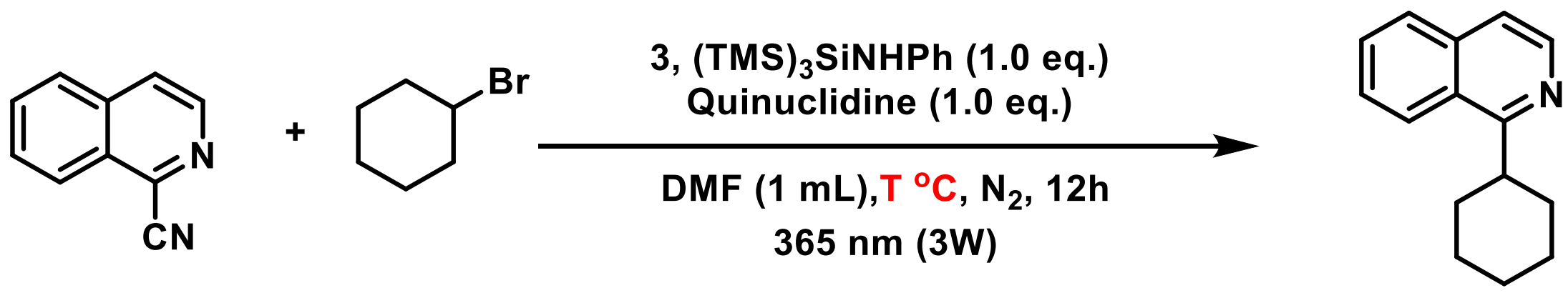

1, 0.125 mmol **2, 0.1 mmol**

Entry	T (°C)	Yield[a] (%)
1	5	38
2	25	56
3	55	44

Standard reaction conditions: **1** (0.125 mmol), **2** (0.1 mmol), $(TMS)_3SiNHPh$ (1.0 eq.) and Quinuclidine (1.0 eq.) in the presence of 1 mL DMF under 365 nm LEDs light irradiation at T °C under N_2 atmosphere for 12 h. [a]Yields are determined by GC-FID with 0.1 mmol n-dodecane as standard.

Table S7. Reaction time Screening

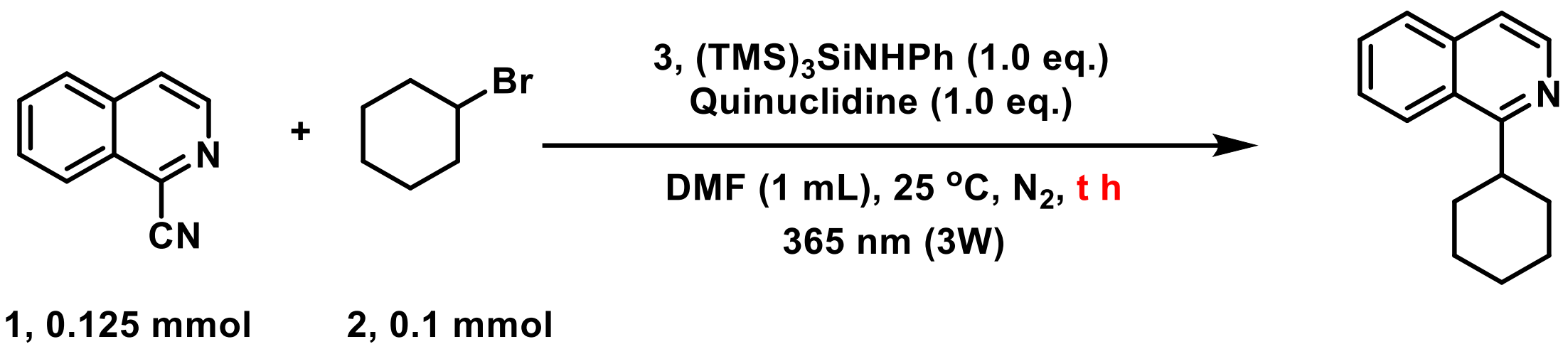

Entry	t h	Yield[a] (%)
1	3	56
2	6	56
3	12	56
4	18	57
5	24	38
6[b]	1	43

Standard reaction conditions: **1** (0.125 mmol), **2** (0.1 mmol), $(TMS)_3SiNHPh$ (1.0 eq.) and Quinuclidine (1.0 eq.) in the presence of 1 mL DMF under 365 nm LEDs light irradiation at 25 °C under N_2 atmosphere for t h. [a]Yields are determined by GC-FID with 0.1 mmol n-dodecane as standard. [b]DMA as Solvent and NEt_3 as additive.

Table S8. Screening of the ratio of Bromocyclohexane

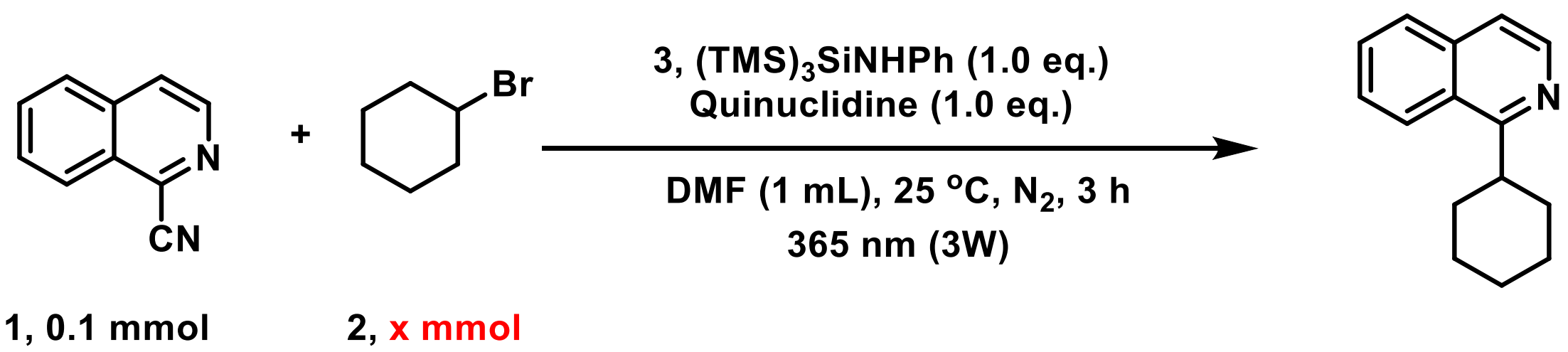

Entry	2, x (mmol)	Yield[a] (%)
1	0.1	55
2	0.12	56
3	0.14	42
4	0.16	57
5	0.18	47
6	0.2	39

Standard reaction conditions: **1** (0.1 mmol), **2** (x mmol), $(TMS)_3SiNHPh$ (1.0 eq.) and Quinuclidine (1.0 eq.) in the presence of 1 mL DMF under 365 nm LEDs light irradiation at 25 °C under N_2 atmosphere for 3 h. [a]Yields are determined by GC-FID with 0.1 mmol n-dodecane as standard.

Table S9. Solvent Screening

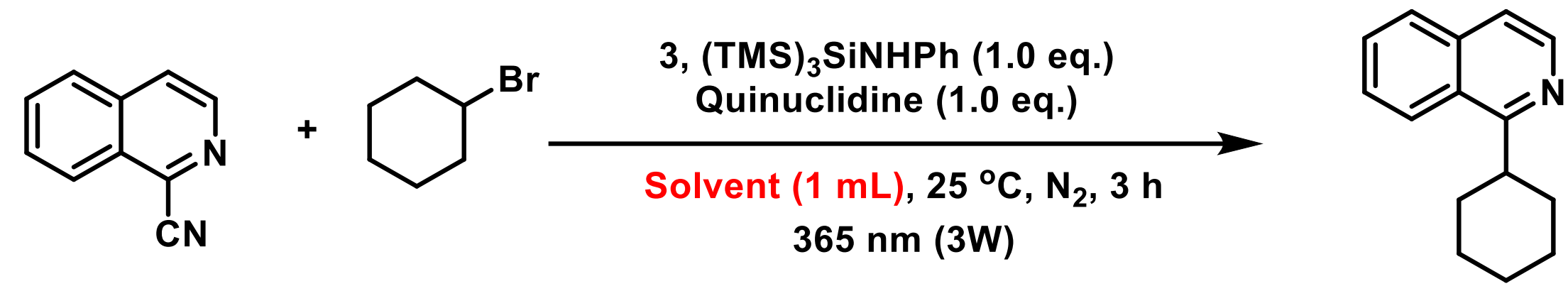

1, 0.1 mmol **2**, 0.16 mmol

Entry	Solvent	Yield[a] (%)
1	H_2O	12
2	Chlorobenzene	24
3	DMF	57
4	DMA	58
5	Acetone	44
6	MeCN	17
7	Toulune	29
8	EA	n.d.
9	DMSO	37

Standard reaction conditions: **1** (0.1 mmol), **2** (0.16 mmol), $(TMS)_3SiNHPh$ (1.0 eq.) and Quinuclidine (1.0 eq.) in the presence of 1 mL Solvent under 365 nm LEDs light irradiation at 25 °C under N_2 atmosphere for 3 h. [a]Yields are determined by GC-FID with 0.1 mmol n-dodecane as standard.

Table S10. Additive Screening

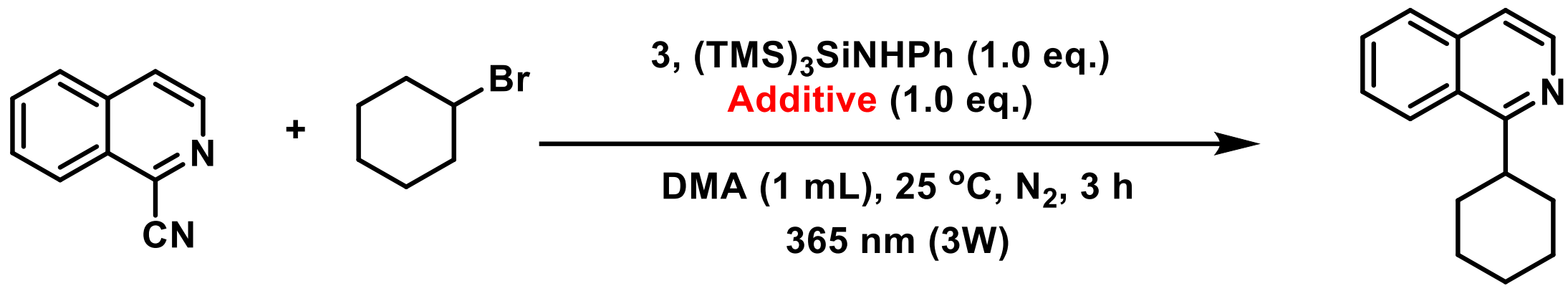

1, 0.1 mmol **2, 0.16 mmol**

Entry	Additive	Yield[a] (%)
1	Quinuclidine	58
2	DIPEA	50
3	TMG	39
4	NEt_3	58
5	none	40

Standard reaction conditions: **1** (0.1 mmol), **2** (0.16 mmol), $(TMS)_3SiNHPh$ (1.0 eq.) and Additive (1.0 eq.) in the presence of 1 mL DMA under 365 nm LEDs light irradiation at 25 °C under N_2 atmosphere for 3 h. [a]Yields are determined by GC-FID with 0.1 mmol n-dodecane as standard.

Table S11. Screening of the ratio of Additive

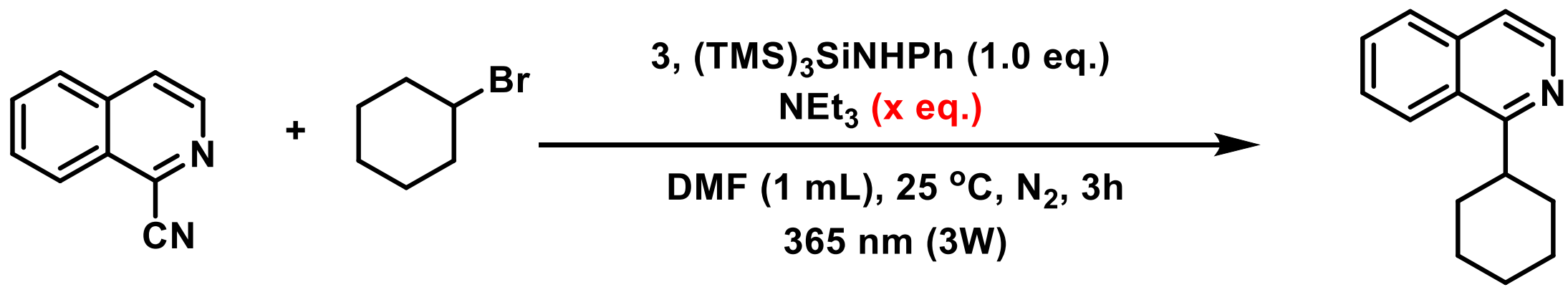

1, 0.1 mmol **2, 0.16 mmol**

Entry	NEt_3 (x eq.)	Yield[a] (%)
1	1.0	58
2	1.1	48
3	1.2	43
4	1.3	52
5	1.4	45
6	1.5	41

Standard reaction conditions: **1** (0.1 mmol), **2** (0.16 mmol), $(TMS)_3SiNHPh$ (1.0 eq.) and NEt_3 (x eq.) in the presence of 1 mL DMA under 365 nm LEDs light irradiation at 25 °C under N_2 atmosphere for 3 h. [a]Yields are determined by GC-FID with 0.1 mmol n-dodecane as standard.

Table S12. Screening of the ratio of Aminosilane

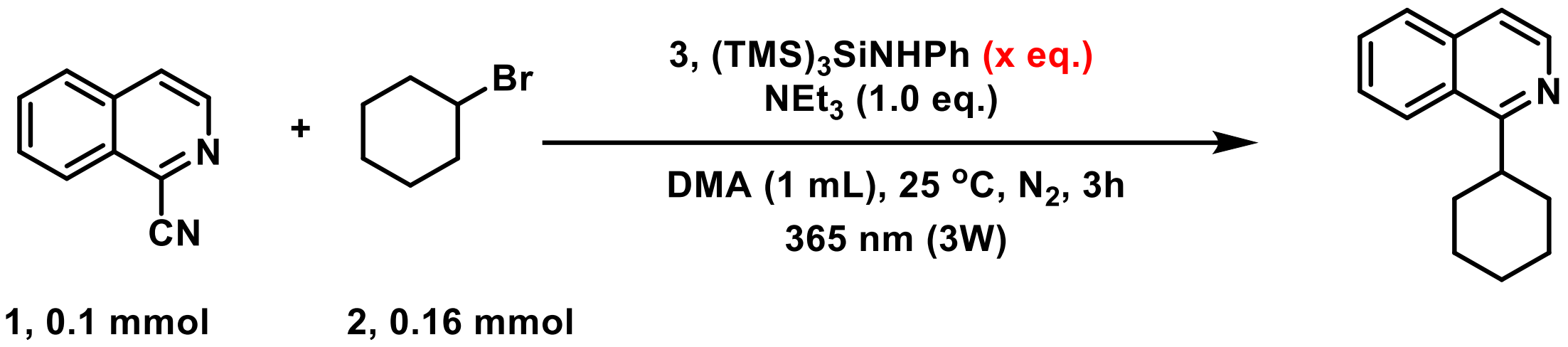

Entry	$(TMS)_3SiNHPh$ (x eq.)	Yield[a] (%)
1	1.0	58
2	1.1	56
3	1.2	51
4	1.3	56
5	1.4	56
6	1.5	48
7	1.6	62
8	1.7	53
9	1.8	61
10	1.9	57
11	2.0	48

Standard reaction conditions: **1** (0.1 mmol), **2** (0.16 mmol), $(TMS)_3SiNHPh$ (x eq.) and NEt_3 (1.0 eq.) in the presence of 1 mL DMA under 365 nm LEDs light irradiation at 25 °C under N_2 atmosphere for 3 h. [a]Yields are determined by GC-FID with 0.1 mmol n-dodecane as standard.

Table S13. Control experiment for the reaction

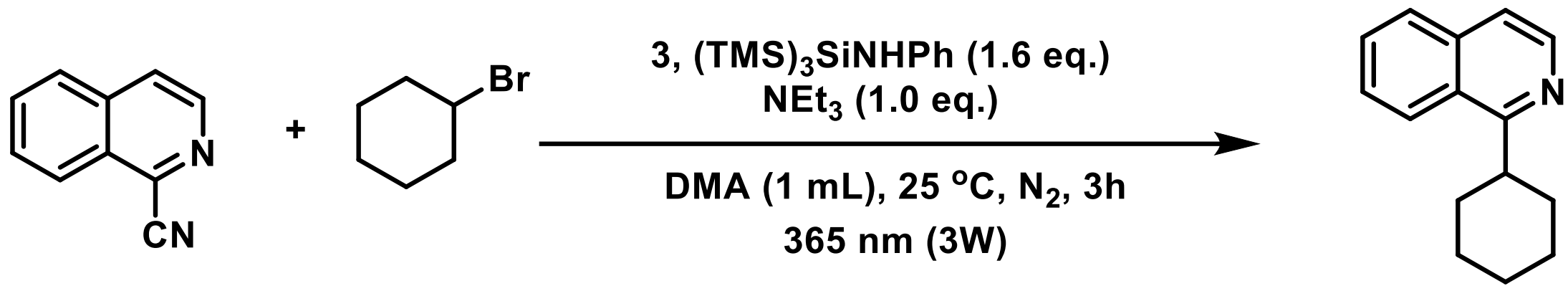

1, 0.1 mmol **2, 0.16 mmol**

Entry	conditions	Yield[a] (%)
1	none	62(60)
2	no light	0
3	no aminosilane	<5
4	no aminosilane and NEt_3	<5
5	no NEt_3	54
6	TTMSS as Si reagent	<5
7	$AdNHSi(TMS)_3$ as Si reagent	39
8	under air	44
9	at 55 °C	43

Standard reaction conditions: **1** (0.1 mmol), **2** (0.16 mmol), $(TMS)_3SiNHPh$ (1.6 eq.) and NEt_3 (1.0 eq.) in the presence of 1 mL DMA under 365 nm LEDs light irradiation at 25 °C under N_2 atmosphere for 3 h. [a]Yields are determined by GC-FID with 0.1 mmol n-dodecane as standard. Yields in parentheses are isolated yields

3.3 Optimization of Minisci type reaction

Table S14. Solvent Screening

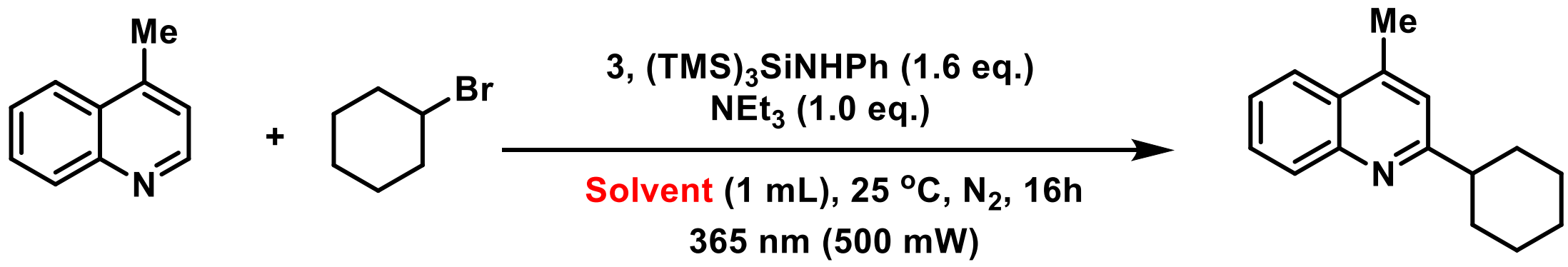

5, 0.1 mmol **2, 0.15 mmol**

Entry	Solvent	Yield[a] (%)
1	DMA	39
2	DMF	34
3	MeCN	29
4	DMSO	37
5	EA	18
6	Acetone	18
7	THF	13
8	Toluene	10
9	DCE	13
10	MeOH	14

Standard reaction conditions: **5** (0.1 mmol), **2** (0.15 mmol), $(TMS)_3SiNHPh$ (1.6 eq.) and NEt_3 (1.0 eq.) in the presence of 1 mL DMA under 365 nm LEDs light irradiation at 25 °C under N_2 atmosphere for 16 h. [a]Yields are determined by GC-FID with 0.1 mmol n-dodecane as standard.

Table S15. Additive Screening

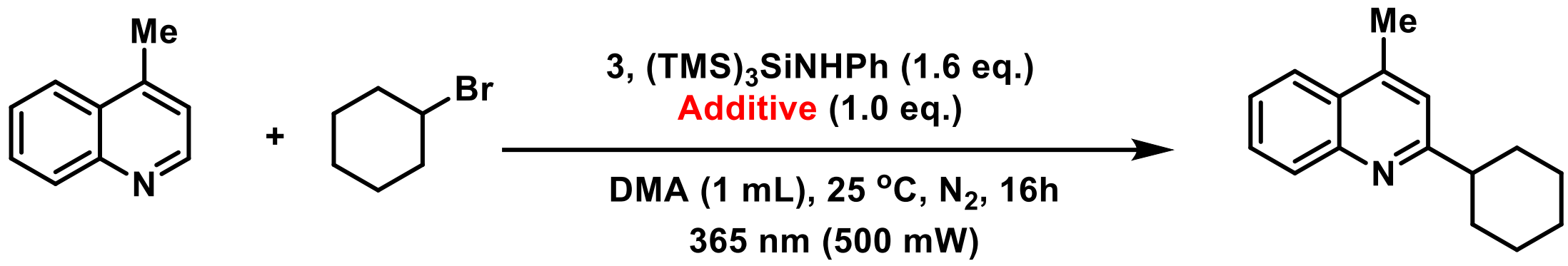

5, 0.1 mmol **2, 0.15 mmol**

Entry	Additive	Yield[a] (%)
1	TFA	27
2	DIPEA	36
3	Quinuclidine	31
4	DABCO	27
5	BTMG	29
6	Na_2CO_3	27
7	K_3PO_4	34
8	Cs_2CO_3	35
9	NEt_3 (0.75 eq)	38
10	NEt_3 (1.5 eq)	37
11	NEt_3 (2 eq)	29
12	NEt_3	39

Standard reaction conditions: **5** (0.1 mmol), **2** (0.15 mmol), $(TMS)_3SiNHPh$ (1.6 eq.) and Additive (1.0 eq.) in the presence of 1 mL DMA under 365 nm LEDs light irradiation at 25 °C under N_2 atmosphere for 16 h. [a]Yields are determined by GC-FID with 0.1 mmol n-dodecane as standard.

Table S16. Screening of the ratio of Starting materials

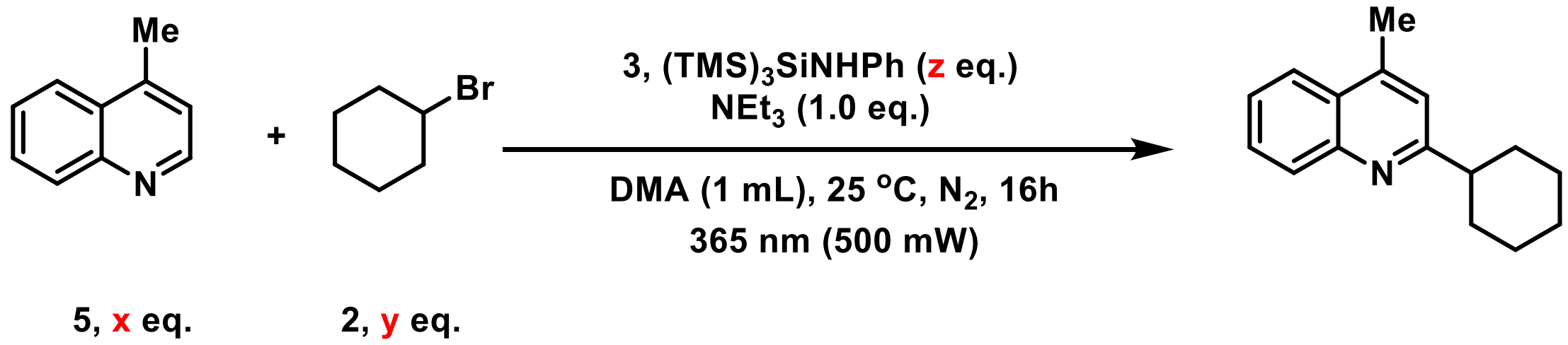

Entry	x (eq.)	y (eq.)	z (eq.)	Yield[a] (%)
1	1	1.5	2	37
2	1	2	1.6	38
3	1	2	2	36
4	1.5	1	1.6	44
5	1.5	1	2	45

Standard reaction conditions: **5** (x equiv.), **2** (y equiv.), $(TMS)_3SiNHPh$ (z equiv.) and NEt_3 (1.0 eq.) in the presence of 1 mL DMA under 365 nm LEDs light irradiation at 25 °C under N_2 atmosphere for 16 h. [a]Yields are determined by GC-FID with 0.1 mmol n-dodecane as standard.

Table S17. Additive Screening

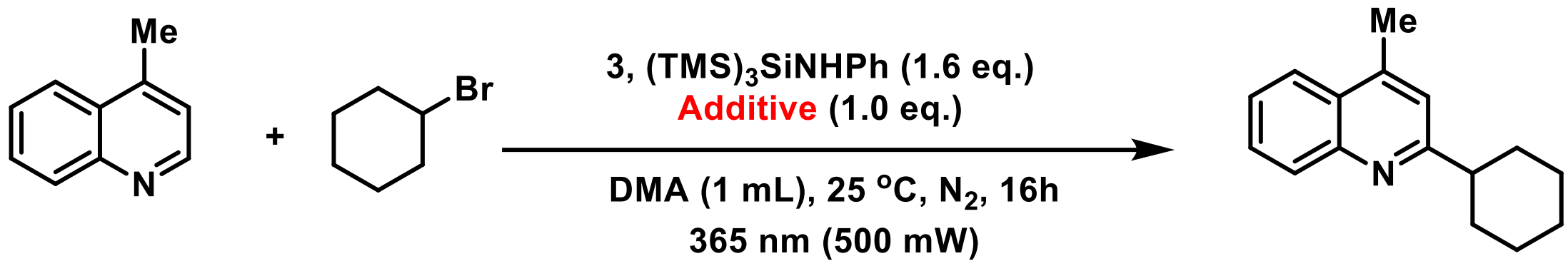

5, 0.15 mmol 2, 0.1 mmol

Entry	Additive	Yield[a] (%)
1	DMAP	28
2	PPh_3	36
3	NPh_3	48
4	TMEDA	37
5	N(*n*-Bu)$_3$	42
6	NH(*i*-Pr)$_2$	42
7	NPh_3	47[b]
8	NPh_3	5[c]

Standard reaction conditions: **5** (0.15 mmol), **2** (0.1 mmol), (TMS)$_3$SiNHPh (1.6 eq.) and Additive (1.0 eq.) in the presence of 1 mL DMA under 365 nm LEDs light irradiation at 25 °C under N_2 atmosphere for 16 h. [a]Yields are determined by GC-FID with 0.1 mmol n-dodecane as standard. [b]With 1.2 equiv. (TMS)$_3$SiNHPh. [c]Without (TMS)$_3$SiNHPh.

Table S18. LED Screening

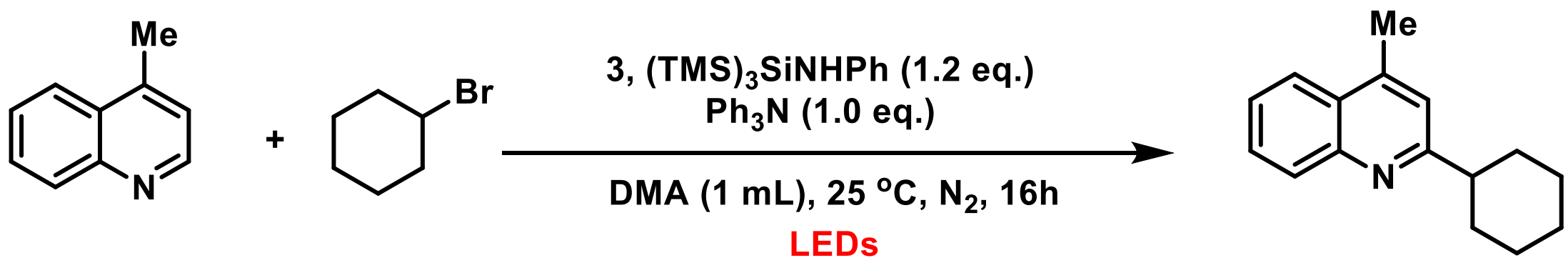

5, 0.15 mmol 2, 0.1 mmol

Entry	LEDs	Yield[a] (%)
1	365 nm (500 mW)	47
2	365 nm (3W)	49 (42)[b]

Standard reaction conditions: **5** (0.15 mmol), **2** (0.1 mmol), (TMS)$_3$SiNHPh (1.2 eq.) and NPh_3 (1.0 eq.) in the presence of 1 mL DMA under 365 nm LEDs light irradiation at 25 °C under N_2 atmosphere for 16 h. [a]Yields are determined by GC-FID with 0.1 mmol n-dodecane as standard. Yields in parentheses are isolated yields [b]Reaction time is 6h.

4. Mechanistic Studies

4.1 UV-vis Absorption Studies

UV-vis absorption spectra of reaction components in DMA showed a new absorption band upon mixing **a** and **b**. **a** (0.03 M), **b** (0.03 M), **a**: **b** = 1:1.

Preparation of the sample for compound 1-CN-isoquinoline (**a**): add compound **a** (0.1 mmol) to a 5 mL vial, followed by adding 3 mL of DMA into the vial. Afterward, filter the sample through a plug of celite (Ø 3 mm × 8 mm) into the cuvette for the UV-vis measurements.

Preparation of the sample for compound $(TMS)_3SiNHPh$ (**b**): add compound **b** (0.1 mmol) to a 5 mL vial, followed by adding 3 mL of DMA into the vial. Afterward, filter the sample through a plug of celite (Ø 3 mm × 8 mm) into the cuvette for the UV-vis measurements.

Preparation of the sample for compound **c**: add compound NEt_3 (**c**) (0.1 mmol) to a 5 mL vial, followed by adding 3 mL of DMA into the vial. Afterward, filter the sample through a plug of celite (Ø 3 mm × 8 mm) into the cuvette for the UV-vis measurements.

Preparation of the binary (**a**+**b**, **a**+**c**, **b**+**c**) and ternary (**a**+**b**+**c**) mixtures: the respective compounds (0.1 mmol) were weighed into a 5 mL vial, followed by adding 3 mL of DMA into the vial. Afterward, filter the sample through a plug of celite (Ø 3 mm × 8 mm) into the cuvette for the UV-vis measurements.

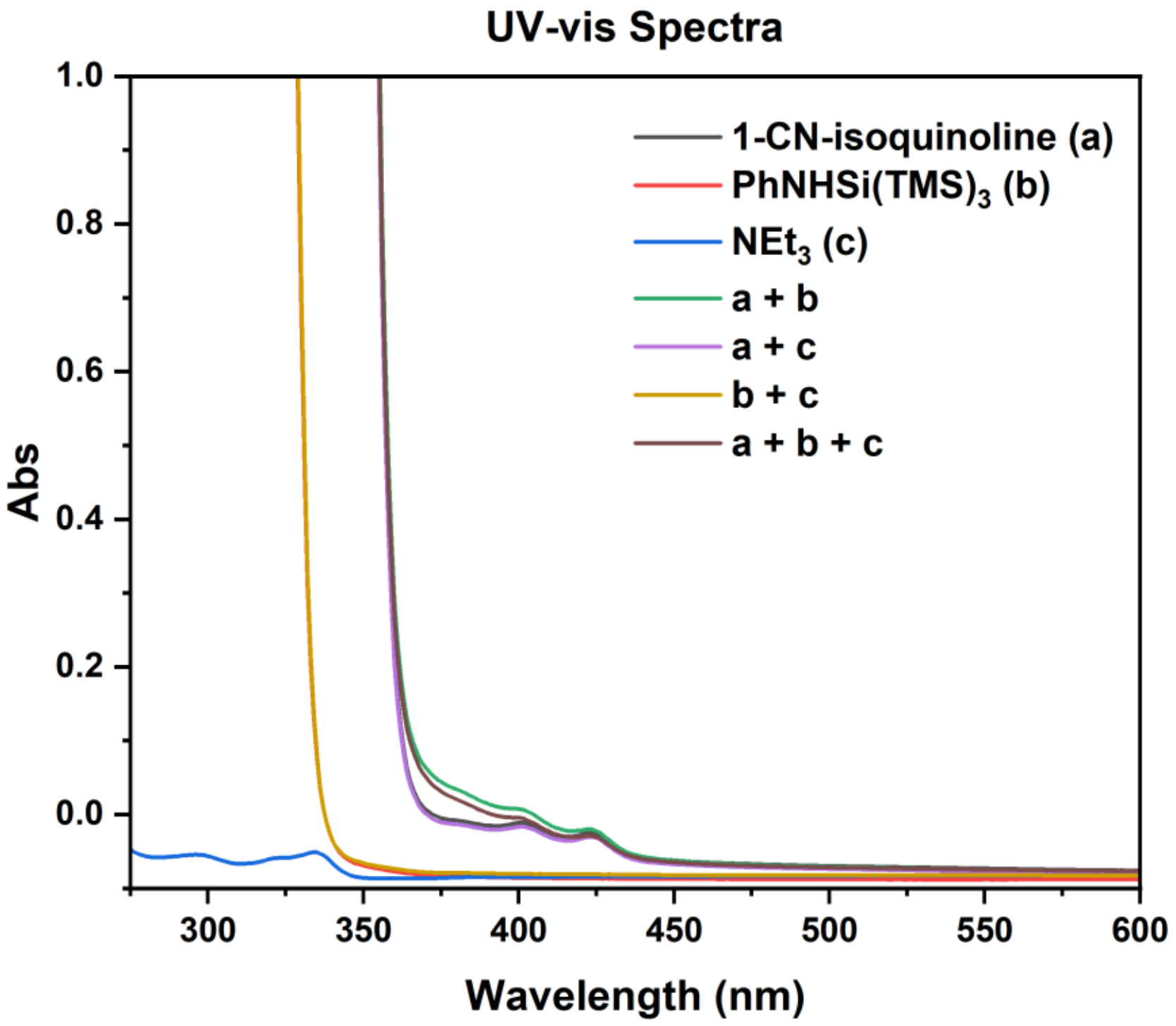

Figure S2. UV-vis Spectra of reaction components

Note: We have observed that $(TMS)_3SiNHPh$ decompose slowly in DMA, measurements should be performed immediately after solution preparation.

UV-vis absorption spectra of Minisci reaction components in DMA showed a new absorption band upon mixing **a'**, **b** and **c'**.

Preparation of the sample for compound 4-Me-quinoline (**a'**): add compound **a'** (0.1 mmol) to a 5 mL vial, followed by adding 3 mL of DMA into the vial. Afterward, filter the sample through a plug of celite (Ø 3 mm × 8 mm) into the cuvette for the UV-vis measurements.

Preparation of the sample for compound $(TMS)_3SiNHPh$ (**b**): add compound **b** (0.1 mmol) to a 5 mL vial, followed by adding 3 mL of DMA into the vial. Afterward, filter the sample through a plug of celite (Ø 3 mm × 8 mm) into the cuvette for the UV-vis measurements.

Preparation of the sample for compound NPh_3 (**c'**): add compound **c'** (0.1 mmol) to a 5 mL vial, followed by adding 3 mL of DMA into the vial. Afterward, filter the sample through a plug of celite (Ø 3 mm × 8 mm) into the cuvette for the UV-vis measurements.

Preparation of the binary (**a'**+**b**, **a'**+**c'**, **b**+**c'**) and ternary (**a'**+**b**+**c'**) mixtures: the respective compounds (0.1 mmol) were weighed into a 5 mL vial, followed by adding 3 mL of DMA into the vial. Afterward, filter the sample through a plug of celite (Ø 3 mm × 8 mm) into the cuvette for the UV-vis measurements.

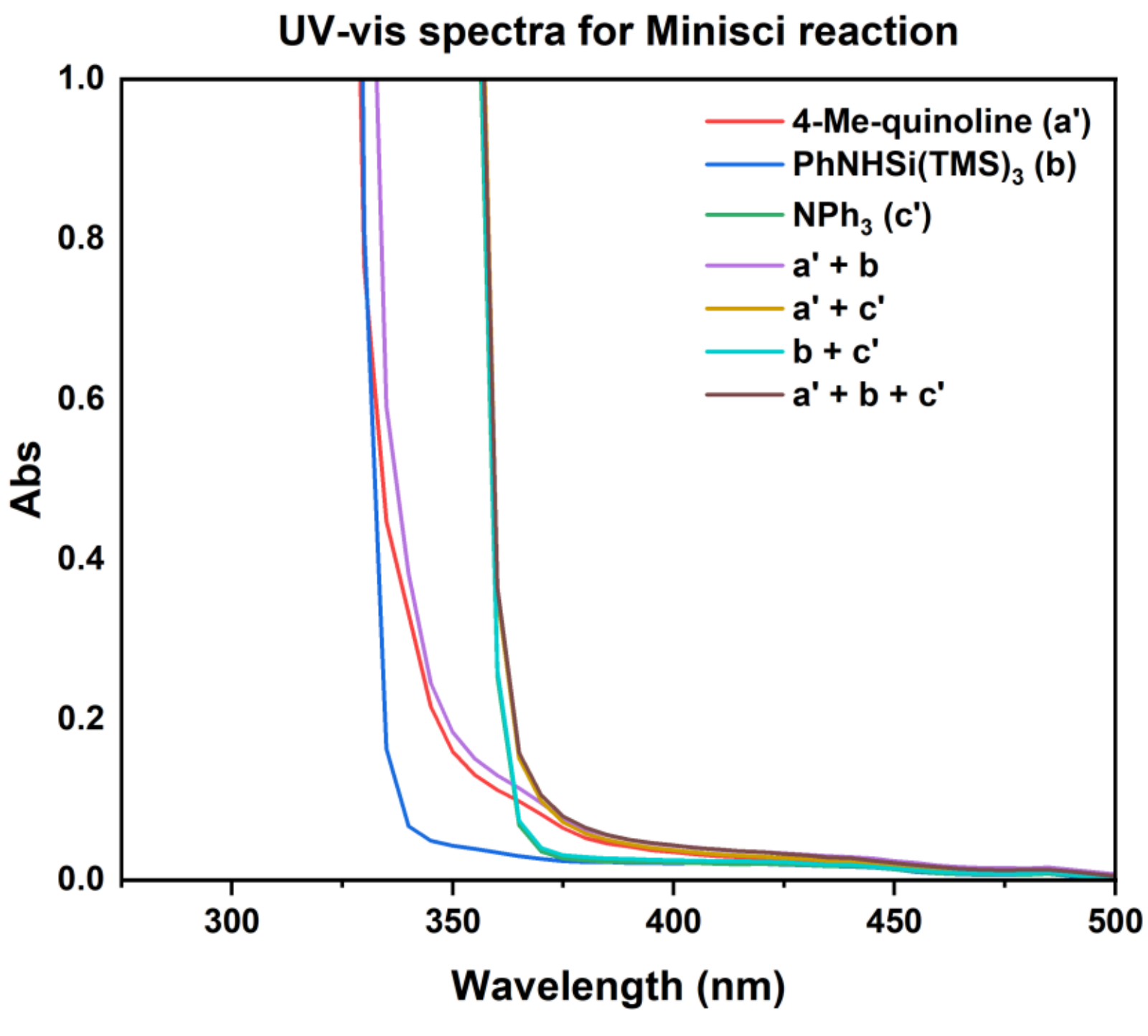

Figure S3. UV-vis Spectra of Minisci reaction

Note: We have observed that $(TMS)_3SiNHPh$ decompose slowly in DMA, measurements should be performed immediately after solution preparation.

4.2 Radical Clock Experiment

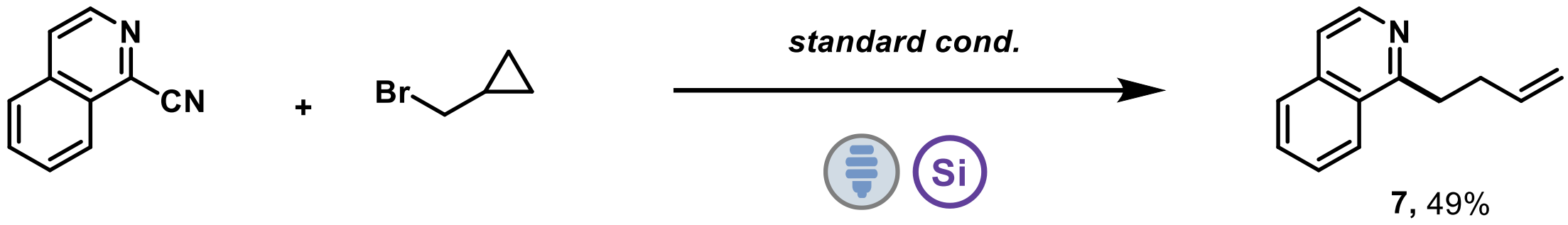

7, 49%

A mixture of 1-isoquinolinecarbonitrile (0.2 mmol), $(TMS_3)_3SiNHPh$ (0.32 mmol) (bromomethyl)cyclopropane (0.32 mmol) and NEt_3 (0.2 mmol) was added to an oven-dried 5 mL snap vial equipped with a magnetic stirring bar. The capped vial was evacuated and back filled with N_2 for three times and 2 mL DMA was added to the vial by syringe. Then, the cap was sealed with parafilm. The reaction mixture was stirred vigorously and irradiated with a 365 nm (3.0 W) LED at 25 °C for 3 h. Then diluted with Na_2CO_3 solution and extracted with Diethylether (3 x 15 mL) After addition of CH_2Br_2 (0.2 mmol) as an internal standard for calibration, the product yield was determined by ^1H NMR. The residue was purified by silica gel flash chromatography to give the desired product as a yellow oil (17.9 mg, 49% isolated yields).

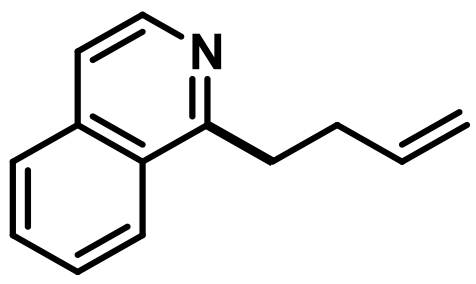

1-(but-3-en-1-yl)isoquinoline (7)[6]

^{1}H NMR (400 MHz, CDCl$_3$) δ 8.44 (d, J = 5.8 Hz, 1H), 8.16 (dd, J = 8.4, 1.2 Hz, 1H), 7.82 (d, J = 8.2 Hz, 1H), 7.67 (ddd, J = 8.2, 6.9, 1.2 Hz, 1H), 7.60 (ddd, J = 8.3, 6.8, 1.4 Hz, 1H), 7.52 (d, J = 5.7 Hz, 1H), 5.99 (ddt, J = 16.9, 10.2, 6.6 Hz, 1H), 5.11 (dq, J = 17.1, 1.7 Hz, 1H), 5.01 (dq, J = 10.1, 1.3 Hz, 1H), 3.44 – 3.34 (m, 2H), 2.70 – 2.59 (m, 2H).

^{13}C NMR (101 MHz, CDCl$_3$) δ 161.4, 141.8, 138.1, 136.4, 130.0, 127.6, 127.2, 127.1, 125.4, 119.5, 115.2, 34.7, 33.6.

LRMS (EI): *m/z* Calculated for $C_{13}H_{13}N$ ($[M]^+$): 183.1; found:183.1.

4.3 Radical Capturing Experiment

4.3.1 Using TEMPO

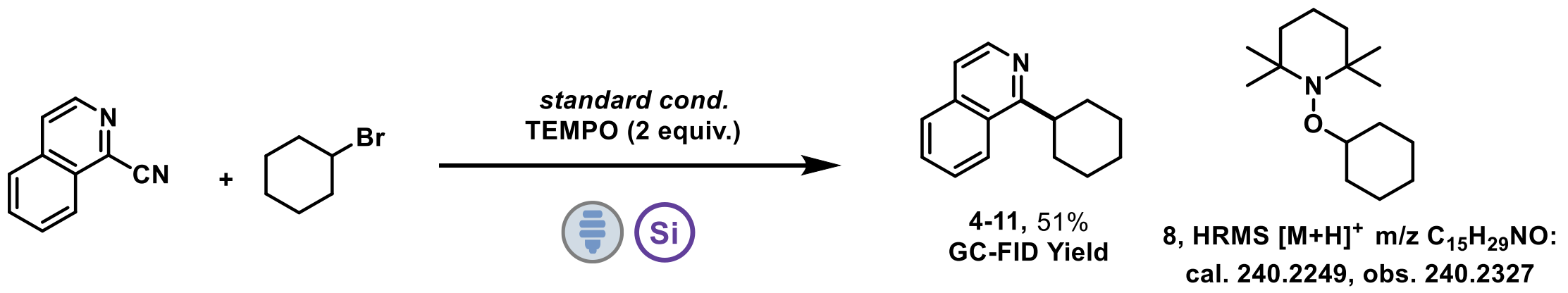

A mixture of 1-isoquinolinecarbonitrile (0.2 mmol), $(TMS_3)_3SiNHPh$ (0.32 mmol) (bromomethyl)cyclopropane (0.32 mmol), NEt_3 (0.2 mmol) and TEMPO (0.4 mmol) was added to an oven-dried 5 mL snap vial equipped with a magnetic stirring bar. The capped vial was evacuated and backfilled with N_2 three times and 2 mL DMA was added to the vial by syringe. Then, the cap was sealed with parafilm. The reaction mixture was stirred vigorously and irradiated with a 365 nm (3.0 W) LED at 25 °C for 3 h. Then diluted with Na_2CO_3 solution and extracted with Diethyl Ether (3 x 15 mL). After the addition of n-dodecane (0.2 mmol) as an internal standard for calibration, the product yield was determined by GC-FID. TEMPO capturing product **8** was detected by **HRMS (ESI):** *m/z* Calculated for $C_{15}H_{29}NO$ ($[M+H]^+$): 240.2322; found: 240.2327.

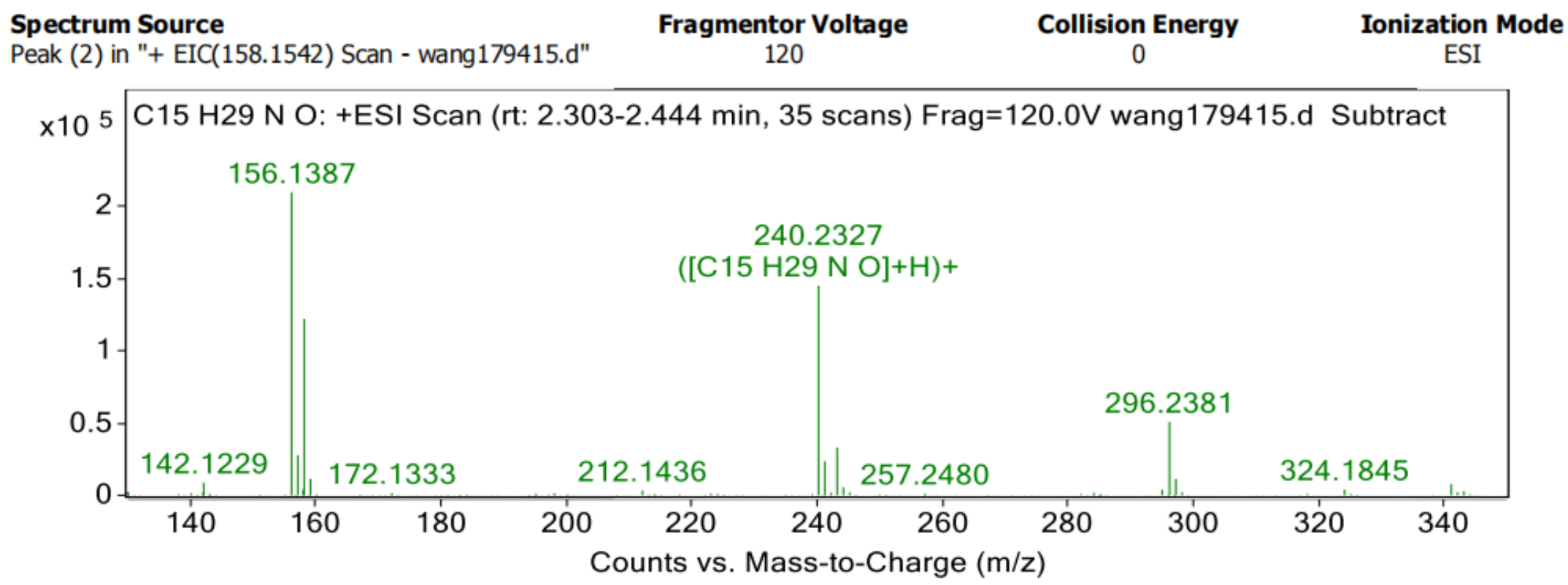

Figure S4. HRMS (ESI) reports of the adducts obtained.

4.3.2 Using BHT

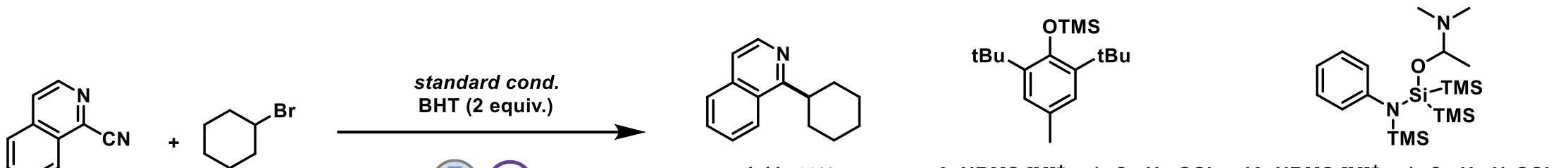

4-11, 46% GC-FID Yield

9, HRMS $[M]^+$ m/z $C_{18}H_{32}OSi$ cal.292.2222, obs. 292.2214

10, HRMS $[M]^+$ m/z $C_{19}H_{42}N_2OSi_4$ cal. 426.2374, obs. 426.2380

A mixture of 1-isoquinolinecarbonitrile (0.2 mmol), $(TMS_3)_3SiNHPh$ (0.32 mmol) (bromomethyl)cyclopropane (0.32 mmol), NEt_3 (0.2 mmol) and BHT (0.4 mmol) was added to an oven-dried 5 mL snap vial equipped with a magnetic stirring bar. The capped vial was evacuated and backfilled with N_2 three times and 2 mL DMA was added to the vial by syringe. Then, the cap was sealed with parafilm. The reaction mixture was stirred vigorously and irradiated with a 365 nm (3.0 W) LED at 25 °C for 3 h. Then diluted with Na_2CO_3 solution and extracted with Diethyl Ether (3 x 15 mL). After the addition of n-dodecane (0.2 mmol) as an internal standard for calibration, the product yield was determined by GC-FID.

BHT capturing product **9** was detected by **HRMS (EI):** *m/z* Calculated for $C_{18}H_{32}OSi$ ($[M]^+$): 292.2222; found: 292.2214.

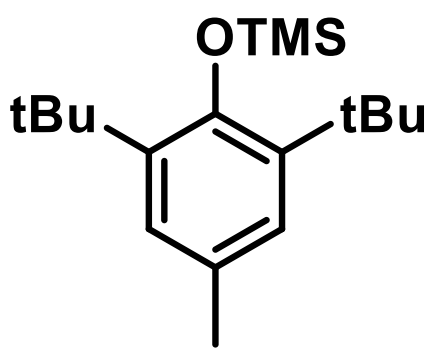

9, HRMS $[M]^+$ m/z $C_{18}H_{32}OSi$
cal.292.2222, obs. 292.2214

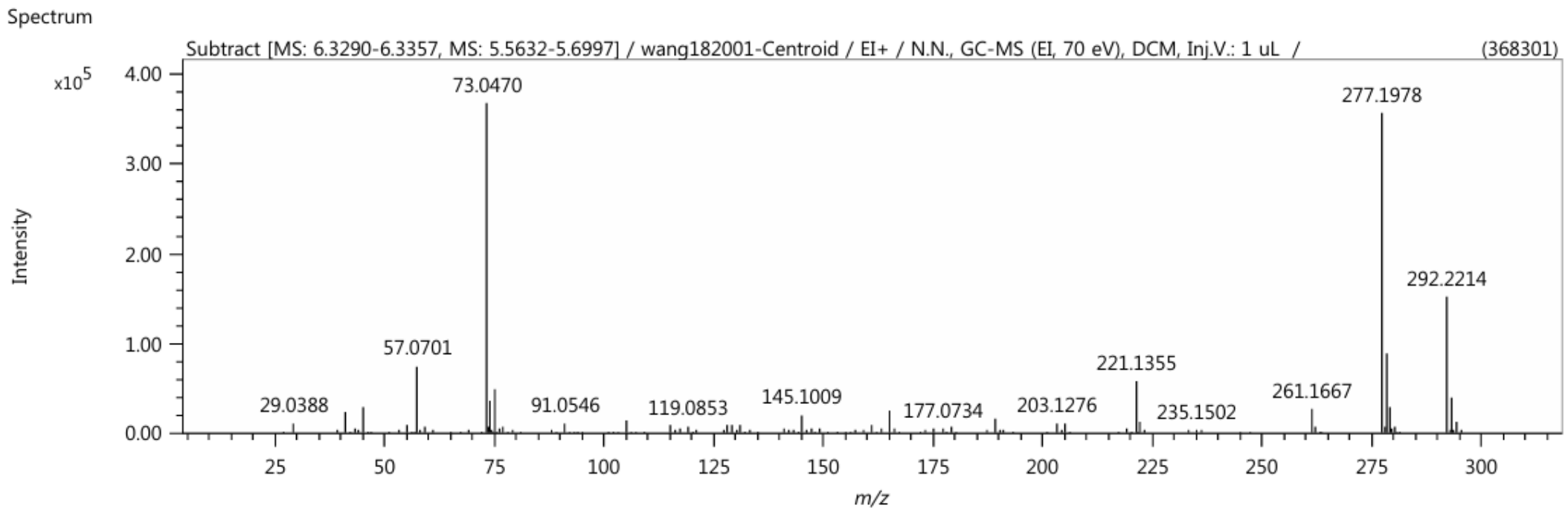

Elemental Composition

Parameters		Elements:									
		Symbol	C	H	Br	I	N	O	P	S	Si
Tolerance:	3.00 ppm	Min	1	1	0	0	0	0	0	0	0
Electron:	Odd/Even	Max	100	100	0	0	3	3	0	0	4
Charge:	+1										
DBE:	-1.5 - 50.0										

Results

Mass	Intensity	Formula	Calculated Mass	Mass Difference [mDa]	Mass Difference [ppm]	DBE
292.22136	152927.33	C18 H32 O Si	292.22169	-0.34	-1.15	4.0

Figure S5. HRMS (EI) reports of the adducts obtained.

A product arising from recombination of the silicon-centered radical formed after the aza-Brook rearrangement with the solvent DMA was also detected by **HRMS(EI)**: *m/z* Calculated for $C_{19}H_{42}N_2OSi_4$ ($[M]^+$): 426.2374; found: 426.2380.

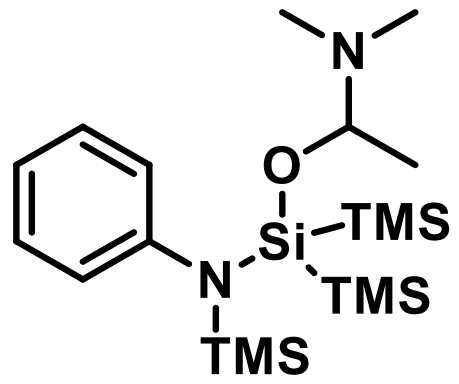

10, HRMS $[M]^+$ m/z $C_{19}H_{42}N_2OSi_4$
cal. 426.2374, obs. 426.2380

Spectrum

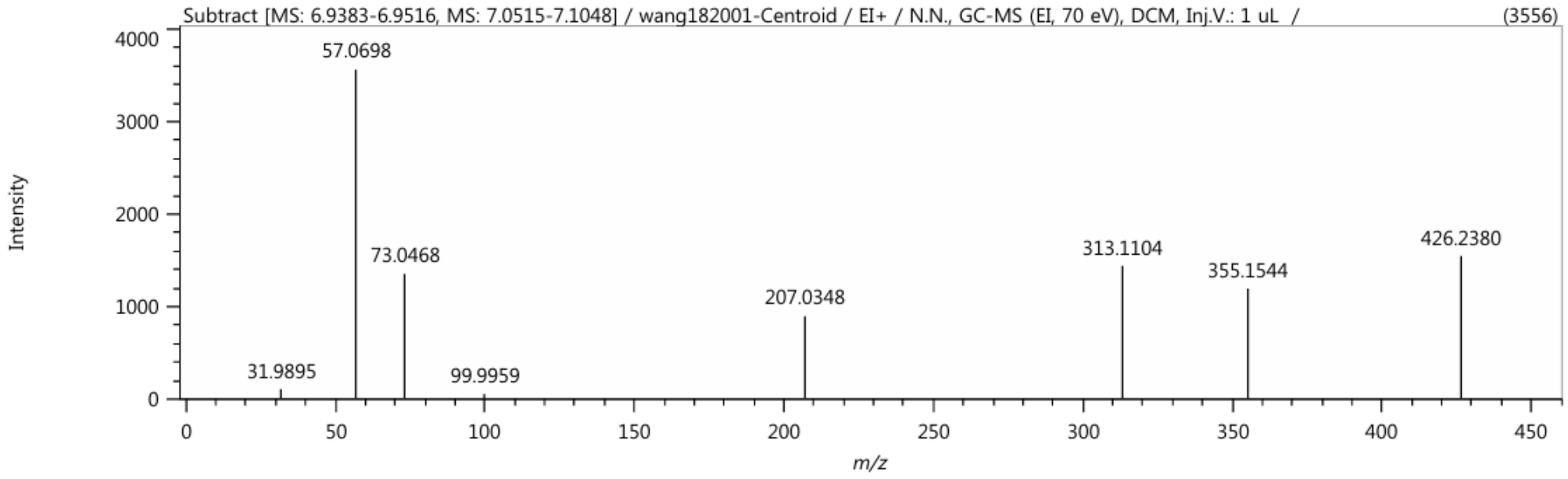

Elemental Composition

Parameters		Elements:									
Tolerance:	3.00 ppm	Symbol	C	H	Br	I	N	O	P	S	Si
Electron:	Odd/Even	Min	1	1	0	0	0	0	0	0	0
Charge:	+1	Max	100	100	0	0	3	3	0	0	4
DBE:	-1.5 - 50.0										

Results

Mass	Intensity	Formula	Calculated Mass	Mass Difference [mDa]	Mass Difference [ppm]	DBE
426.23797	1542.80	C29 H34 O Si	426.23734	0.63	1.47	14.0
		C19 H42 N2 O Si4	426.23687	1.10	2.58	4.0
		C23 H36 N3 O Si2	426.23914	-1.17	-2.75	9.5

Figure S6. HRMS (EI) reports of the adducts obtained.

4.3.3 Using 1,1-diphenyl ethylene

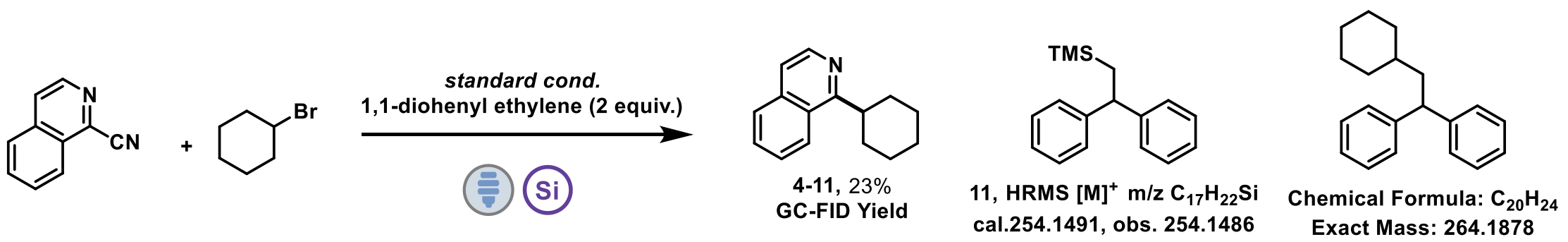

A mixture of 1-isoquinolinecarbonitrile (0.2 mmol), $(TMS_3)_3SiNHPh$ (0.32 mmol) (bromomethyl)cyclopropane (0.32 mmol), NEt_3 (0.2 mmol) and 1,1-diphenyl ethylene (0.4 mmol) was added to an oven-dried 5 mL snap vial equipped with a magnetic stirring bar. The capped vial was evacuated and backfilled with N_2 three times and 2 mL DMA was added to the vial by syringe. Then, the cap was sealed with parafilm. The reaction mixture was stirred vigorously and irradiated with a 365 nm (3.0 W) LED at 25 °C for 3 h. Then diluted with Na_2CO_3 solution and extracted with Diethyl Ether (3 x 15 mL). After the addition of n-dodecane (0.2 mmol) as an internal standard for calibration, the product yield was determined by GC-FID.

1,1-diphenyl ethylene capturing product **11** was detected by **HRMS (EI):** *m/z* Calculated for $C_{17}H_{22}Si$ ($[M]^+$): 254.1491; found: 254.1486.

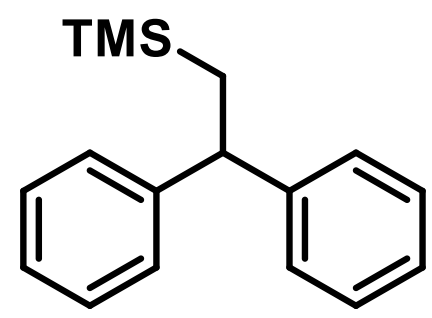

11, HRMS $[M]^+$ m/z $C_{17}H_{22}Si$
cal.254.1491, obs. 254.1486

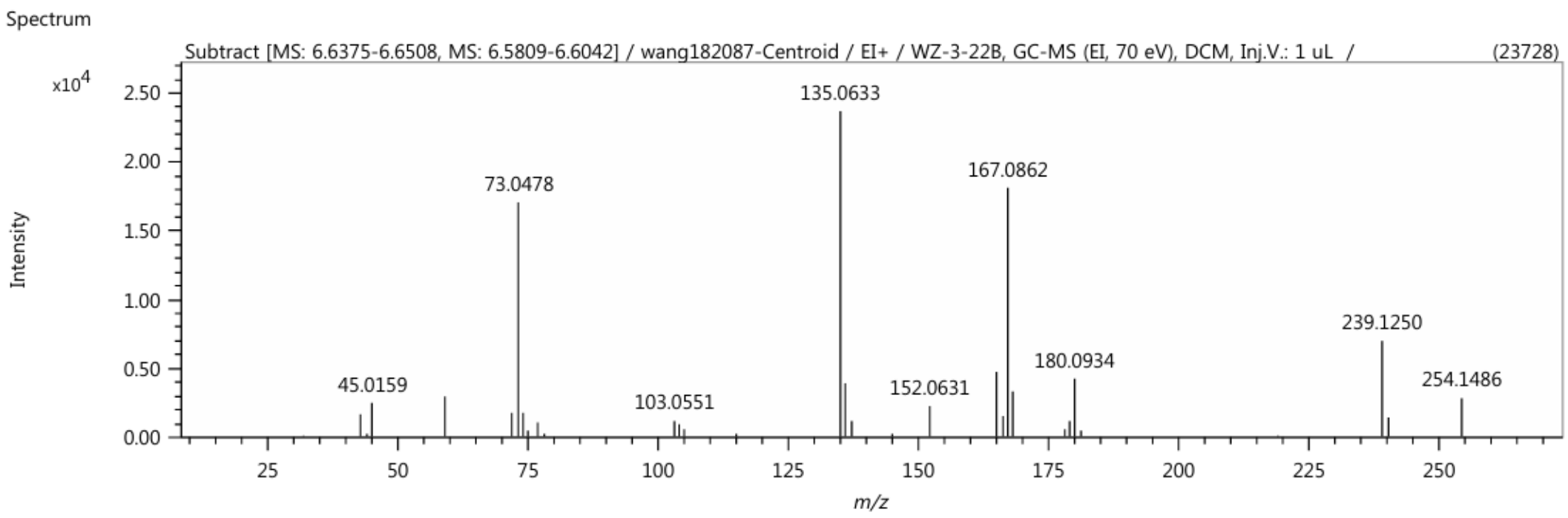

Elemental Composition

Parameters		Elements:									
Tolerance:	5.00 ppm	Symbol	C	H	Br	I	N	O	P	S	Si
Electron:	Odd/Even	Min	1	1	0	0	0	0	0	0	0
Charge:	+1	Max	100	100	0	0	3	0	0	0	4
DBE:	-1.5 - 50.0										

Results

Mass	Intensity	Formula	Calculated Mass	Mass Difference [mDa]	Mass Difference [ppm]	DBE
254.14860	2900.40	C17 H22 Si	254.14853	0.07	0.28	8.0

Figure S7. HRMS (EI) reports of the adducts obtained.

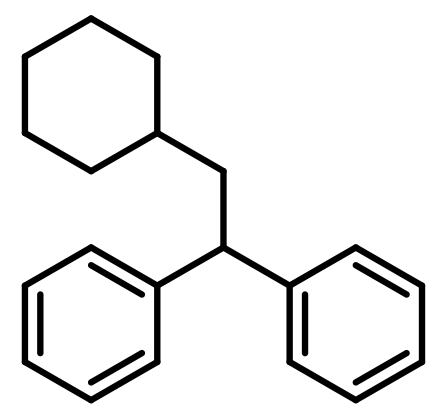

12, HRMS [M]$^+$ m/z $C_{20}H_{24}$
cal.264.1878, obs. 264.1869

1,1-diphenyl ethylene capturing product **12** was detected by **HRMS (EI):** *m/z* Calculated for $C_{20}H_{24}$ ([M]$^+$): 264.1878; found: 264.1869

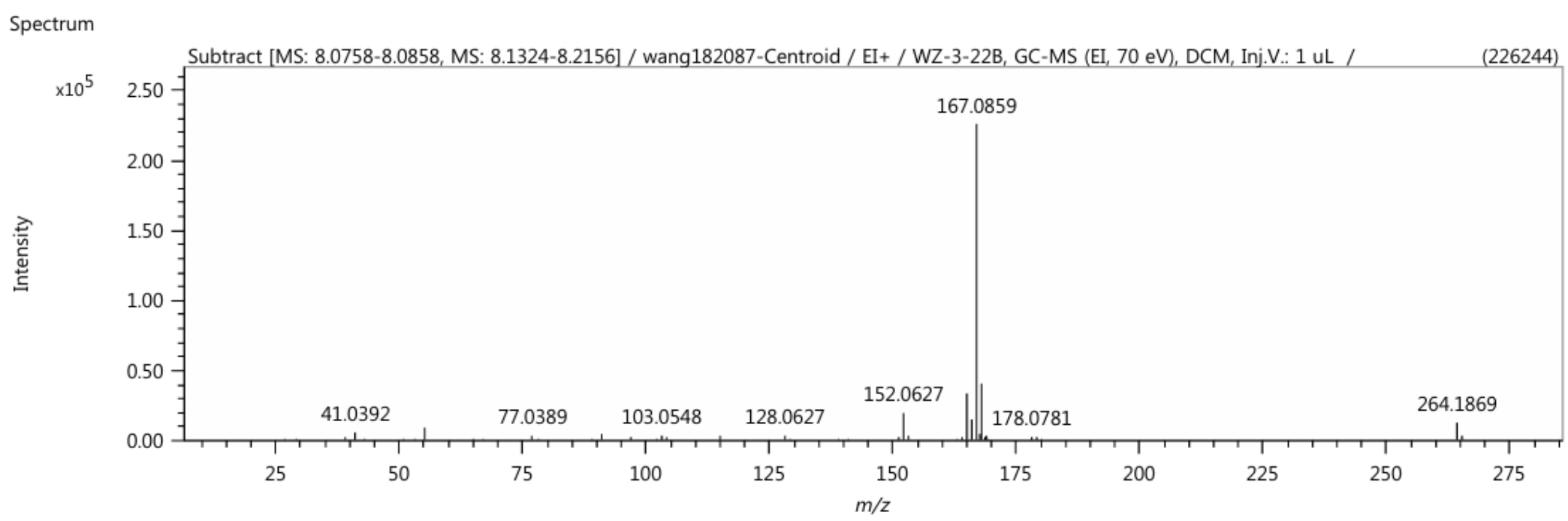

Elemental Composition

Parameters	
Tolerance:	5.00 ppm
Electron:	Odd/Even
Charge:	+1
DBE:	-1.5 - 50.0

Elements:

Symbol	C	H	Br	I	N	O	P	S	Si
Min	1	1	0	0	0	0	0	0	0
Max	100	100	0	0	3	0	0	0	4

Results

Mass	Intensity	Formula	Calculated Mass	Mass Difference [mDa]	Mass Difference [ppm]	DBE
264.18690	12773.75	C10 H32 N2 Si3	264.18678	0.12	0.46	-1.0
		C20 H24	264.18725	-0.35	-1.33	9.0

Figure S8. HRMS (EI) reports of the adducts obtained.

4.4 Fluorescence Quenching Experiments

To visualize the involvement of light for the cross-coupling reaction, the excited state quenching study of 1-isoquinolinecarbonitrile (**1**), fluorescence quenching experiment was conducted. The components under consideration must satisfy the linear correlation obtained from the Stern-Volmer plot for successful quenching:

$$\frac{F_0}{F} = 1 + K_{SV}[\boldsymbol{Q}]$$

Where, F_0 = Fluorescence intensity in absence of quencher, F = Fluorescence intensity in presence of quencher, KSV = Stern-Volmer constant, [Q] = concentration of quencher. At first, the fluorescence emission intensity of a solution containing 0.5 mM 1-isoquinolinecarbonitrile (in DMA) in 1 cm path length air-tight quartz cuvette equipped with a Teflon® septum was recorded by exciting at 365 nm and data was collected from 380 nm to 500 nm. Then to the solution of 0.5 mM **1** sequential addition of 10 mM, 20 mM, 30 mM, 40 mM, 50 mM NEt_3, $(TMS_3)_3SiNHPh$ **(3)** and mixture of NEt_3 and **3** (1:1 in DMA) results in steady decrease in the fluorescence intensity (fluorescence quenching) after each addition.

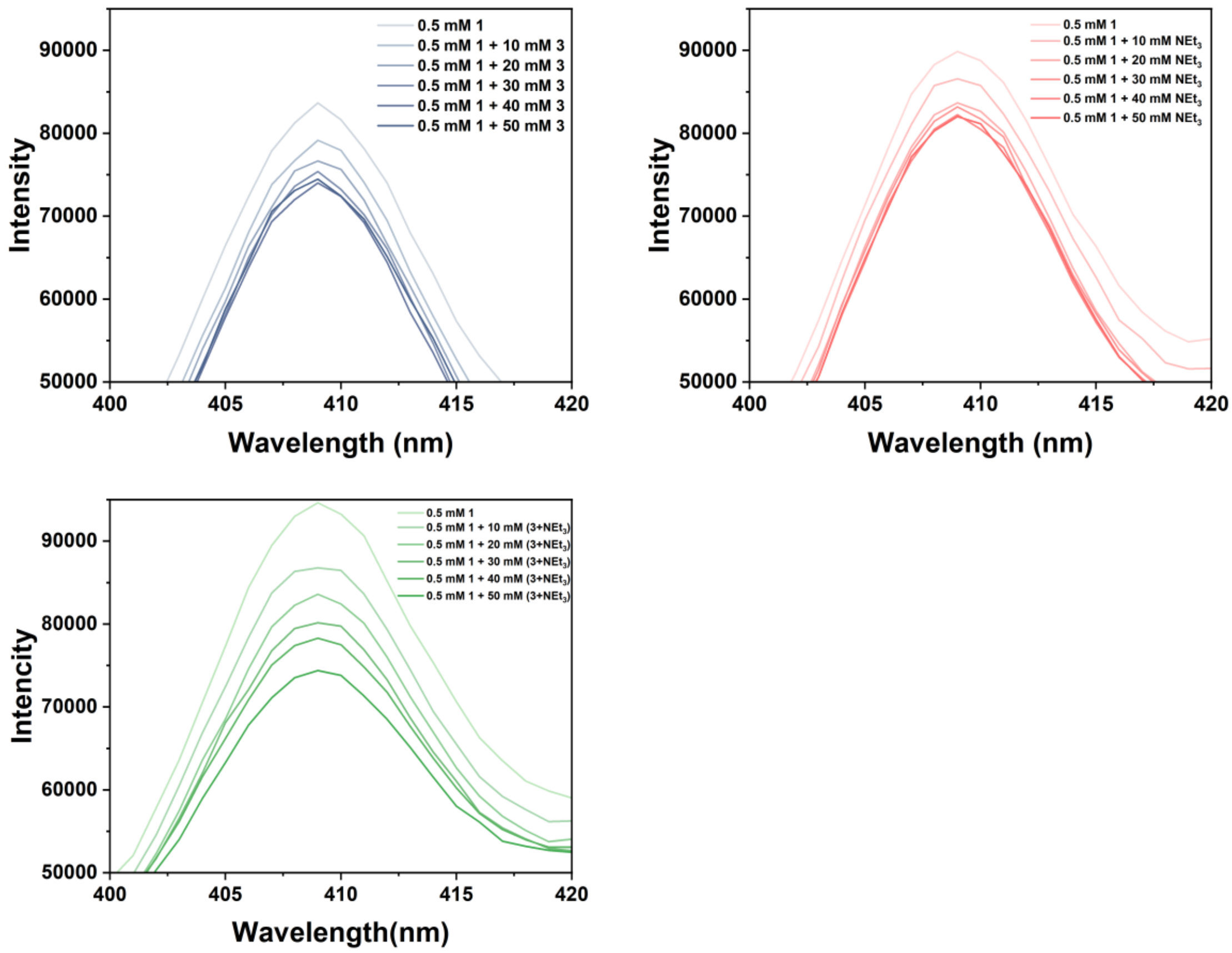

Figure S9. Fluorescence quenching study

Stock solutions of **1** (1-isoquinolinecarbonitrile, 0.1 mmol in 20 mL, 5 mM), **3** ($(TMS)_3SiNHPh$, 0.5 mmol in 5 mL, 100 mM), and NEt_3 (0.5 mmol in 5 mL, 100 mM) were prepared in DMA. In addition, a 1:1 mixed stock solution of **3** and NEt_3 was prepared by dissolving 0.25 mmol of each component in DMA and diluting to a total volume of 5 mL, resulting in final concentrations of 50 mM for each component.

For fluorescence measurements, an aliquot of **1** (300 μL, 1.5 μmol) was diluted to a total volume of 3.0 mL in DMA, giving a final concentration of 0.5 mM. Quenching studies were performed by sequential addition of 300, 600, 900, 1200, and 1500 μL aliquots of these quencher solutions (**3**, NEt_3, or a 1:1 mixture of both) to the cuvette, and the fluorescence spectra were recorded after each addition.

From the data sets obtained, a plot of F_0/F vs [Quencher] was drawn (**Fig. S10**).

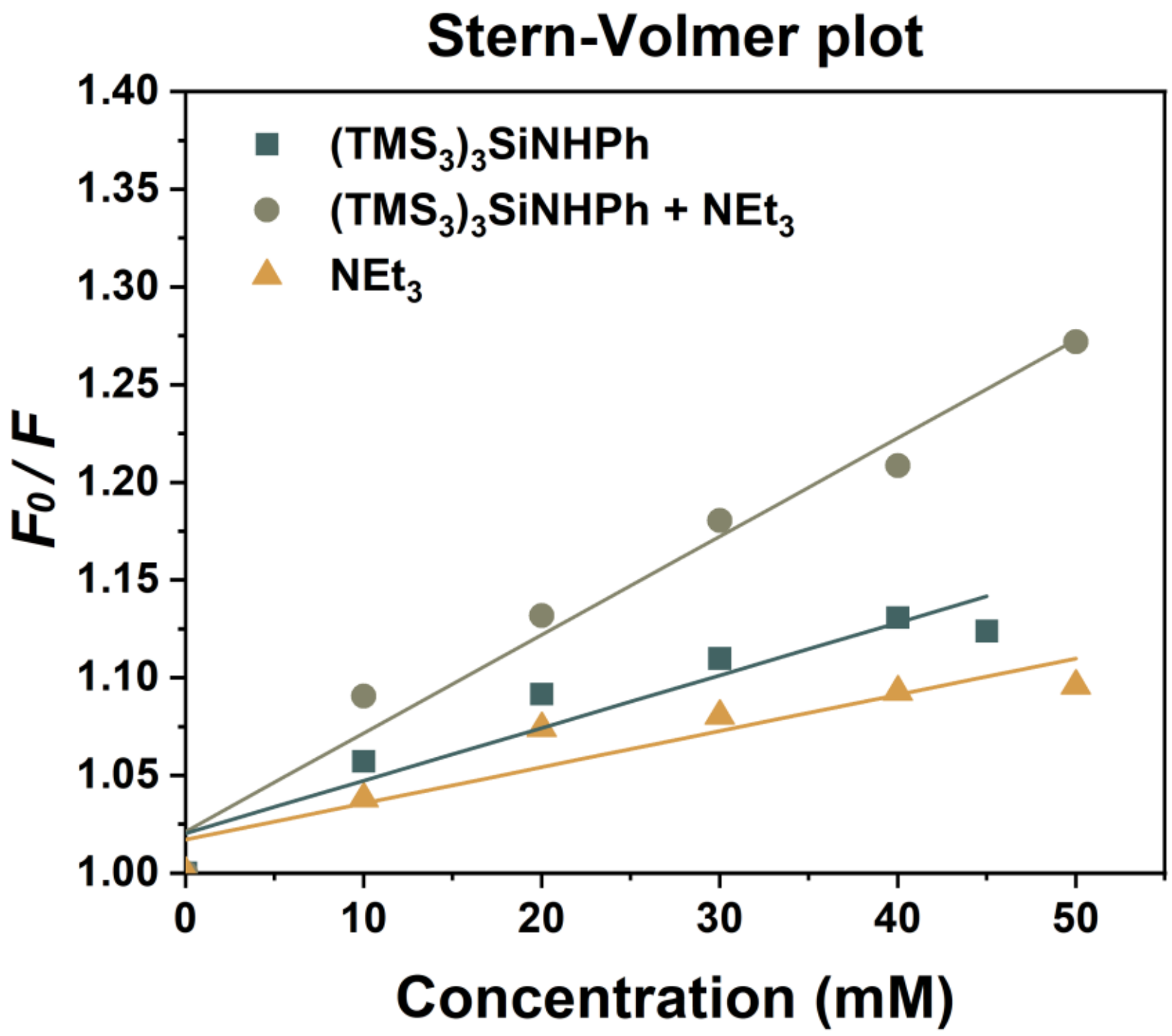

Figure S10. Stern-Volmer Plot

4.5 Excited State Lifetime Studies

To visualize the dynamic quenching of 1-isoquinolinecarbonitrile **(1)**, the excited state lifetime quenching study of **1** through time correlated single photon count (TCSPC) experiment was conducted. The components under consideration must satisfy the linear correlation obtained from Stern Volmer[7] plot for successful quenching:

$$\frac{\tau_0}{\tau} = 1 + K_{SV}[\boldsymbol{Q}]$$

Where, τ_0 = Excited state lifetime in absence of quencher, τ = Excited state lifetime in presence of quencher, K_{SV} = Stern-Volmer constant, [Q] = concentration of quencher.

The fluorescence emission intensity of a solution containing 0.5 mM 1-isoquinolinecarbonitrile (in DMA) in 1 cm path length air-tight quartz cuvette equipped with a Teflon® septum was recorded by exciting at 365 nm and data was collected using ND (neutral density) filter 1.5 (decreasing laser beam intensity by $10^{1.5}$ times) and light-pass filter 370 nm (collecting emission maxima above 370 nm). Then to the solution of 0.5 mM **1** sequential addition of 0.25 mM, 0.33 mM, 0.45 mM $(TMS)_3SiNHPh$ **(3)**, and NEt_3 (in DMA) results in steady decrease in the excited state lifetime (lifetime quenching) after each addition.

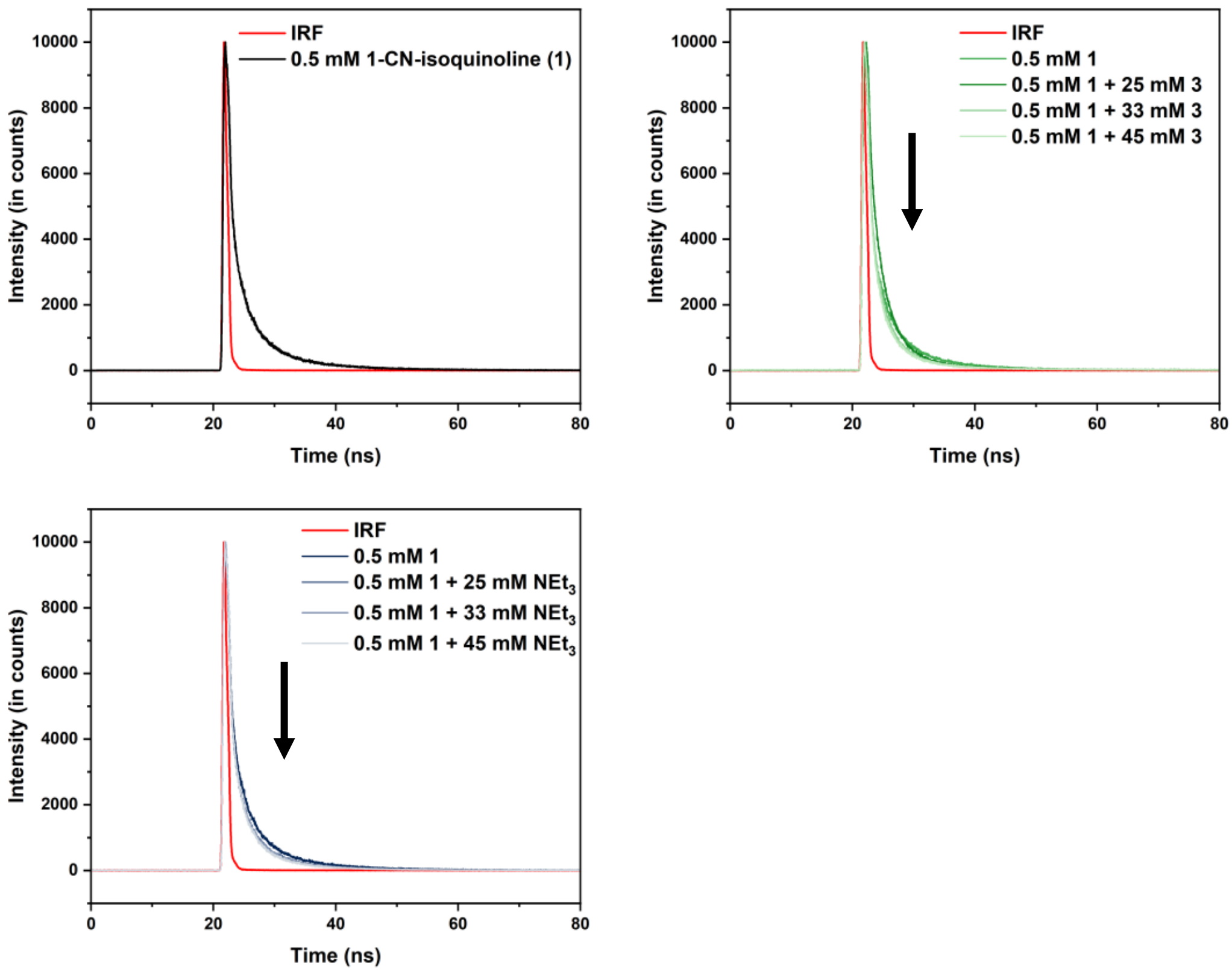

Figure S11. Excited State Lifetime Studies

Stock solutions of **1** (1-isoquinolinecarbonitrile, 0.1 mmol in 20 mL, 5 mM), **3** ($(TMS)_3SiNHPh$, 0.25 mmol in 5 mL, 50 mM), and NEt_3 (0.25 mmol in 5 mL, 50 mM) were prepared in DMA.

For fluorescence measurements, an aliquot of **1** (300 µL, 1.5 µmol) was diluted to a total volume of 3.0 mL in DMA, giving a final concentration of 0.5 mM. Quenching studies were performed by sequential addition 1500, 2000 and 2700 µL aliquots of these quencher solutions (**3** and NEt_3) to the cuvette, all keeping a total volume of 3.0 mL in DMA. And the fluorescence spectra were

recorded after each addition.

From the data sets obtained, a plot of τ_0/τ vs [Quencher] was drawn.

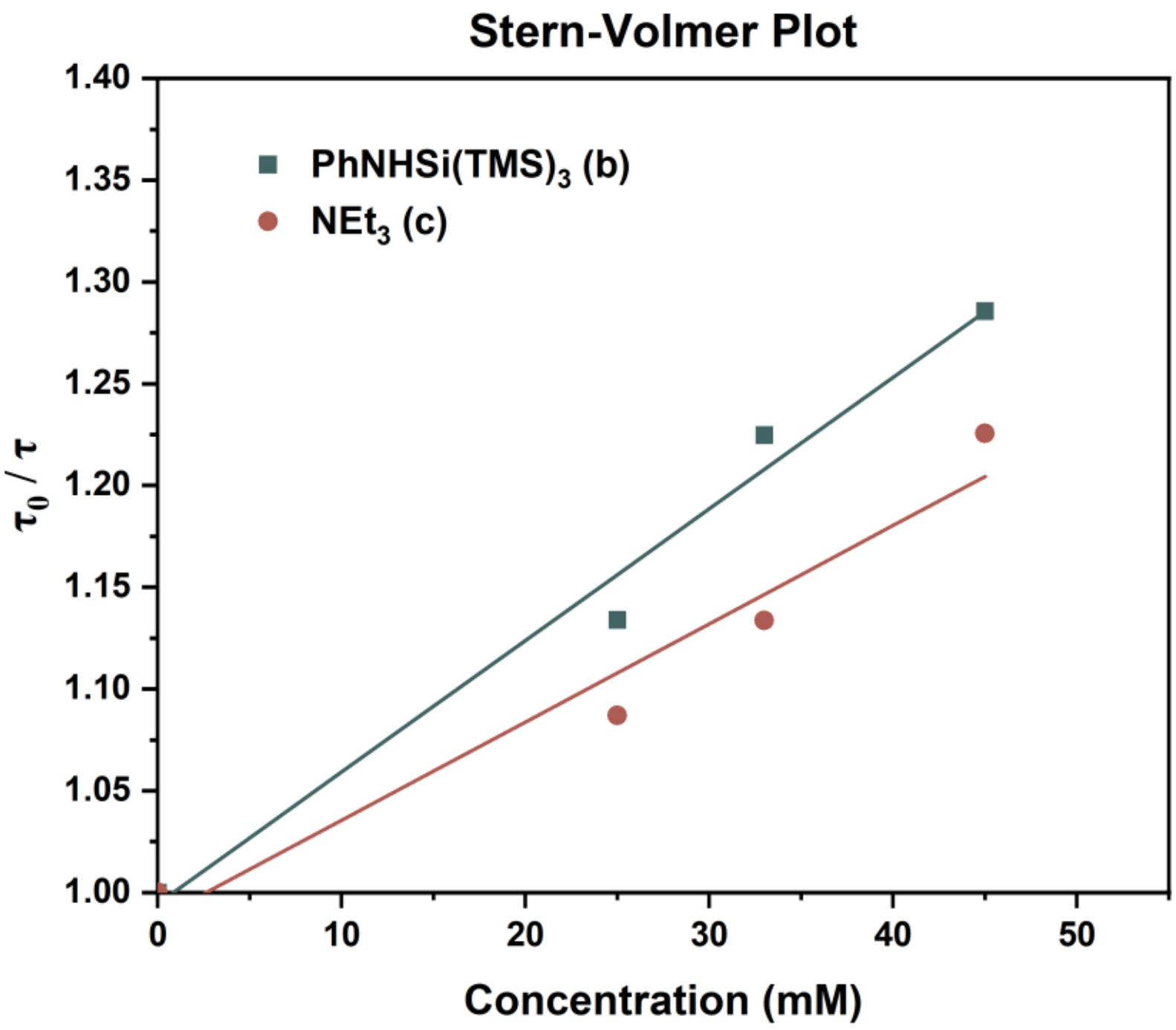

Figure S12. Stern-Volmer Plot for dynamic quenching

Note: The Stern-Volmer plot for dynamic quenching was constructed using the lifetime of the major emissive component (τ_1)

4.6 Cyclic Voltammetry Studies

Cyclic voltammetry (CV) measurements were performed using an Autolab PGSTAT302N potentiostat (Metrohm) in a conventional three-electrode configuration, consisting of a glassy carbon working electrode, a platinum wire counter electrode, and a silver wire pseudo-reference electrode. The electrolyte solution contained 0.1 M tetrabutylammonium tetrafluoroborate ($TBABF_4$, Fluka) in N, N-dimethylacetamide (DMA). Prior to each measurement, the solution was thoroughly degassed with argon for at least 15 minutes, and all experiments were carried out under an argon atmosphere. Ferrocene (Fc) was used as an internal standard, and all potentials were initially referenced to the Fc/Fc^+ redox couple. The potentials were subsequently converted to the saturated calomel electrode (SCE) scale using the following relation:

E (vs. SCE) = **E** (vs. Fc/Fc^+) + 0.38 V.

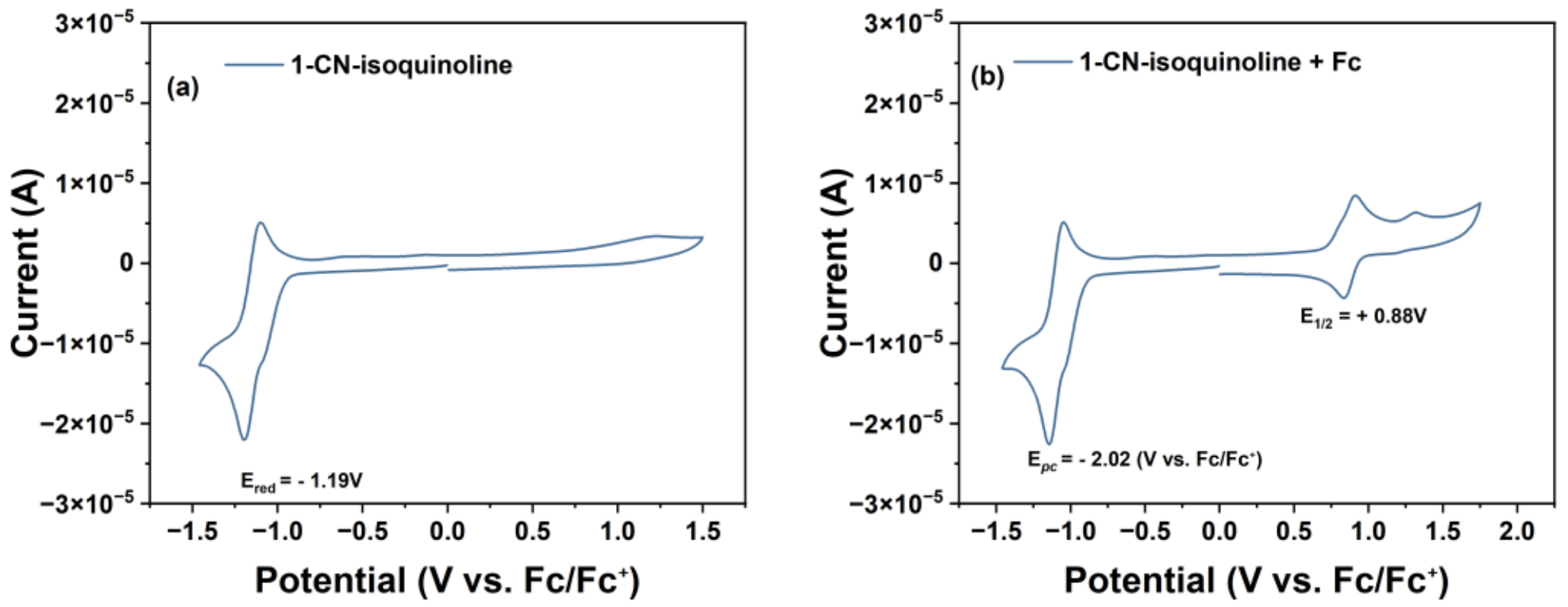

Figure S13. (a) CV of 1-CN-isoquinoline (b) CV of 1-CN-isoquinoline with ferrocene

The cyclic voltammogram of 1-CN-isoquinoline (using 0.1 M $TBABF_4$ as a supporting electrolyte in DMA) was recorded (initial potential = 0 V, Upper vertex potential = +2.0 V (with Fc), Lower vertex potential = - 1.5 V, stopping potential = 0 V) with 50 mV/s scan rate.

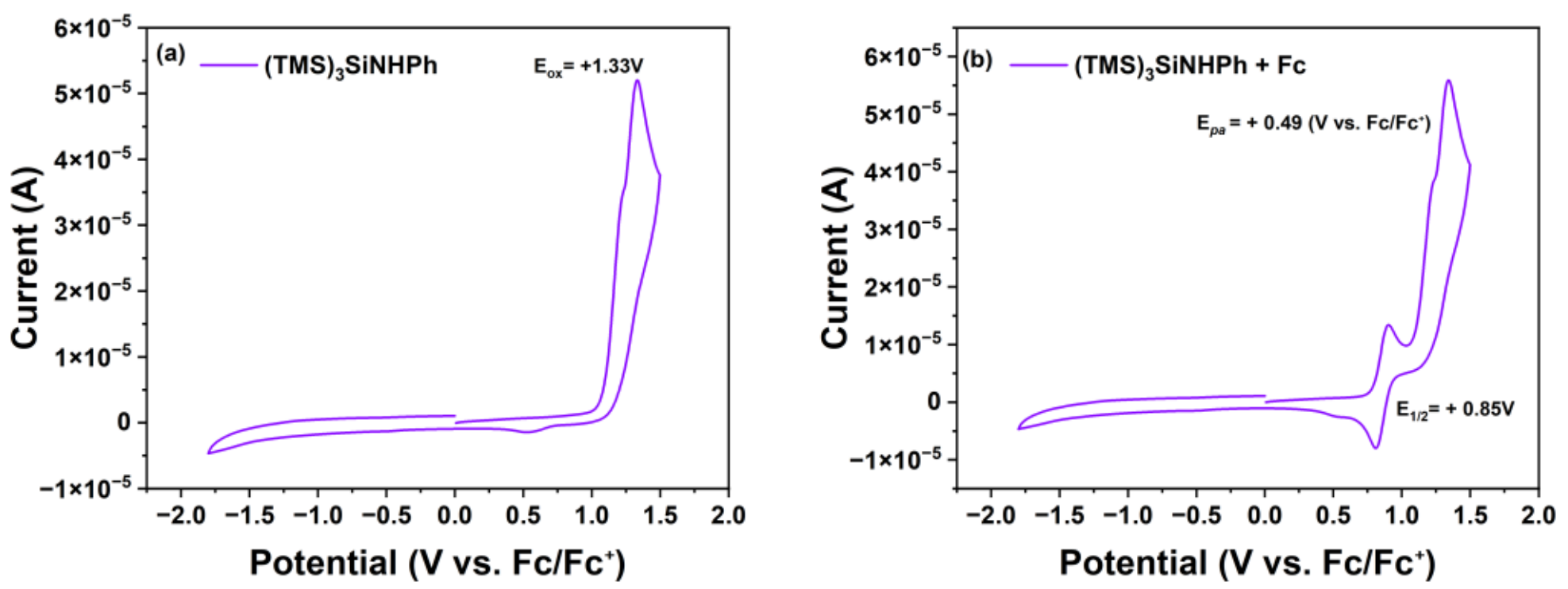

Figure S14. (a) CV of $(TMS_3)_3SiNHPh$ (b) CV of $(TMS_3)_3SiNHPh$ with ferrocene

The cyclic voltammogram of $(TMS_3)_3SiNHPh$ (using 0.1 M $TBABF_4$ as a supporting electrolyte in DMA) was recorded (initial potential = 0 V, Upper vertex potential = +1.5 V (with Fc), Lower vertex potential = - 2.0 V, stopping potential = 0 V) with 50 mV/s scan rate.

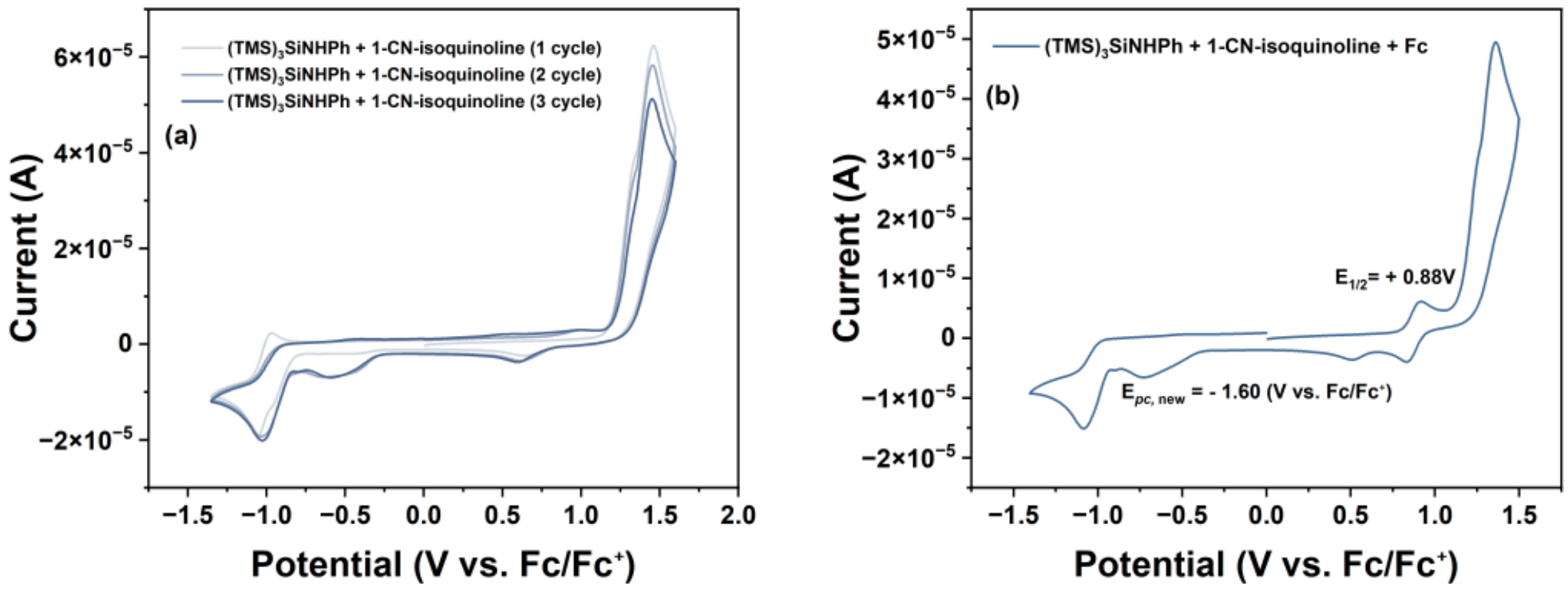

Figure S15. (a) CV of mixture of 1-CN-isoquinoline and $(TMS_3)_3SiNHPh$ (b) CV of mixture of 1-CN-isoquinoline and $(TMS_3)_3SiNHPh$ with ferrocene

The cyclic voltammogram of mixture of 1-CN-isoquinoline and $(TMS_3)_3SiNHPh$ (using 0.1 M $TBABF_4$ as a supporting electrolyte in DMA) was recorded (initial potential = 0 V, Upper vertex potential = +1.5 V (with Fc), Lower vertex potential = - 1.5 V, stopping potential = 0 V) with 50 mV/s scan rate.

$\mathbf{E}_{pc,\ \mathrm{new}}$ = - 1.60 (V vs. Fc/Fc$^+$)

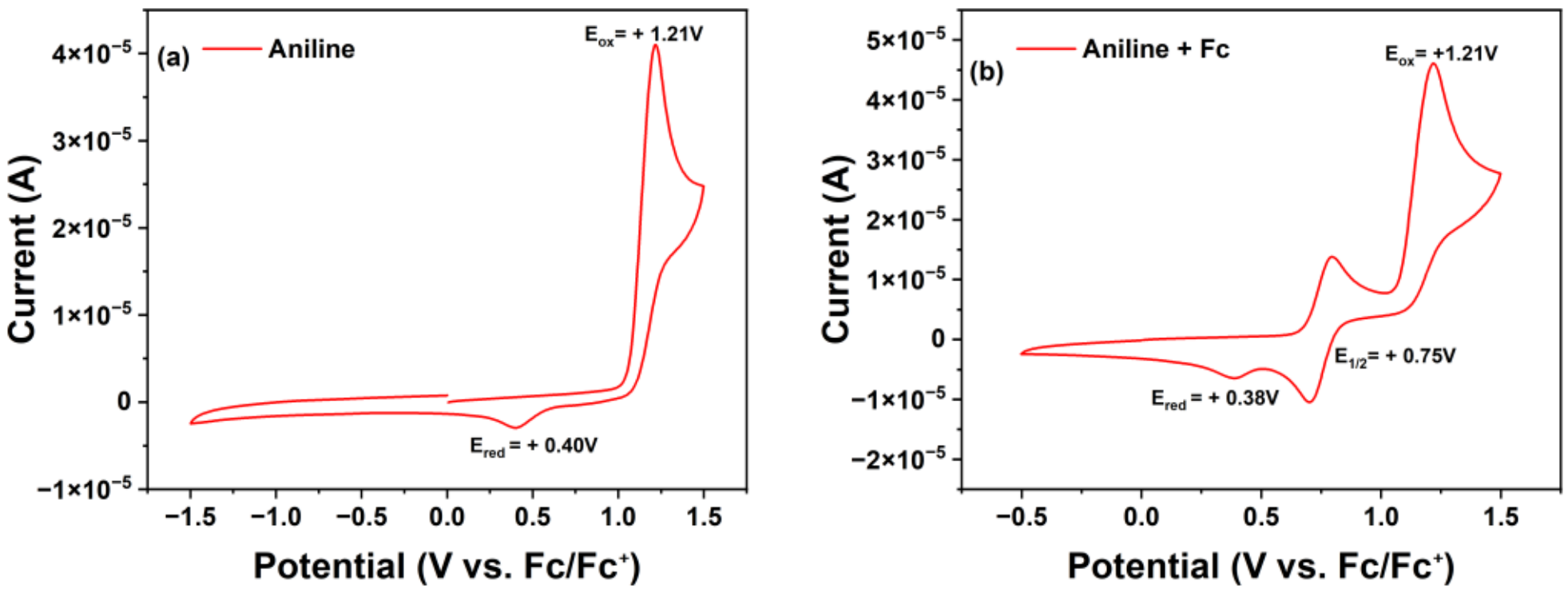

Figure S16. (a) CV of Aniline (b) CV of Aniline with ferrocene

The cyclic voltammogram of Aniline (using 0.1 M $TBABF_4$ as a supporting electrolyte in DMA) was recorded (initial potential = 0 V, Upper vertex potential = +1.5 V (with Fc), Lower vertex potential = - 1.5 V, stopping potential = 0 V) with 50 mV/s scan rate.

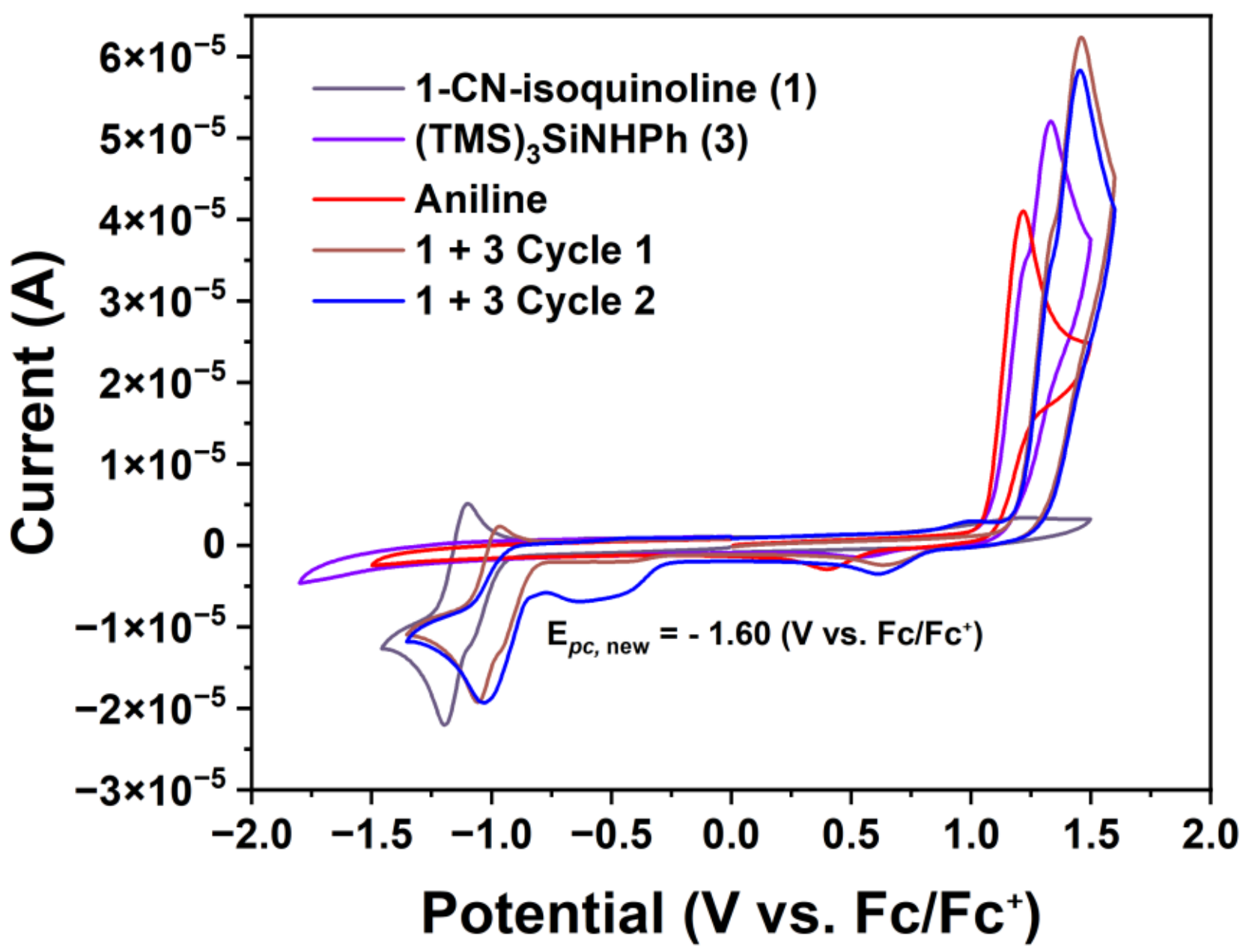

Figure S17. Stacked CV studies

4.7 Light On-Off Experiment

Following the general procedure, a light on-off experiment was designed. A 1 mmol reaction was conducted, and 1 mL of the sample was taken at alternating hours of light-on and light-off conditions. The crude yields were recorded by GC-FID analyses with 0.1 mmol n-dodecane as an inert standard. The nature of the graph indicates that the cross-coupling reaction is light dependent, as negligible formation of the desired product was observed in the absence of light.

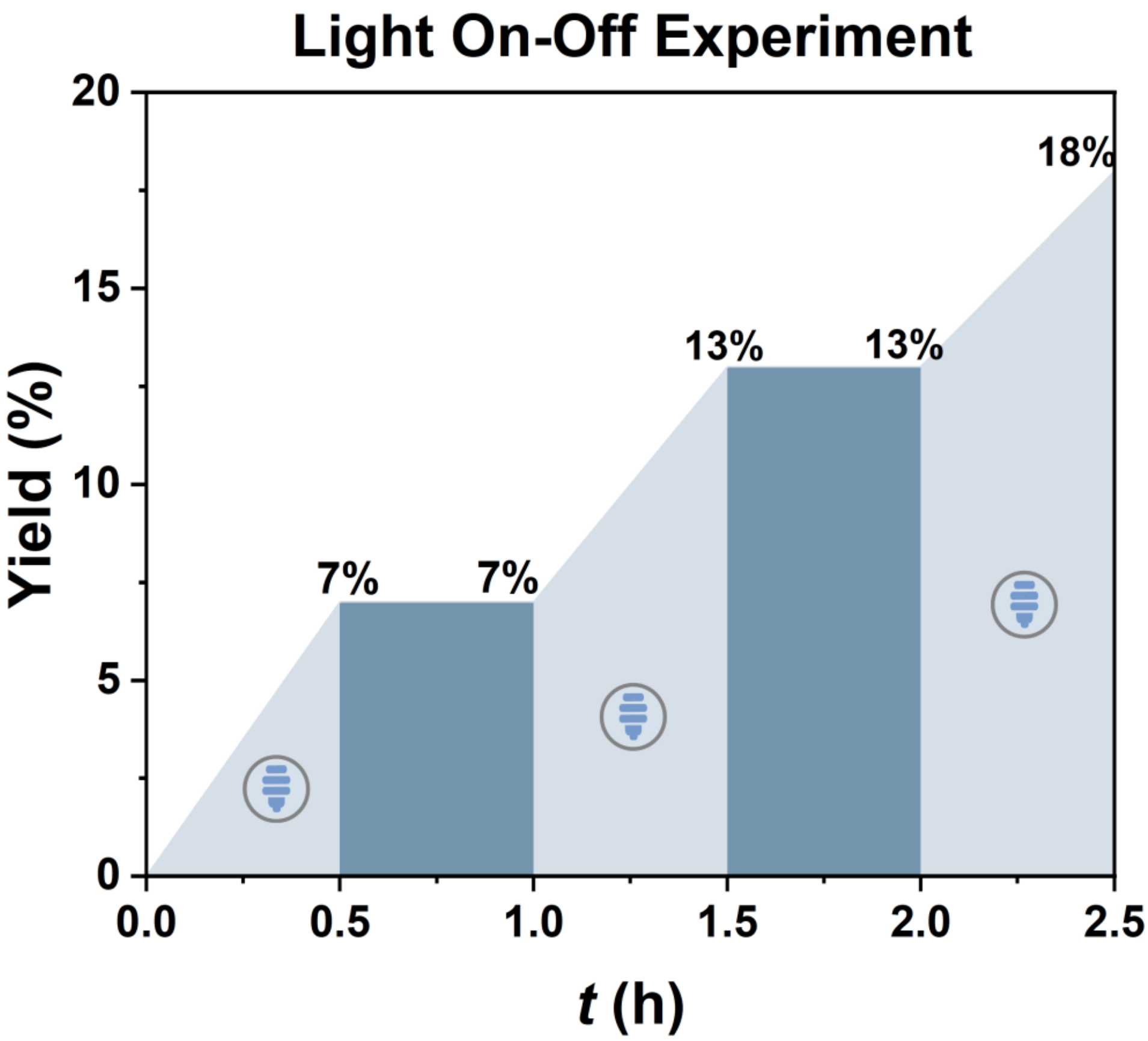

Figure S18. Light On-Off Experiment

4.8 π-π Interaction Studies

UV–vis absorption experiments were conducted to probe the π-π interactions between $(TMS_3)_3SiNHPh$ and different heteroaryl nitriles with varying degrees of π-conjugation. A bathochromic shift was observed upon addition of the aryl nitriles, and the position of the absorption band is largely consistent with the intrinsic absorption features of the individual heteroaryl nitriles, which shift to longer wavelengths with increasing π-conjugation. These results suggest that the spectral features of the EDA complex are primarily governed by the electronic structure of the substrates, while still reflecting intermolecular interactions with $(TMS_3)_3SiNHPh$.

Preparation of the sample for compound **a, b, c** and **d**: add compound **a** (**b, c or d**) (0.1 mmol) to a 5 mL vial, followed by adding 3 mL of DMA into the vial. Afterward, filter the sample through a plug of celite (Ø 3 mm × 8 mm) into the cuvette for the UV-vis measurements.

Preparation of the binary (**a**+**b**, **b**+**c**, **b+d**, **b**+**e**) the respective compounds (0.1 mmol) were weighed into a 5 mL vial, followed by adding 3 mL of DMA into the vial. Afterward, filter the sample through a plug of celite (Ø 3 mm × 8 mm) into the cuvette for the UV-vis measurements.

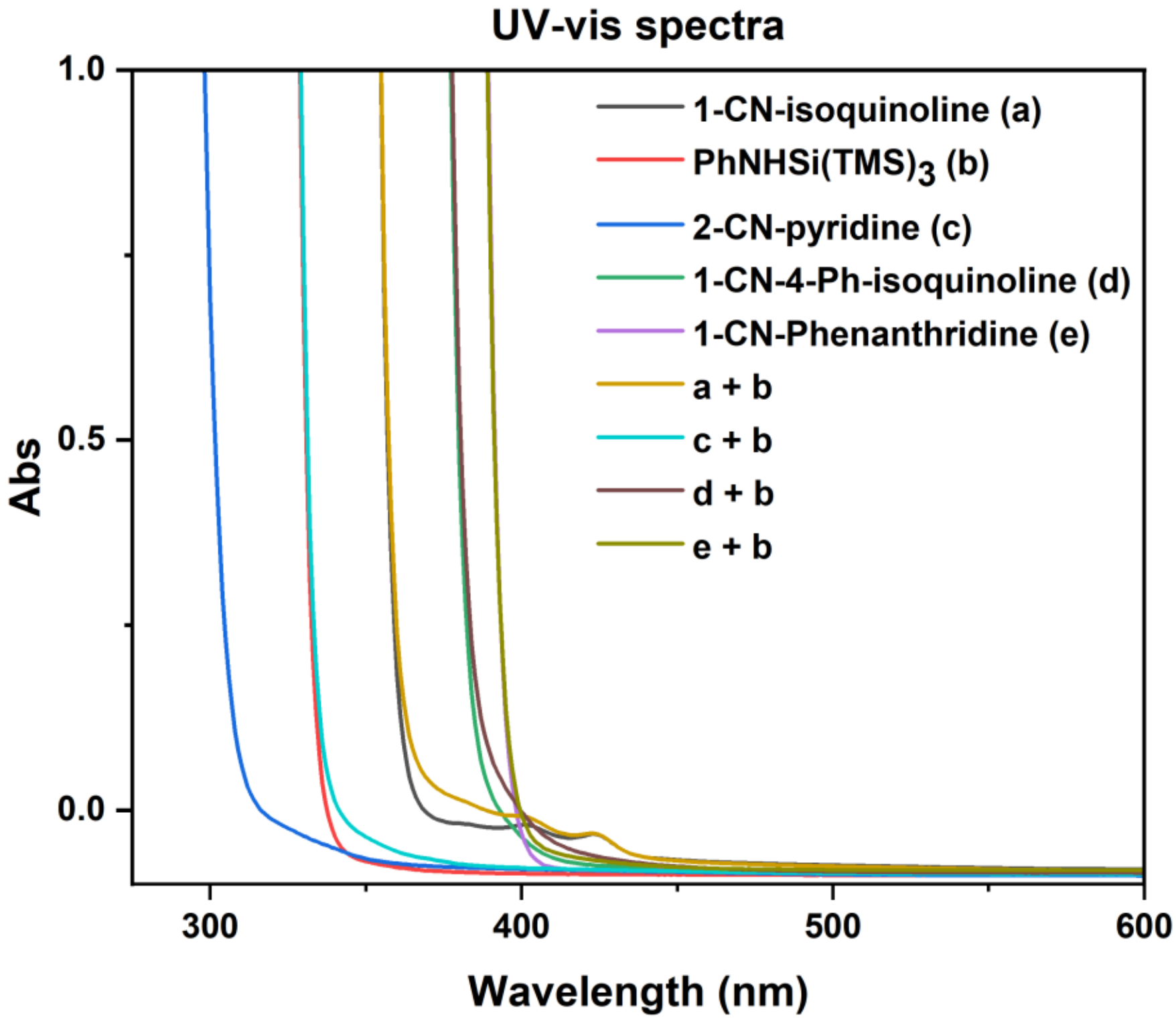

Figure S19. π-π Interaction Studies

Note: We have observed that $(TMS)_3SiNHPh$ decompose slowly in DMA, measurements should be performed immediately after solution preparation.

4.9 Quantum yield measurements

The quantum yield (Φ) was determined using a custom-built setup[8], which includes a magnetic stirrer with a Faulhaber motor (1524B024S R) and a 14:1 gear (15A). The setup also features an adjustable power supply "Basetech BT-153 0–15 V/DC 0–3 A 45 W," PowerMax software, a PS19Q power sensor from Coherent, and translation stages (horizontal and vertical) such as Thorlabs DT 25/M or DT S25/M. Additionally, a photographic lens with f = 50 mm was employed in the measurements. The quantum yield (Φ) was calculated as follows:

$$\Phi = \frac{N_{prod}}{N_{ph,abs}} = \frac{c_{prod} \cdot V \cdot N_A \cdot h \cdot c}{P_{abs} \cdot \Delta t \cdot \lambda}$$

Here, N_{prod} is the number of product molecules, $N_{ph,abs}$ is the absorbed photons, c_{prod} is the concentration of the product species, V is the sample volume, N_A is the Avogadro number, h is the Planck constant, c is the speed of light, P_{abs} is the absorbed optical power, Δt is the illumination time and λ is the central wavelength of the LED.

The absorbed optical power P_{abs} can be calculated from the optimal power transmitted by the reference cuvette (P_{ref}) and the sample cuvette (P_{sample}):

$$P_{abs} = (P_{ref} - P_{sample}) \cdot f$$

The constant f is a correction factor on the order of a few % that depends on the reflection coefficient R of the air-glass-interface. Neglecting second order effects, f can be written as:

$$f = \frac{1 + R\frac{P_{sample}}{P_{ref}}}{1 - R}$$

The denominator 1−R corrects for the lower light power measured as compared to the light power at the sample due to partial reflection at the exit surface. The additional term in the numerator accounts for the fact that the black reflected light is again absorbed by the solution.

The experimental procedure for determining the quantum yield of the reaction is as follows: First, the cuvette was degassed with N_2 for 3 min, along with a stirring bar. Then **2 mL DMA** was added into the experimental cuvette. Then the aforementioned cuvette was carefully put into the measurement instrument, stirred, and irradiated with a precisely calibrated 365 nm light source, providing data $P_{ref.}$ The cuvette was then removed from the instrument, and **2 ml 0.05M reaction mixture** was added into the cuvette. The mixture was degassed with N_2 for 3 min and then put back into the measurement instrument. Samples were collected at different time points, and the resulting yields were analyzed using ^{1}H NMR. The quantum yield value calculated based on the average of measurement data is Φ = 1.8%.

5. Synthesis and characterization of products

Procedures for the reaction conditions

A) General procedure of cross-coupling reaction

Unless specified otherwise, a mixture of an electron acceptor substrate, e.g., 1-isoquinolinecarbonitrile (0.2 mmol), electron donor reagent $(TMS)_3SiNHPh$ (0.32 mmol) Alkyl bromide, eg., bromocyclohexane (0.32 mmol), and additive, eg., NEt_3 (0.2 mmol) was added to an oven-dried 5 mL snap vial equipped with a magnetic stirring bar. The capped vial was evacuated and backfilled with N_2 three times, and 2 mL DMA was added to the vial by syringe. Then, the cap was sealed with parafilm. The reaction mixture was stirred vigorously and irradiated with a 365 nm (3.0 W or 500 mW) LED at 25 °C for 3 h (for the usage of 500 mW LED, reaction time is 12h). Then diluted with Na_2CO_3 solution and extracted with diethyl ether (3 x 15 mL). After addition of CH_2Br_2 (0.2 mmol) as an internal standard for calibration, the product yield was determined by ^{1}H NMR. The residue was purified by silica gel flash chromatography to give the desired product **4**.

Caution! *Cyanide-containing byproducts may be generated during the reaction. Upon completion, the reaction mixture should be carefully quenched with an aqueous Na_2CO_3 solution in a well-ventilated fume hood. Appropriate personal protective equipment should be worn, and all waste must be handled in accordance with established safety protocols for cyanide-containing materials.*

B) General procedure of Minisci type reaction

Unless specified otherwise, a mixture of an electron acceptor substrate, e.g., 4-Me-quinoline (0.3 mmol), electron donor reagent $(TMS)_3SiNHPh$ (0.24 mmol) Alkyl bromide, eg., bromocyclohexane (0.2 mmol) and additive, eg., NPh_3 (0.2 mmol) was added to an oven-dried 5 mL snap vial equipped with a magnetic stirring bar. The capped vial was evacuated and backfilled with N_2 three times and 2 mL DMA was added to the vial by syringe. Then, the cap was sealed with parafilm. The reaction mixture was stirred vigorously and irradiated with a 365 nm (3.0 W or 500 mW) LED at 25 °C for 6 h (for the usage of 500 mW LED, reaction time is 16h). Then diluted with Na_2CO_3 solution and extracted with diethyl ether (3 x 15 mL). After addition of CH_2Br_2 (0.2 mmol) as an internal standard for calibration, the product yield was determined by ^{1}H NMR. The residue was purified by silica gel flash chromatography to give the desired product **6**.

5.1 Characterization of products

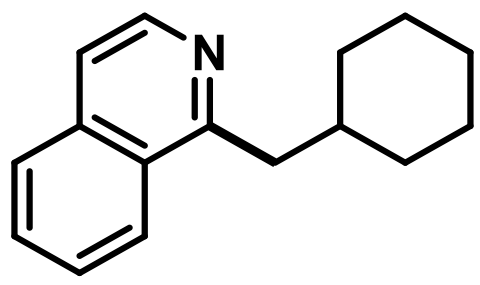

1-(cyclohexylmethyl)isoquinoline (4-1)[9]**:** Following the general procedure A, substrate **4-1** was obtained as a yellow oil (27.5 mg, 61% yield).

^{1}H NMR (400 MHz, CDCl$_3$) δ 8.44 (d, *J* = 5.8 Hz, 1H), 8.16 (d, *J* = 8.6 Hz, 1H), 7.81 (d, *J* = 8.0 Hz, 1H), 7.66 (ddd, *J* = 8.1, 6.8, 1.2 Hz, 1H), 7.58 (ddd, *J* = 8.3, 6.8, 1.4 Hz, 1H), 7.50 (d, *J* = 5.7 Hz, 1H), 3.18 (d, *J* = 7.2 Hz, 2H), 2.01 – 1.84 (m, 1H), 1.76 – 1.56 (m, 5H), 1.40 – 1.05 (m, 5H).

^{13}C NMR (101 MHz, CDCl$_3$) δ 161.6, 141.6, 136.5, 130.0, 127.6, 127.5, 127.1, 125.9, 119.3, 43.0, 39.2, 33.7, 26.6, 26.4.

LRMS (**EI**): *m/z* Calculated for $C_{16}H_{19}N$ ($[M]^+$): 225.2; found: 225.2.

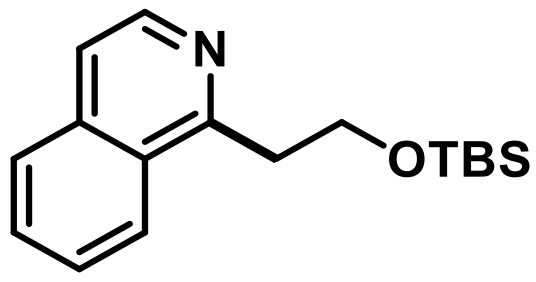

1-(2-((tert-butyldimethylsilyl)oxy)ethyl)isoquinoline (4-2): Following the general procedure A, substrate **4-2** was obtained as a yellow oil (29.2 mg, 52% yield).

^{1}H NMR (400 MHz, CDCl$_3$) δ 8.44 (d, *J* = 5.7 Hz, 1H), 8.26 (dd, *J* = 8.5, 1.1 Hz, 1H), 7.80 (d, *J* = 8.1 Hz, 1H), 7.66 (ddd, *J* = 8.2, 6.8, 1.2 Hz, 1H), 7.58 (ddd, *J* = 8.3, 6.8, 1.4 Hz, 1H), 7.52 (d, *J* = 5.8 Hz, 1H), 4.15 (t, *J* = 6.9 Hz, 2H), 3.54 (t, *J* = 6.9 Hz, 2H), 0.78 (s, 9H), -0.11 (s, 6H).

^{13}C NMR (101 MHz, CDCl$_3$) δ 159.9, 141.6, 136.3, 130.1, 128.0, 127.23, 127.15, 126.3, 119.7, 63.3, 38.4, 26.0, 18.3, -5.4.

HRMS (**EI**): *m/z* Calculated for $C_{17}H_{25}NOSi$ ($[M]^+$): 287.1705; found: 287.1665.

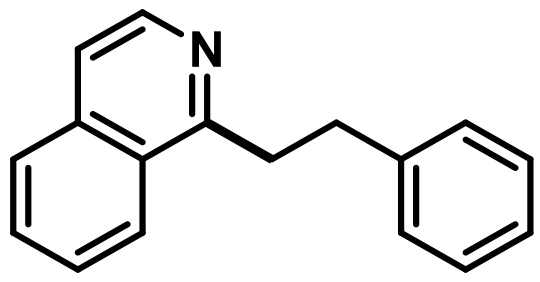

1-phenethylisoquinoline (4-3)[10]**:** Following the general procedure A, substrate **4-3** was obtained as a yellow oil (29.2 mg, 62% yield).

^{1}H NMR (400 MHz, CDCl$_3$) δ 8.48 (d, *J* = 5.7 Hz, 1H), 8.16 (d, *J* = 8.3 Hz, 1H), 7.83 (d, *J* = 8.2 Hz, 1H), 7.67 (ddd, *J* = 8.1, 6.8, 1.2 Hz, 1H), 7.59 (ddd, *J* = 8.3, 6.8, 1.3 Hz, 1H), 7.54 (d, *J* = 5.7 Hz, 1H), 7.32 (d, *J* = 4.7 Hz, 4H), 7.25 – 7.17 (m, 1H), 3.69 – 3.51 (m, 2H), 3.37 – 3.08 (m, 2H).

^{13}C NMR (101 MHz, CDCl$_3$) δ 161.2, 142.1, 142.0, 136.4, 130.0, 128.6, 128.61, 127.58, 127.2, 127.1, 126.2, 125.2, 119.6, 37.4, 35.6.

LRMS (**EI**): *m/z* Calculated for $C_{17}H_{15}N$ ($[M]^+$): 233.1; found: 233.1.

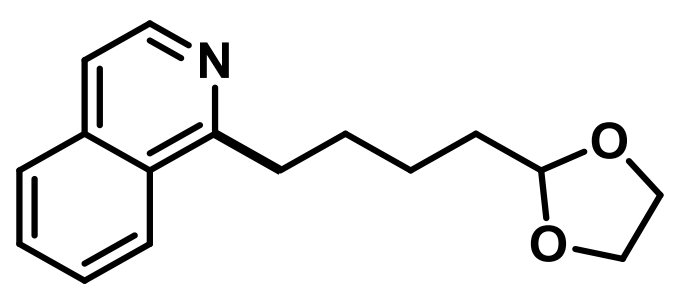

1-(4-(1,3-dioxolan-2-yl)butyl)isoquinoline (4-4): Following the general procedure A, using 365 nm (500 mW) LED and reaction time is 12h, substrate **4-4** was obtained as a yellow oil (19.4 mg, 38% yield).

^{1}H NMR (400 MHz, CDCl$_3$) δ 8.42 (d, J = 5.7 Hz, 1H), 8.15 (d, J = 8.2 Hz, 1H), 7.81 (d, J = 8.1 Hz, 1H), 7.66 (ddd, J = 8.2, 6.9, 1.2 Hz, 1H), 7.58 (ddd, J = 8.3, 6.9, 1.4 Hz, 1H), 7.50 (d, J = 5.4 Hz, 1H), 4.87 (t, J = 4.7 Hz, 1H), 4.02 – 3.93 (m, 2H), 3.93 – 3.78 (m, 2H), 3.35 – 3.27 (m, 2H), 1.99 – 1.86 (m, 2H), 1.79 – 1.72 (m, 2H), 1.69 – 1.48 (m, 2H).

^{13}C NMR (101 MHz, CDCl$_3$) δ 162.2, 142.0, 136.4, 129.9, 127.5, 127.13, 127.06, 125.5, 119.4, 104.7, 65.0, 35.5, 33.9, 29.7, 24.5.

HRMS (**EI**): *m/z* Calculated for $C_{16}H_{19}NO_2$ ([M]$^+$): 257.1416; found: 257.1410.

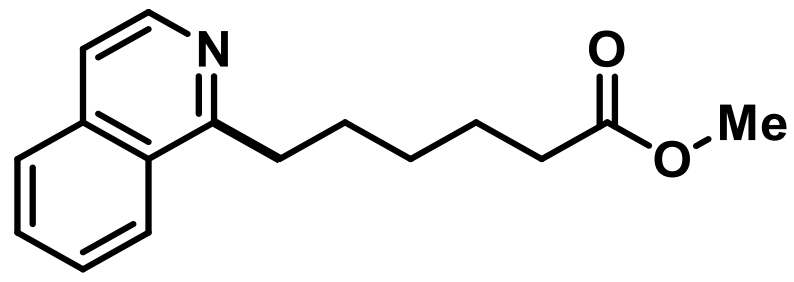

methyl 6-(isoquinolin-1-yl)hexanoate (4-5): Following the general procedure A, substrate **4-5** was obtained as a brown oil (31.5 mg, 62% yield).

^{1}H NMR (400 MHz, CDCl$_3$) δ 8.41 (d, J = 5.8 Hz, 1H), 8.16 (d, J = 8.7 Hz, 1H), 7.83 (d, J = 8.1 Hz, 1H), 7.69 (ddd, J = 8.2, 6.8, 1.3 Hz, 1H), 7.61 (ddd, J = 8.3, 6.8, 1.3 Hz, 1H), 7.54 (d, J = 5.8 Hz, 1H), 3.64 (s, 3H), 3.38 – 3.29 (m, 2H), 2.32 (t, J = 7.5 Hz, 2H), 1.89 (qd, J = 9.9, 8.9, 6.9 Hz, 2H), 1.71 (p, J = 7.5 Hz, 2H), 1.51 (tt, J = 10.0, 6.3 Hz, 2H).

^{13}C NMR (101 MHz, CDCl$_3$) δ 174.3, 162.0, 140.9, 136.6, 130.5, 127.6, 127.5, 126.9, 125.5, 119.7, 51.6, 34.9, 34.1, 29.5, 29.4, 24.9.

HRMS (**EI**): *m/z* Calculated for $C_{16}H_{19}NO_2$ ([M]$^+$): 257.1416; found: 257.1403.

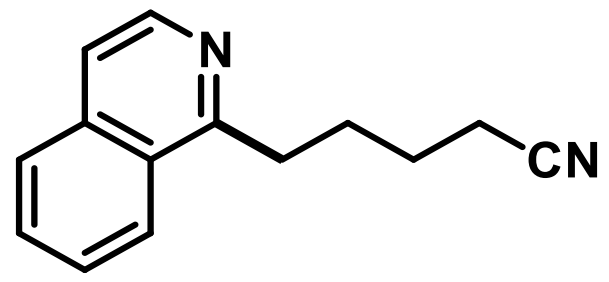

5-(isoquinolin-1-yl)pentanenitrile (4-6)[11]: Following the general procedure A, substrate **4-6** was obtained as a yellow oil (23.0 mg, 54% yield).

^{1}H NMR (400 MHz, CDCl$_3$) δ 8.42 (d, J = 5.8 Hz, 1H), 8.14 (d, J = 8.1 Hz, 1H), 7.83 (d, J = 7.9 Hz, 1H), 7.72 – 7.66 (m, 1H), 7.65 – 7.59 (m, 1H), 7.54 (d, J = 5.8 Hz, 1H), 3.36 (t, J = 7.6 Hz, 2H), 2.42 (t, J = 7.2 Hz, 2H), 2.12 – 1.99 (m, 2H), 1.91 – 1.73 (m, 2H).

^{13}C NMR (101 MHz, CDCl$_3$) δ 160.7, 141.4 (t, J = 3.4 Hz), 136.5, 130.3 (t, J = 1.8 Hz), 127.7, 127.6, 126.9, 125.1, 119.8, 119.7, 34.0, 28.2, 25.4, 17.2.

LRMS (**EI**): *m/z* Calculated for $C_{14}H_{14}N_2$ ([M]$^+$): 210.1; found: 210.1.

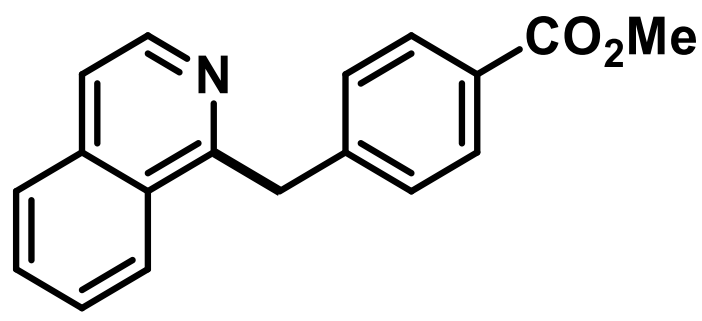

methyl 4-(isoquinolin-1-ylmethyl)benzoate (4-7)[12]: Following the general procedure A, substrate **4-7** was obtained as a yellow oil (23.0 mg, 54% yield).

^{1}H NMR (400 MHz, CDCl$_3$) δ 8.50 (d, *J* = 5.8 Hz, 1H), 8.10 (d, *J* = 8.3 Hz, 1H), 8.05 – 7.89 (m, 2H), 7.84 (d, *J* = 8.0 Hz, 1H), 7.67 (ddd, *J* = 8.2, 6.9, 1.2 Hz, 1H), 7.62 (d, *J* = 5.8 Hz, 1H), 7.55 (ddd, *J* = 8.3, 6.9, 1.3 Hz, 1H), 7.35 (d, *J* = 8.4 Hz, 2H), 4.76 (s, 2H), 3.86 (s, 3H).

^{13}C NMR (101 MHz, CDCl$_3$) δ 167.1, 159.3, 144.6, 141.3, 136.9, 130.6, 130.0, 128.8, 128.5, 127.8, 127.7, 127.2, 125.8, 120.5, 52.1, 41.7.

LRMS (**EI**): *m/z* Calculated for $C_{18}H_{15}NO_2$ ([M]$^+$): 277.1; found: 277.1.

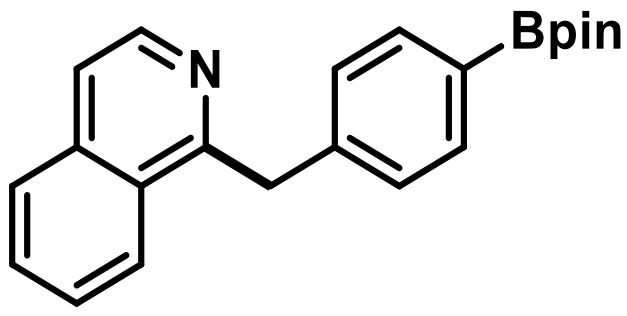

1-(4-(4,4,5,5-tetramethyl-1,3,2-dioxaborolan-2-yl)benzyl)isoquinoline (4-8): Following the general procedure A, substrate **4-8** was obtained as a yellow oil (35.2 mg, 52% yield).

^{1}H NMR (400 MHz, CDCl$_3$) δ 8.50 (d, *J* = 5.8 Hz, 1H), 8.10 (d, *J* = 8.5 Hz, 1H), 7.80 (d, *J* = 8.2 Hz, 1H), 7.70 (d, *J* = 7.7 Hz, 2H), 7.62 (ddd, *J* = 8.1, 6.8, 1.2 Hz, 1H), 7.56 (d, *J* = 5.8 Hz, 1H), 7.49 (ddd, *J* = 8.3, 6.8, 1.3 Hz, 1H), 7.29 (d, *J* = 7.7 Hz, 2H), 4.69 (s, 2H), 1.30 (s, 12H).

^{13}C NMR (101 MHz, CDCl$_3$) δ 160.0, 142.9, 142.1, 136.7, 135.2, 130.0, 128.2, 127.5, 127.4, 127.3, 125.9, 120.0, 83.8, 42.6, 25.0.

HRMS (**EI**): *m/z* Calculated for $C_{22}H_{24}BNO_2$ ([M]$^+$): 345.1900; found: 345.1894.

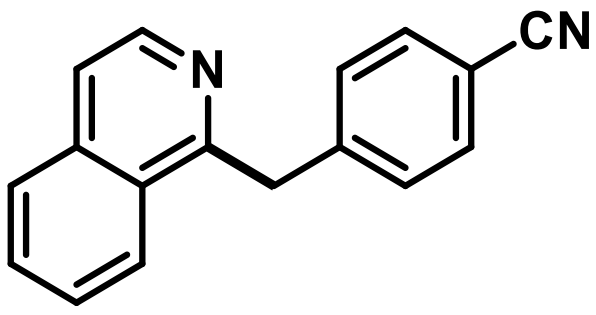

4-(isoquinolin-1-ylmethyl)benzonitrile (4-9)[12]: Following the general procedure A, substrate **4-9** was obtained as a yellow oil (19.7 mg, 41% yield).

^{1}H NMR (400 MHz, CDCl$_3$) δ 8.49 (d, *J* = 5.8 Hz, 1H), 8.07 (d, *J* = 8.4 Hz, 1H), 7.86 (d, *J* = 8.2 Hz, 1H), 7.70 (ddd, *J* = 8.2, 6.9, 1.2 Hz, 1H), 7.63 (d, *J* = 5.8 Hz, 1H), 7.62 – 7.52 (m, 3H), 7.39 (d, *J* = 8.1 Hz, 2H), 4.75 (s, 2H).

^{13}C NMR (101 MHz, CDCl$_3$) δ 158.6, 144.7, 141.4 (t, *J* = 5.7 Hz), 136.9, 132.5, 130.7, 129.6, 128.0, 127.8, 127.1, 125.4, 120.7, 119.0, 110.5, 41.5.

LRMS (**EI**): *m/z* Calculated for $C_{17}H_{12}N_2$ ([M]$^+$): 244.1; found: 244.1.

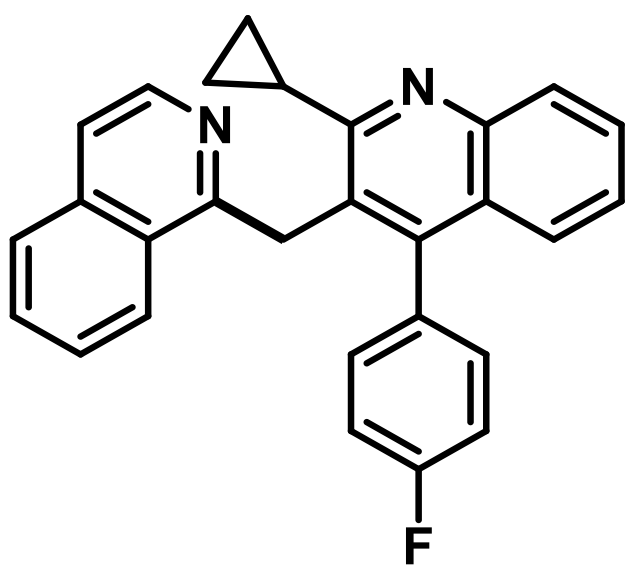

2-cyclopropyl-4-(4-fluorophenyl)-3-(isoquinolin-1-ylmethyl)quinoline (4-10): Following the general procedure A, substrate **4-10** was obtained as a colorless oil (41 mg, 50% yield).
^{1}H NMR (400 MHz, CDCl$_3$) δ 8.35 (d, *J* = 5.7 Hz, 1H), 8.13 – 8.04 (m, 2H), 7.83 (d, *J* = 8.2 Hz, 1H), 7.68 (ddd, *J* = 8.2, 6.9, 1.2 Hz, 1H), 7.62 – 7.53 (m, 2H), 7.49 (d, *J* = 5.8 Hz, 1H), 7.33 (d, *J* = 3.7 Hz, 2H), 7.30 – 7.25 (m, 2H), 7.06 – 6.96 (m, 2H), 4.77 (s, 2H), 1.94 (tt, *J* = 8.2, 4.9 Hz, 1H), 1.29 – 1.22 (m, 2H), 0.78 – 0.67 (m, 2H).
^{13}C NMR (101 MHz, CDCl$_3$) δ 163.7, 162.4, 161.2, 159.5, 146.4 (d, *J* = 42.3 Hz), 142.2, 136.1, 133.6 (d, *J* = 3.4 Hz), 131.3, 131.2, 130.0, 129.5, 128.9, 128.5, 127.6, 127.4, 127.0, 126.9, 126.2, 125.4, 124.5, 119.4, 115.5, 115.3, 35.5, 15.8, 9.1.
^{19}F NMR (376 MHz, CDCl$_3$) δ -114.89 (s, 1F).
HRMS (**ESI**): *m/z* Calculated for $C_{28}H_{21}FN_2$ ($[M+H]^+$): 405.1762; found: 405.1767.

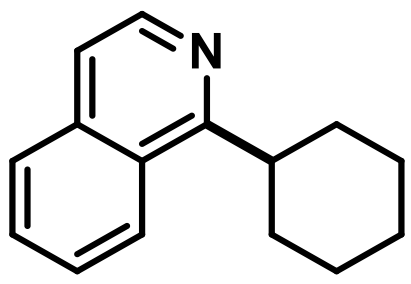

1-cyclohexylisoquinoline (4-11)[13]**:** Following the general procedure A, substrate **4-11** was obtained as a yellow oil (25.3 mg, 60% yield).
^{1}H NMR (400 MHz, CDCl$_3$) δ 8.49 (d, *J* = 5.7 Hz, 1H), 8.24 (d, *J* = 8.5 Hz, 1H), 7.82 (d, *J* = 8.2 Hz, 1H), 7.68 (ddd, *J* = 8.1, 6.8, 1.2 Hz, 1H), 7.60 (ddd, *J* = 8.3, 6.8, 1.4 Hz, 1H), 7.51 (d, *J* = 5.7 Hz, 1H), 3.58 (tt, *J* = 11.6, 3.4 Hz, 1H), 2.08 – 1.77 (m, 7H), 1.63 – 1.34 (m, 3H).
^{13}C NMR (101 MHz, CDCl$_3$) δ 165.7, 141.4, 136.7, 130.1, 127.8, 127.2, 126.4, 125.0, 119.3, 41.6, 32.7, 27.0, 26.3.
LRMS (**EI**): *m/z* Calculated for $C_{15}H_{17}N$ ($[M]^+$): 211.1; found: 211.1.

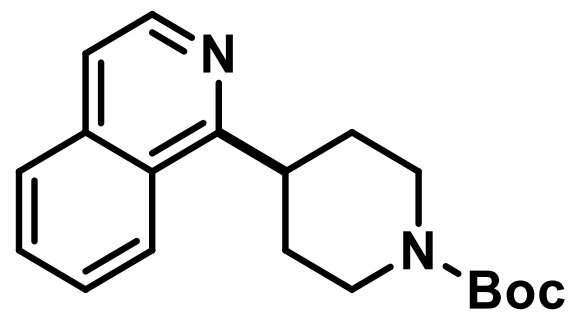

tert-butyl 4-(isoquinolin-1-yl)piperidine-1-carboxylate (4-12)[14]: Following the general procedure A, substrate **4-12** was obtained as a yellow oil (39.5 mg, 63% yield).

^{1}H NMR (400 MHz, CDCl$_3$) δ 8.48 (d, *J* = 5.7 Hz, 1H), 8.21 (d, *J* = 8.4 Hz, 1H), 7.84 (d, *J* = 8.1 Hz, 1H), 7.69 (ddd, *J* = 8.2, 6.8, 1.2 Hz, 1H), 7.62 (ddd, *J* = 8.3, 6.8, 1.4 Hz, 1H), 7.53 (d, *J* = 5.7 Hz, 1H), 4.33 (s, 2H), 3.88 – 3.60 (m, 1H), 3.10 – 2.84 (m, 2H), 2.18 – 1.86 (m, 4H), 1.48 (s, 9H).

^{13}C NMR (101 MHz, CDCl$_3$) δ 163.5, 154.9, 141.5, 136.7, 130.2, 127.9, 127.4, 126.3, 124.5, 119.7, 79.5, 44.0, 39.9, 31.5, 28.6.

LRMS (**EI**): *m/z* Calculated for $C_{19}H_{24}N_2O_2$ ($[M]^+$): 312.2; found: 312.2.

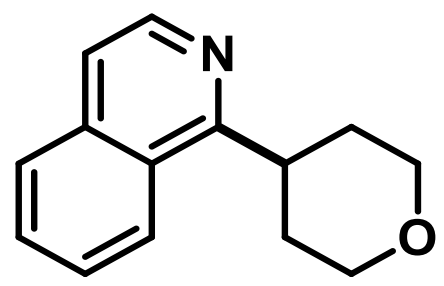

1-(tetrahydro-2H-pyran-4-yl)isoquinoline (4-13)[15]: Following the general procedure A, substrate **4-13** was obtained as a yellow solid (25 mg, 59% yield).

^{1}H NMR (400 MHz, CDCl$_3$) δ 8.51 (d, *J* = 5.8 Hz, 1H), 8.23 (d, *J* = 8.4 Hz, 1H), 7.85 (d, *J* = 8.0 Hz, 1H), 7.70 (ddd, *J* = 8.2, 6.8, 1.2 Hz, 1H), 7.62 (ddd, *J* = 8.3, 6.9, 1.4 Hz, 1H), 7.55 (d, *J* = 5.7 Hz, 1H), 4.21 – 4.13 (m, 2H), 3.83 (tt, *J* = 11.7, 3.6 Hz, 1H), 3.69 (td, *J* = 11.9, 2.0 Hz, 2H), 2.27 (dtd, *J* = 13.6, 12.0, 4.3 Hz, 2H), 1.88 (ddt, *J* = 13.0, 5.5, 1.9 Hz, 2H).

^{13}C NMR (101 MHz, CDCl$_3$) δ 163.4, 141.4, 136.7, 130.2, 127.9, 127.4, 126.2, 124.6, 119.7, 68.4, 39.1, 32.3.

LRMS (**EI**): *m/z* Calculated for $C_{14}H_{15}NO$ ($[M]^+$): 213.1; found: 213.1.

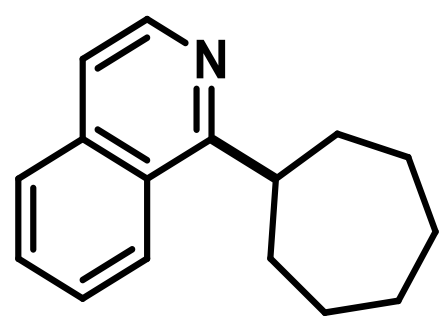

1-cycloheptylisoquinoline (4-14)[15]: Following the general procedure A, substrate **4-14** was obtained as a yellow oil (30.7 mg, 68% yield).

^{1}H NMR (400 MHz, CDCl$_3$) δ 8.47 (d, *J* = 5.7 Hz, 1H), 8.21 (d, *J* = 8.4 Hz, 1H), 7.81 (d, *J* = 8.2 Hz, 1H), 7.66 (ddd, *J* = 8.2, 6.8, 1.3 Hz, 1H), 7.59 (ddd, *J* = 8.3, 6.8, 1.4 Hz, 1H), 7.48 (d, *J* = 5.7 Hz, 1H), 3.73 (tt, *J* = 9.1, 4.4 Hz, 1H), 2.16 – 1.86 (m, 6H), 1.86 – 1.56 (m, 6H).

^{13}C NMR (101 MHz, CDCl$_3$) δ 167.3, 141.6, 136.7, 129.9, 127.7, 127.1, 126.0, 125.1, 119.0, 43.4, 34.7, 28.1, 27.7.

LRMS (**EI**): *m/z* Calculated for $C_{16}H_{19}N$ ($[M]^+$): 225.2; found: 225.1.

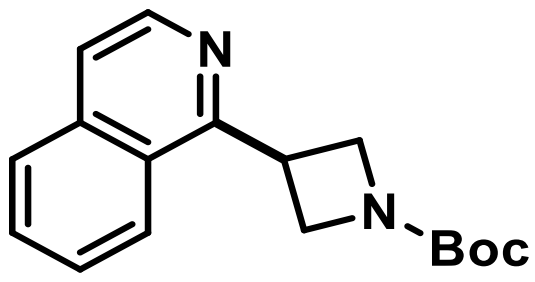

tert-butyl 3-(isoquinolin-1-yl)azetidine-1-carboxylate (4-15): Following the general procedure A, substrate **4-15** was obtained as a yellow oil (30.7 mg, 68% yield).

^{1}H NMR (400 MHz, CDCl$_3$) δ 8.51 (d, *J* = 5.8 Hz, 1H), 7.85 (d, *J* = 6.0 Hz, 2H), 7.69 (t, *J* = 7.5 Hz, 1H), 7.64 – 7.54 (m, 2H), 4.65 – 4.33 (m, 5H), 1.46 (s, 9H).

^{13}C NMR (101 MHz, CDCl$_3$) δ 159.3, 156.6, 141.8, 136.3, 130.1, 127.7, 127.4, 126.4, 124.2, 119.9, 79.4, 53.5, 32.4, 28.4.

HRMS (**ESI**): *m/z* Calculated for $C_{17}H_{20}N_2O_2$ ($[M+H]^+$): 285.1598; found: 285.1603.

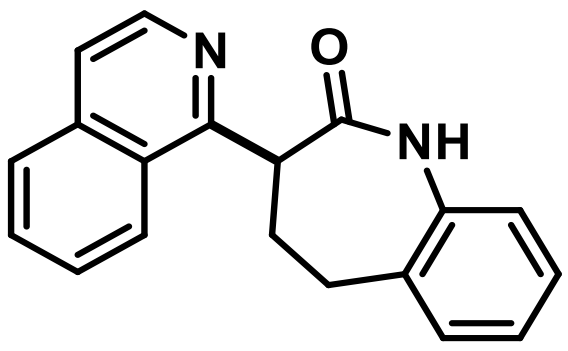

3-(isoquinolin-1-yl)-1,3,4,5-tetrahydro-2H-benzo[b]azepin-2-one (4-16): Following the general procedure A, substrate **4-16** was obtained as a yellow solid (30.6 mg, 24% yield).

^{1}H NMR (400 MHz, DMSO-d6) δ 9.94 (s, 1H), 8.44 (d, *J* = 5.7 Hz, 1H), 7.93 (d, *J* = 8.2 Hz, 1H), 7.74 – 7.63 (m, 3H), 7.64 – 7.56 (m, 1H), 7.38 – 7.30 (m, 2H), 7.22 (d, *J* = 8.0 Hz, 1H), 7.17 (td, *J* = 7.4, 1.3 Hz, 1H), 4.48 (dd, *J* = 12.0, 7.2 Hz, 1H), 3.27 (tdd, *J* = 13.4, 6.6, 2.5 Hz, 1H), 2.99 – 2.83 (m, 2H), 2.39 – 2.25 (m, 1H).

^{13}C NMR (101 MHz, DMSO-d6) δ 173.2, 157.3, 141.1, 138.5, 135.5, 134.5, 129.8, 129.7, 127.5, 127.44, 127.42, 126.9, 125.2, 124.0, 122.0, 119.9, 45.6, 32.3, 29.4.

HRMS (**EI**): *m/z* Calculated for $C_{19}H_{16}N_2O$ ($[M]^+$): 288.1263; found: 288.1257.

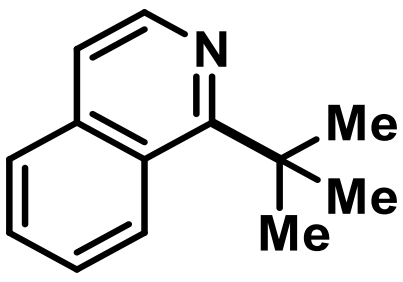

1-(tert-butyl)isoquinoline (4-17)[16]: Following the general procedure A, substrate **4-17** was obtained as a yellow oil (15.2 mg, 41% yield).

^{1}H NMR (400 MHz, CDCl$_3$) δ 8.55 (d, *J* = 8.6 Hz, 1H), 8.47 (d, *J* = 5.6 Hz, 1H), 7.84 (d, *J* = 8.5 Hz, 1H), 7.67 – 7.61 (m, 1H), 7.56 (ddd, *J* = 8.5, 6.8, 1.5 Hz, 1H), 7.52 (d, *J* = 5.7 Hz, 1H), 1.68 (s, 9H).

^{13}C NMR (101 MHz, CDCl$_3$) δ 167.4, 140.3, 137.7, 129.3, 128.4, 127.6, 126.3, 126.1, 120.1, 40.0, 31.4.

LRMS (**EI**): *m/z* Calculated for $C_{13}H_{15}N$ ($[M]^+$): 185.1; found: 185.1.

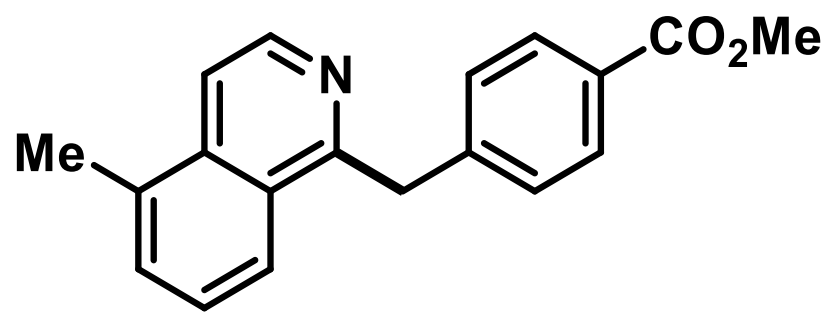

methyl 4-((5-methylisoquinolin-1-yl)methyl)benzoate (4-18): Following the general procedure A, substrate **4-18** was obtained as a yellow oil (34.7 mg, 60% yield).

^{1}H NMR (400 MHz, CDCl$_3$) δ 8.53 (d, *J* = 6.0 Hz, 1H), 7.97 – 7.88 (m, 3H), 7.71 (dd, *J* = 5.9, 1.0 Hz, 1H), 7.47 (dt, *J* = 7.0, 1.1 Hz, 1H), 7.40 (dd, *J* = 8.4, 7.0 Hz, 1H), 7.32 (d, *J* = 8.4 Hz, 2H), 4.72 (s, 2H), 3.86 (s, 3H), 2.67 (s, 3H).

^{13}C NMR (101 MHz, CDCl$_3$) δ 167.1, 159.8, 145.1, 142.1, 136.2, 134.5, 130.5, 130.0, 128.8, 128.3, 127.3, 127.0, 123.7, 116.6, 52.1, 42.4, 19.1.

HRMS (**EI**): *m/z* Calculated for $C_{19}H_{17}NO_2$ ([M]$^+$): 291.1259; found: 291.1232.

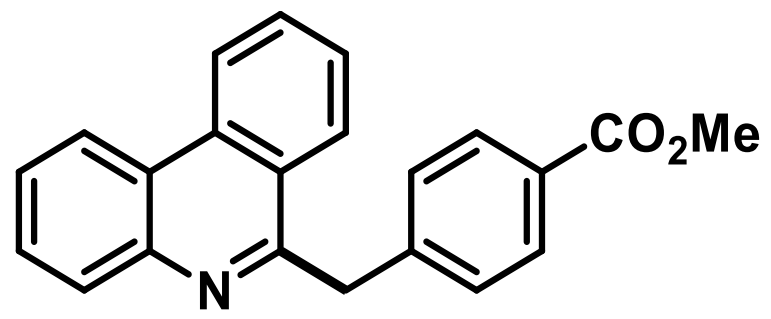

methyl 4-(phenanthridin-6-ylmethyl)benzoate (4-19)[17]**:** Following the general procedure A, substrate **4-19** was obtained as a yellow oil (55.8 mg, 85% yield).

^{1}H NMR (400 MHz, CDCl$_3$) δ 8.62 (d, *J* = 8.3 Hz, 1H), 8.56 (dd, *J* = 8.2, 1.4 Hz, 1H), 8.21 (dd, *J* = 8.1, 1.4 Hz, 1H), 8.11 (d, *J* = 7.9 Hz, 1H), 7.95 – 7.89 (m, 2H), 7.77 (dddd, *J* = 9.6, 8.3, 7.0, 1.4 Hz, 2H), 7.72 – 7.62 (m, 1H), 7.57 (ddd, *J* = 8.3, 7.0, 1.2 Hz, 1H), 7.38 (d, *J* = 8.4 Hz, 2H), 4.81 (s, 2H), 3.86 (s, 3H).

^{13}C NMR (101 MHz, CDCl$_3$) δ 167.0, 159.4, 144.5, 143.6, 133.5, 130.7, 130.0, 129.7 (t, *J* = 23.5 Hz), 128.9, 128.7, 128.5, 127.6, 127.1, 126.9, 125.3, 124.1, 122.7, 122.1, 52.1, 43.0.

LRMS (**EI**): *m/z* Calculated for $C_{22}H_{27}NO_2$ ([M]$^+$): 327.1; found: 327.1.

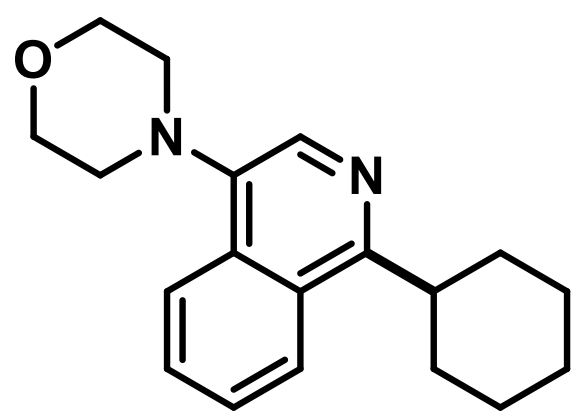

4-(1-cyclohexylisoquinolin-4-yl)morpholine (4-20): Following the general procedure A, substrate **4-20** was obtained as a brown solid (31.5 mg, 53% yield).

^{1}H NMR (400 MHz, CDCl$_3$) δ 8.26 – 8.16 (m, 3H), 7.70 (ddd, *J* = 8.3, 6.8, 1.2 Hz, 1H), 7.61 (ddd, *J* = 8.3, 6.8, 1.4 Hz, 1H), 4.02 – 3.93 (m, 2H), 3.51 (tt, *J* = 11.5, 3.3 Hz, 1H), 3.20 – 3.11 (m, 2H), 2.00 – 1.89 (m, 4H), 1.85 – 1.77 (m, 3H), 1.63 – 1.44 (m, 2H), 1.45 – 1.34 (m, 1H).

^{13}C NMR (101 MHz, CDCl$_3$) δ 161.1, 141.8, 132.3, 131.0, 129.9 – 129.3 (m), 127.1, 126.8, 125.5, 123.4, 67.4, 53.3, 41.3, 32.7, 27.0, 26.3.

HRMS (**ESI**): *m/z* Calculated for $C_{19}H_{24}N_2O$ ([M+H]$^+$): 297.1961; found: 297.1967.

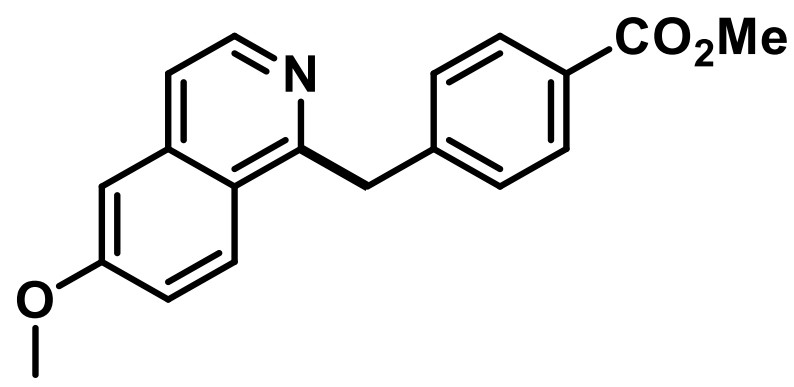

methyl 4-((6-methoxyisoquinolin-1-yl)methyl)benzoate (4-21): Following the general procedure A, substrate **4-21** was obtained as a orange oil (42.2 mg, 69% yield).

^{1}H NMR (400 MHz, CDCl$_3$) δ 8.41 (d, J = 5.8 Hz, 1H), 7.98 (d, J = 9.3 Hz, 1H), 7.91 (d, J = 8.2 Hz, 2H), 7.50 (d, J = 5.9 Hz, 1H), 7.32 (d, J = 8.1 Hz, 2H), 7.16 (dd, J = 9.2, 2.6 Hz, 1H), 7.07 (d, J = 2.6 Hz, 1H), 4.69 (s, 2H), 3.92 (s, 3H), 3.85 (s, 3H).

^{13}C NMR (101 MHz, CDCl$_3$) δ 167.0, 161.0, 158.5, 144.7, 141.8, 139.1, 130.0, 128.7, 128.5, 127.6, 122.8, 120.6, 119.8, 105.2, 55.6, 52.1, 41.6.

HRMS (EI): *m/z* Calculated for $C_{19}H_{17}NO_3$ ($[M]^+$): 307.1208; found: 307.1202.

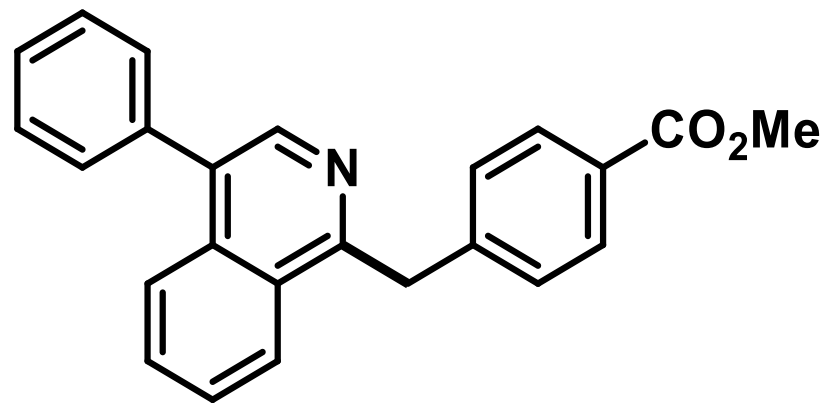

methyl 4-((4-phenylisoquinolin-1-yl)methyl)benzoate (4-22): Following the general procedure A, substrate **4-22** was obtained as a yellow solid (56.6 mg, 80% yield).

^{1}H NMR (400 MHz, CDCl$_3$) δ 8.46 (s, 1H), 8.19 (d, J = 7.9 Hz, 1H), 8.04 – 7.91 (m, 3H), 7.65 (ddd, J = 8.4, 6.9, 1.3 Hz, 1H), 7.58 (ddd, J = 8.3, 6.9, 1.3 Hz, 1H), 7.56 – 7.45 (m, 5H), 7.42 (d, J = 8.4 Hz, 2H), 4.82 (s, 2H), 3.87 (s, 3H).

^{13}C NMR (101 MHz, CDCl$_3$) δ 167.0, 158.4, 144.5, 140.7, 136.8, 135.4, 133.3, 130.8, 130.3, 130.1, 128.9, 128.8, 128.6, 128.2, 127.7, 126.9, 126.03, 126.02, 52.1, 41.6.

HRMS (EI): *m/z* Calculated for $C_{24}H_{19}NO_2$ ($[M]^+$): 353.1416; found: 353.1410.

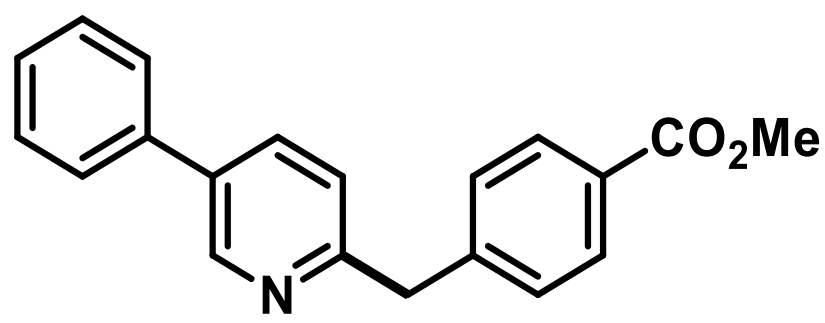

methyl 4-((5-phenylpyridin-2-yl)methyl)benzoate (4-23): Following the general procedure A, substrate **4-23** was obtained as a orange solid (12.2 mg, 20% yield).

^{1}H NMR (400 MHz, CDCl$_3$) δ 8.79 (d, J = 2.0 Hz, 1H), 8.00 (d, J = 8.3 Hz, 2H), 7.86 (dd, J = 8.1, 2.4 Hz, 1H), 7.62 – 7.53 (m, 2H), 7.50 – 7.45 (m, 2H), 7.44 – 7.36 (m, 3H), 7.23 (d, J = 8.1 Hz, 1H), 4.31 (s, 2H), 3.90 (s, 3H).

^{13}C NMR (101 MHz, CDCl$_3$) δ 167.1, 158.3, 147.0, 144.4, 137.2, 136.2, 135.2, 130.2, 129.33, 129.31, 128.8, 128.4, 127.2, 123.6, 52.2, 43.7.

HRMS (EI): *m/z* Calculated for $C_{20}H_{17}NO_2$ ($[M]^+$): 303.1259; found: 303.1253.

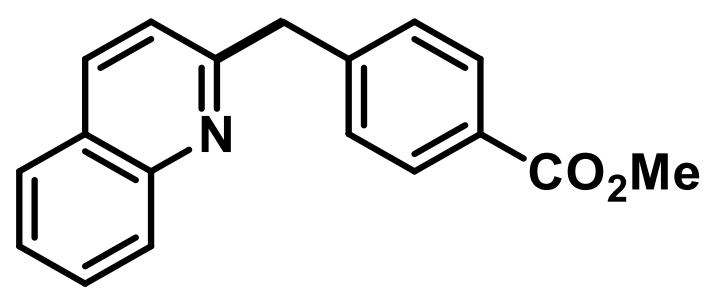

methyl 4-(quinolin-2-ylmethyl)benzoate (4-24): Following the general procedure A, substrate **4-24** was obtained as a yellow oil (15.4 mg, 28% yield).

^{1}H NMR (400 MHz, CDCl$_3$) δ 8.14 (d, J = 8.5 Hz, 1H), 8.08 (d, J = 8.1 Hz, 1H), 8.04 – 7.91 (m, 2H), 7.79 (d, J = 8.2 Hz, 1H), 7.73 (ddd, J = 8.4, 6.9, 1.4 Hz, 1H), 7.53 (ddd, J = 8.2, 6.9, 1.2 Hz, 1H), 7.42 – 7.36 (m, 2H), 7.22 (d, J = 8.5 Hz, 1H), 4.43 (s, 2H), 3.89 (s, 3H).

^{13}C NMR (101 MHz, CDCl$_3$) δ 167.1, 160.2, 144.4, 137.3, 130.1, 130.0, 129.6, 129.4, 128.8, 128.7, 127.7, 127.0, 126.5, 121.6, 52.2, 45.3.

HRMS (**EI**): *m/z* Calculated for $C_{18}H_{15}NO_2$ ([M]$^+$): 277.1103; found: 277.1084.

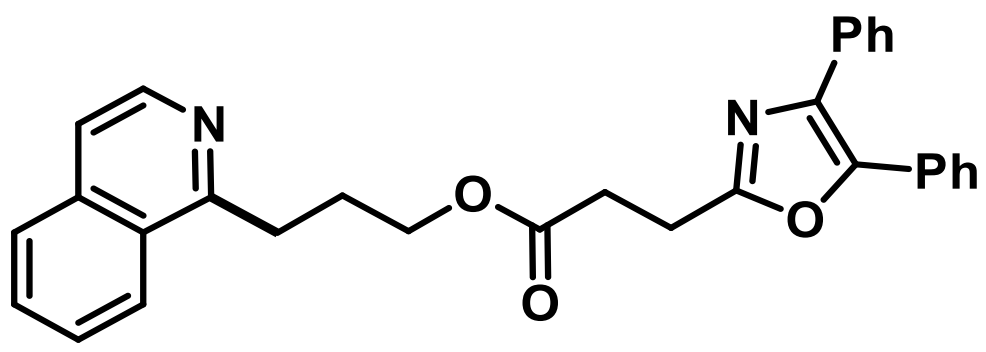

3-(isoquinolin-1-yl)propyl 3-(4,5-diphenyloxazol-2-yl)propanoate (4-25): Following the general procedure A, substrate **4-25** was obtained as a yellow oil (52.2 mg, 56% yield).

^{1}H NMR (400 MHz, CDCl$_3$) δ 8.41 (d, J = 5.9 Hz, 1H), 8.13 (d, J = 8.6 Hz, 1H), 7.84 (d, J = 7.9 Hz, 1H), 7.70 (ddd, J = 8.2, 6.8, 1.2 Hz, 1H), 7.64 – 7.51 (m, 6H), 7.41 – 7.23 (m, 6H), 4.30 (t, J = 6.3 Hz, 2H), 3.52 – 3.36 (m, 2H), 3.18 (dd, J = 8.1, 6.9 Hz, 2H), 2.91 (dd, J = 8.1, 6.8 Hz, 2H), 2.34 – 2.23 (m, 2H).

^{13}C NMR (101 MHz, CDCl$_3$) δ 172.1, 161.9, 160.6, 145.5, 139.9, 136.7, 135.2, 132.5, 131.0, 129.0, 128.7, 128.6, 128.5, 128.1, 128.0, 127.9, 127.7, 126.9, 126.5, 125.4, 120.2, 64.5, 31.2, 30.8, 28.3, 23.6.

HRMS (**ESI**): *m/z* Calculated for $C_{30}H_{26}N_2O_3$ ([M+H]$^+$): 463.2016; found: 463.2020.

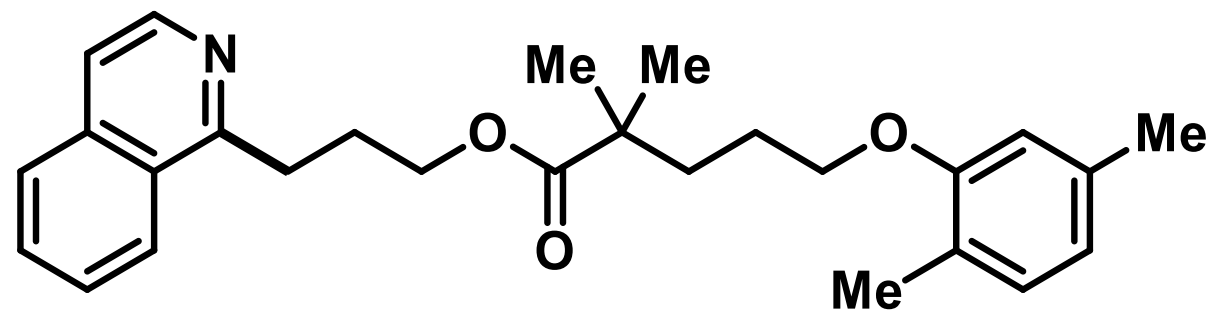

3-(isoquinolin-1-yl)propyl 5-(2,5-dimethylphenoxy)-2,2-dimethylpentanoate (4-26): Following the general procedure A, substrate **4-26** was obtained as a yellow oil (54.7 mg, 65% yield).

^{1}H NMR (400 MHz, CDCl$_3$) δ 8.44 (d, *J* = 5.8 Hz, 1H), 8.15 (d, *J* = 9.2 Hz, 1H), 7.83 (d, *J* = 8.1 Hz, 1H), 7.68 (ddd, *J* = 8.2, 6.9, 1.2 Hz, 1H), 7.58 (ddd, *J* = 8.5, 6.9, 1.4 Hz, 1H), 7.55 (d, *J* = 5.8 Hz, 1H), 6.97 (d, *J* = 7.4 Hz, 1H), 6.64 (d, *J* = 7.0 Hz, 1H), 6.59 (s, 1H), 4.24 (t, *J* = 6.4 Hz, 2H), 3.91 (t, *J* = 5.3 Hz, 2H), 3.52 – 3.35 (m, 2H), 2.32 – 2.22 (m, 5H), 2.15 (s, 3H), 1.82 – 1.66 (m, 4H), 1.24 (s, 6H).

^{13}C NMR (101 MHz, CDCl$_3$) δ 177.9, 160.7, 157.0, 141.3, 136.53, 136.46, 130.4, 130.3, 127.6, 127.5, 127.0, 125.1, 123.7, 120.8, 119.8, 112.1, 68.0, 64.3, 42.2, 37.3, 31.3, 28.3, 25.3, 21.5, 15.9.

HRMS (**ESI**): *m/z* Calculated for $C_{27}H_{33}NO_3$ ($[M+H]^+$): 420.2533; found: 420.2538.

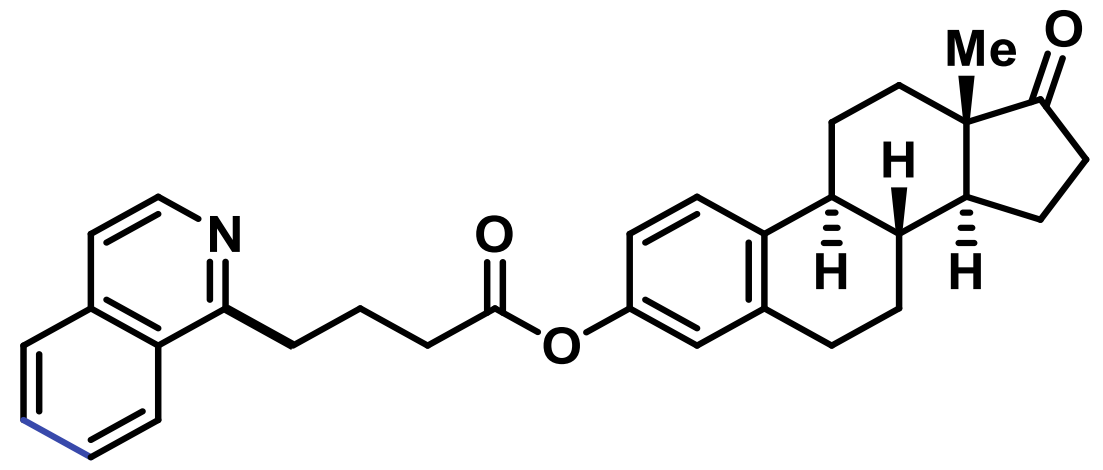

(8R,9S,13S,14S)-13-methyl-17-oxo-7,8,9,11,12,13,14,15,16,17-decahydro-6H-cyclo penta[a]phenanthren-3-yl 4-(isoquinolin-1-yl)butanoate (4-27): Following the general procedure A, substrate **4-27** was obtained as a yellow oil (53.4 mg, 57% yield).

^{1}H NMR (400 MHz, CDCl$_3$) δ 8.43 – 8.39 (m, 2H)., 7.93 (d, *J* = 8.2 Hz, 1H), 7.84 (t, *J* = 7.4 Hz, 1H), 7.77 – 7.69 (m, 2H), δ 7.28 (d, *J* = 8.6 Hz, 1H)., 6.86 (dd, *J* = 8.5, 2.6 Hz, 1H), 6.81 (d, *J* = 2.6 Hz, 1H), 3.69 – 3.61 (m, 2H), 2.91 (dd, *J* = 8.4, 3.8 Hz, 2H), 2.81 (t, *J* = 6.8 Hz, 2H), 2.65 – 2.39 (m, 1H), 2.38 (q, *J* = 5.4, 4.8 Hz, 1H), 2.35 – 2.23 (m, 3H), 2.19 – 2.10 (m, 1H), 2.09 – 1.98 (m, 2H), 1.96 (dt, *J* = 12.4, 2.8 Hz, 1H), 1.69 – 1.60 (m, 1H), 1.57 (dt, *J* = 12.9, 2.7 Hz, 2H), 1.54 – 1.40 (m, 4H), 0.90 (s, 3H).

^{13}C NMR (101 MHz, CDCl$_3$) δ 220.9, 172.4, 160.8, 148.6, 138.2, 137.6, 137.3, 132.6, 129.0, 127.9, 126.9, 126.6, 126.4, 121.7, 121.3, 118.8, 50.5, 48.0, 44.3, 38.1, 36.0, 33.6, 31.6, 29.5, 26.4, 25.7, 25.0, 21.7, 13.9.

HRMS (**ESI**): *m/z* Calculated for $C_{31}H_{33}NO_3$ ($[M+H]^+$): 468.2533; found: 468.2539.

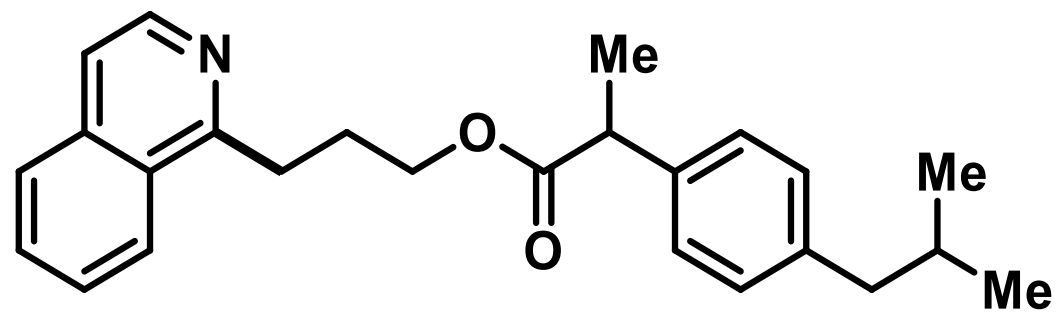

3-(isoquinolin-1-yl)propyl 2-(4-isobutylphenyl)propanoate (4-28): Following the general procedure A, substrate **4-28** was obtained as a yellow oil (45.1 mg, 60% yield).

^{1}H NMR (400 MHz, CDCl$_3$) δ 8.41 (d, *J* = 5.8 Hz, 1H), 7.96 (d, *J* = 9.0 Hz, 1H), 7.82 (d, *J* = 8.1 Hz, 1H), 7.69 (ddd, *J* = 8.1, 6.9, 1.2 Hz, 1H), 7.59 – 7.51 (m, 2H), 7.27 – 7.15 (m, 2H), 7.13 – 7.05 (m, 2H), 4.30 – 4.15 (m, 2H), 3.71 (q, *J* = 7.1 Hz, 1H), 3.33 – 3.24 (m, 2H), 2.41 (d, *J* = 7.2 Hz, 2H), 2.26 – 2.14 (m, 2H), 1.80 (dh, *J* = 13.5, 6.8 Hz, 1H), 1.50 (d, *J* = 7.2 Hz, 3H), 0.84 (d, *J* = 6.6 Hz, 6H).

^{13}C NMR (101 MHz, CDCl$_3$) δ 174.8, 160.8, 140.9, 140.6, 138.0, 136.5, 130.5, 129.5, 127.6, 127.5, 127.3, 127.0, 125.3, 119.9, 64.4, 45.3, 45.1, 31.0, 30.3, 28.3, 22.5, 18.5.

HRMS (**ESI**): *m/z* Calculated for $C_{25}H_{29}NO_2$ ([M+H]$^+$): 376.2271; found: 376.2278.

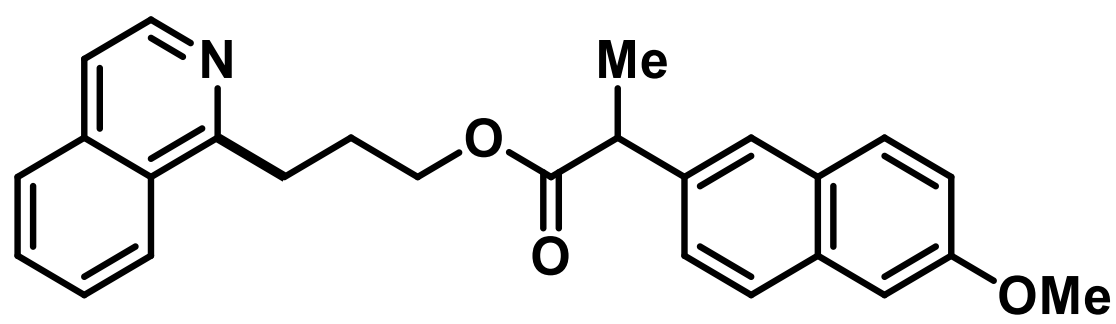

3-(isoquinolin-1-yl)propyl 2-(6-methoxynaphthalen-2-yl)propanoate (4-29): Following the general procedure A, substrate **4-29** was obtained as a yellow oil (50.4 mg, 63% yield).

^{1}H NMR (400 MHz, CDCl$_3$) δ 8.39 (d, *J* = 5.8 Hz, 1H), 7.78 (d, *J* = 9.5 Hz, 2H), 7.74 – 7.67 (m, 3H), 7.62 (ddd, *J* = 7.9, 6.8, 1.2 Hz, 1H), 7.51 (d, *J* = 5.8 Hz, 1H), 7.43 (dd, *J* = 8.6, 1.8 Hz, 1H), 7.33 (ddd, *J* = 8.4, 6.9, 1.3 Hz, 1H), 7.13 (dd, *J* = 9.0, 2.5 Hz, 1H), 7.08 (d, *J* = 2.5 Hz, 1H), 4.25 (qt, *J* = 11.0, 6.2 Hz, 2H), 3.93 – 3.82 (m, 4H), 3.29 – 3.16 (m, 2H), 2.25 – 2.11 (m, 2H), 1.59 (d, *J* = 7.2 Hz, 3H).

^{13}C NMR (101 MHz, CDCl$_3$) δ 174.7, 160.7, 157.7, 140.9, 136.4, 136.0, 133.8, 130.4, 129.4, 129.1, 127.5, 127.5, 127.3, 126.9, 126.4, 126.1, 125.1, 119.8, 119.1, 105.7, 64.5, 55.4, 45.6, 31.0, 28.2, 18.5.

HRMS (**ESI**): *m/z* Calculated for $C_{26}H_{25}NO_3$ ([M+H]$^+$): 400.1907; found: 400.1914.

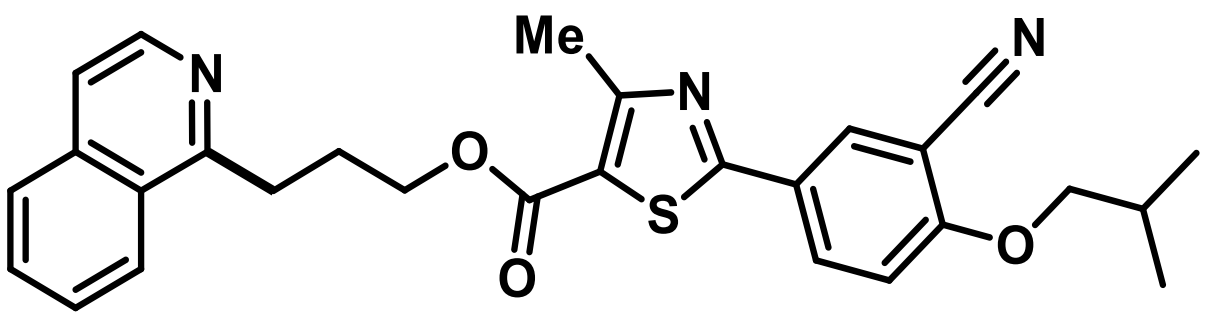

3-(isoquinolin-1-yl)propyl 2-(3-cyano-4-isobutoxyphenyl)-4-methylthiazole-5-carboxylate (4-30): Following the general procedure A, substrate **4-30** was obtained as a yellow oil (49.5 mg, 51% yield).

^{1}H NMR (400 MHz, CDCl$_3$) δ 8.43 (d, J = 5.9 Hz, 1H), 8.22 (d, J = 8.5 Hz, 1H), 8.14 (d, J = 2.3 Hz, 1H), 8.07 (dd, J = 8.8, 2.4 Hz, 1H), 7.88 (d, J = 8.1 Hz, 1H), 7.77 (t, J = 7.5 Hz, 1H), 7.67 (ddd, J = 8.3, 6.9, 1.3 Hz, 1H), 7.63 (d, J = 5.9 Hz, 1H), 7.01 (d, J = 8.9 Hz, 1H), 4.47 (t, J = 6.3 Hz, 2H), 3.89 (d, J = 6.5 Hz, 2H), 3.57 (t, J = 7.6 Hz, 2H), 2.74 (s, 3H), 2.48 – 2.37 (m, 2H), 2.27 – 2.12 (m, 1H), 1.08 (d, J = 6.7 Hz, 6H).

^{13}C NMR (101 MHz, CDCl$_3$) δ 167.2, 162.5, 161.9, 161.3, 160.3, 139.4, 136.8, 132.6, 132.1, 131.3, 128.1, 127.7, 126.9, 126.0, 125.3, 121.5, 120.4, 115.4, 112.7, 103.0, 75.7, 64.9, 30.6, 28.21, 28.16, 19.1, 17.5.

HRMS (**ESI**): *m/z* Calculated for $C_{28}H_{27}N_3O_3S$ ([M+H]$^+$): 486.1846; found: 486.1855.

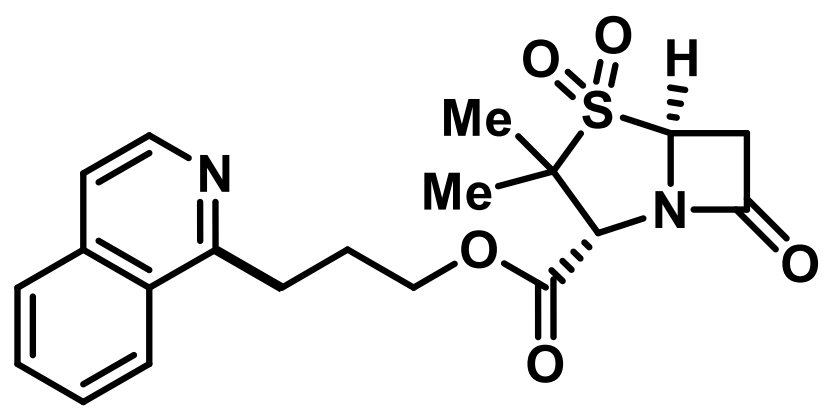

3-(isoquinolin-1-yl)propyl (2R,5S)-3,3-dimethyl-7-oxo-4-thia-1-azabicyclo[3.2.0]heptane-2-carboxylate 4,4-dioxide (4-31): Following the general procedure A, substrate **4-31** was obtained as a yellow oil (38.0 mg, 47% yield).

^{1}H NMR (400 MHz, CDCl$_3$) δ 8.42 (d, J = 5.9 Hz, 1H), 8.17 (d, J = 8.5 Hz, 1H), 7.89 (d, J = 8.1 Hz, 1H), 7.77 (ddd, J = 8.2, 6.8, 1.2 Hz, 1H), 7.70 (ddd, J = 8.3, 6.8, 1.3 Hz, 1H), 7.65 (d, J = 5.9 Hz, 1H), 4.64 (dd, J = 4.2, 2.2 Hz, 1H), 4.44 – 4.28 (m, 3H), 3.59 – 3.34 (m, 4H), 2.42 – 2.24 (m, 2H), 1.58 (s, 3H), 1.42 (s, 3H).

^{13}C NMR (101 MHz, CDCl$_3$) δ 170.8, 167.0, 159.7, 139.5, 136.7, 131.3, 128.2, 127.8, 126.8, 125.1, 120.6, 66.0, 63.3, 62.8, 61.1, 38.3, 30.2, 27.8, 20.3, 18.6.

HRMS (**ESI**): *m/z* Calculated for $C_{20}H_{22}N_2O_5S$ ([M+H]$^+$): 403.1322; found: 403.1328.

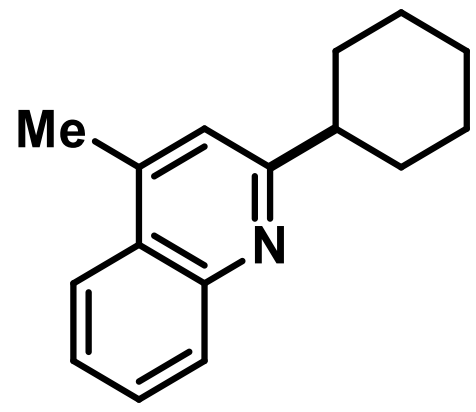

2-cyclohexyl-4-methylquinoline (6-1)[14]**:** Following the general procedure B, substrate **6-1** was obtained as a yellow oil (18.8 mg, 42% yield).

^{1}H NMR (400 MHz, CDCl$_3$) δ 8.08 (d, *J* = 8.4 Hz, 1H), 7.94 (dd, *J* = 8.4, 1.4 Hz, 1H), 7.67 (ddd, *J* = 8.4, 6.9, 1.5 Hz, 1H), 7.50 (ddd, *J* = 8.2, 6.9, 1.3 Hz, 1H), 7.18 (s, 1H), 2.91 (tt, *J* = 12.0, 3.4 Hz, 1H), 2.69 (d, *J* = 0.9 Hz, 3H), 2.07 – 1.96 (m, 2H), 1.95 – 1.84 (m, 2H), 1.84 – 1.73 (m, 1H), 1.63 (qd, *J* = 12.4, 3.1 Hz, 2H), 1.55 – 1.39 (m, 2H), 1.35 (tt, *J* = 12.6, 3.3 Hz, 1H).

^{13}C NMR (101 MHz, CDCl$_3$) δ 166.6, 147.4, 144.8, 129.4, 129.2, 127.2, 125.6, 123.7, 120.4, 47.5, 33.0, 26.7, 26.2, 19.0.

LRMS (**EI**): *m/z* Calculated for $C_{16}H_{19}N$ ($[M]^+$):225.2; found:225.2.

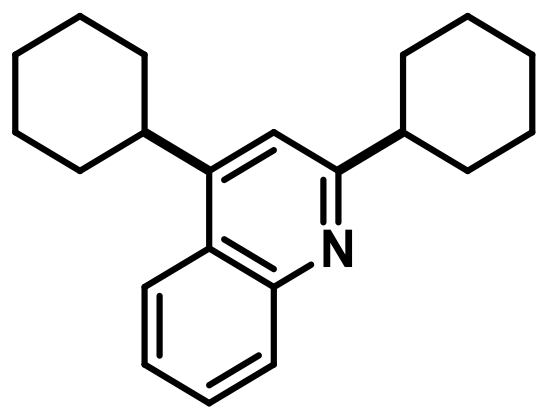

2,4-dicyclohexylquinoline (6-2)[18]**:** Following the general procedure B, substrate **6-2** was obtained as a yellow oil (13.0 mg, 44% yield).

^{1}H NMR (400 MHz, CDCl$_3$) δ 8.09 (d, *J* = 8.4 Hz, 1H), 8.03 (dd, *J* = 8.5, 1.4 Hz, 1H), 7.65 (ddd, *J* = 8.3, 6.8, 1.4 Hz, 1H), 7.48 (ddd, *J* = 8.3, 6.8, 1.4 Hz, 1H), 7.21 (s, 1H), 3.30 (ddt, *J* = 11.5, 7.4, 3.8 Hz, 1H), 2.92 (ddd, *J* = 15.4, 7.8, 3.4 Hz, 1H), 2.05 – 1.99 (m, 4H), 1.96 – 1.76 (m, 6H), 1.71 – 1.29 (m, 10H).

^{13}C NMR (101 MHz, CDCl$_3$) δ 166.5, 150.4, 147.6, 129.6, 128.8, 125.7, 125.3, 122.8, 115.8, 47.6, 39.0, 33.6, 32.9, 27.0, 26.6, 26.3, 26.1.

LRMS (**EI**): *m/z* Calculated for $C_{21}H_{27}N$ ($[M]^+$):293.2; found:293.3.

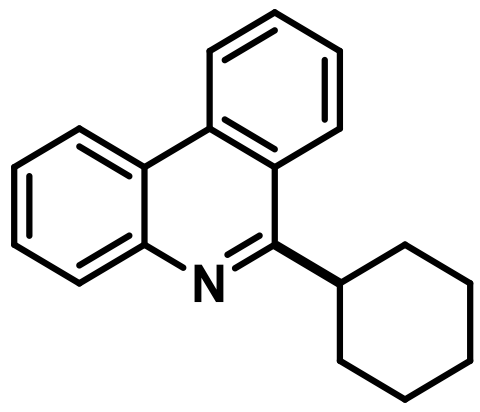

6-cyclohexylphenanthridine (6-3)[15]**:** Following the general procedure B, substrate **6-3** was obtained as a yellow oil (22.0 mg, 42% yield).

^{1}H NMR (400 MHz, CDCl$_3$) δ 8.64 (d, *J* = 1.2 Hz, 1H), 8.54 (dd, *J* = 8.2, 1.4 Hz, 1H), 8.32 (d, *J* = 8.3 Hz, 1H), 8.14 (d, *J* = 8.2 Hz, 1H), 7.86 – 7.77 (m, 1H), 7.70 (dtd, *J* = 8.0, 6.6, 1.3 Hz, 2H), 7.61 (ddd, *J* = 8.3, 7.0, 1.4 Hz, 1H), 3.62 (tt, *J* = 11.4, 3.5 Hz, 1H), 2.12 – 2.05 (m, 2H), 2.02 – 1.89 (m, 4H), 1.89 – 1.82 (m, 1H), 1.69 – 1.51 (m, 2H), 1.50 – 1.34 (m, 1H).

^{13}C NMR (101 MHz, CDCl$_3$) δ 165.4, 144.0, 133.2, 130.1, 128.5, 127.2, 126.3, 125.8, 124.9, 123.5, 122.7, 121.9, 42.1, 32.4, 27.0, 26.4.

LRMS (**EI**): *m/z* Calculated for $C_{19}H_{19}N$ ([M]$^+$):261.2; found:261.1.

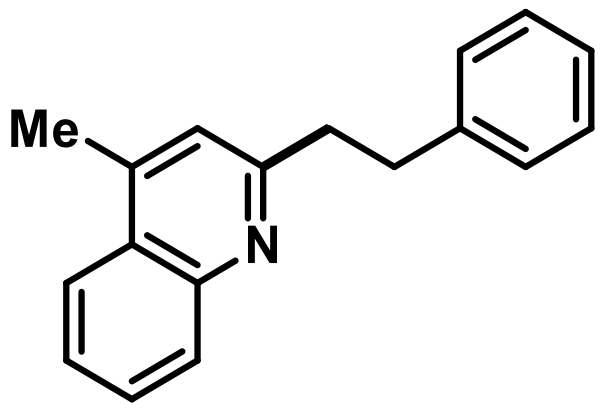

4-methyl-2-phenethylquinoline (6-4)[19]**:** Following the general procedure B, substrate **6-4** was obtained as a yellow oil (18.6 mg, 38% yield).

^{1}H NMR (400 MHz, CDCl$_3$) δ 8.26 (d, *J* = 8.4 Hz, 1H), 8.12 (dd, *J* = 8.3, 1.4 Hz, 1H), 7.85 (ddd, *J* = 8.4, 6.9, 1.5 Hz, 1H), 7.48 – 7.40 (m, 5H), 7.38 – 7.33 (m, 1H), 3.46 – 3.38 (m, 2H), 3.38 – 3.26 (m, 2H), 2.82 (d, *J* = 1.0 Hz, 3H).

^{13}C NMR (101 MHz, CDCl$_3$) δ 161.5, 147.6, 144.8, 141.7, 129.4, 129.3, 128.6, 128.5, 127.0, 126.1, 125.8, 123.8, 122.4, 40.9, 36.1, 18.8.

LRMS (**EI**): *m/z* Calculated for $C_{18}H_{17}N$ ([M]$^+$):247.1; found: 247.1.

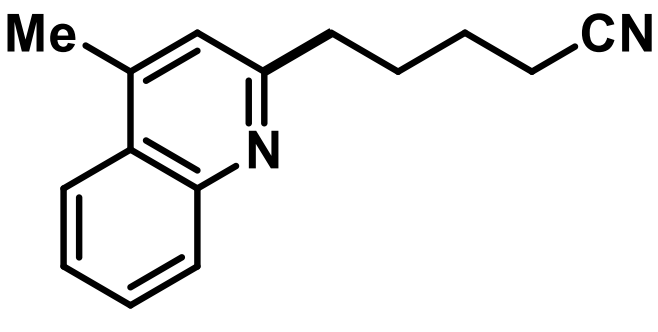

5-(4-methylquinolin-2-yl)pentanenitrile (6-5)[20]**:** Following the general procedure B, substrate **6-5** was obtained as a yellow oil (14.0 mg, 32% yield).

^{1}H NMR (400 MHz, CDCl$_3$) δ 8.05 (d, *J* = 8.3 Hz, 1H), 7.96 (dd, *J* = 8.4, 0.9 Hz, 1H), 7.69 (ddd, *J* = 8.4, 6.9, 1.5 Hz, 1H), 7.53 (ddd, *J* = 8.2, 6.8, 1.3 Hz, 1H), 7.15 (d, *J* = 1.0 Hz, 1H), 3.02 – 2.94 (m, 2H), 2.69 (d, *J* = 0.9 Hz, 3H), 2.40 (t, *J* = 7.1 Hz, 2H), 2.06 – 1.95 (m, 2H), 1.83 – 1.71 (m, 2H).

^{13}C NMR (101 MHz, CDCl$_3$) δ 161.1, 147.5, 145.2, 129.5, 129.2, 127.0, 126.0, 123.8, 122.0, 119.8, 37.9, 28.7, 25.2, 18.9, 17.2.

LRMS (**EI**): *m/z* Calculated for $C_{15}H_{16}N_2$ ([M]$^+$):224.1; found:224.2.

5.2 Scale-up reaction

5.2.1 Gram-scale reaction

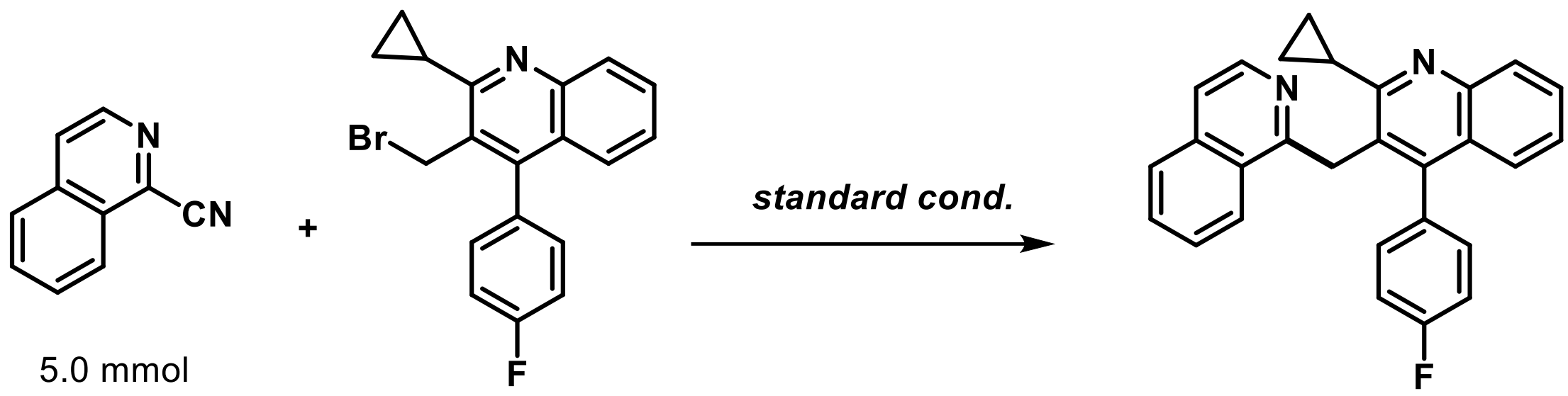

5.0 mmol

1.6 equiv.

4-10, 51% (1.032 g)

General procedure: 1-isoquinolinecarbonitrile (771 mg, 5 mmol), $(TMS)_3SiNHPh$ (3.05mL, 8 mmol) 3-(bromomethyl)-2-cyclopropyl-4-(4-fluorophenyl)quinoline (2.849 g, 8 mmol) and NEt_3 (0.7mL, 5 mmol) were added to an oven-dried 50 mL flask equipped with a magnetic stirring bar. The flask was capped, evacuated, and backfilled with N_2 three times and 50 mL DMA was added to the flask by syringe. Then, the cap was sealed with parafilm. The reaction mixture was stirred vigorously and irradiated with a 365 nm (2 x 18W) LED at 25 °C (cooled by fens) for 3 h. Then diluted with Na_2CO_3 solution and extracted with diethyl ether (3 x 30 mL) and washed with saturated brine (15 mL). The combined organic layers were dried over Na_2SO_4, filtered, and concentrated under reduced pressure. The crude product was purified by column chromatography (PE/EA = 5:1) to afford the desired product **4-10** as a yellow solid (1.032 g, 51% yield).

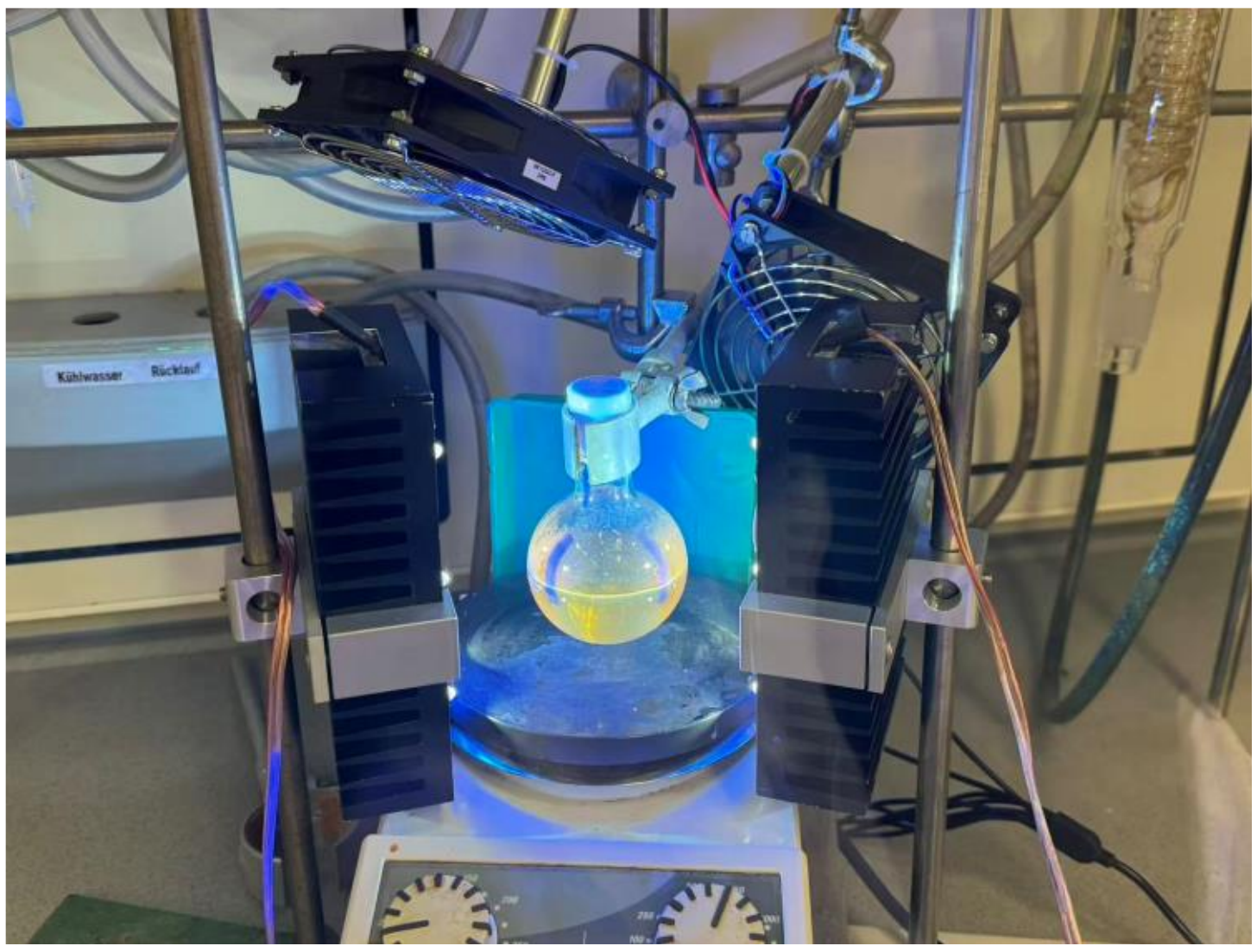

Figure S20. Set up for gram scale reaction

5.2.2 Gram-scale reaction with flow reactor

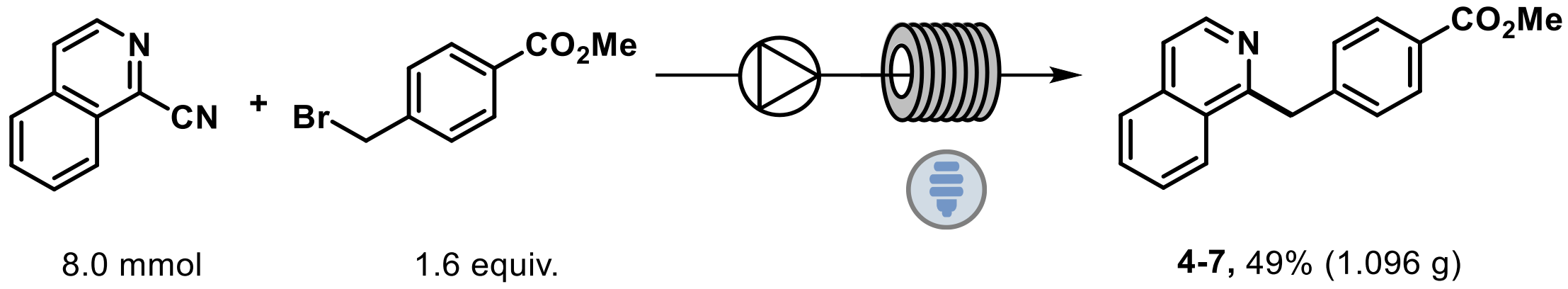

General procedure: 1-isoquinolinecarbonitrile (1.23 g, 8 mmol), $(TMS)_3SiNHPh$ (4.9 mL, 12.8 mmol), tert-butyl methyl 4-(bromomethyl)benzoate (2.93 g, 12.8 mmol) and NEt_3 (1.11mL, 8 mmol) were added to an oven-dried 100 mL snap vial. The capped vial was evacuated and backfilled with N_2 three times and DMA (80 mL) was added to the vial by syringe. The reaction vial was connected to the inlet of a nitrogen-purged flow chemistry device via a syringe (see the picture above), close to the dosing pump. The vial was stirred in the dark till the complete dissolution of solid compounds, and the reaction mixture was continuously injected into the microreactor using a peristaltic pump (flow rate: 0.4 mL/min). The reaction proceeds in a recirculated mode under the irradiation of 365 nm LEDs (47.5 W) for 17 h, while the dosing pump was open. The reaction mixture was subsequently collected at the end of the device. Then, 30 mL saturated Na_2CO_3 solution was added to the reaction system and extracted with diethyl ether (30 mL x 5) and washed with saturated brine (15 mL). The combined organic layers were dried over Na_2SO_4, filtered, and concentrated under reduced pressure. The residue was purified by silica gel flash chromatography to give the desired product **4-9** as a yellow oil (1.0964 g, 49% yield).

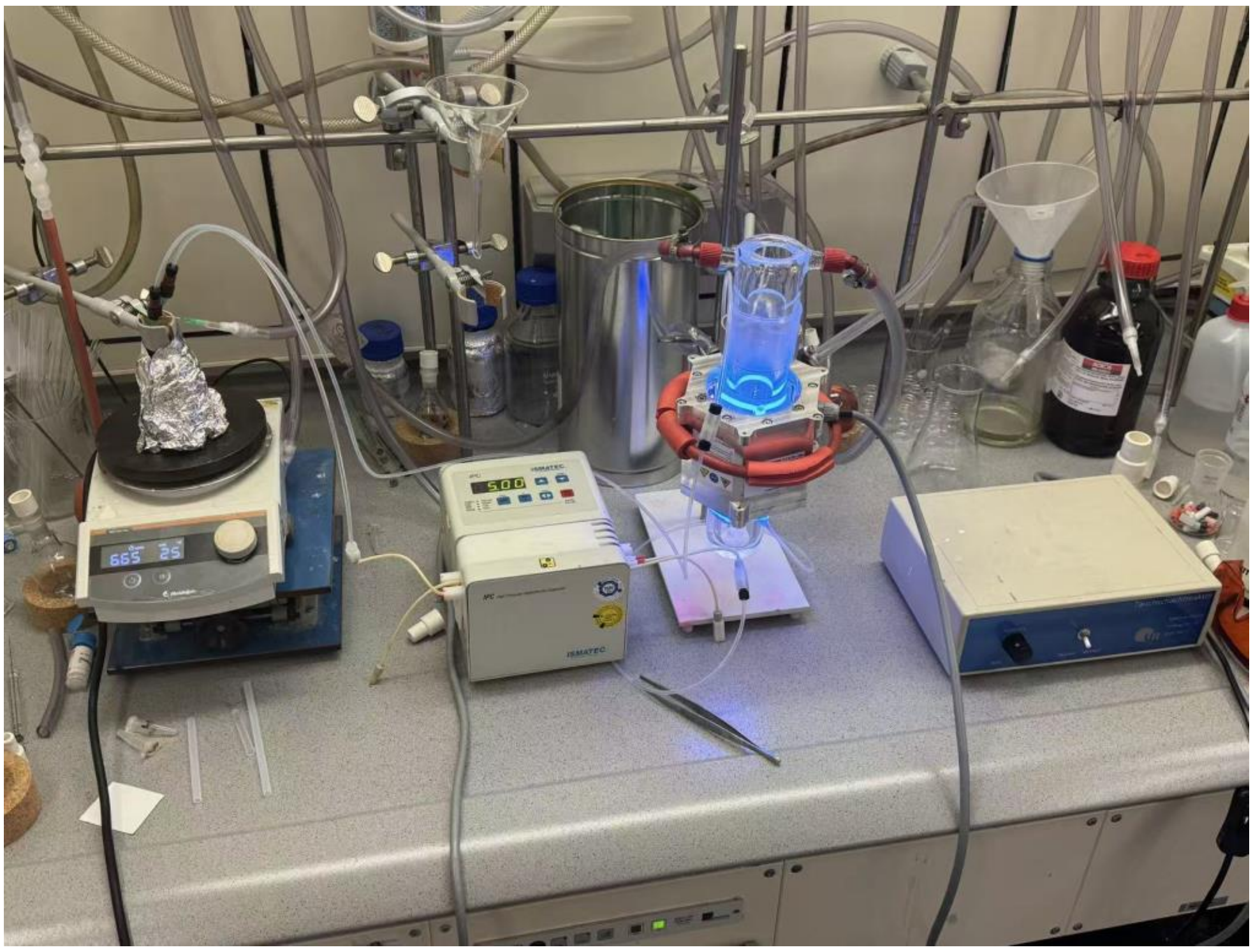

Figure S21. Set up for flow reaction

5.3 Failed substrate scope

Figure S22. Failed sp^3-Br scope[a]

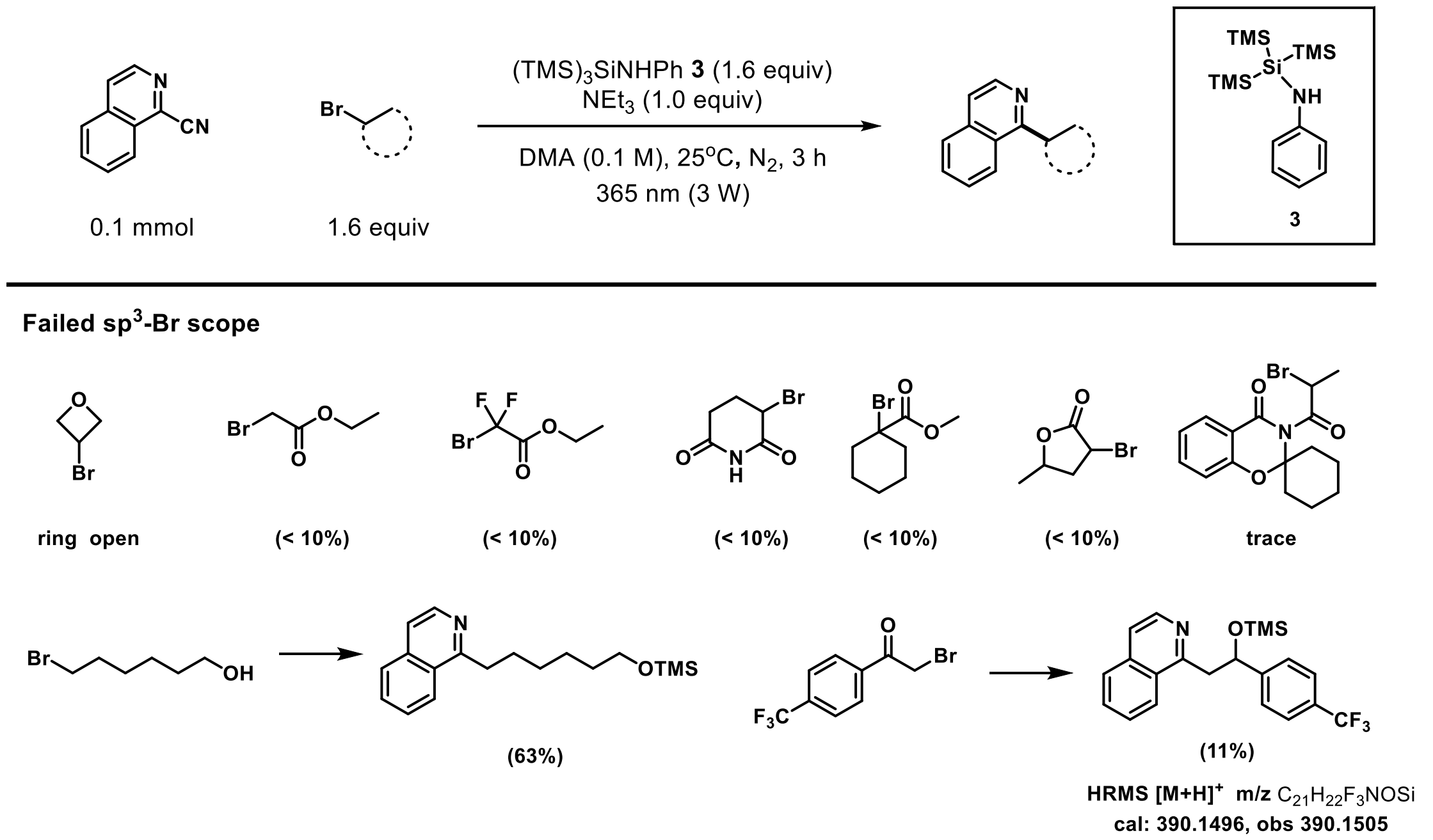

[a]Reaction were conducted on 0.2 mmol scale according to the general procedure. Yield was determined by ^{1}H NMR spectroscopy of the crude reaction mixture using CH_2Br_2 as an internal standard.

Figure S23. Failed sp^2-CN scope[a]

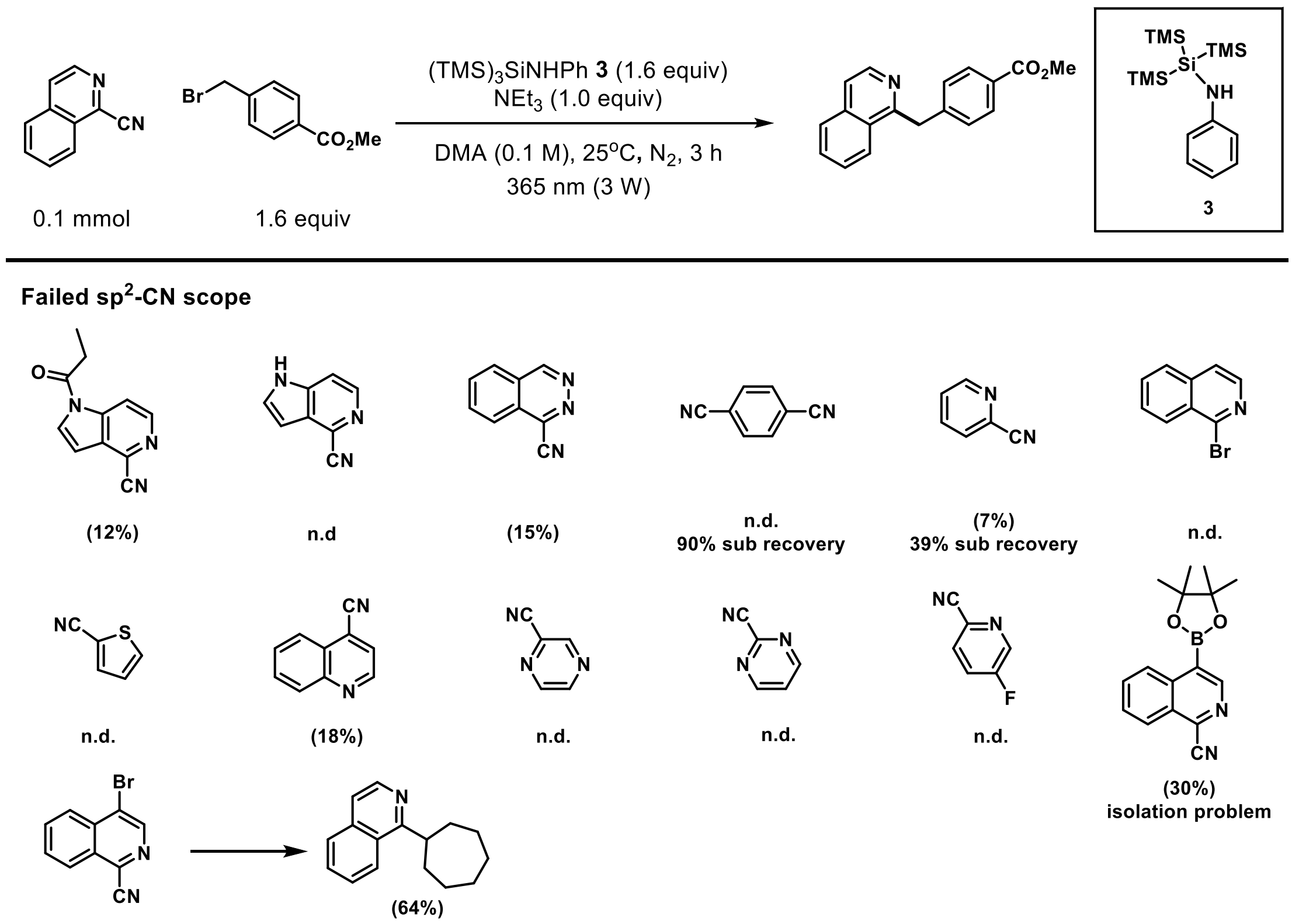

[a]Reaction were conducted on 0.2 mmol scale according to the general procedure. Yield was determined by ^{1}H NMR spectroscopy of the crude reaction mixture using CH_2Br_2 as an internal standard.

5.4 Mass balance analysis

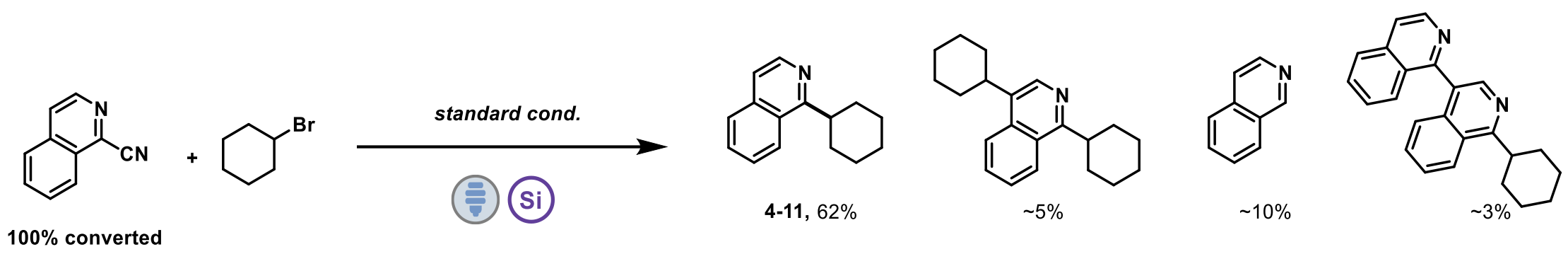

The reaction is conducted under the standard conditions. The yield of byproducts are determined by the integrated area ratio of GCMS analysis. The n-dodecane is used as standard.

The result shows that the starting material is fully consumed (≈100% conversion). The product distribution is as follows: approximately 62% corresponds to the desired cross-coupled product, ~10% corresponds to a reductive hydrogenation byproduct, ~5% corresponds to a dialkylated side product, and ~3% corresponds to a dimeric species arising from coupling with the cyanoquinoline scaffold. Approximately 83% of the starting material can be accounted for by identifiable byproducts, as determined through product distribution and mass balance analysis. The remaining fraction is distributed among a complex mixture of minor species that could not be fully characterized due to their low abundance and structural heterogeneity.

6. NMR Data

^{1}H NMR spectrum of **3** (400 MHz, $CDCl_3$).

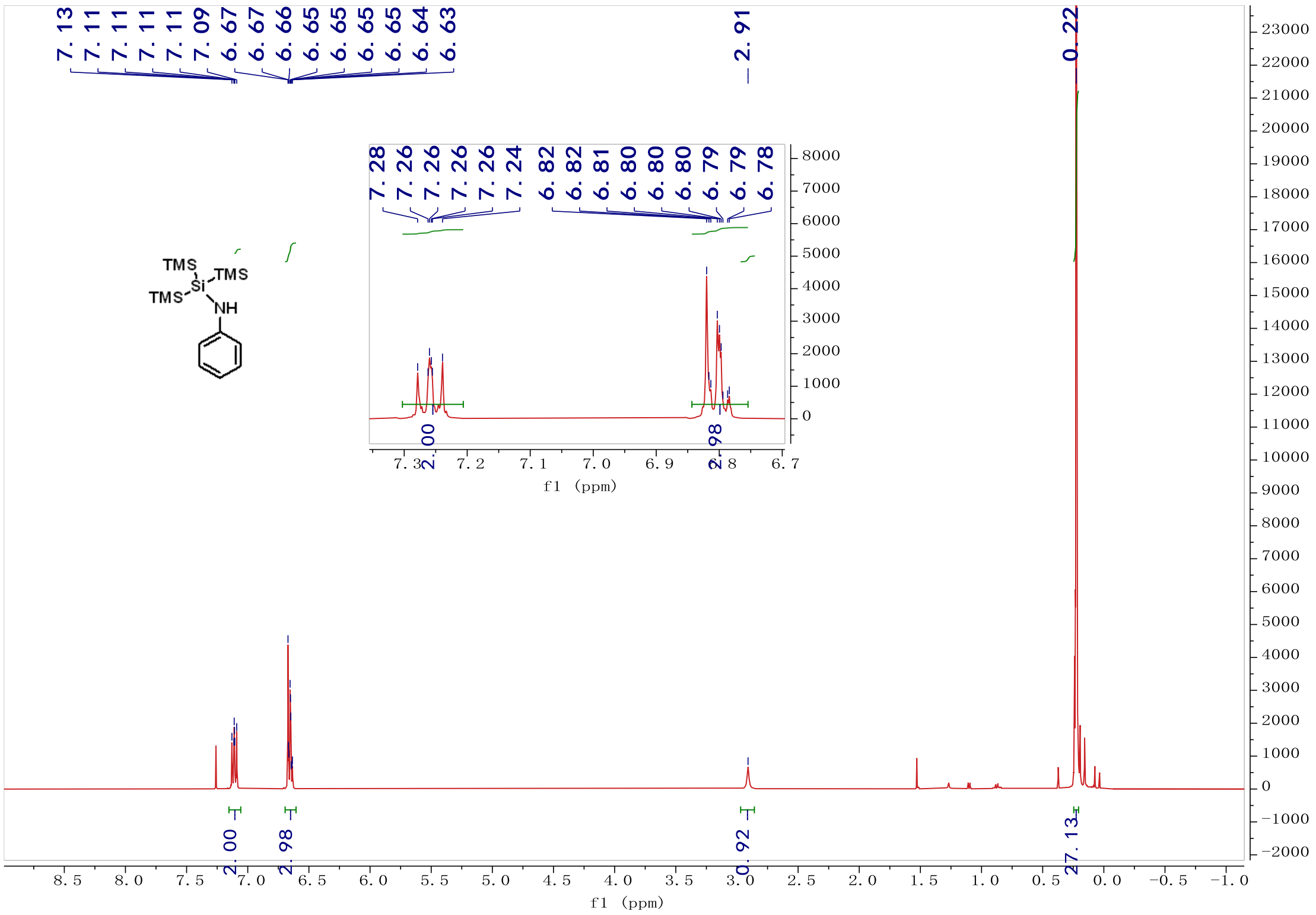

^{13}C{^{1}H} NMR spectrum of **3** (101 MHz, $CDCl_3$).

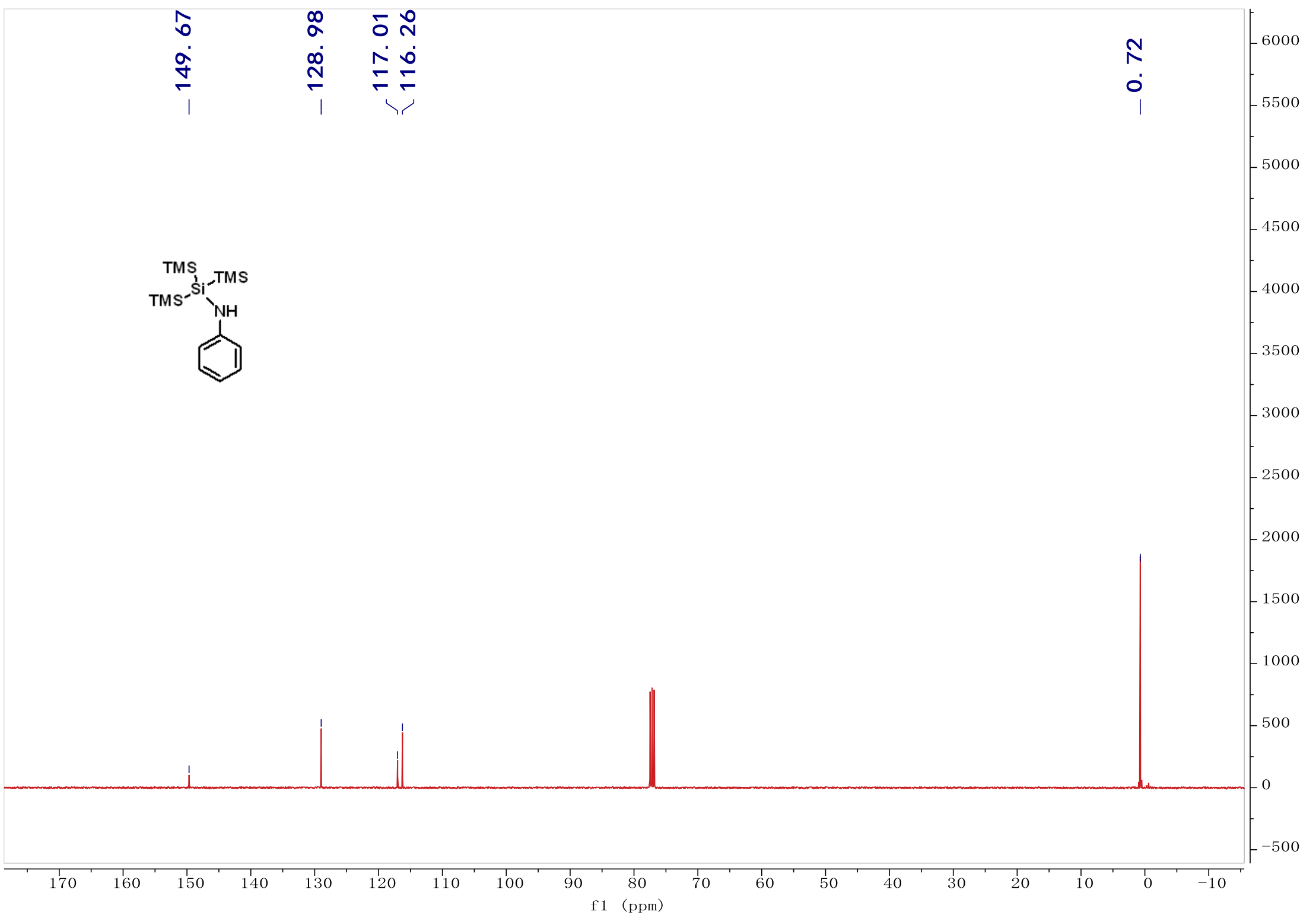

^{1}H NMR spectrum of **S1** (400 MHz, $CDCl_3$).

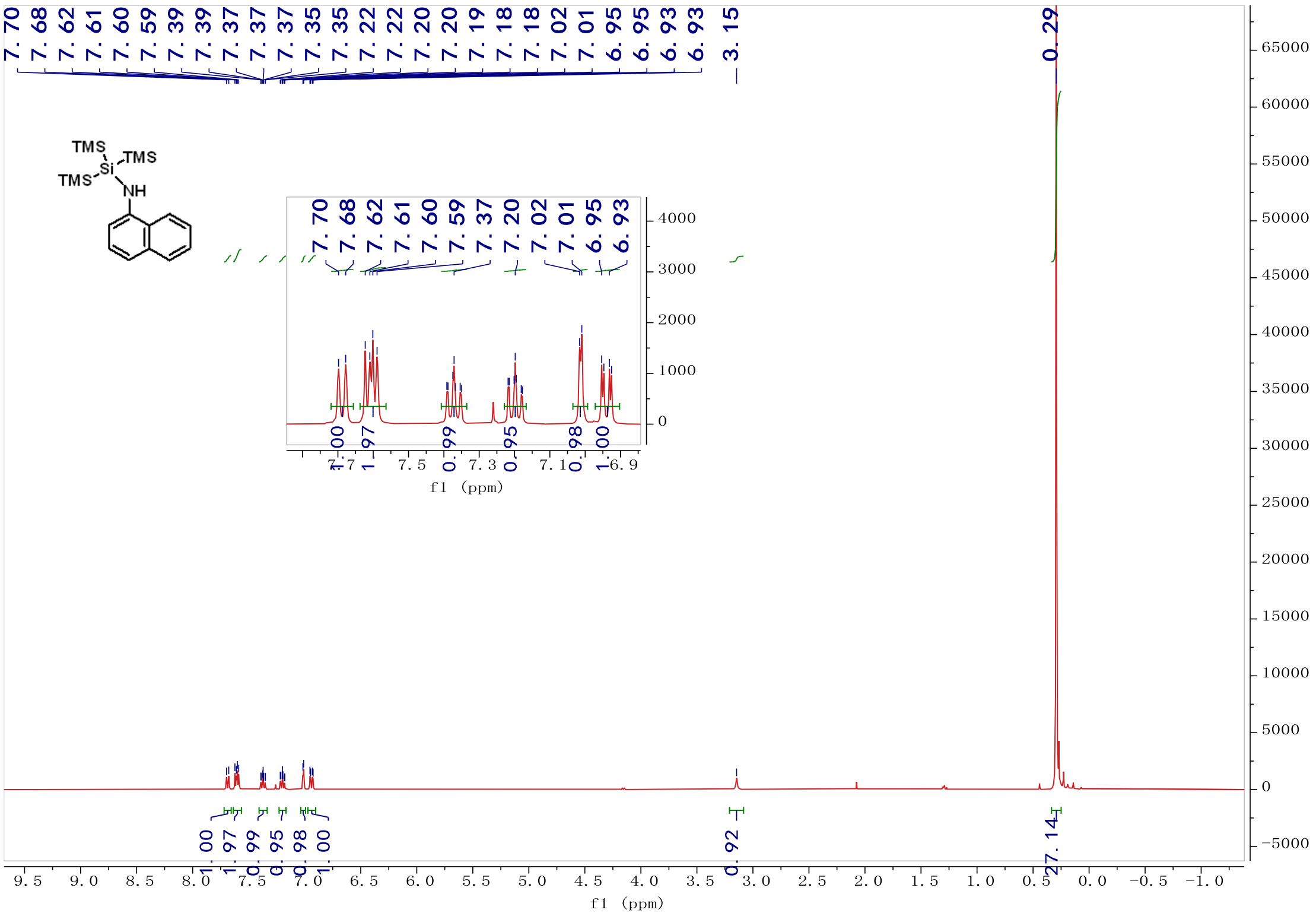

$^{13}C\{^{1}H\}$ NMR spectrum of **S1** (101 MHz, $CDCl_3$).

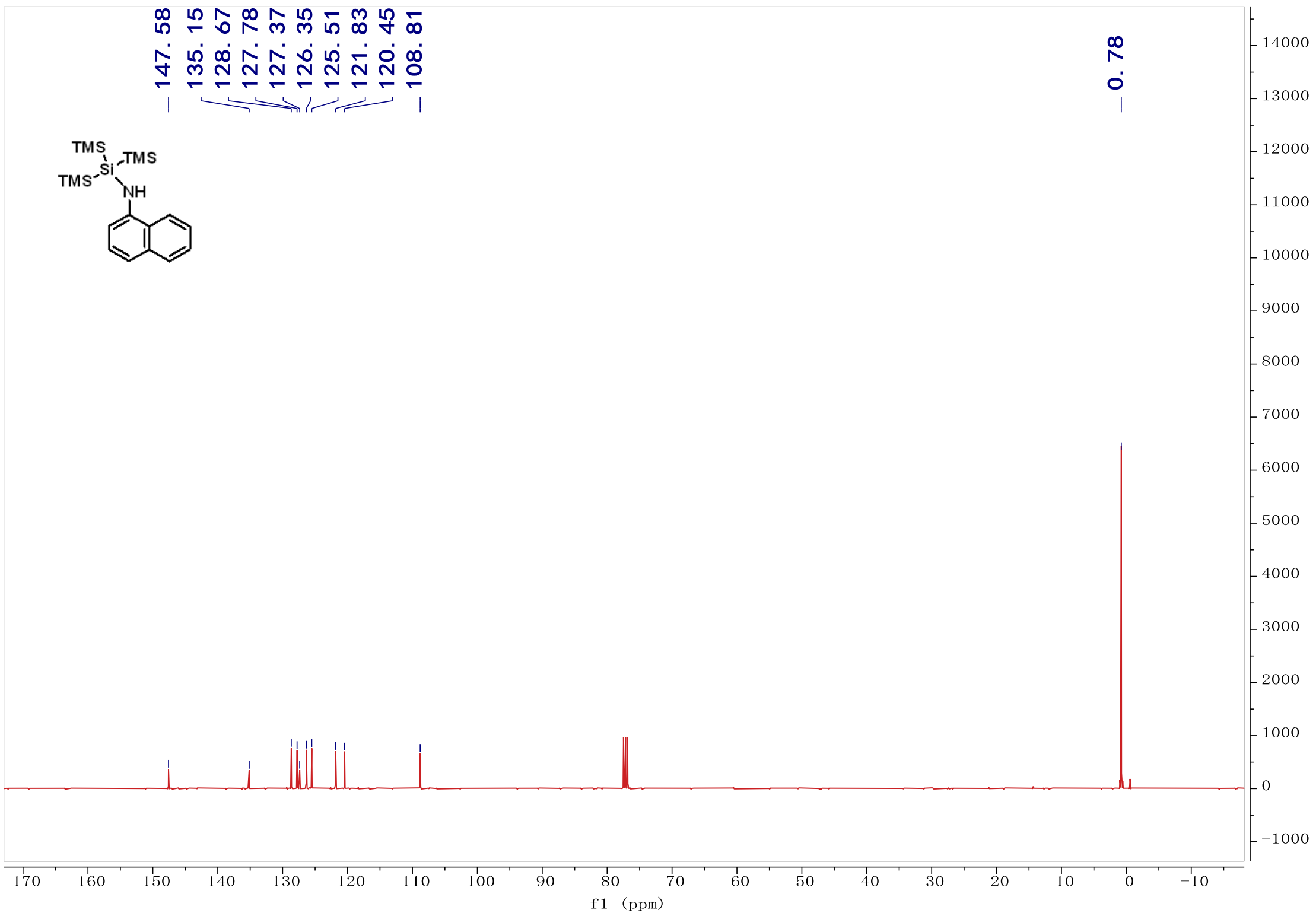

^{1}H NMR spectrum of **S2** (400 MHz, $CDCl_3$).

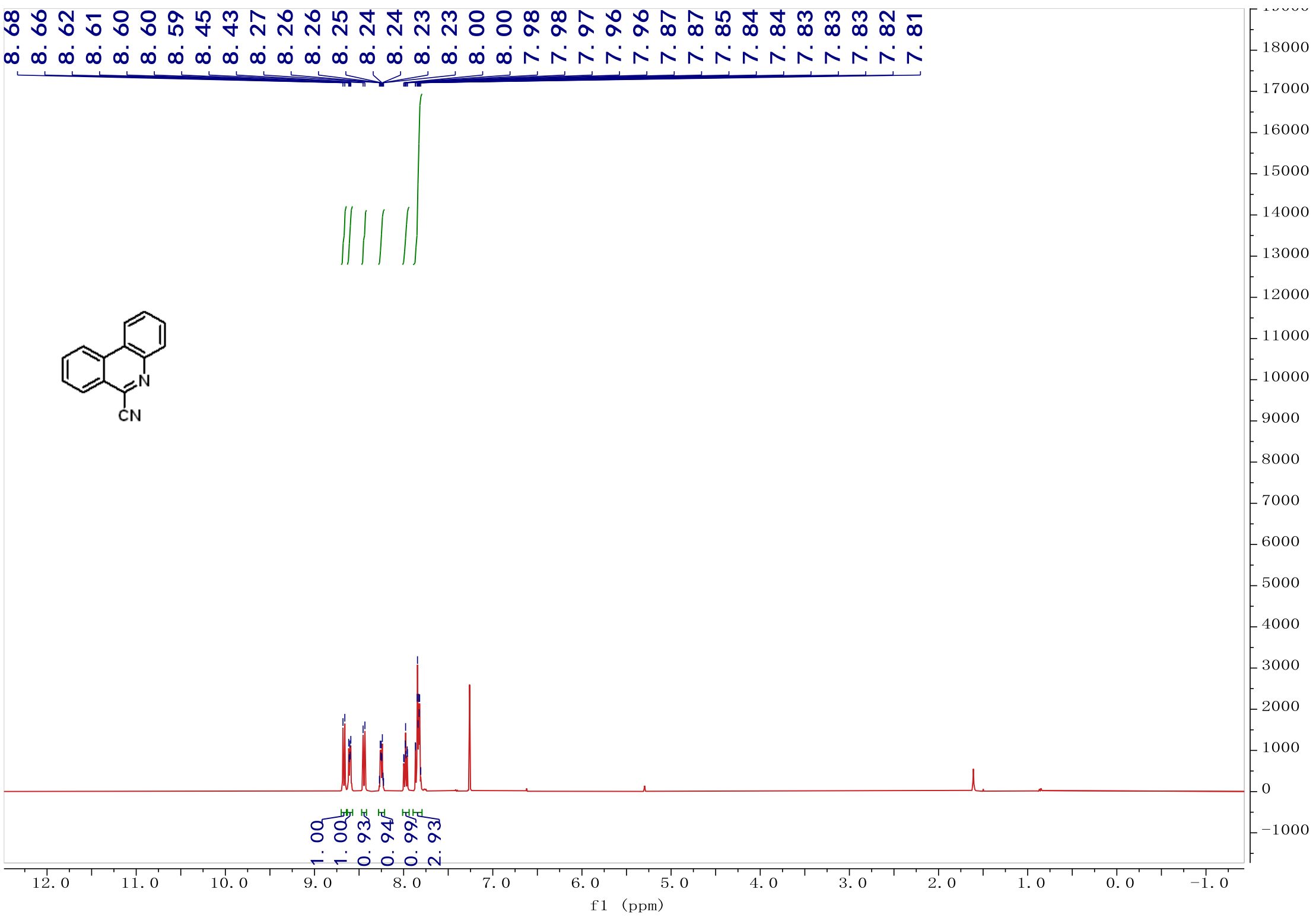

^{13}C{^{1}H} NMR spectrum of **S2** (101 MHz, $CDCl_3$).

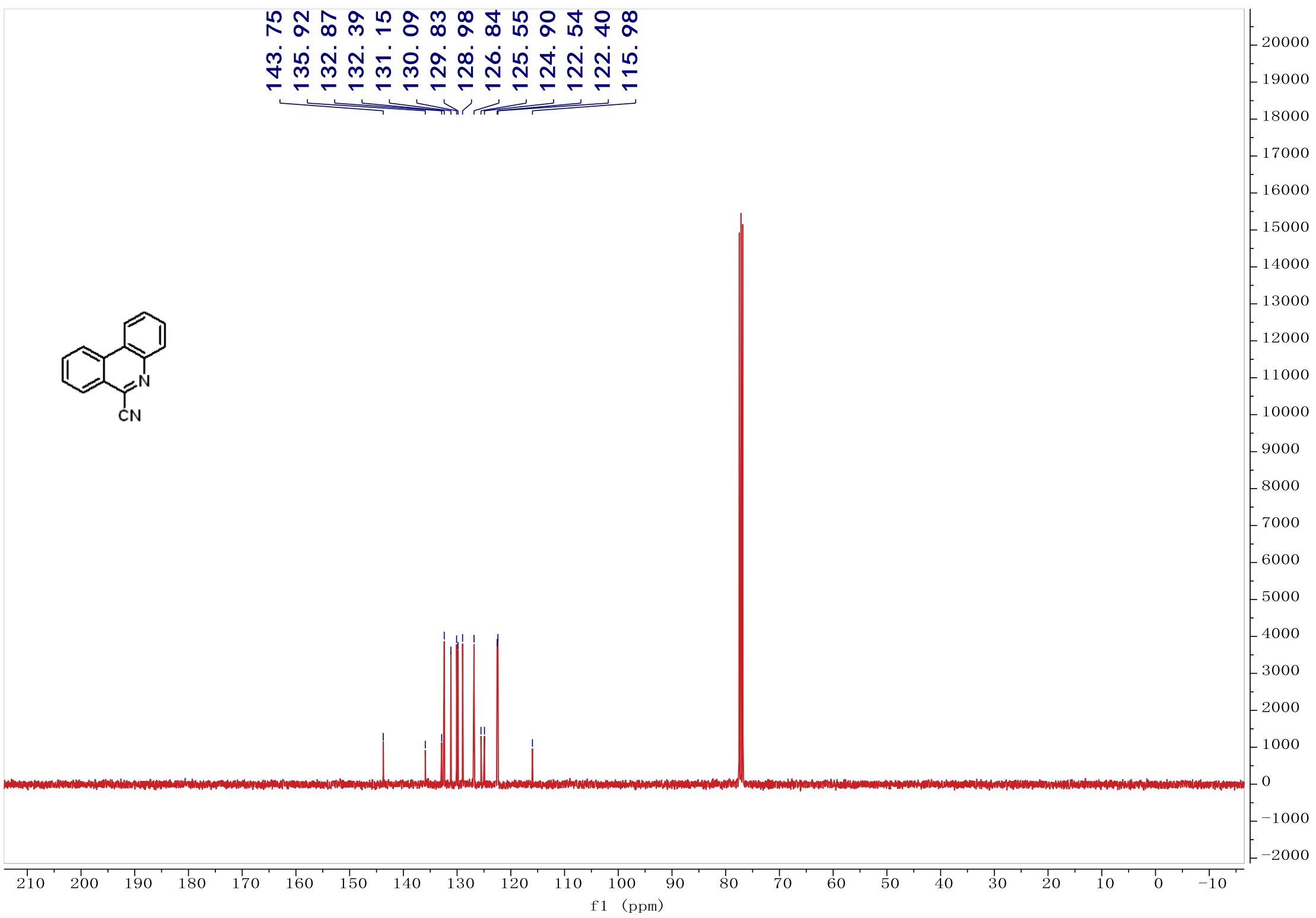

^{1}H NMR spectrum of **S3** (400 MHz, $CDCl_3$).

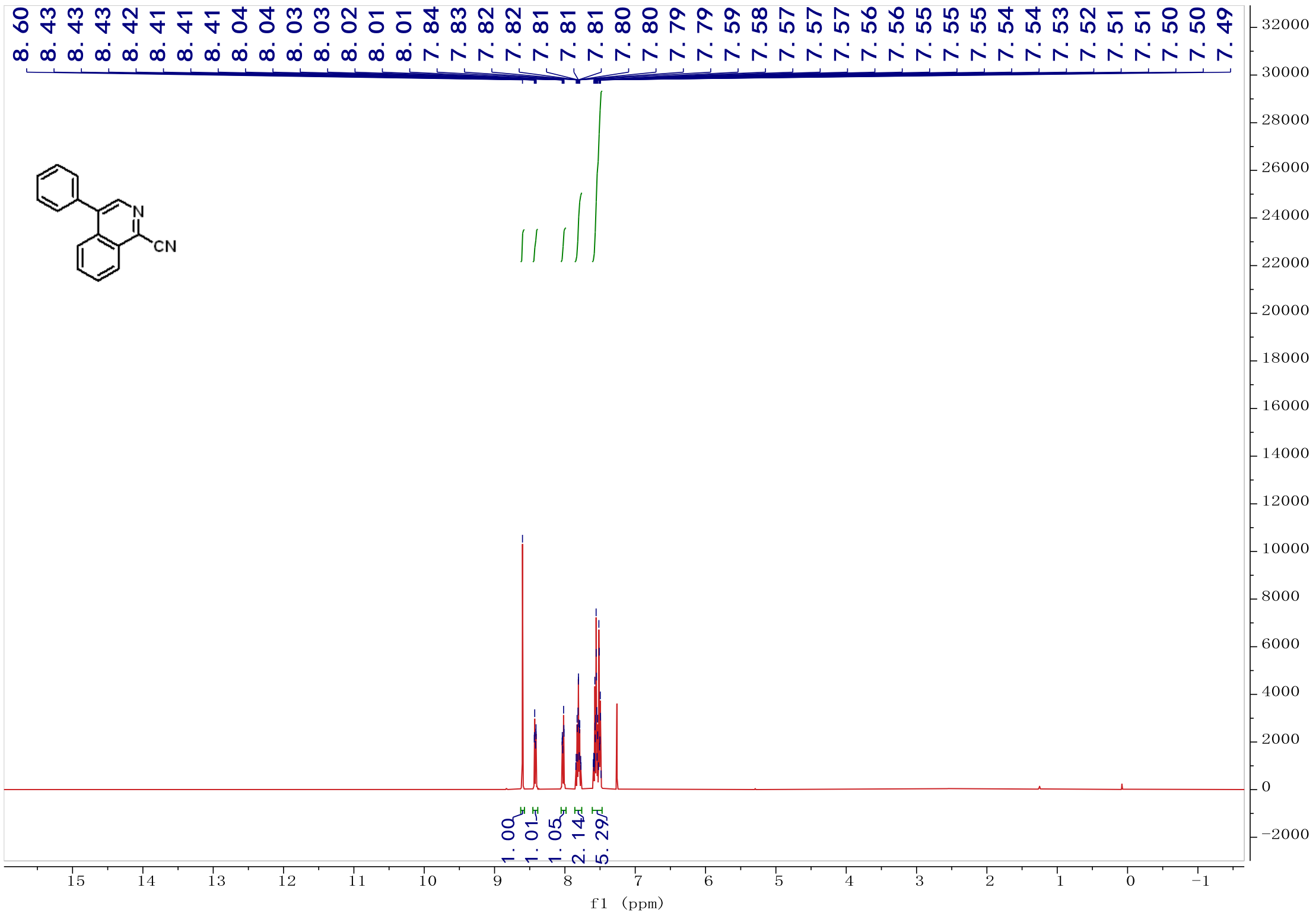

$^{13}C\{^{1}H\}$ NMR spectrum of **S3** (101 MHz, $CDCl_3$).

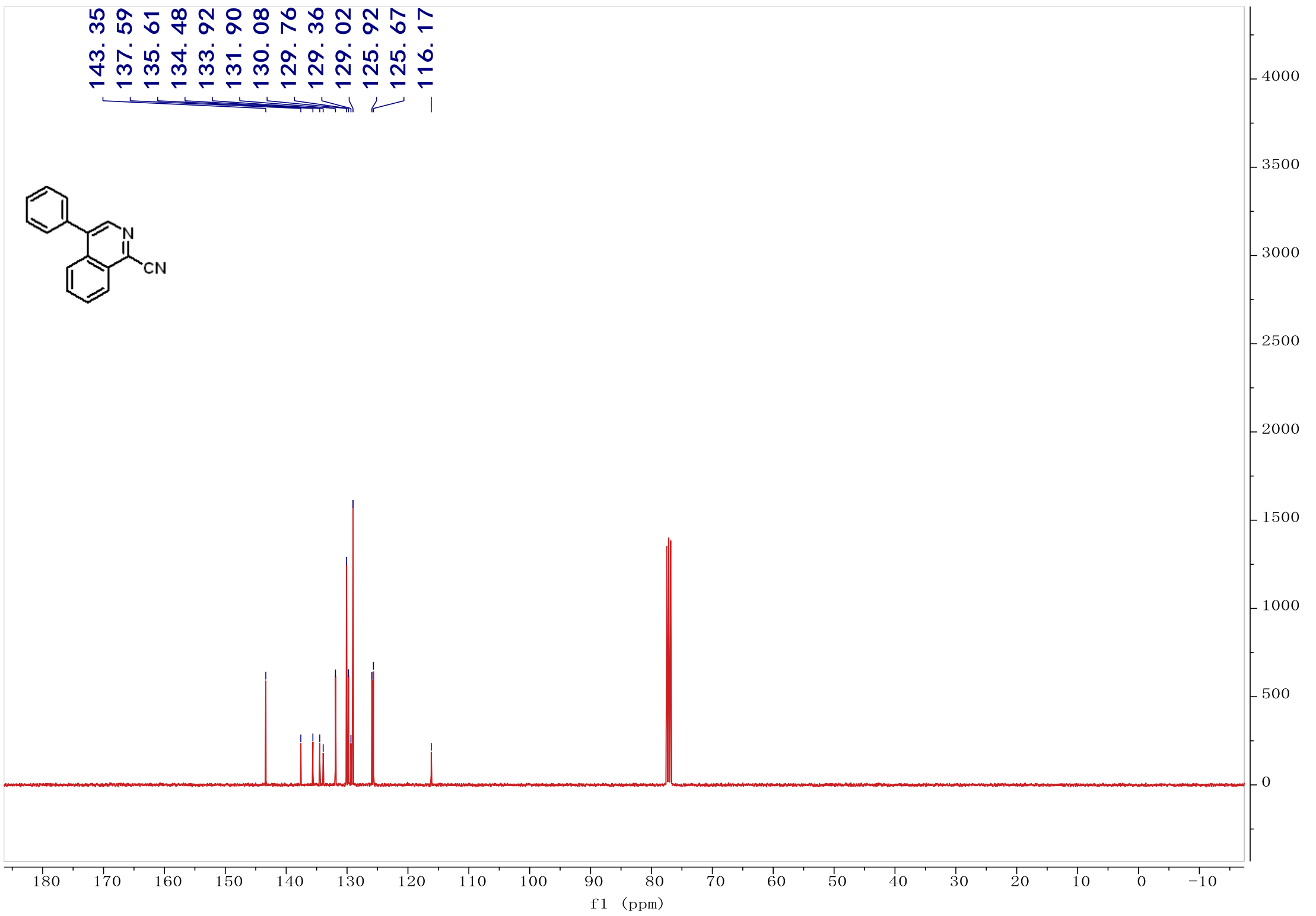

^{1}H NMR spectrum of **S4** (400 MHz, $CDCl_3$).

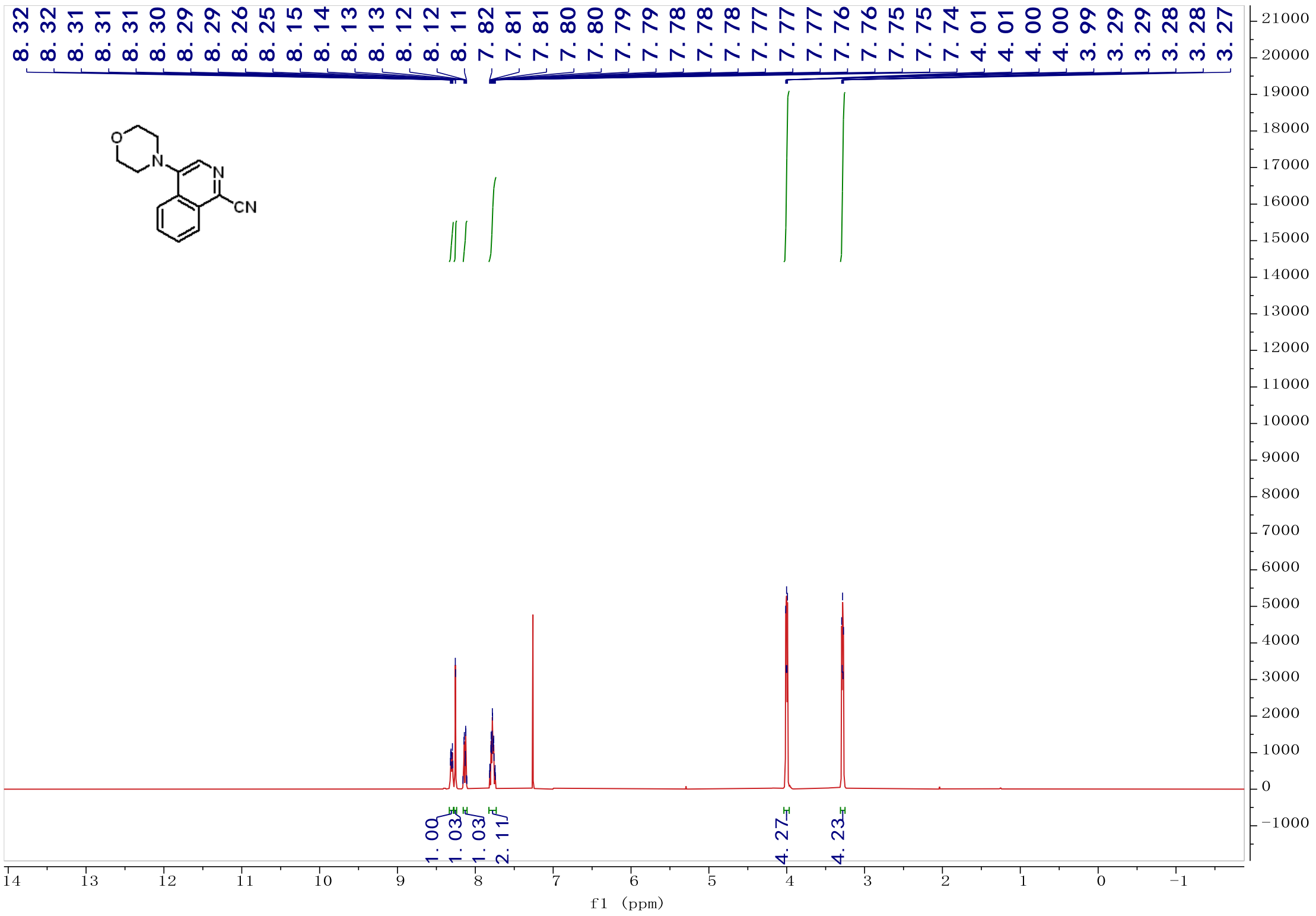

^{13}C{^{1}H} NMR spectrum of **S4** (101 MHz, $CDCl_3$).

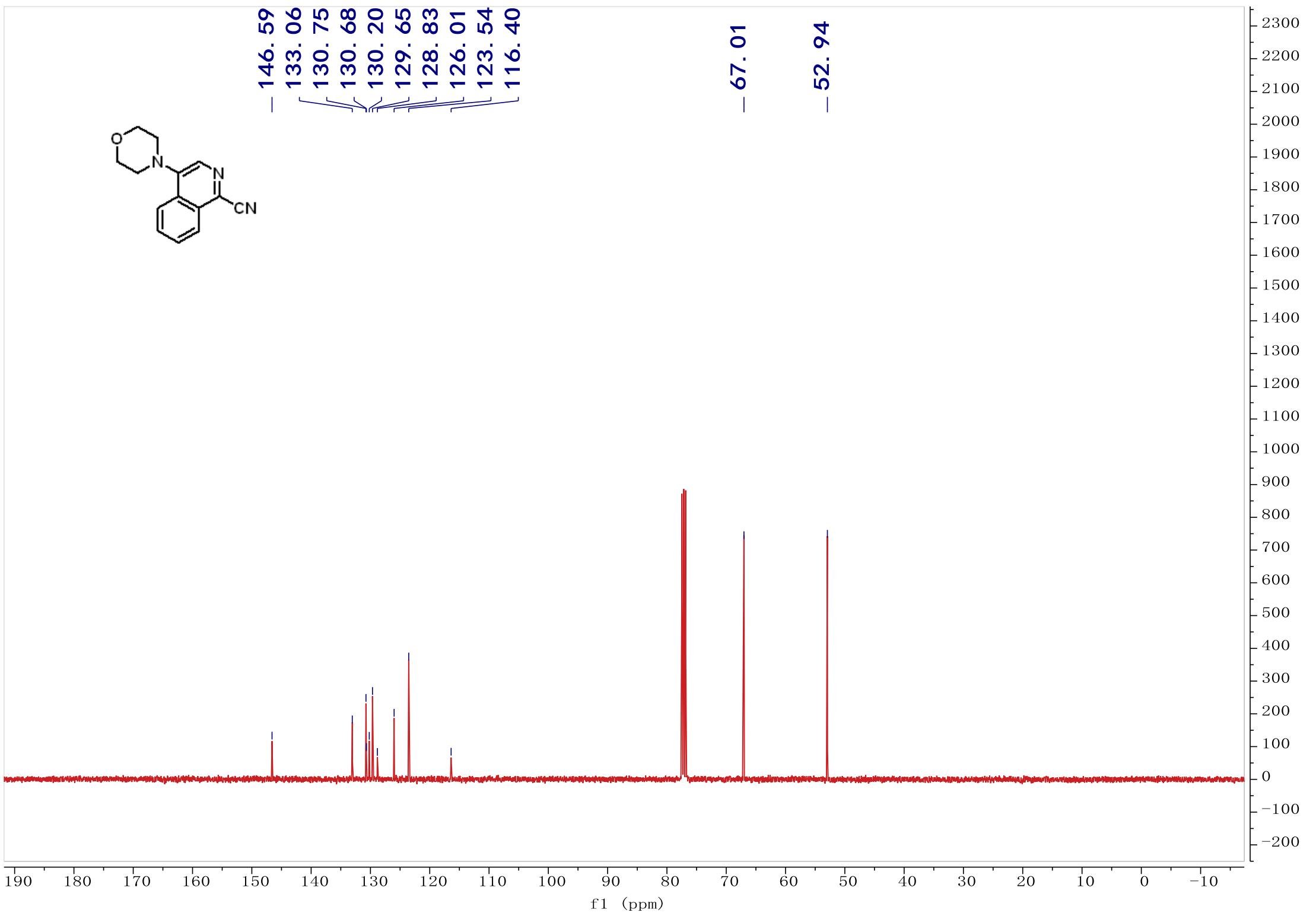

^{1}H NMR spectrum of **S5** (400 MHz, $CDCl_3$).

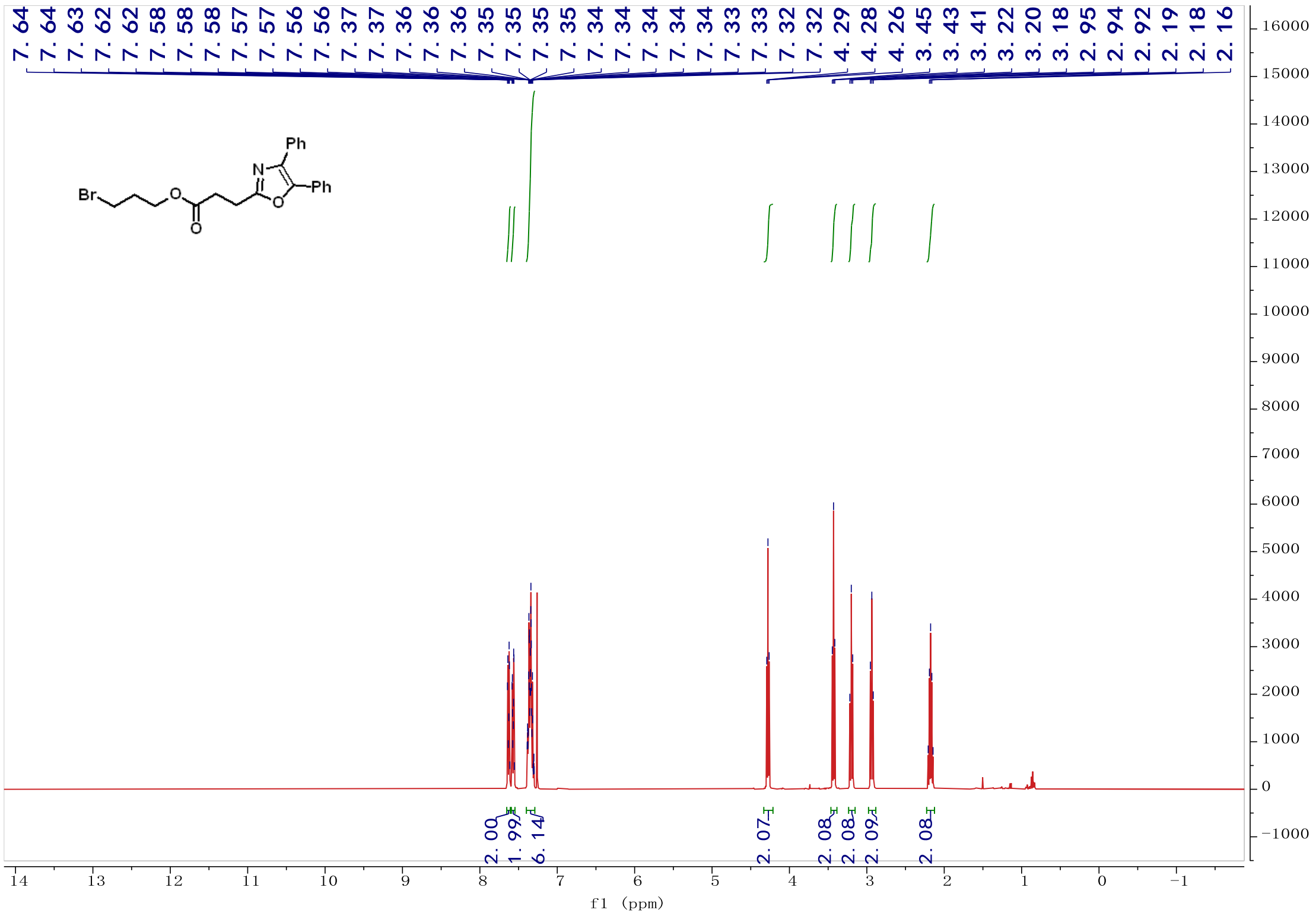

^{13}C{^{1}H} NMR spectrum of **S5** (101 MHz, $CDCl_3$).

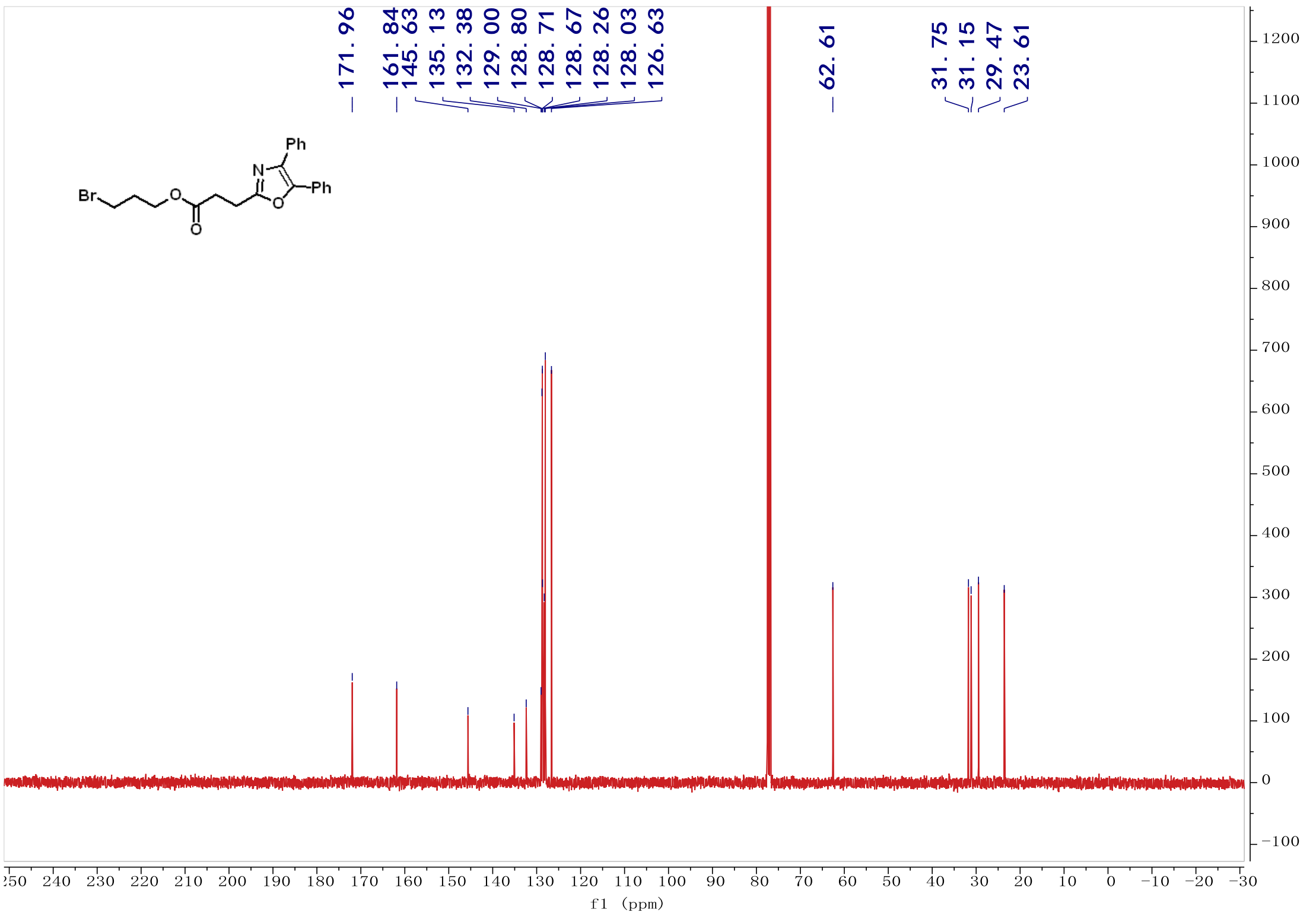

^{1}H NMR spectrum of **S6** (400 MHz, $CDCl_3$).

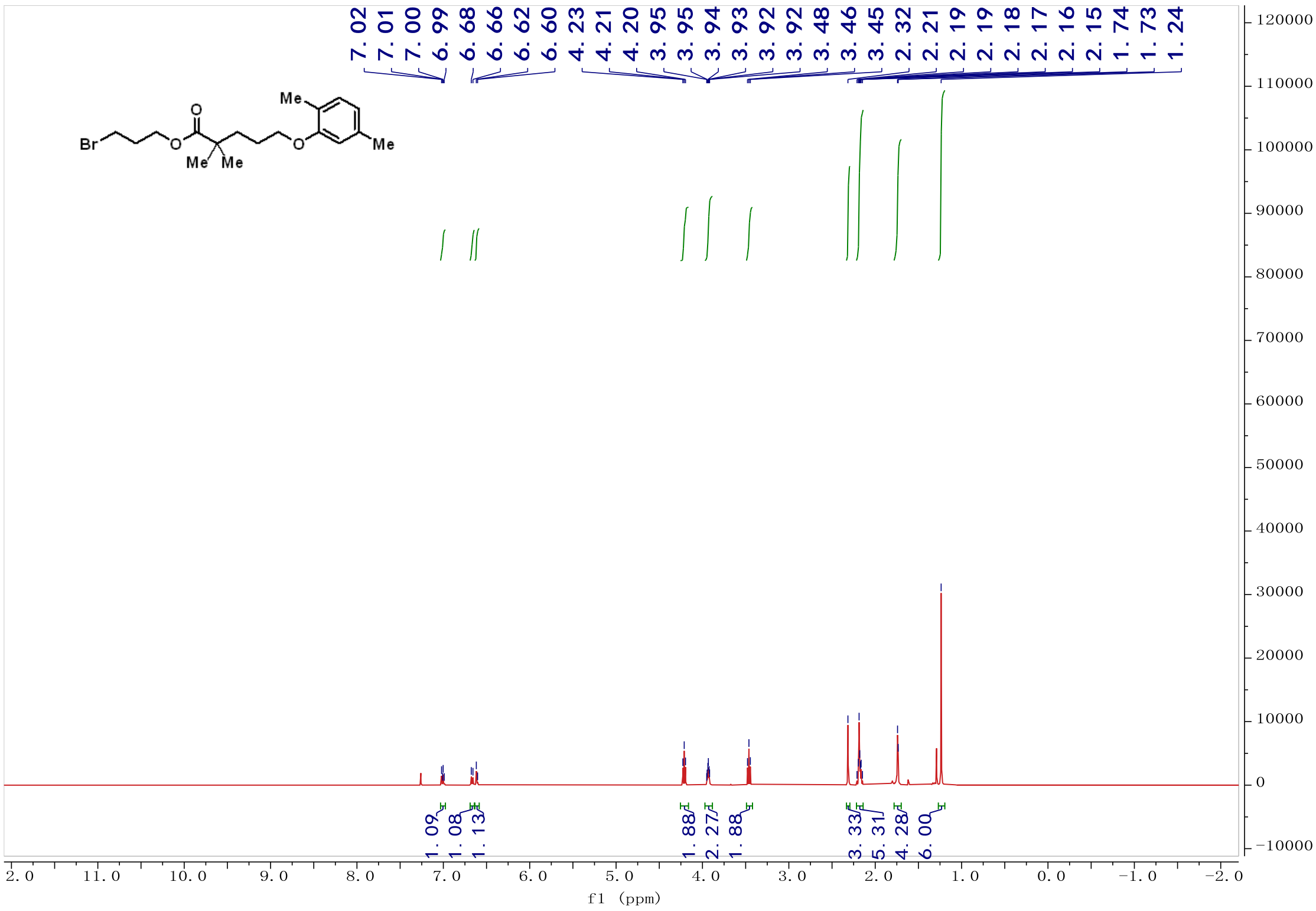

^{13}C{^{1}H} NMR spectrum of **S6** (101 MHz, $CDCl_3$).

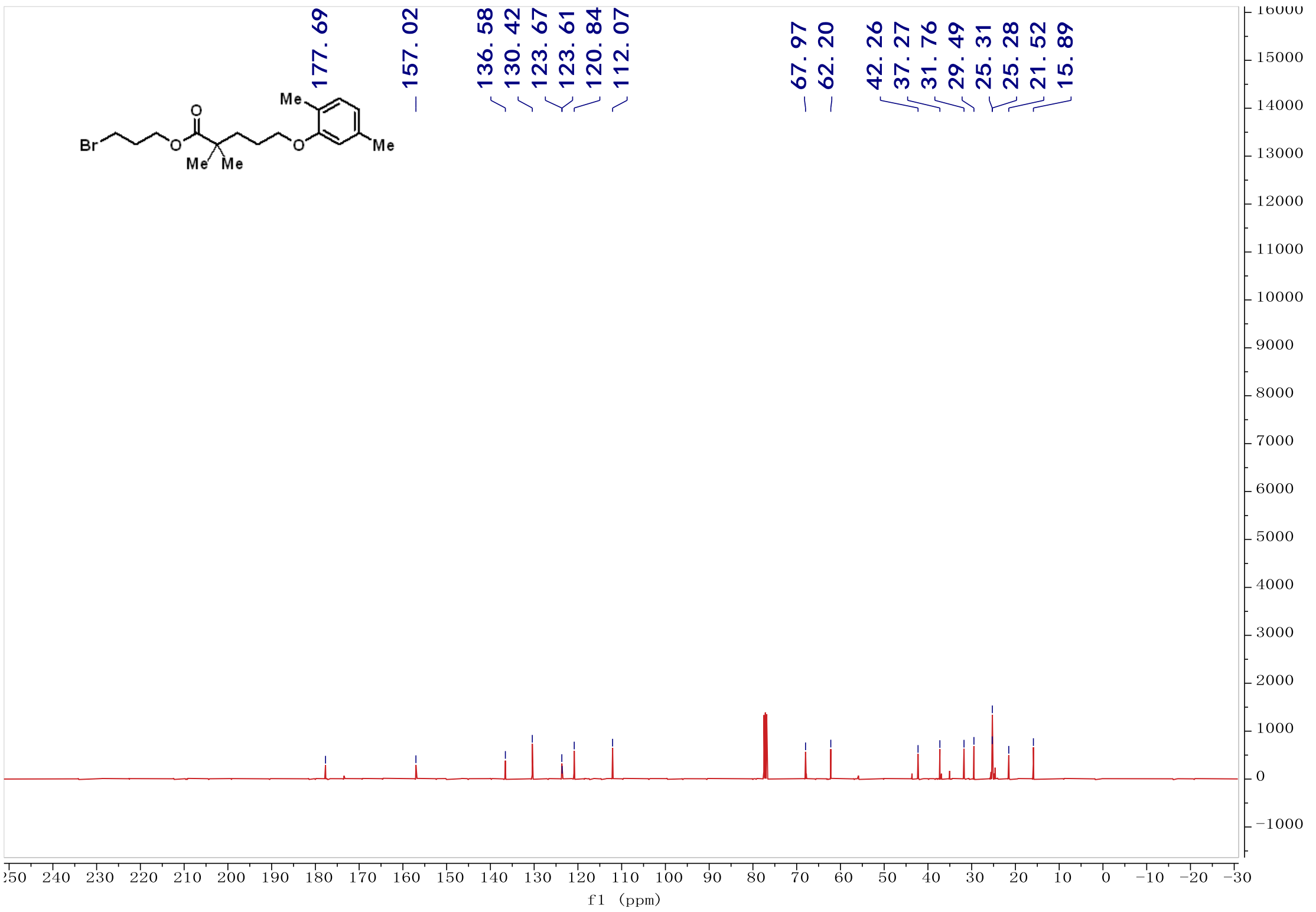

^{1}H NMR spectrum of **S7** (400 MHz, $CDCl_3$).

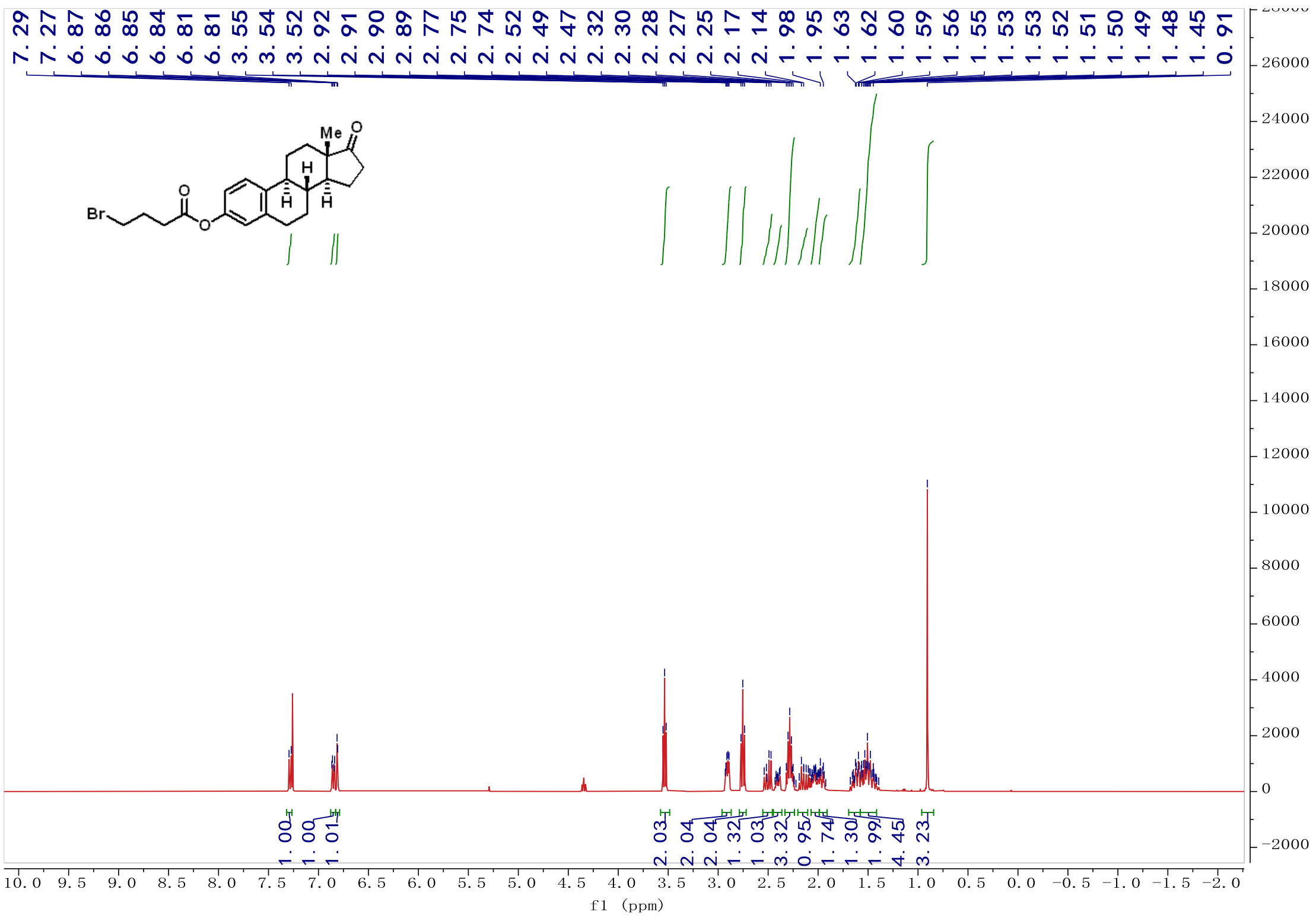

^{13}C{^{1}H} NMR spectrum of **S7** (101 MHz, $CDCl_3$).

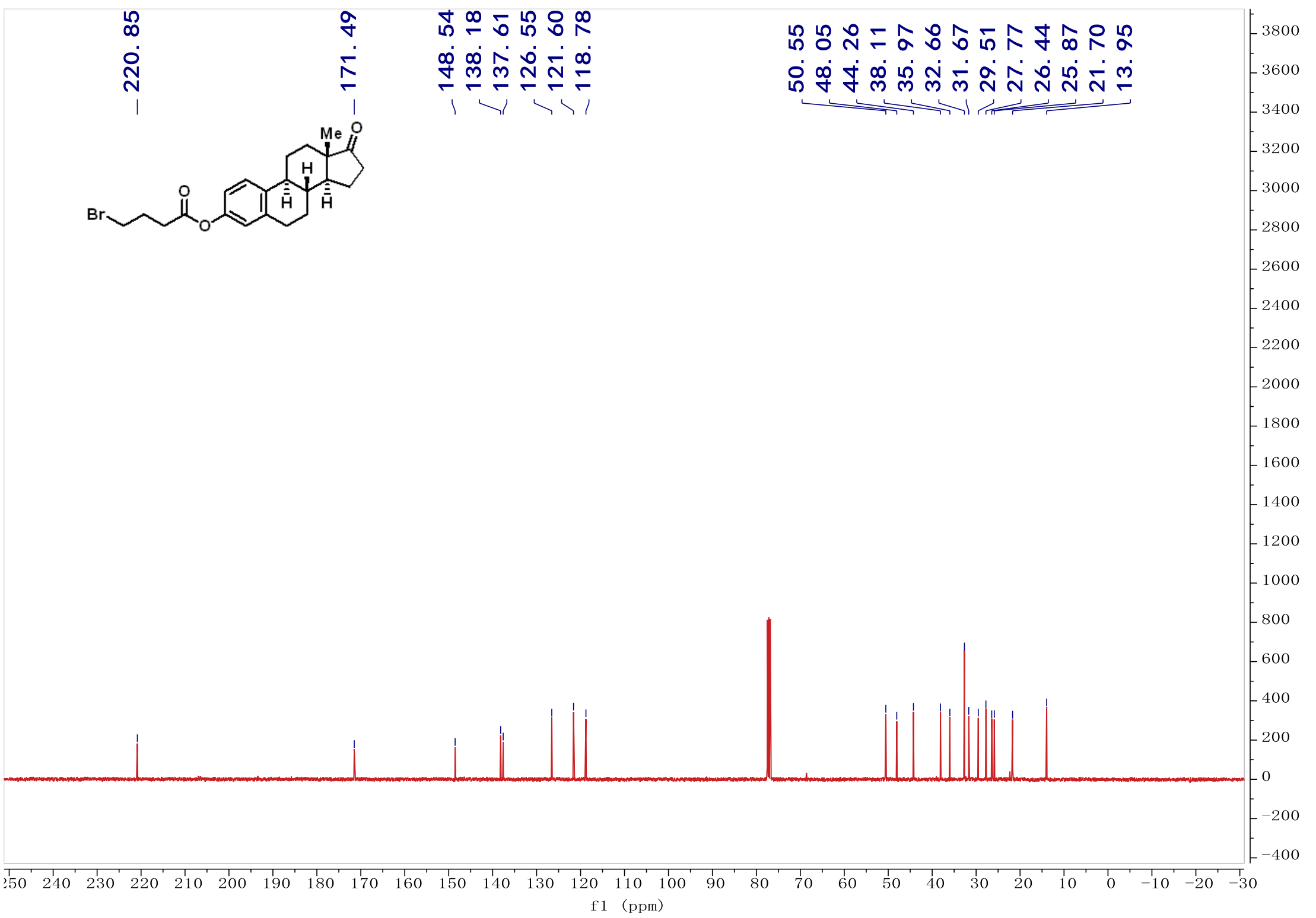

^{1}H NMR spectrum of **S8** (400 MHz, $CDCl_3$).

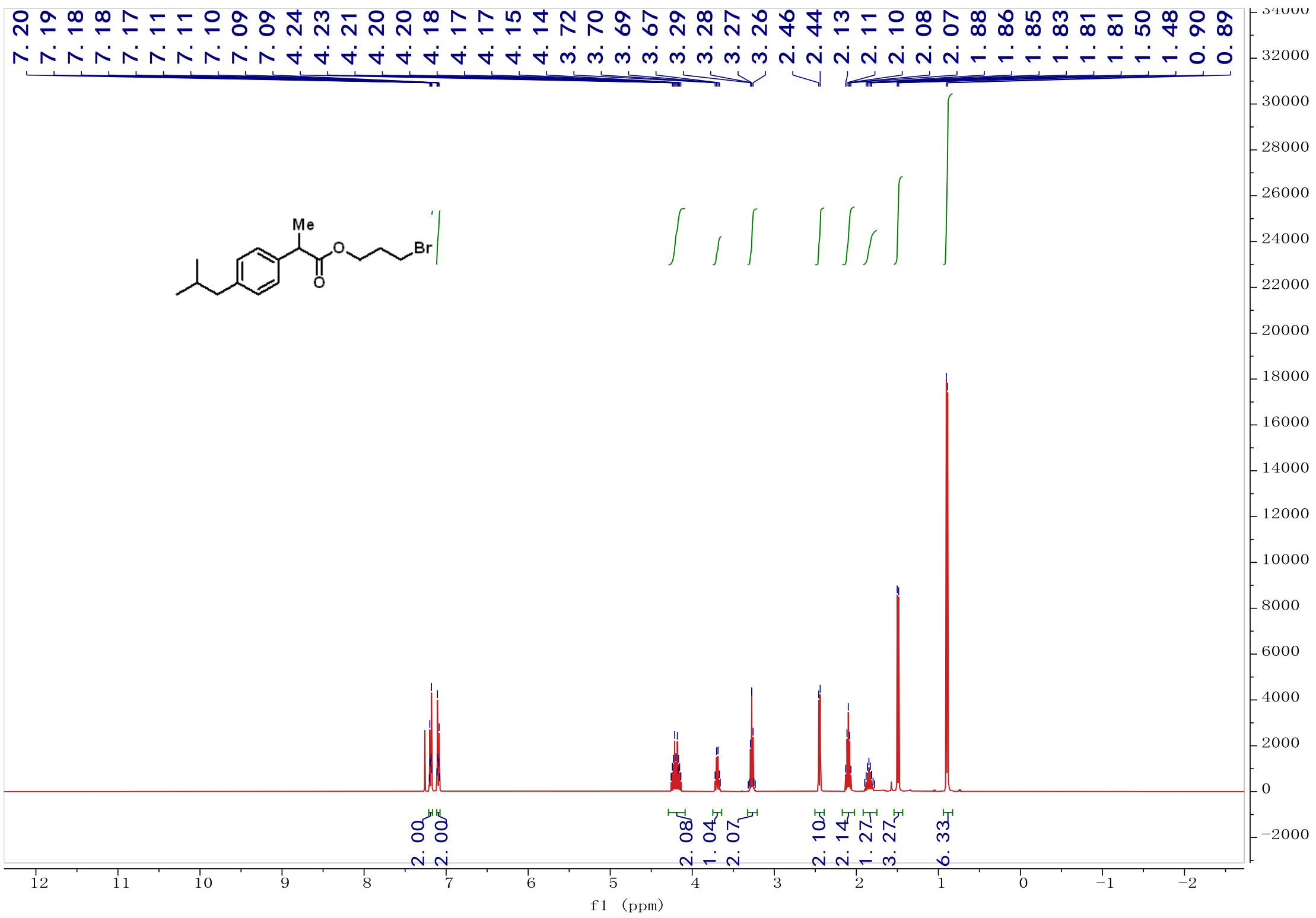

^{13}C{^{1}H} NMR spectrum of **S7** (101 MHz, $CDCl_3$).

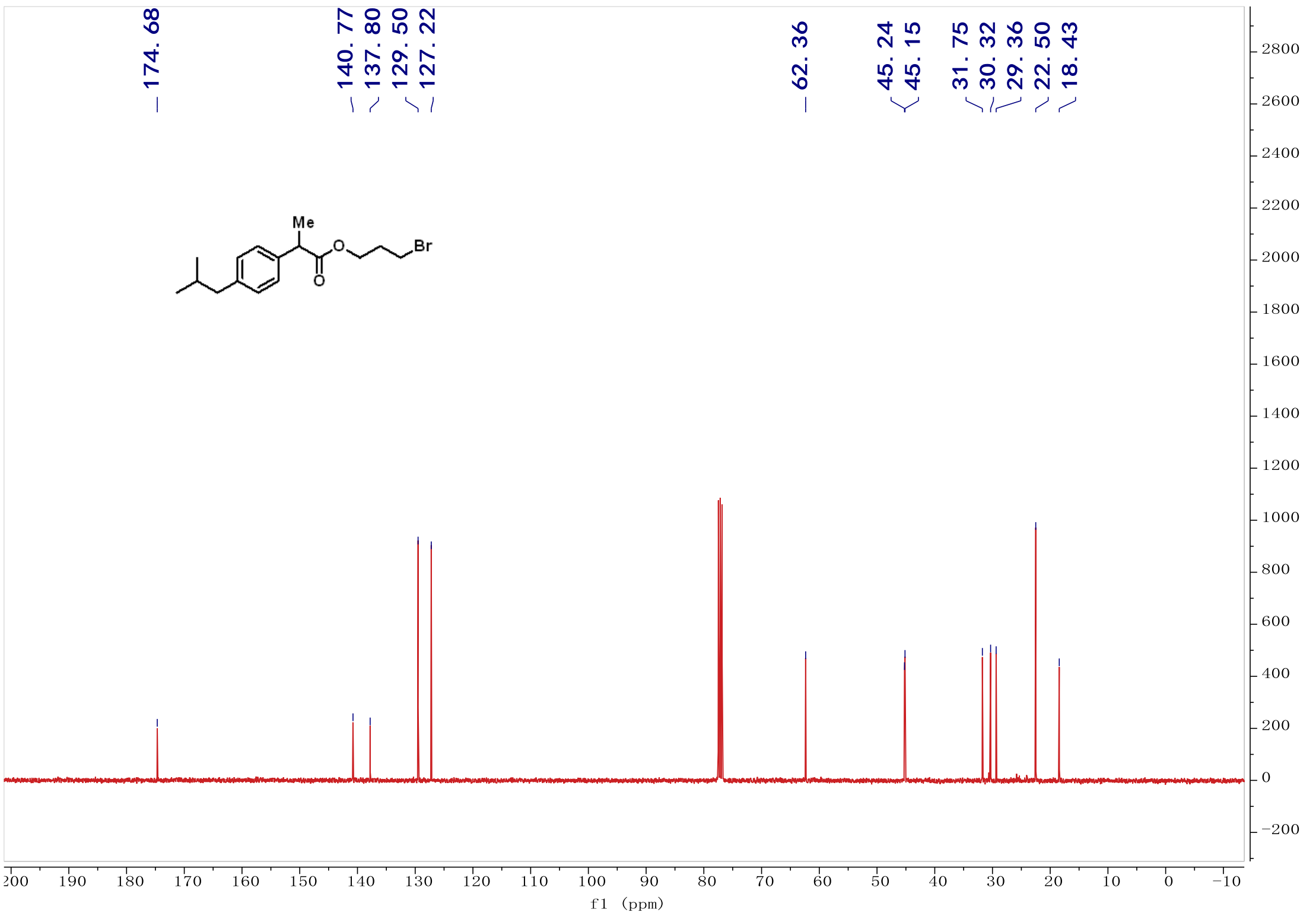

^{1}H NMR spectrum of **S9** (400 MHz, $CDCl_3$).

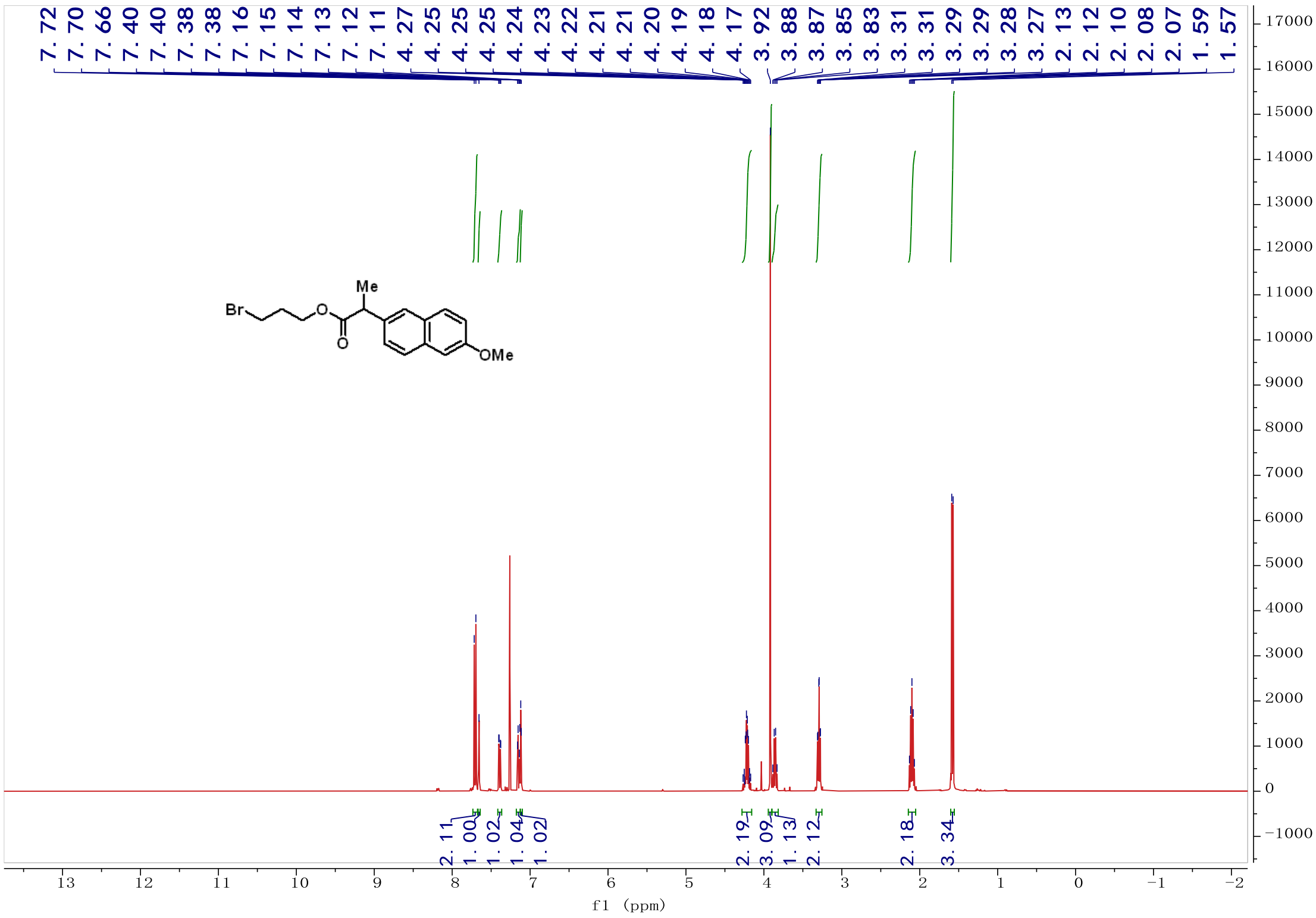

$^{13}C\{^1H\}$ NMR spectrum of **S9** (101 MHz, $CDCl_3$).

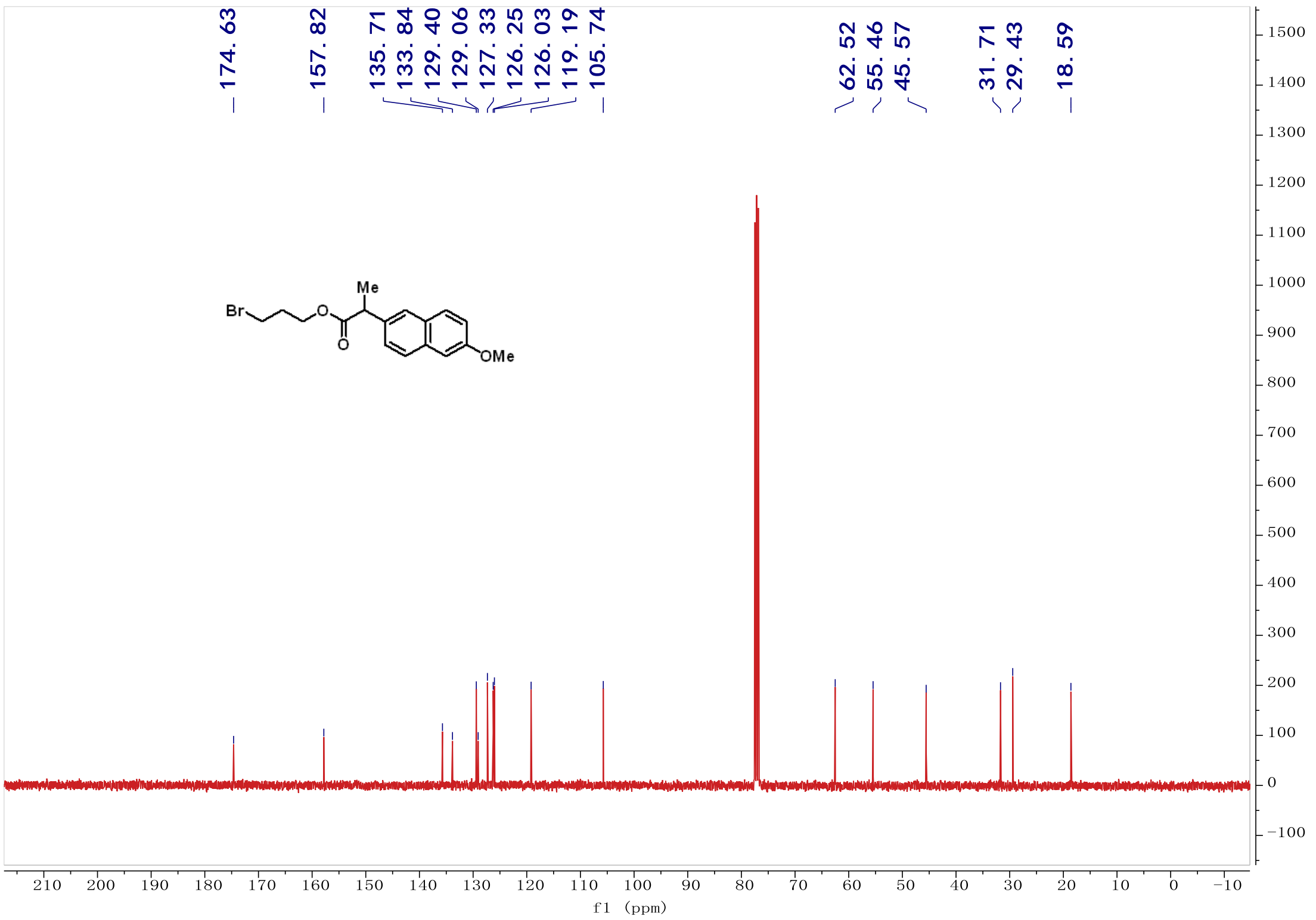

^{1}H NMR spectrum of **S10** (400 MHz, $CDCl_3$).

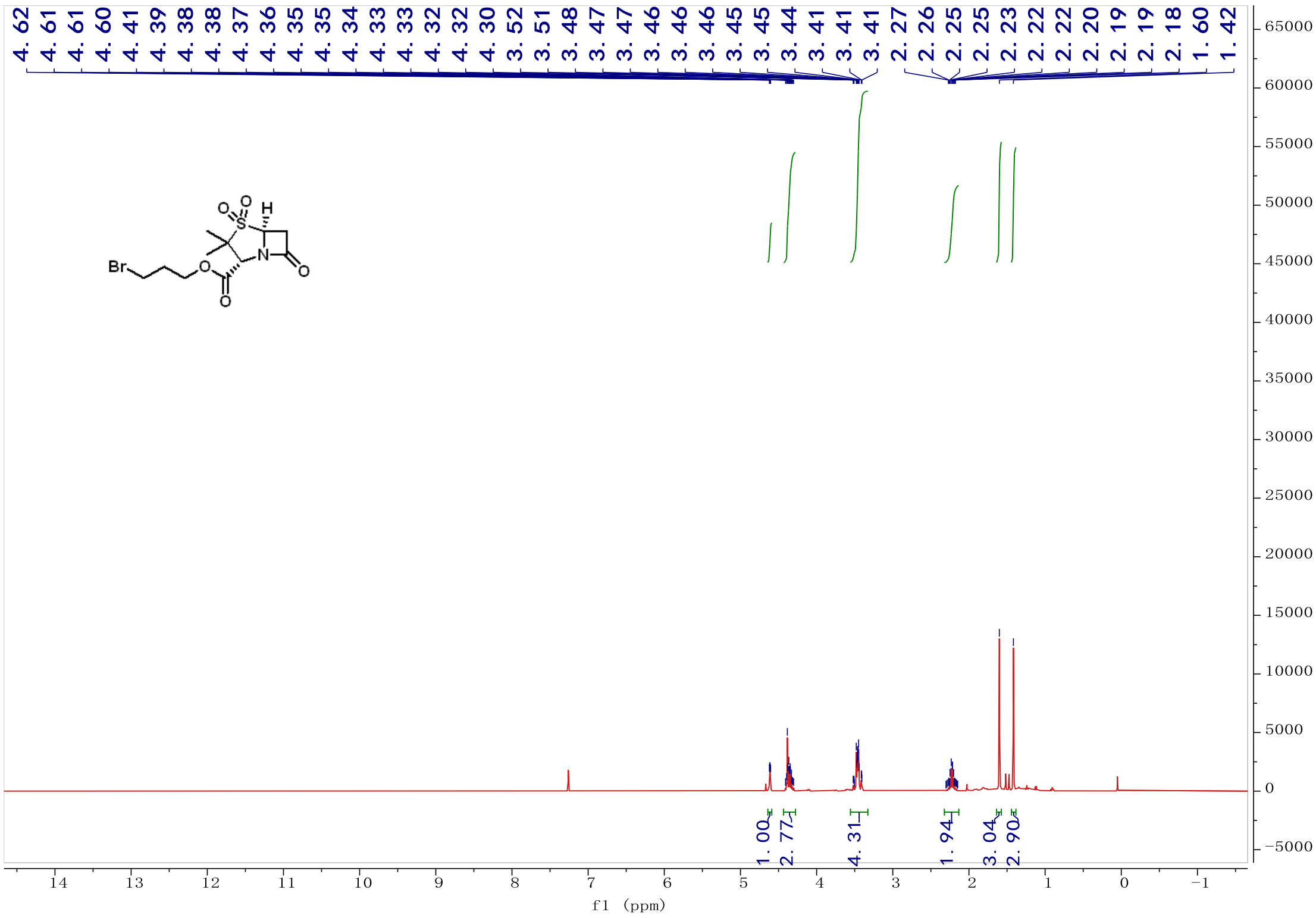

$^{13}C\{^{1}H\}$ NMR spectrum of **S10** (101 MHz, $CDCl_3$).

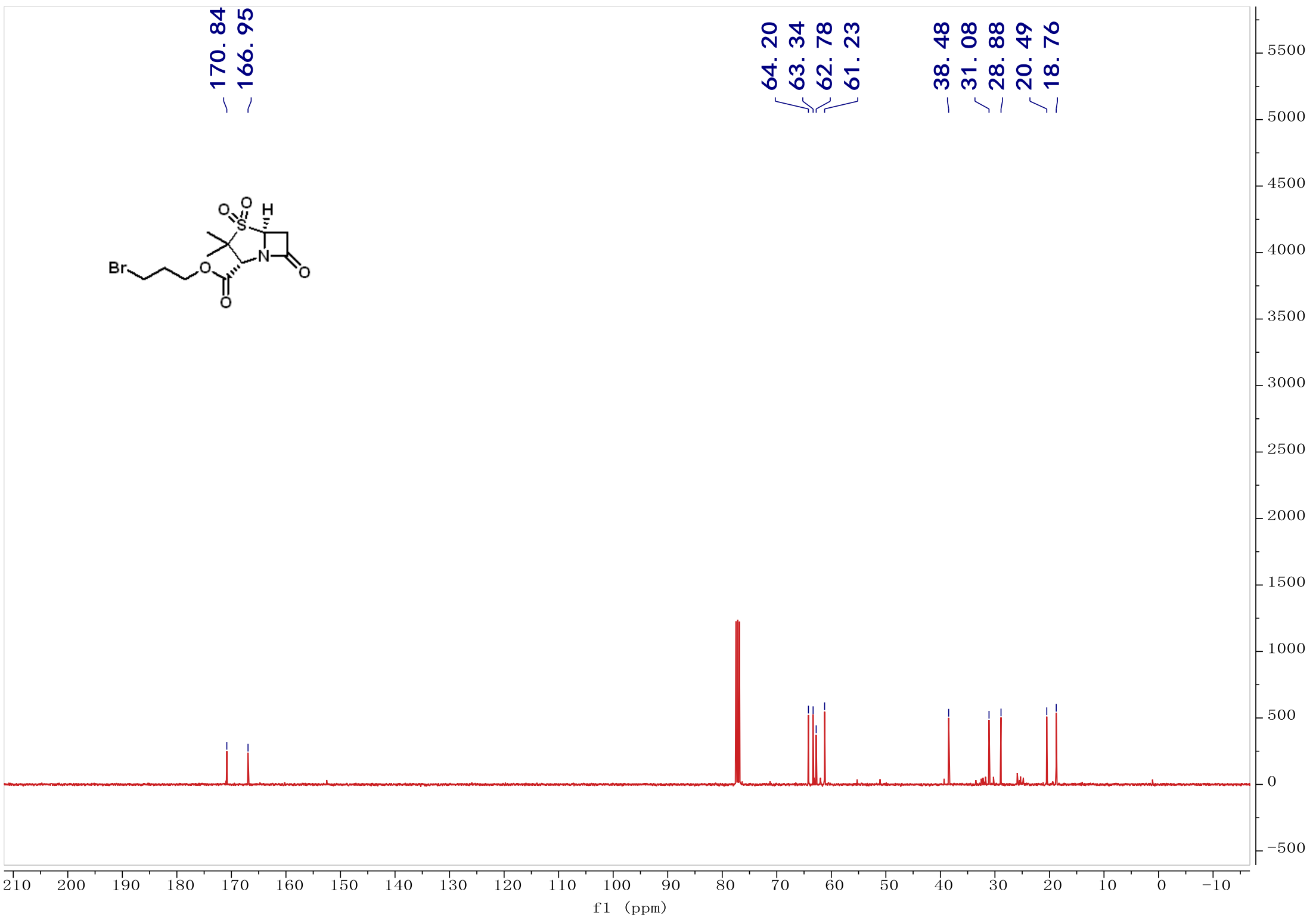

^{1}H NMR spectrum of **S11** (400 MHz, $CDCl_3$).

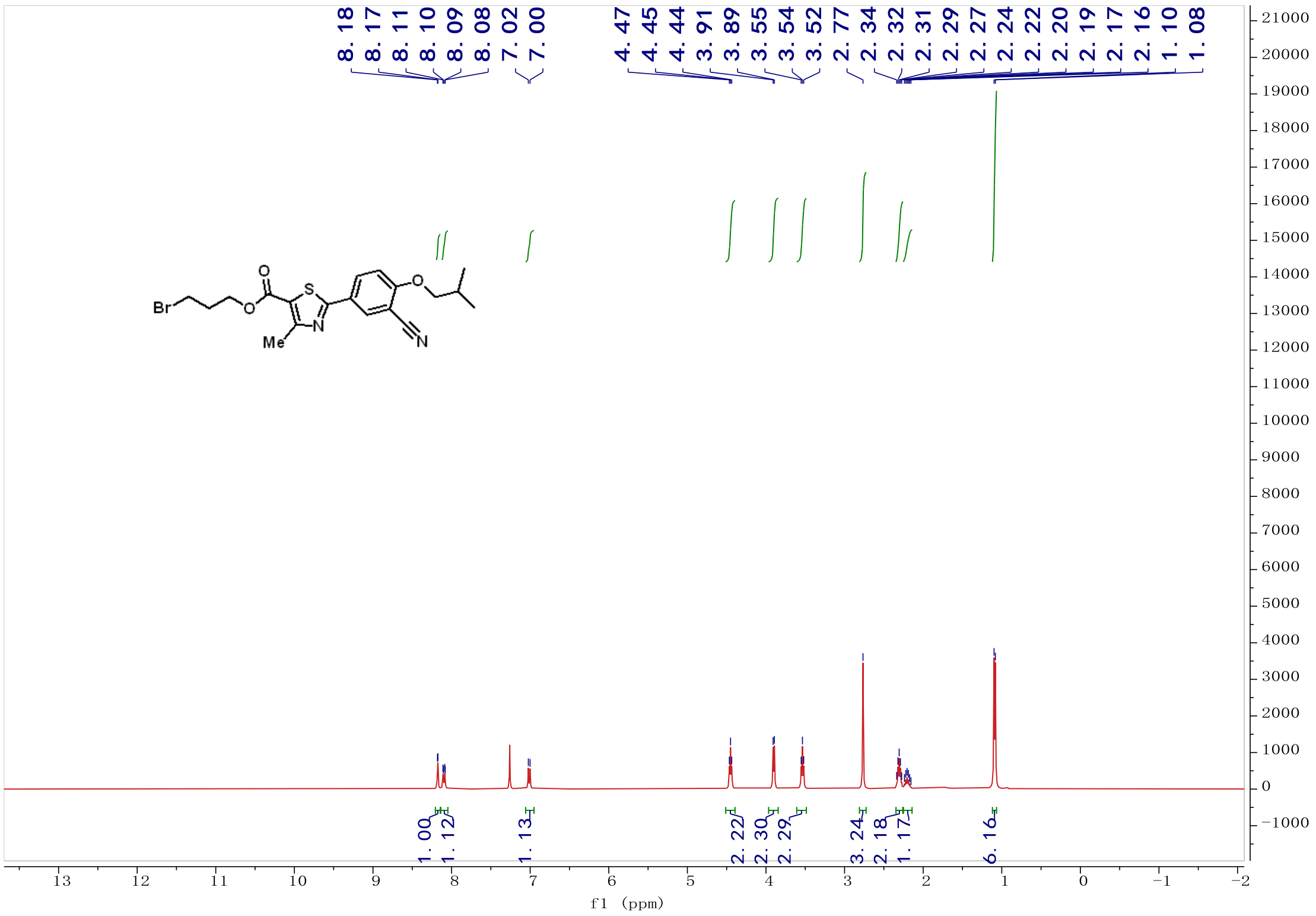

$^{13}C\{^{1}H\}$ NMR spectrum of **S11** (101 MHz, $CDCl_3$).

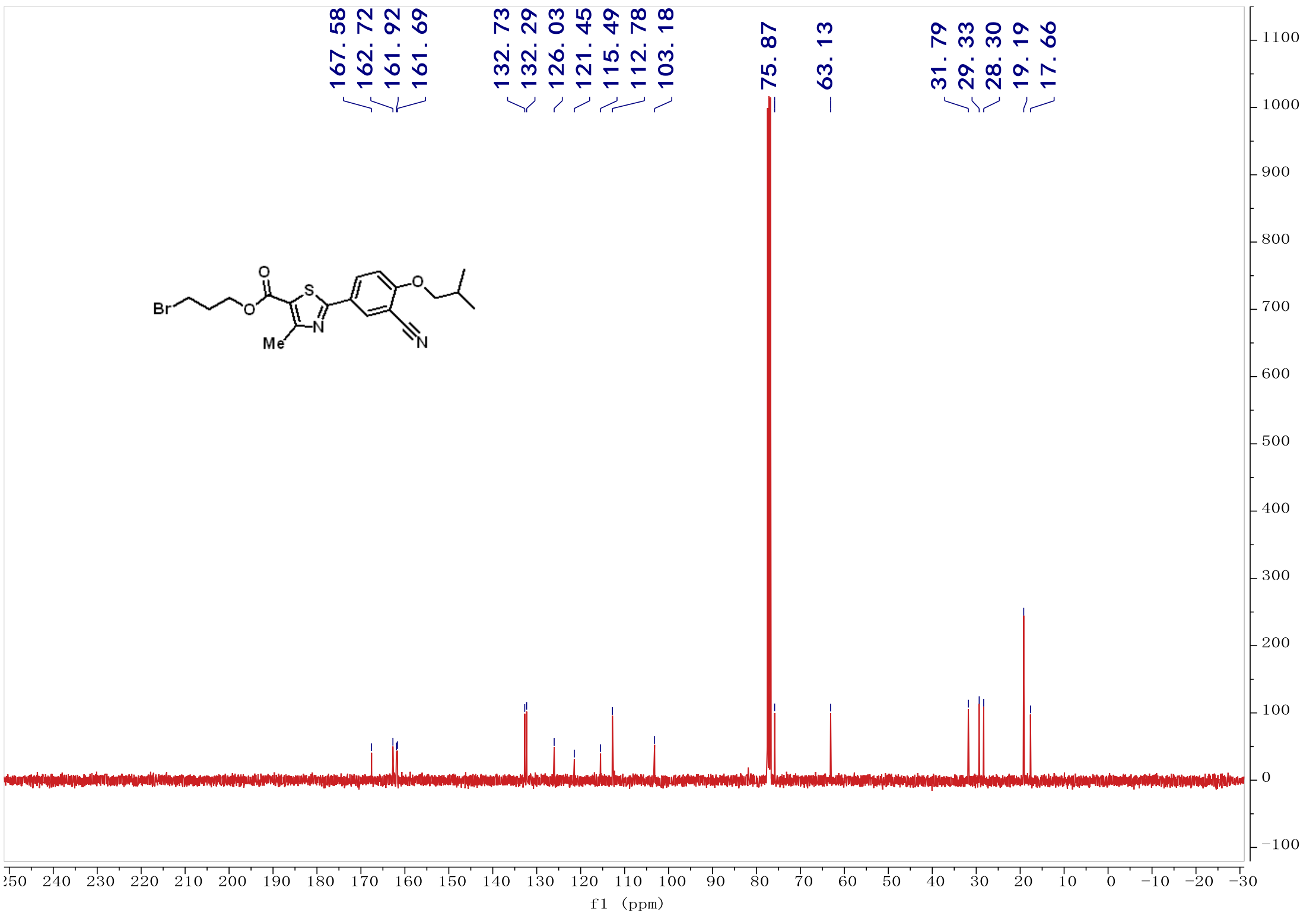

^{1}H NMR spectrum of **4-1** (400 MHz, $CDCl_3$).

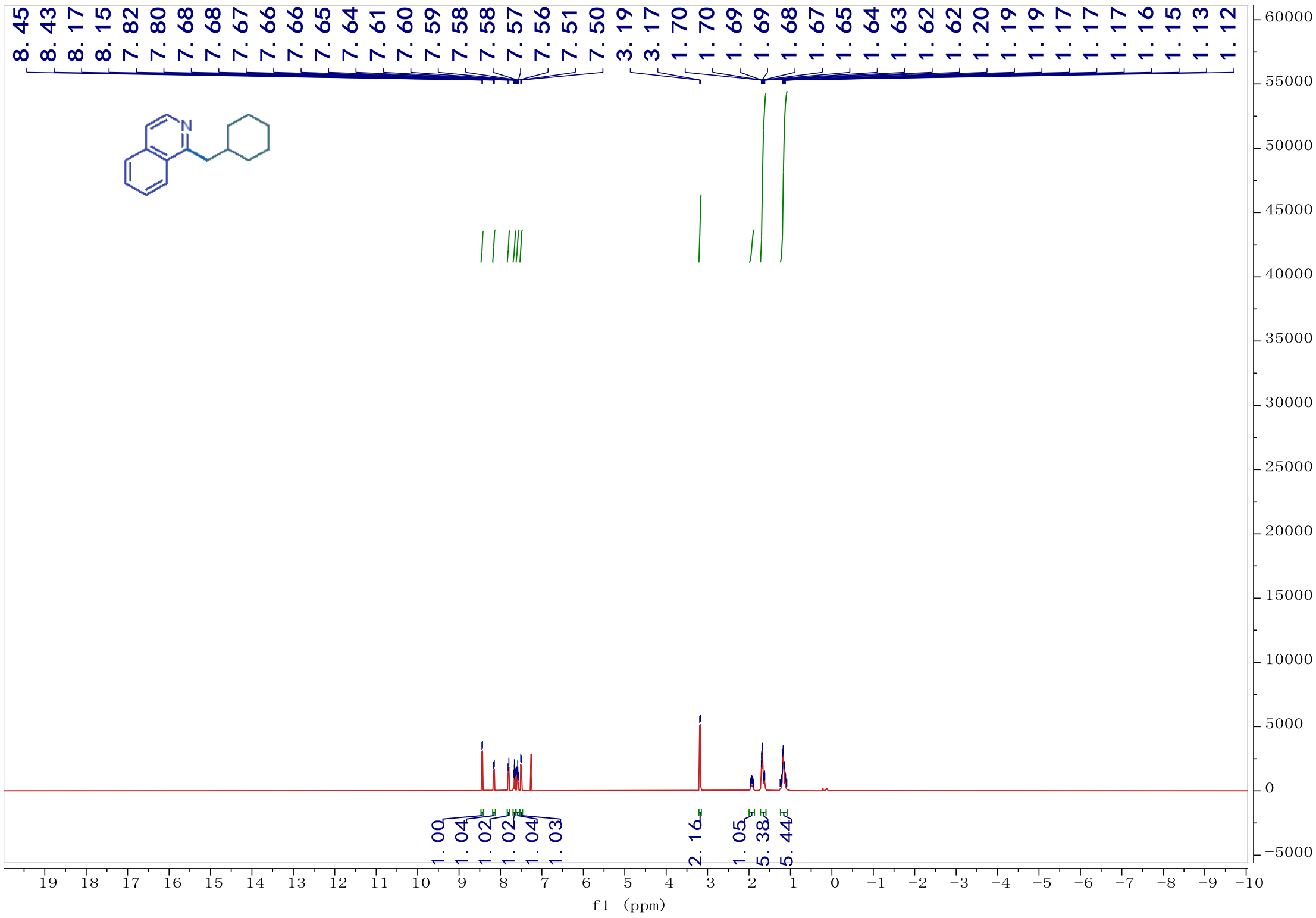

^{13}C{^{1}H} NMR spectrum of **4-1** (101 MHz, $CDCl_3$).

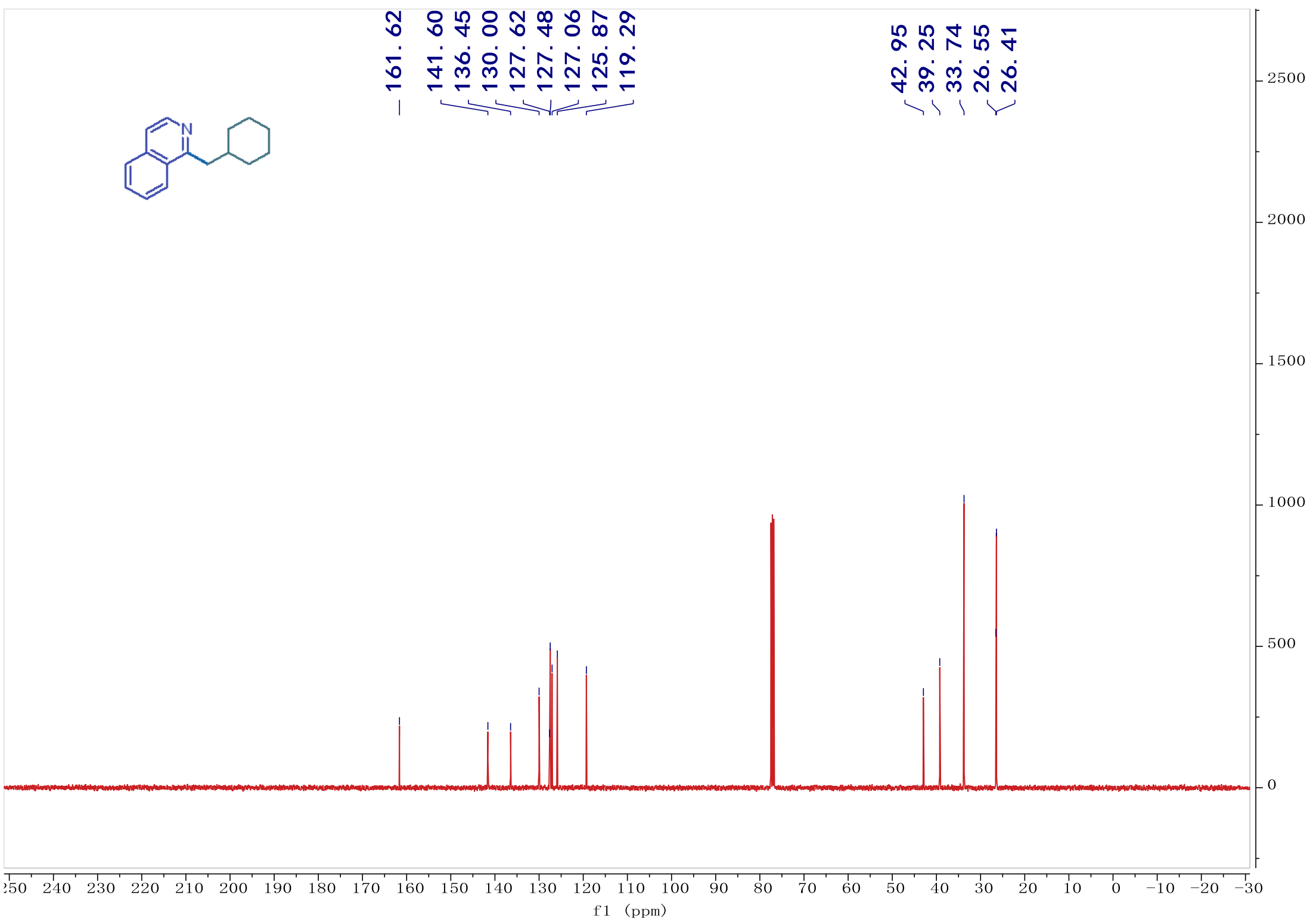

^{1}H NMR spectrum of **4-2** (400 MHz, $CDCl_3$).

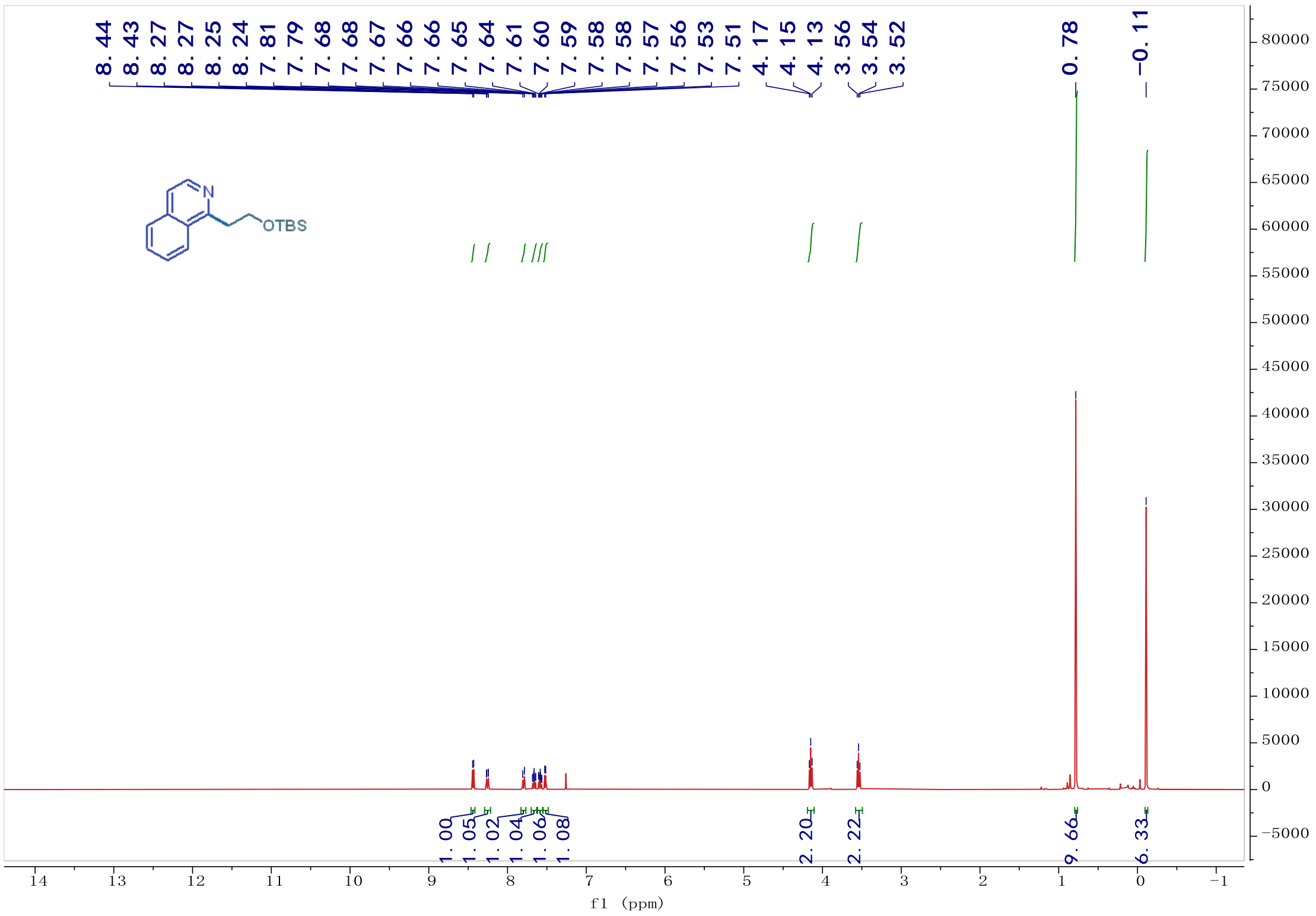

^{13}C{^{1}H} NMR spectrum of **4-2** (101 MHz, $CDCl_3$).

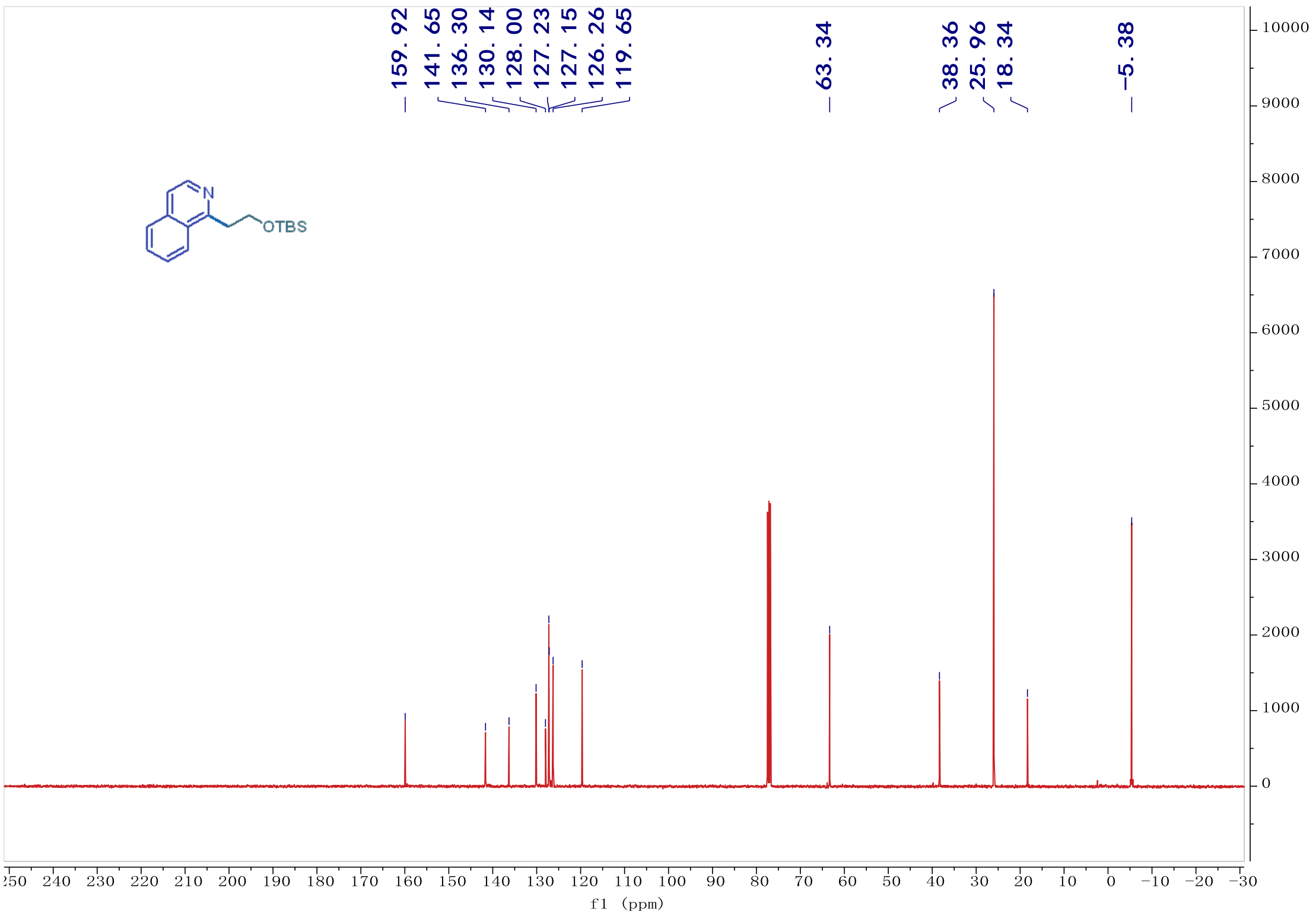

^{1}H NMR spectrum of **4-3** (400 MHz, $CDCl_3$).

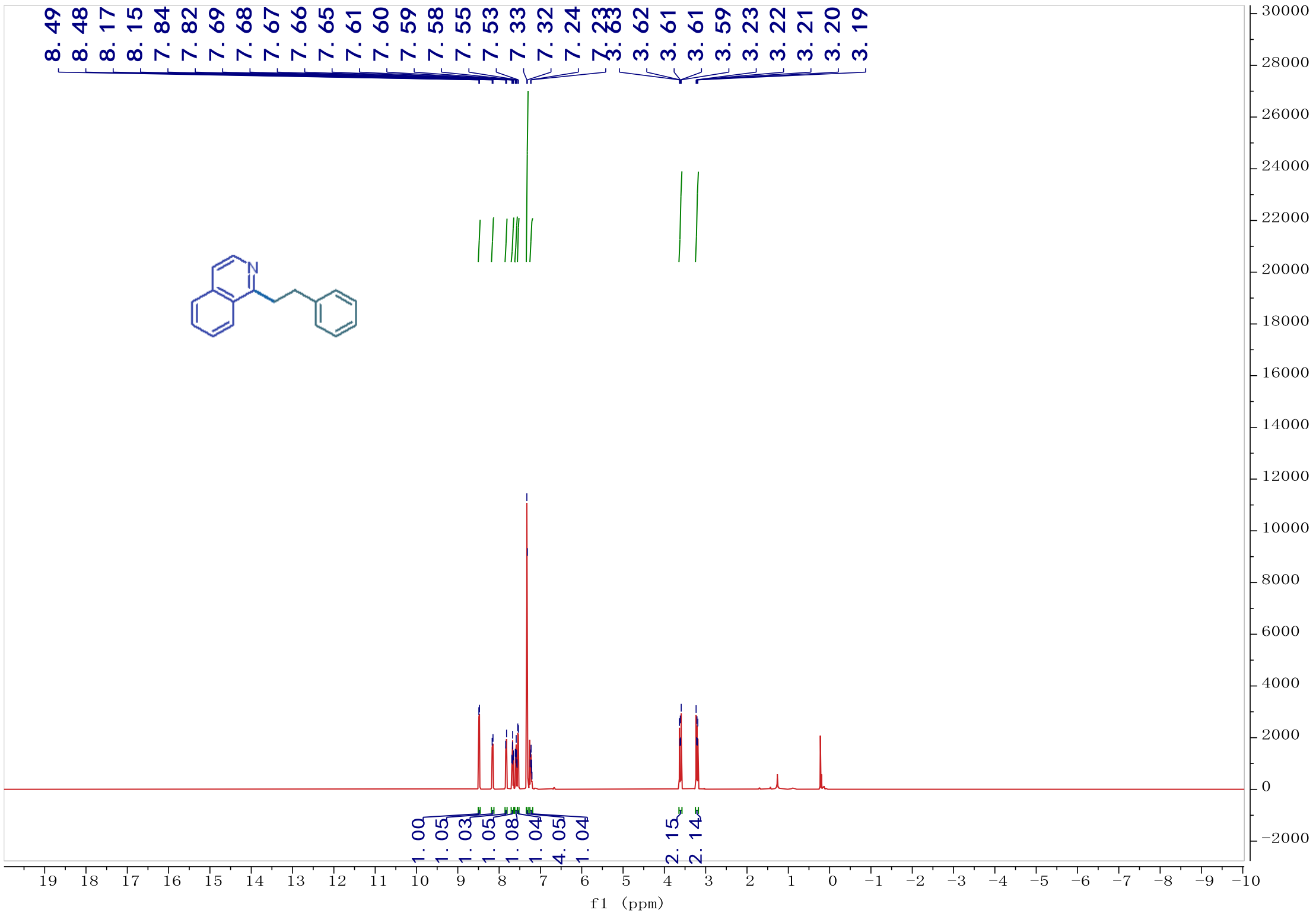

^{13}C{^{1}H} NMR spectrum of **4-3** (101 MHz, $CDCl_3$).

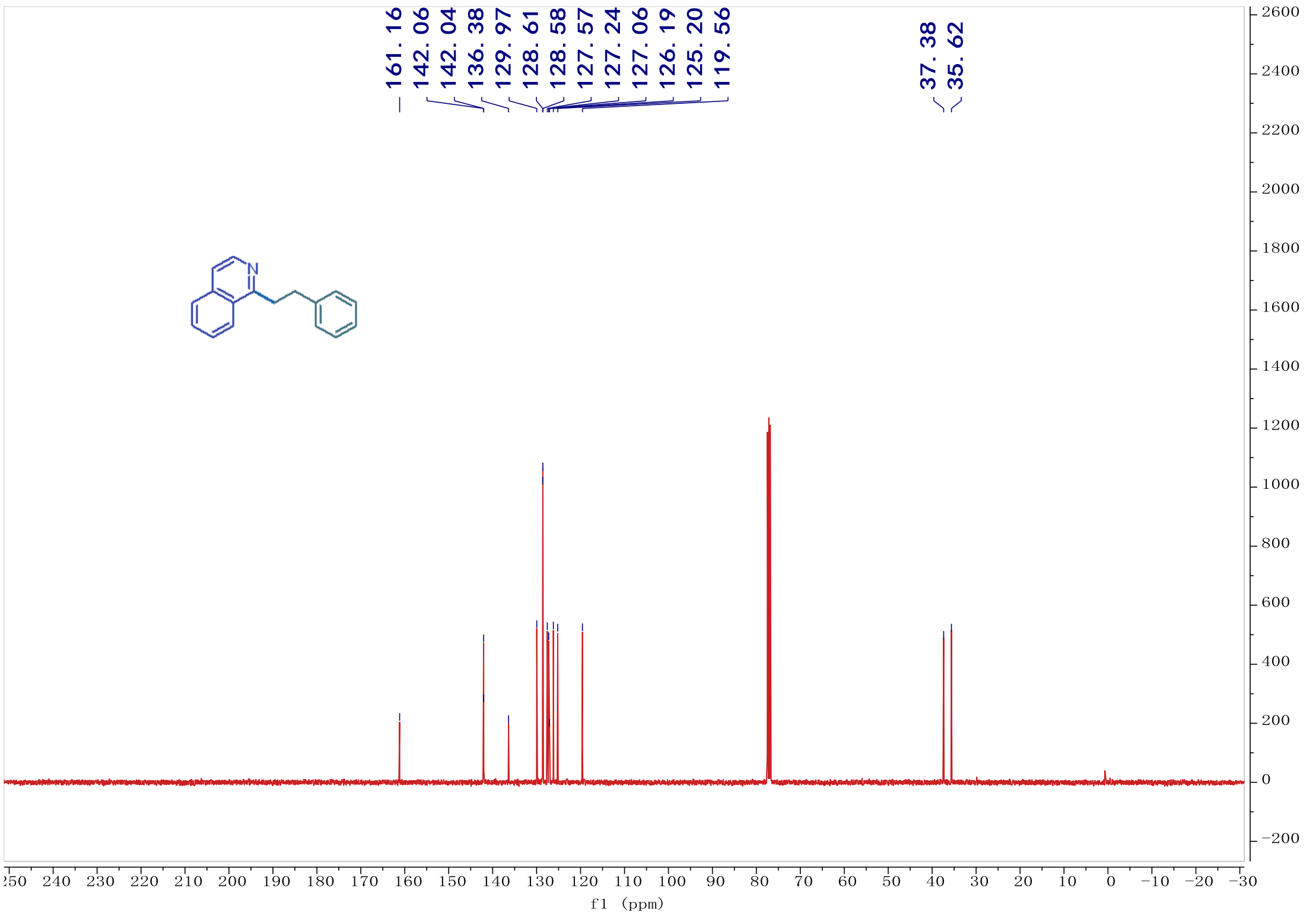

^{1}H NMR spectrum of **4-4** (400 MHz, $CDCl_3$).

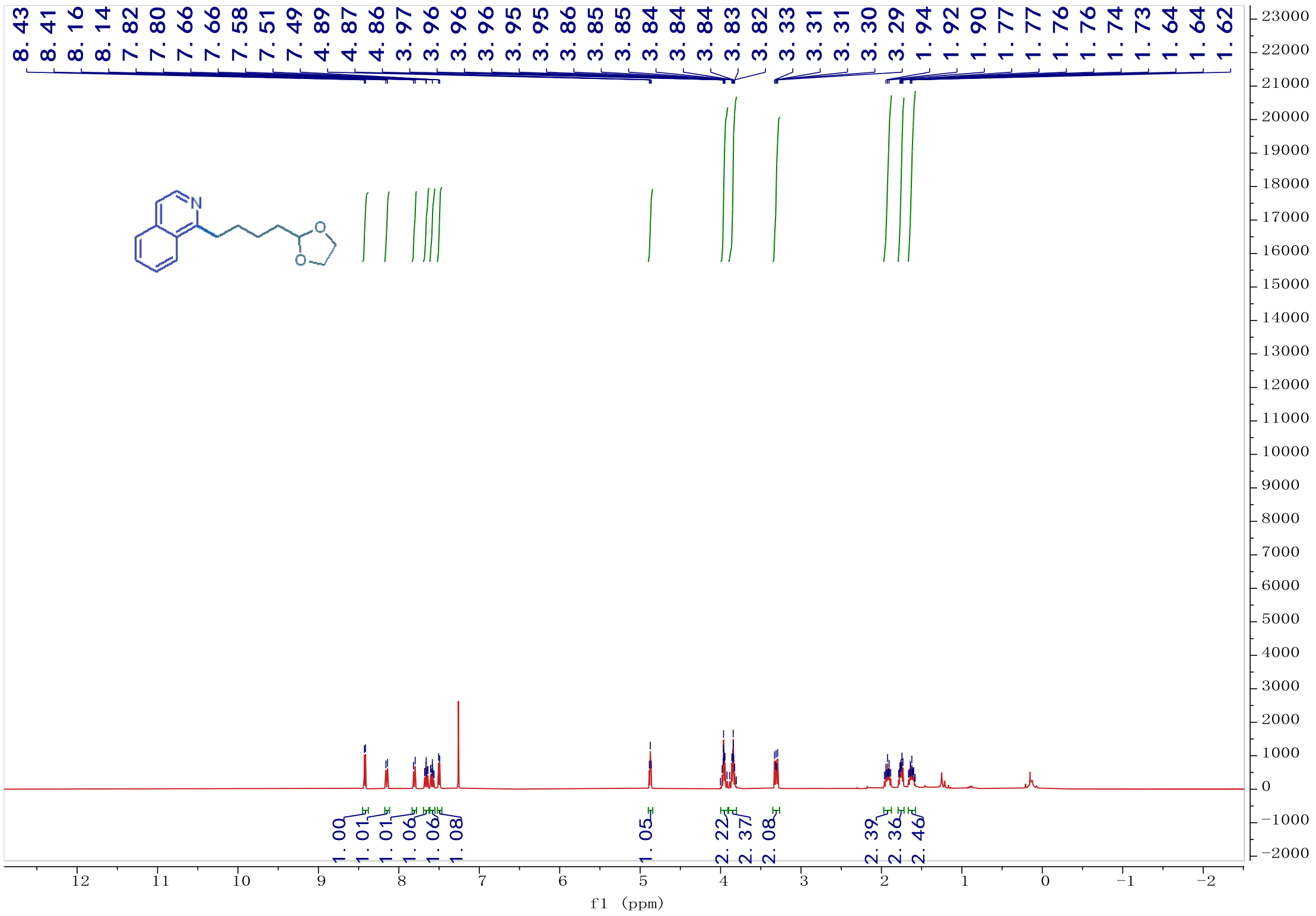

^{13}C{^{1}H} NMR spectrum of **4-4** (101 MHz, $CDCl_3$).

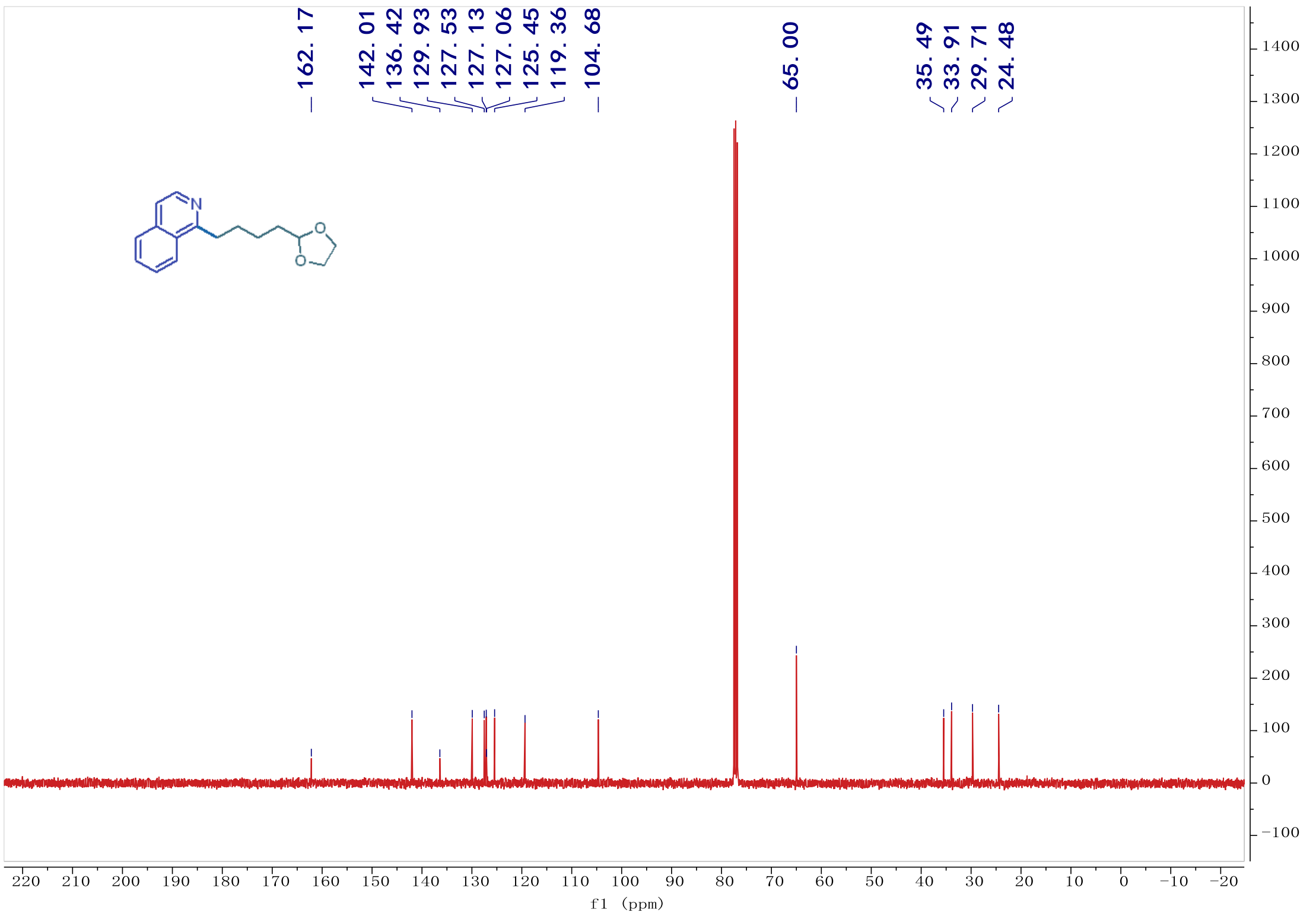

^{1}H NMR spectrum of **4-5** (400 MHz, $CDCl_3$).

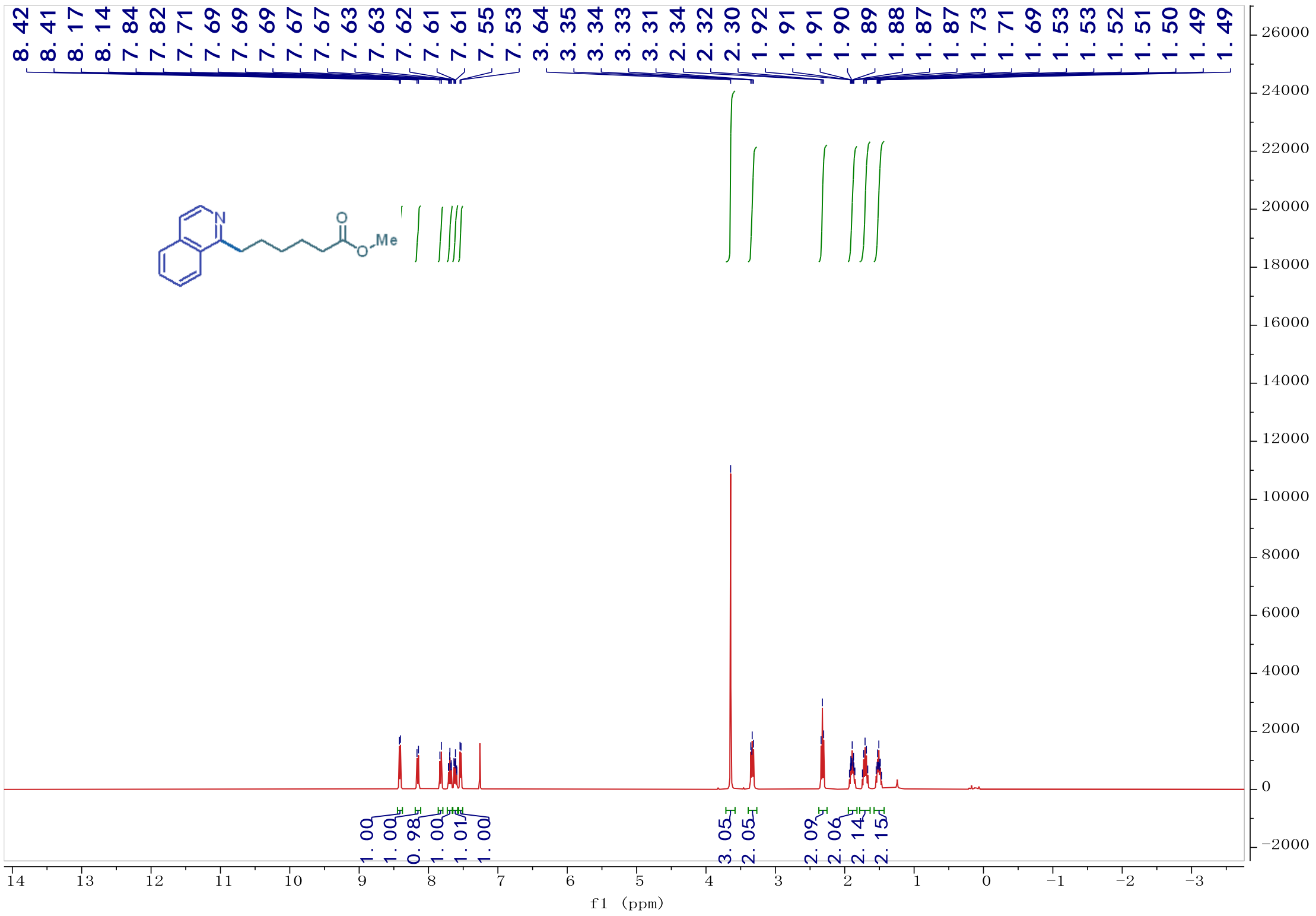

$^{13}C\{^{1}H\}$ NMR spectrum of **4-5** (101 MHz, $CDCl_3$).

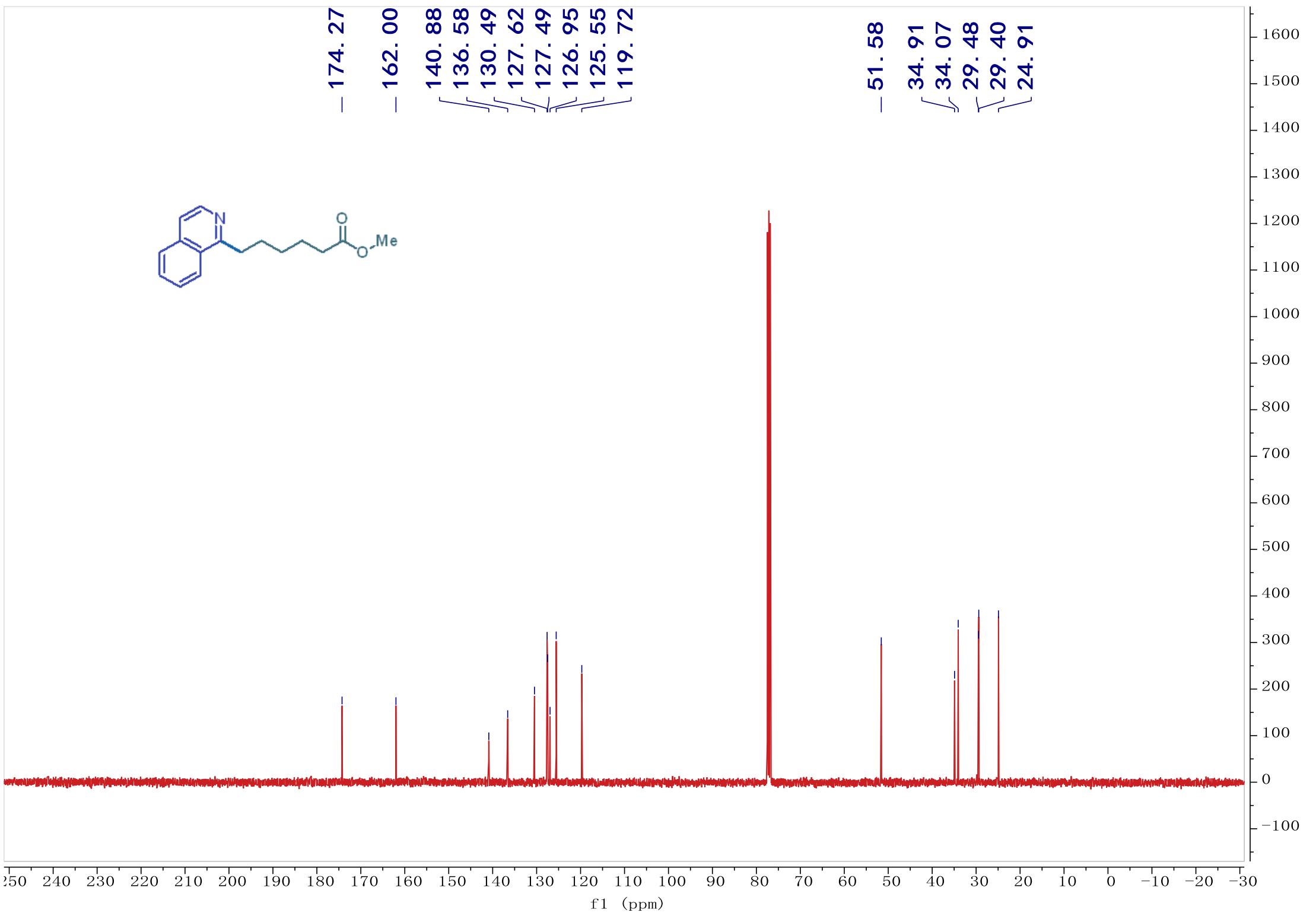

^{1}H NMR spectrum of **4-6** (400 MHz, $CDCl_3$).

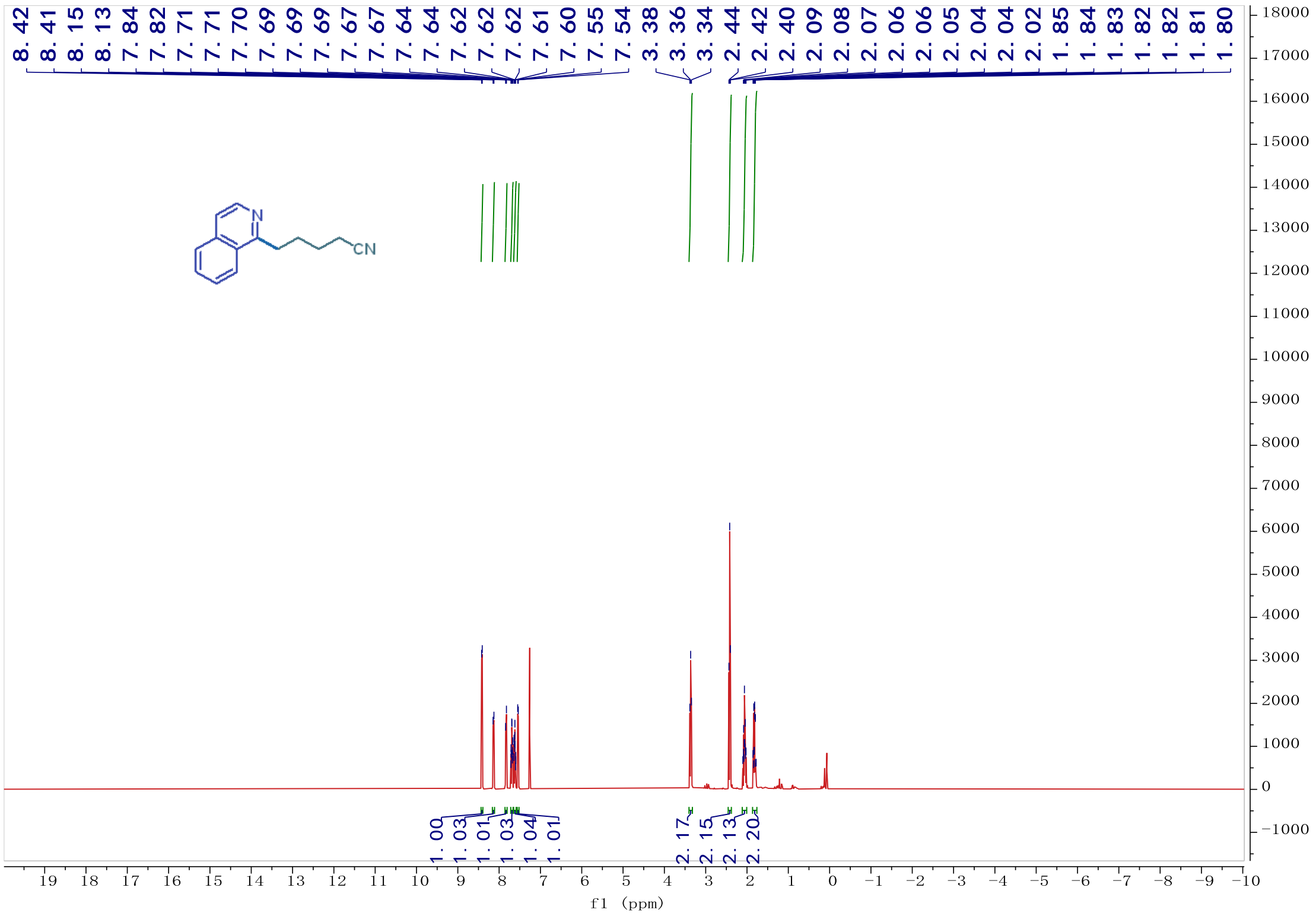

^{13}C{^{1}H} NMR spectrum of **4-6** (101 MHz, $CDCl_3$).

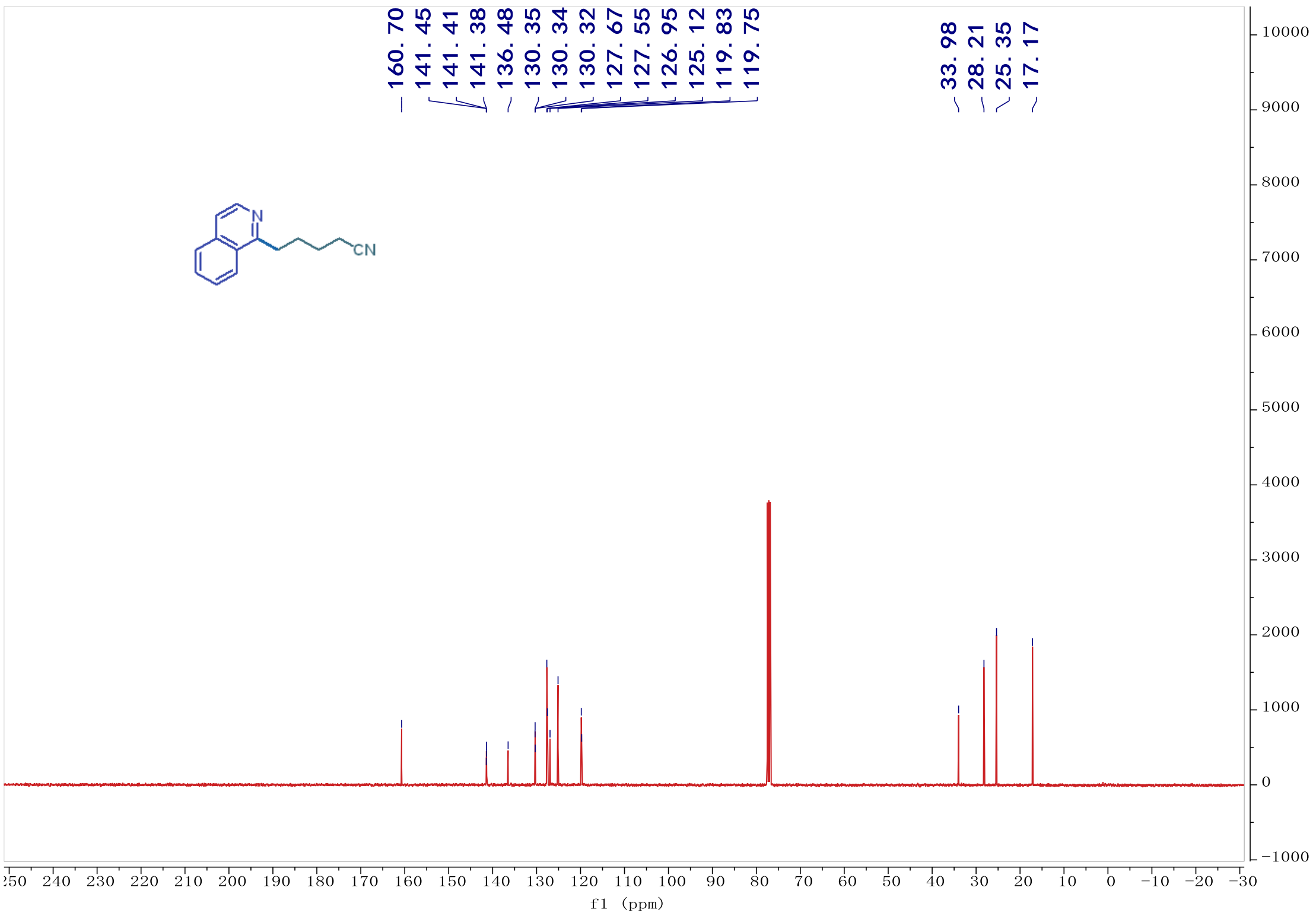

^{1}H NMR spectrum of **4-7** (400 MHz, $CDCl_3$).

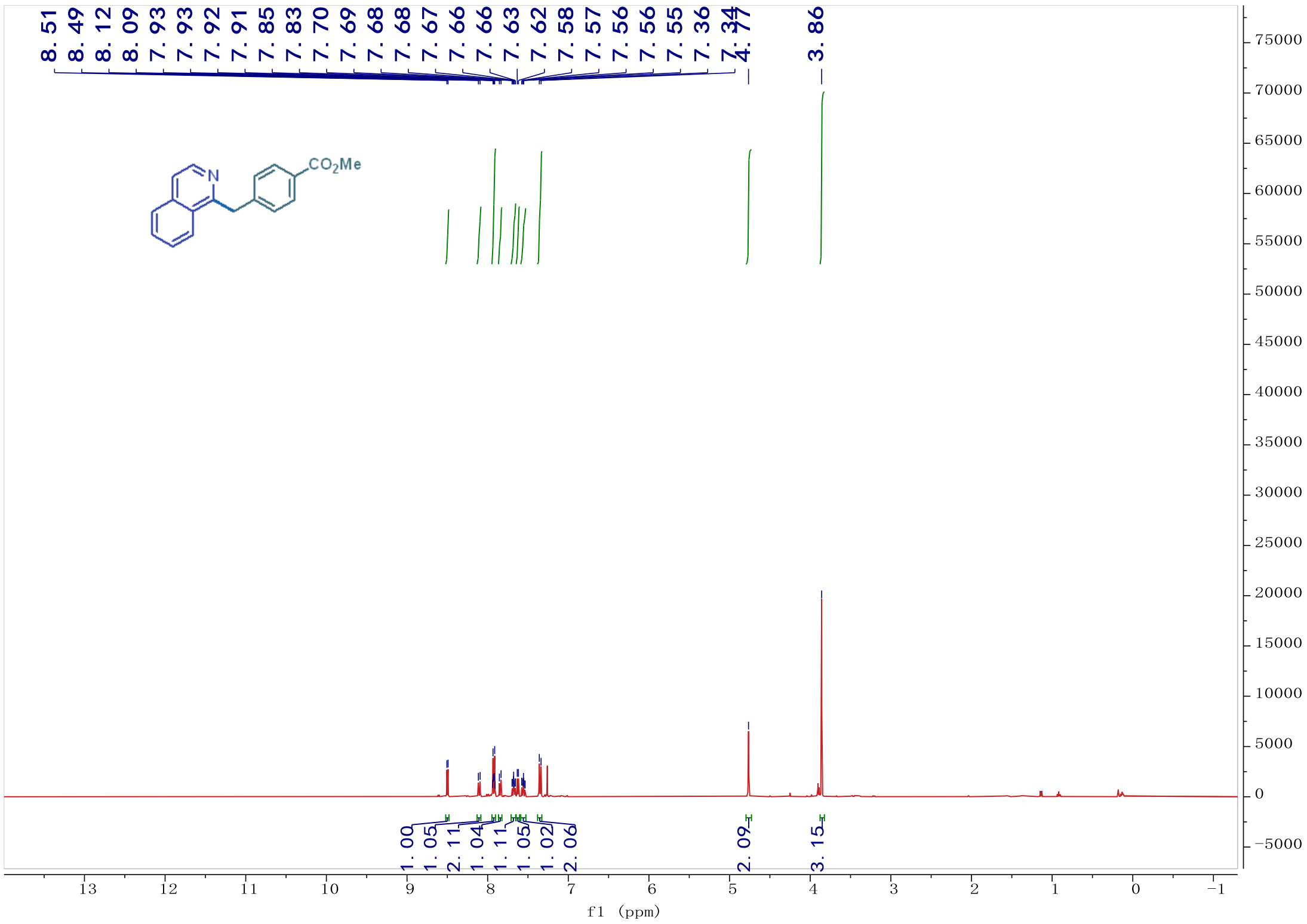

^{13}C{^{1}H} NMR spectrum of **4-7** (101 MHz, $CDCl_3$).

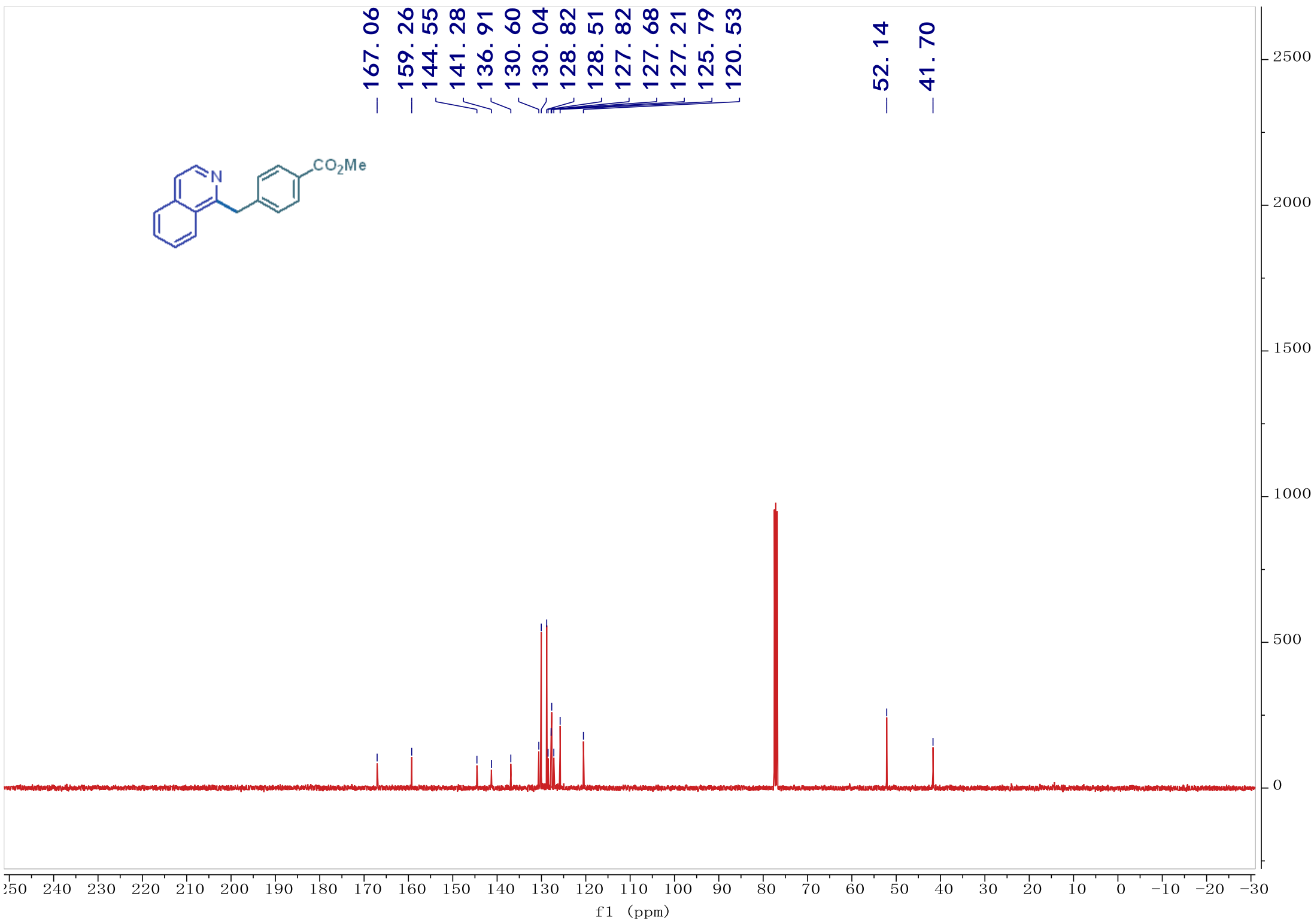

^{1}H NMR spectrum of **4-8** (400 MHz, $CDCl_3$).

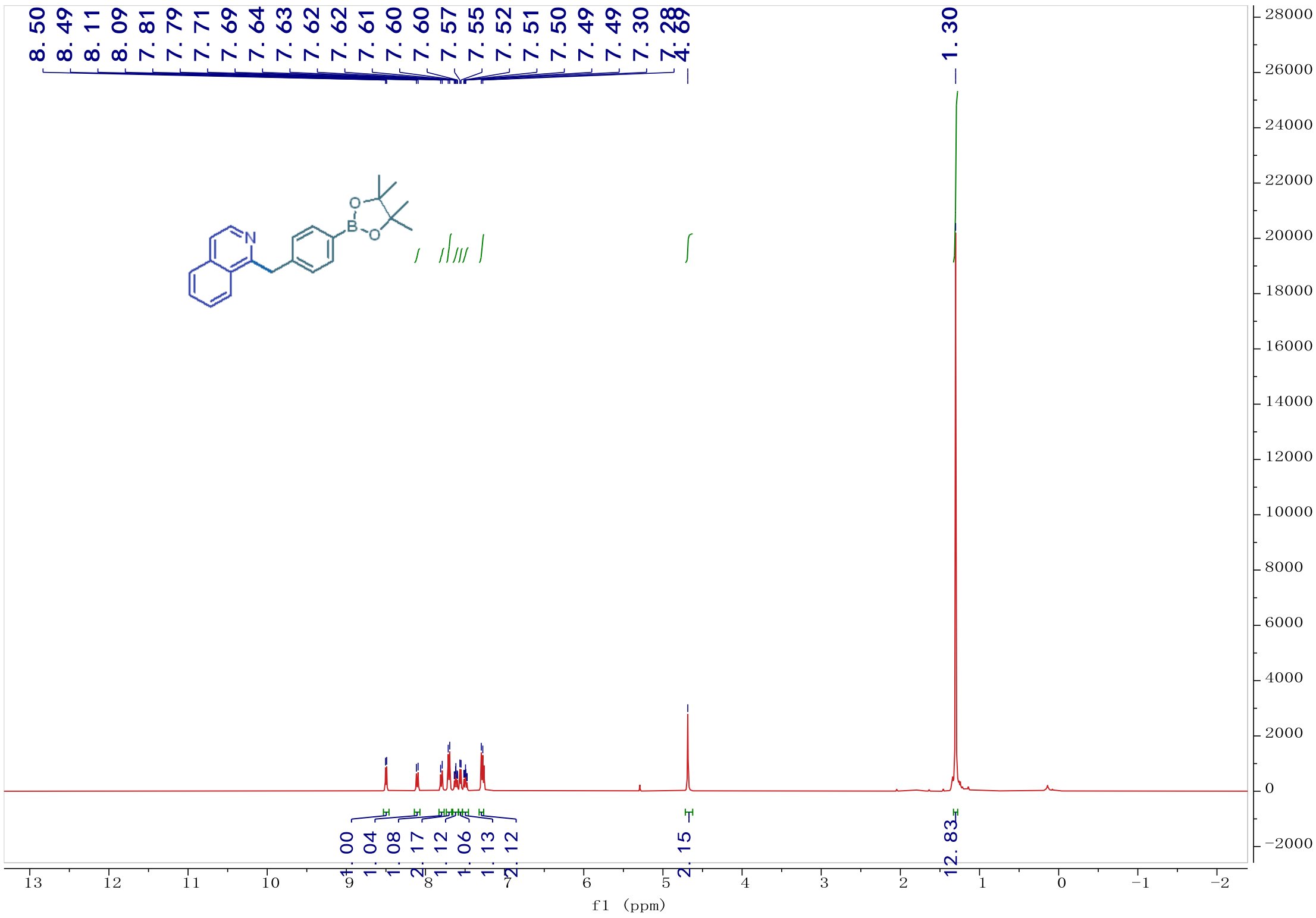

$^{13}C\{^{1}H\}$ NMR spectrum of **4-8** (101 MHz, $CDCl_3$).

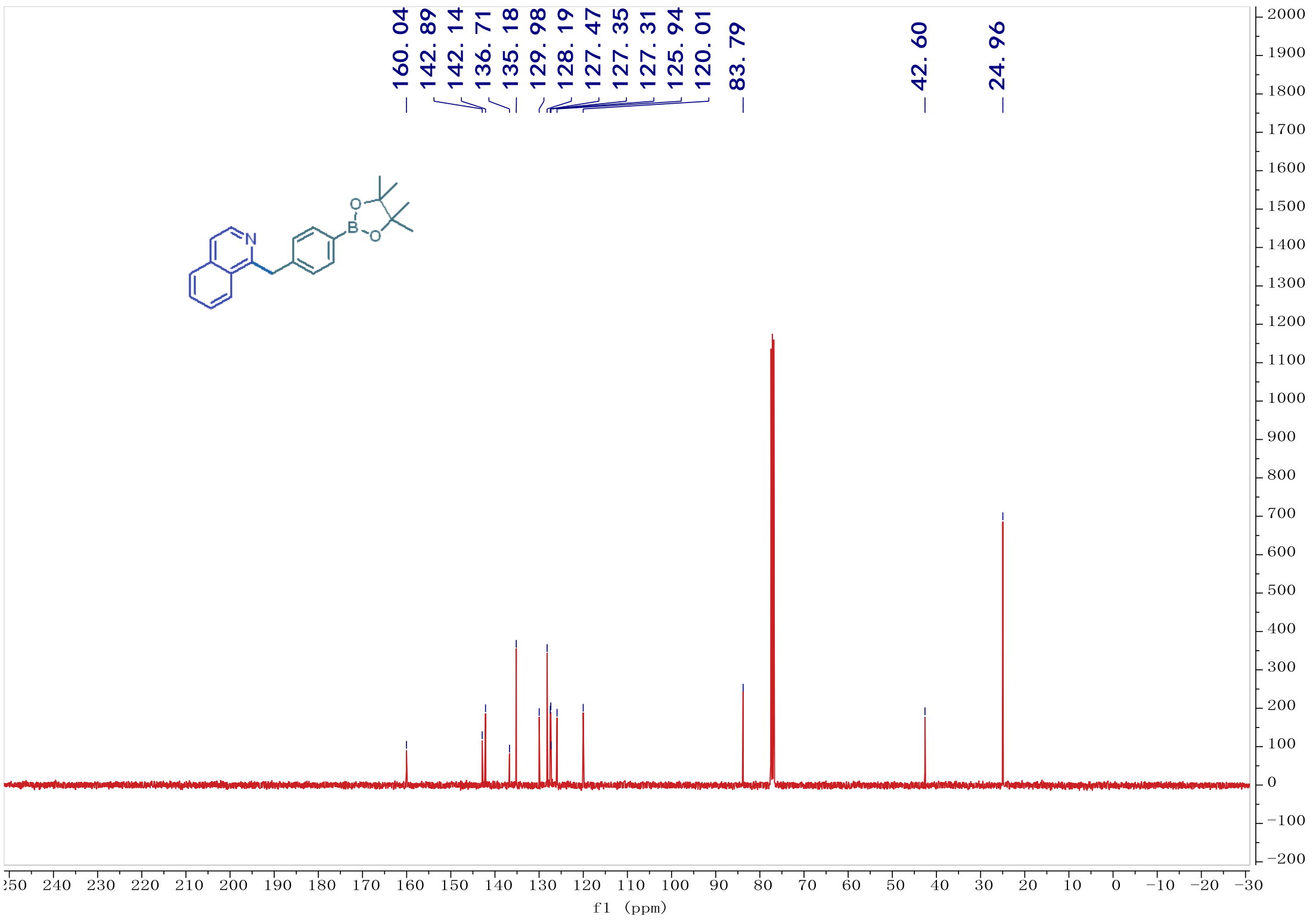

^{1}H NMR spectrum of **4-9** (400 MHz, $CDCl_3$).

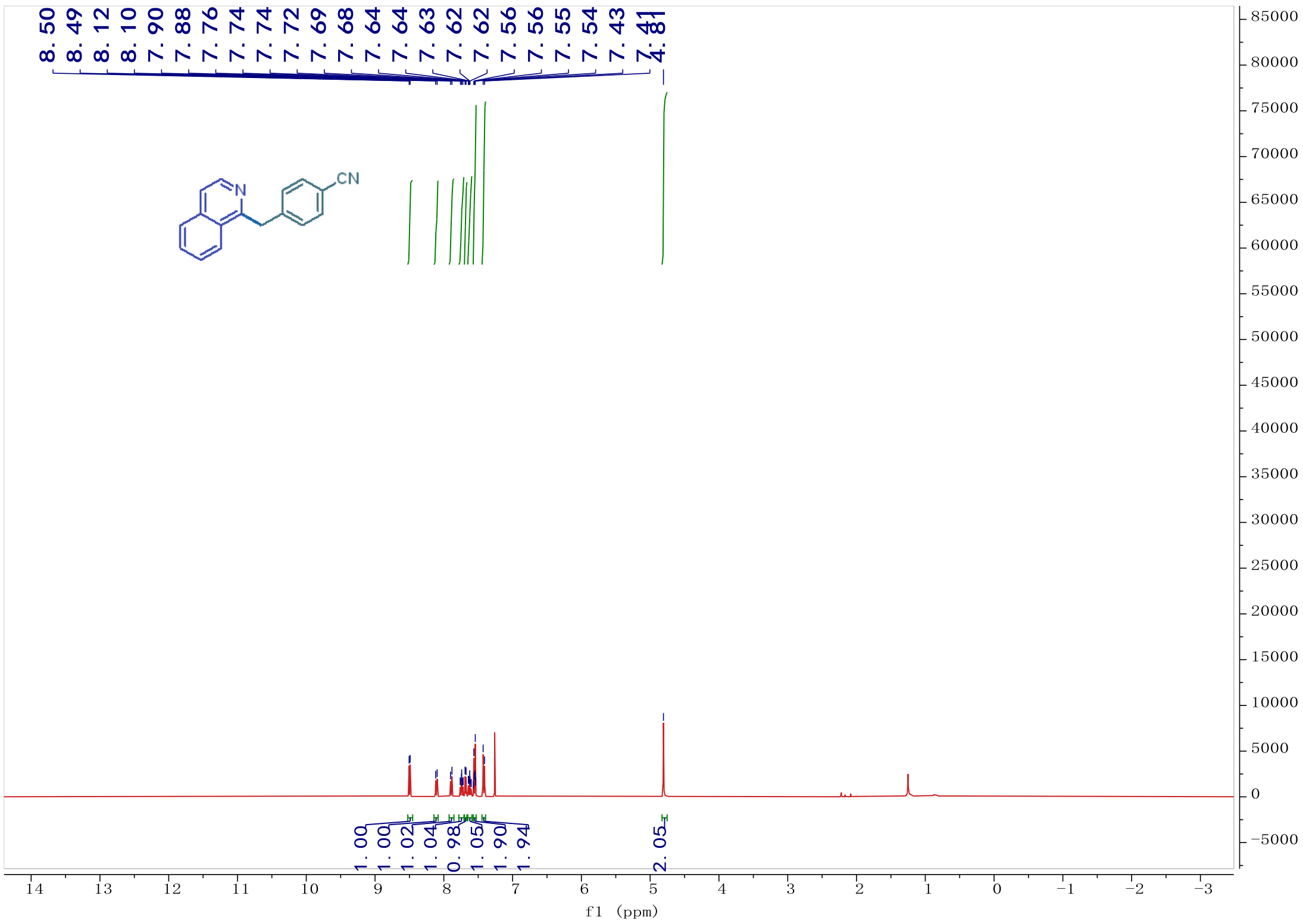

^{13}C{^{1}H} NMR spectrum of **4-9** (101 MHz, $CDCl_3$).

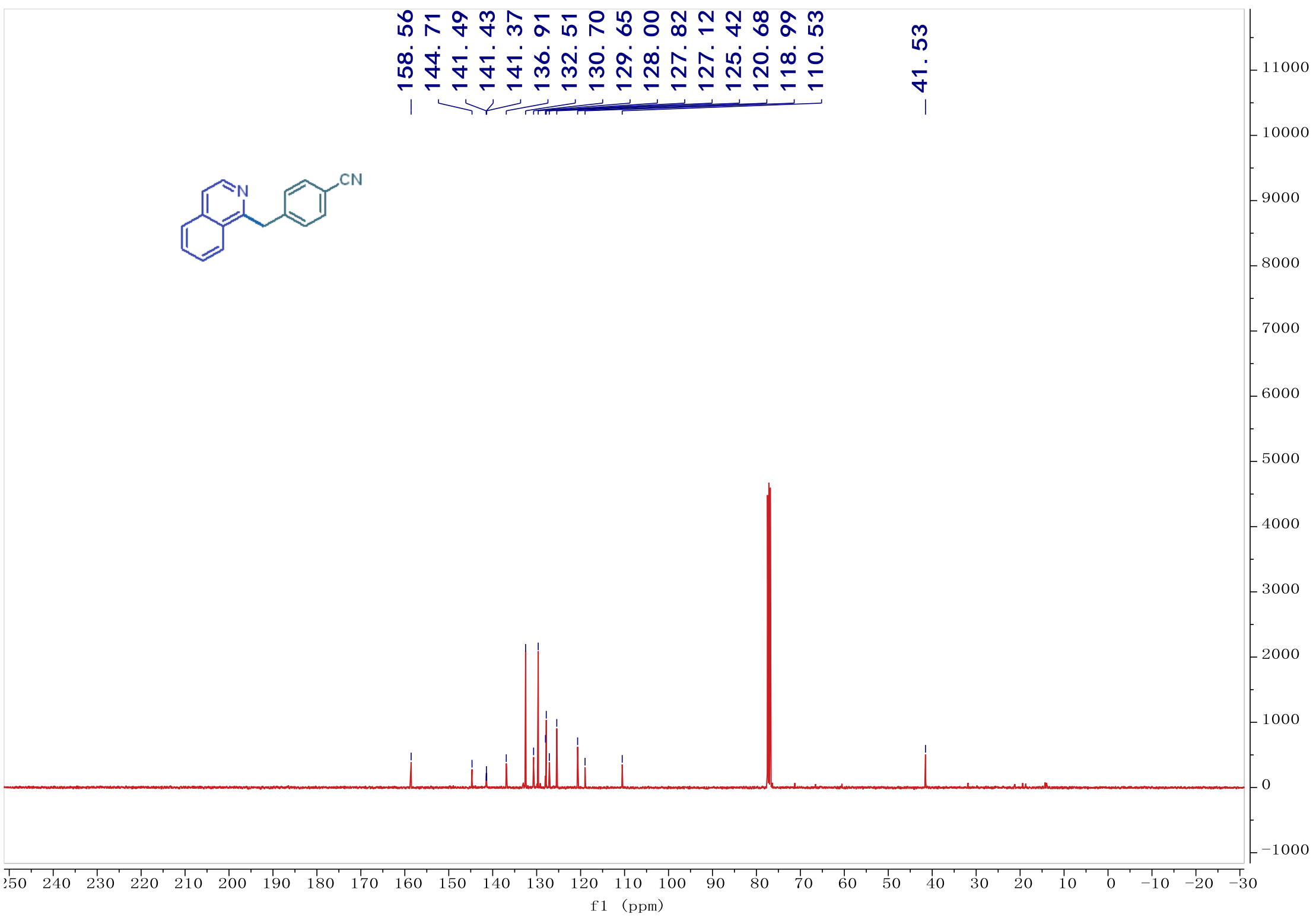

^{1}H NMR spectrum of **4-10** (400 MHz, $CDCl_3$).

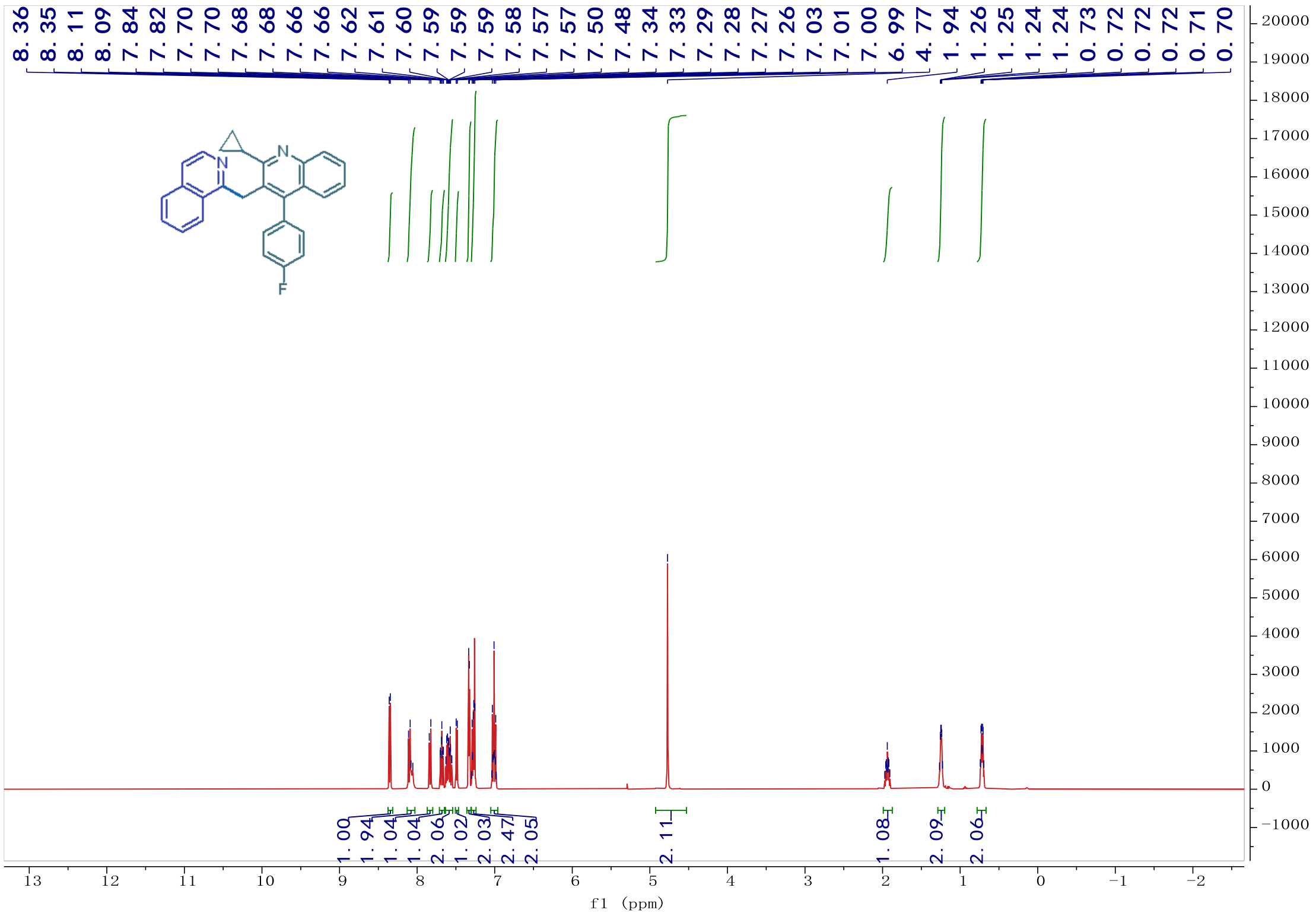

^{13}C{^{1}H} NMR spectrum of **4-10** (101 MHz, $CDCl_3$).

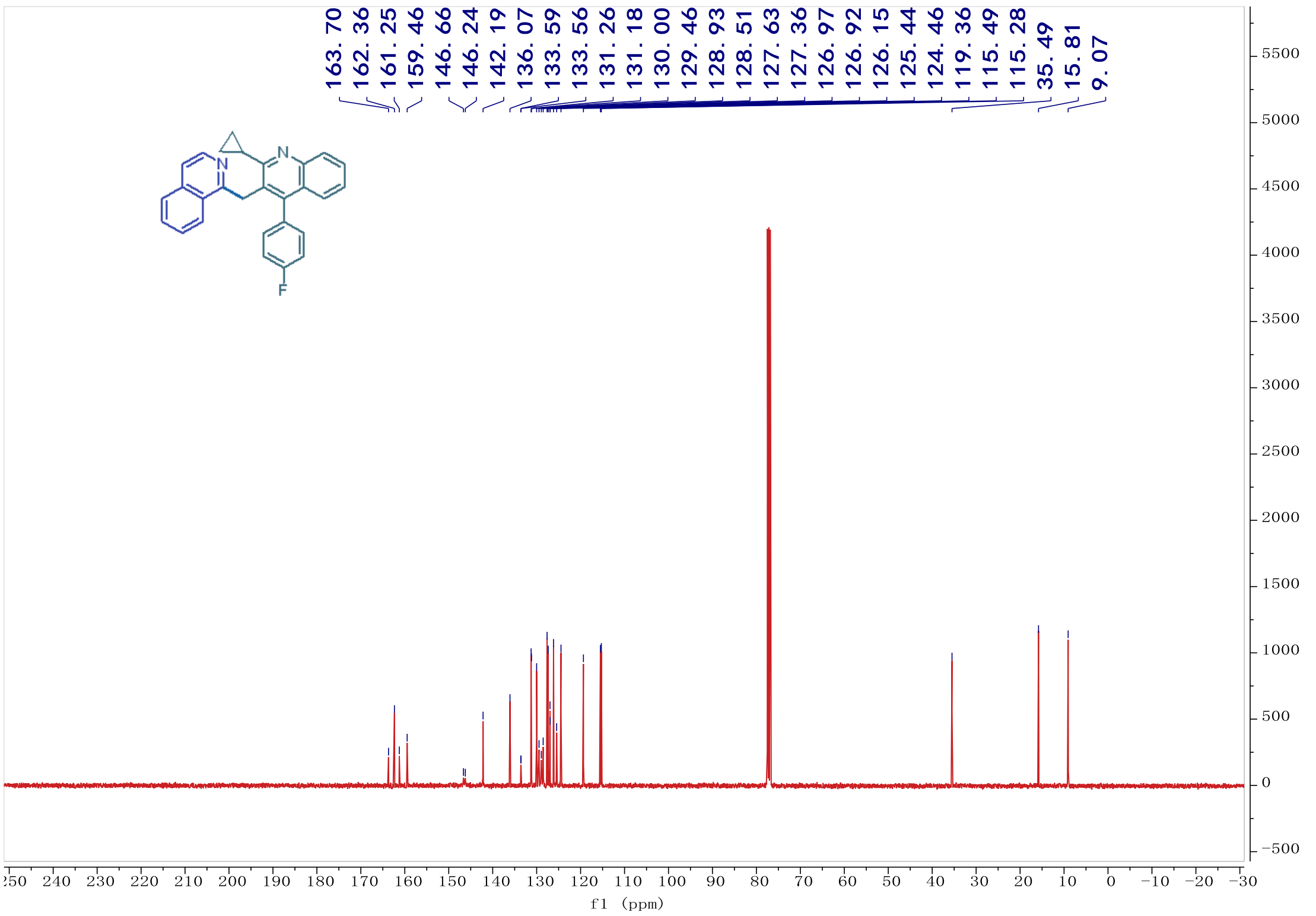

^{19}F NMR spectrum of **4-10** (376 MHz, $CDCl_3$).

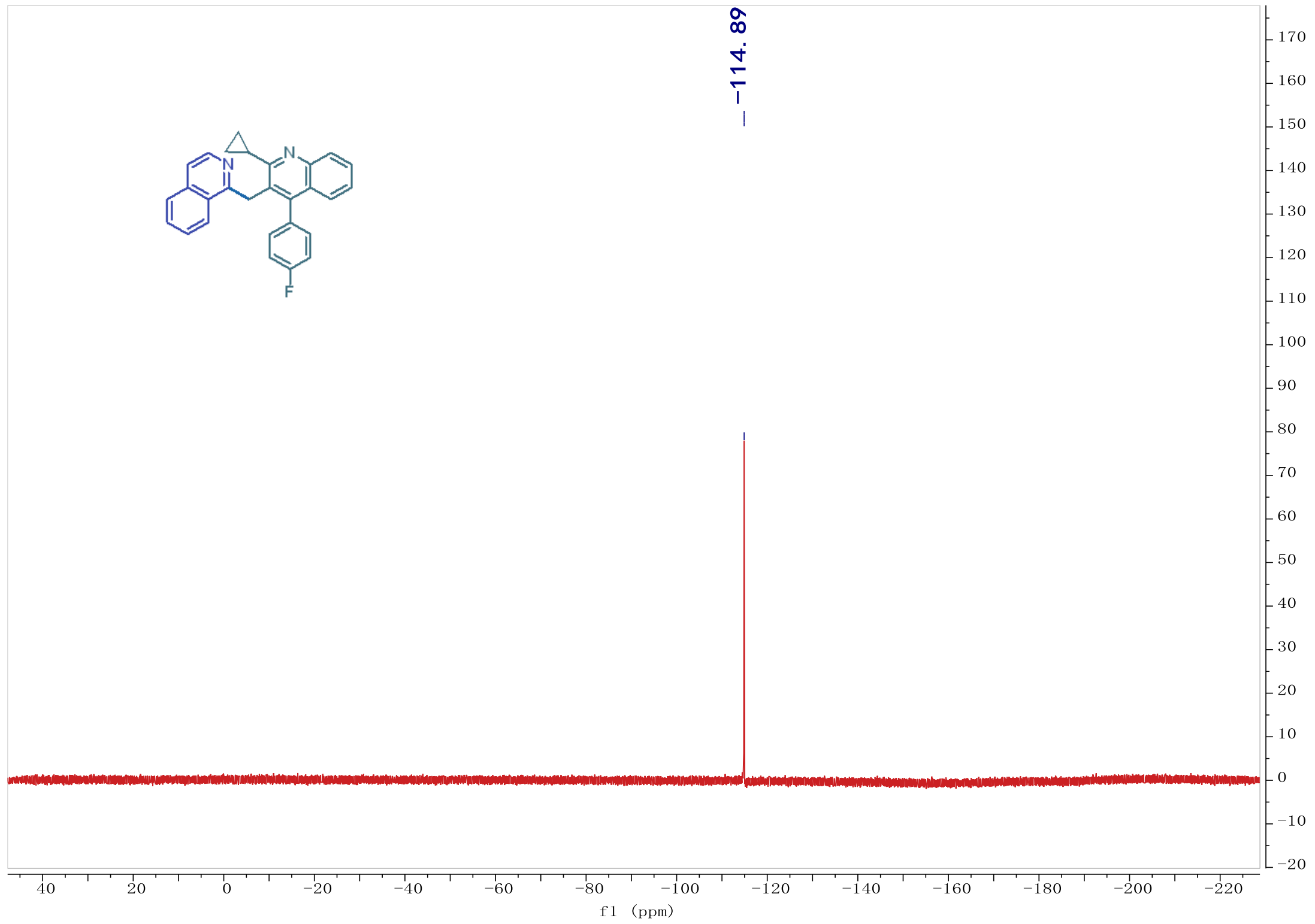

^{1}H NMR spectrum of **4-11** (400 MHz, $CDCl_3$).

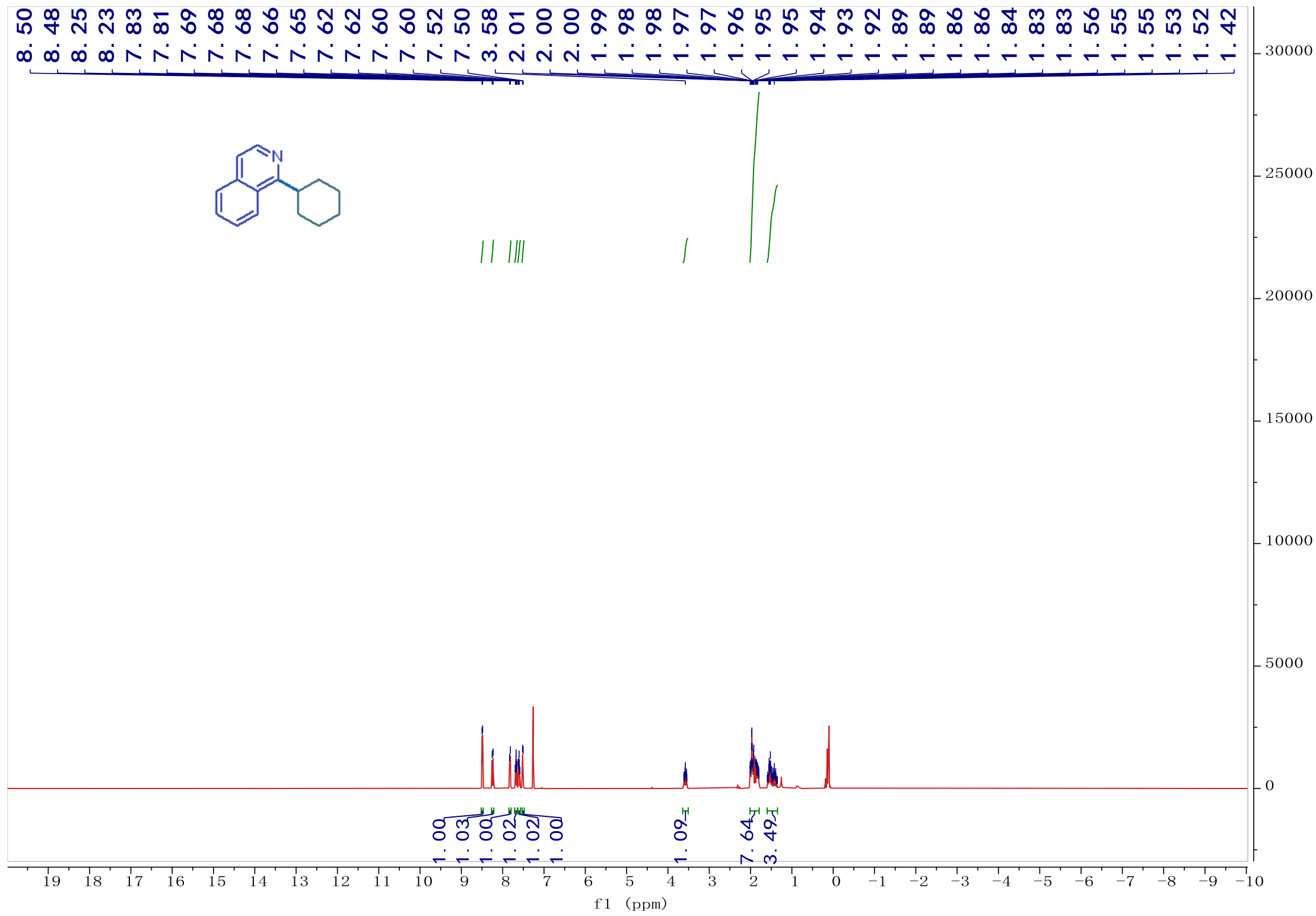

$^{13}C\{^{1}H\}$ NMR spectrum of **4-11** (101 MHz, $CDCl_3$).

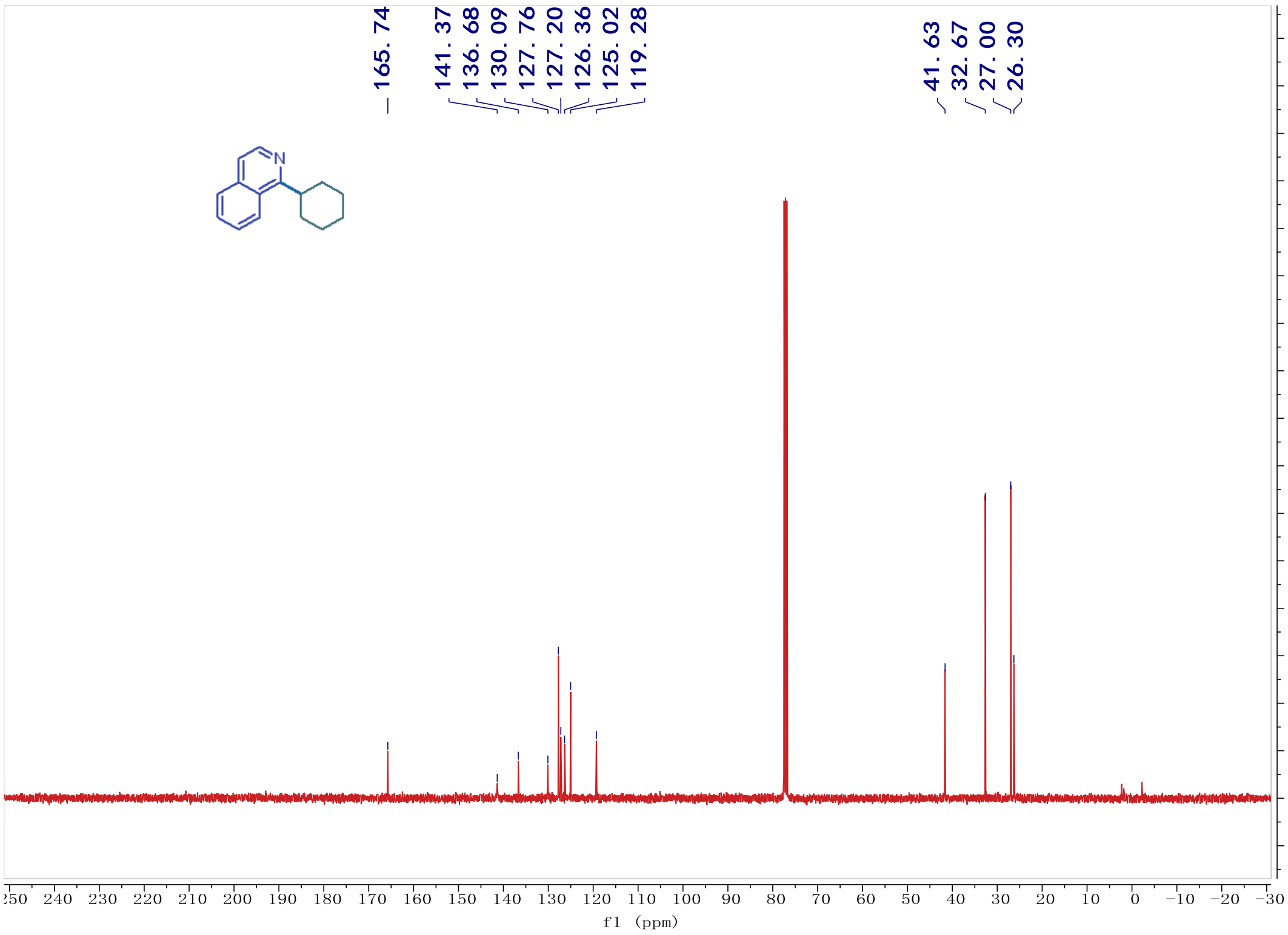

^{1}H NMR spectrum of **4-12** (400 MHz, $CDCl_3$).

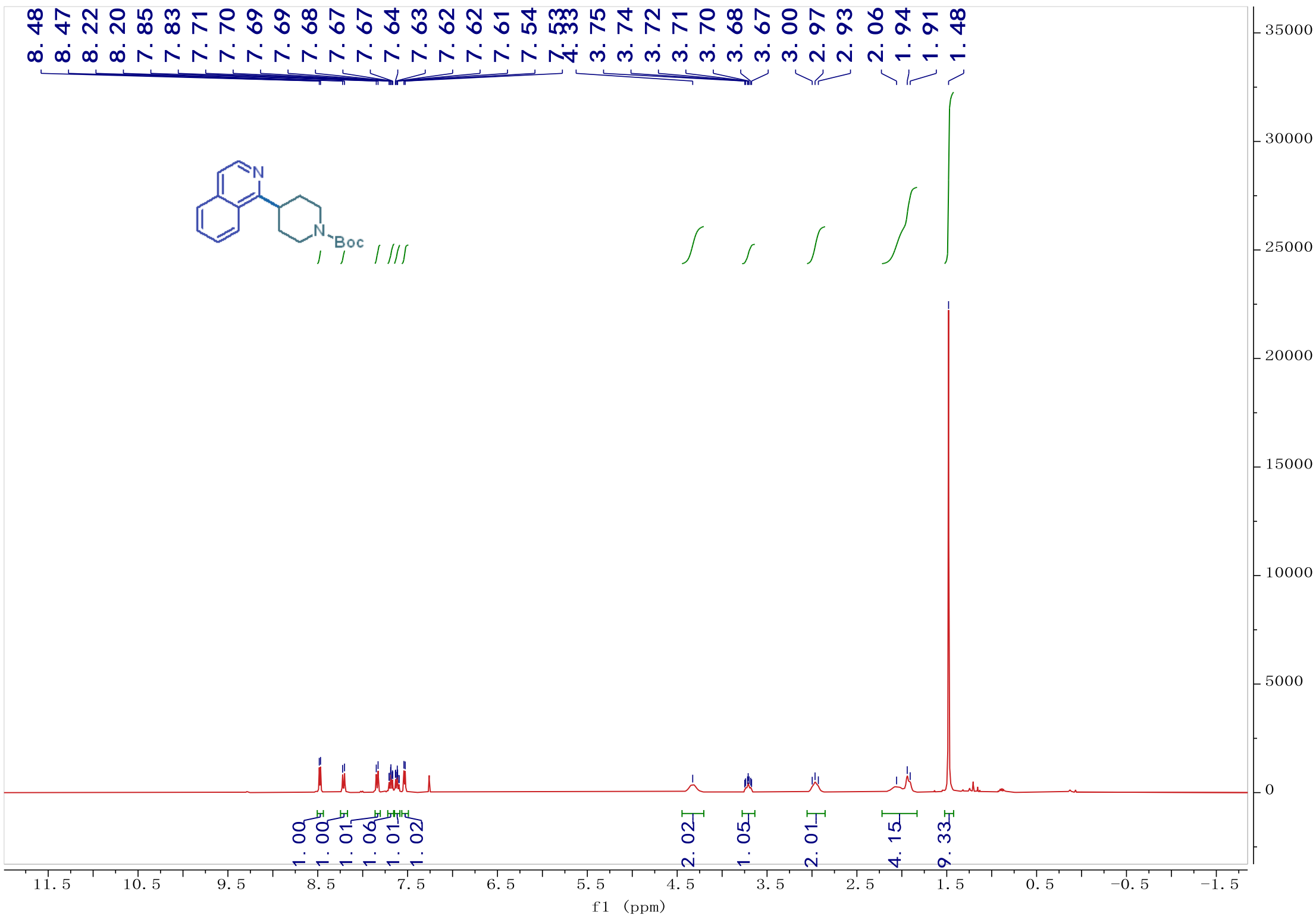

$^{13}C\{^{1}H\}$ NMR spectrum of **4-12** (101 MHz, $CDCl_3$).

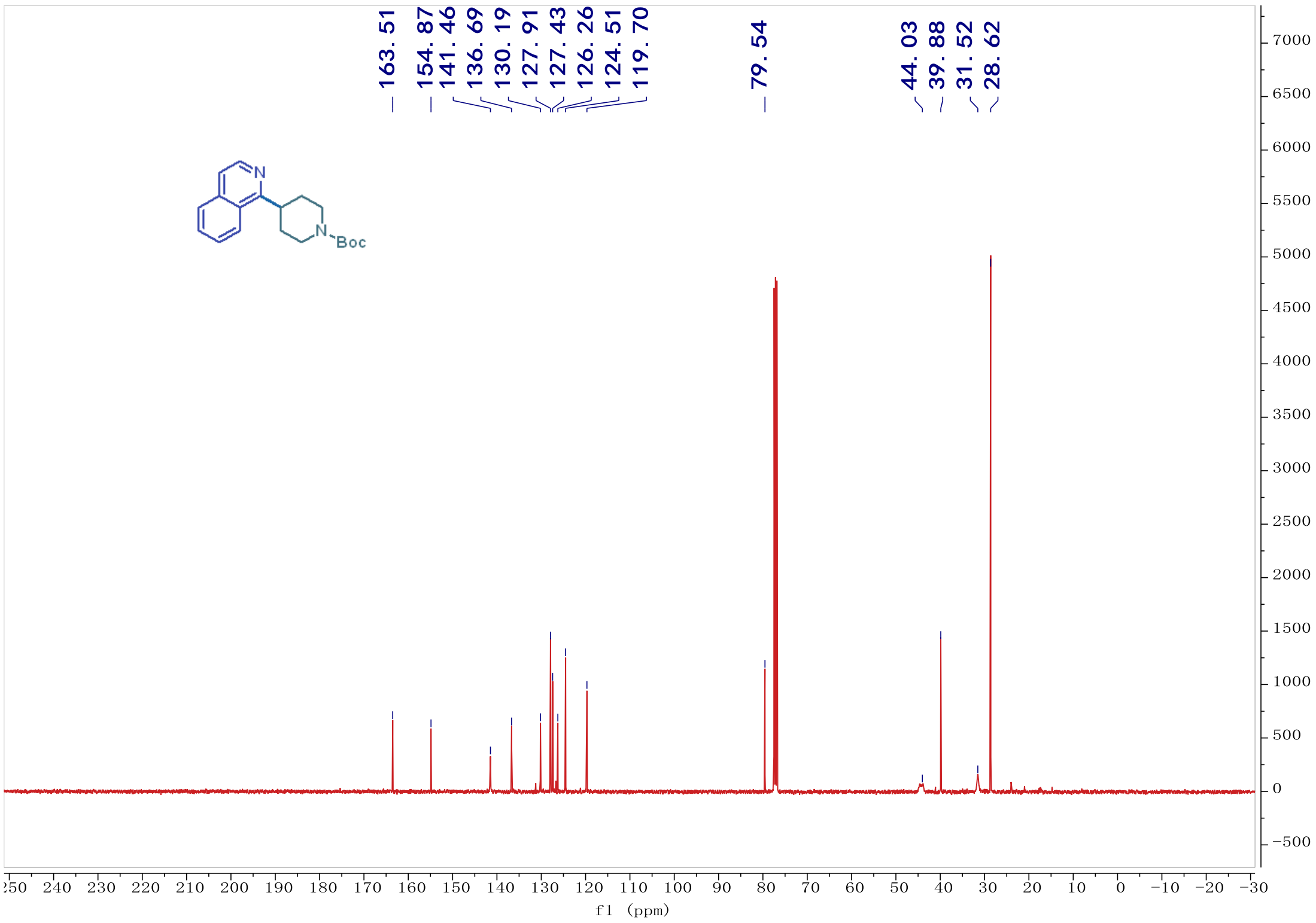

^{1}H NMR spectrum of **4-13** (400 MHz, $CDCl_3$).

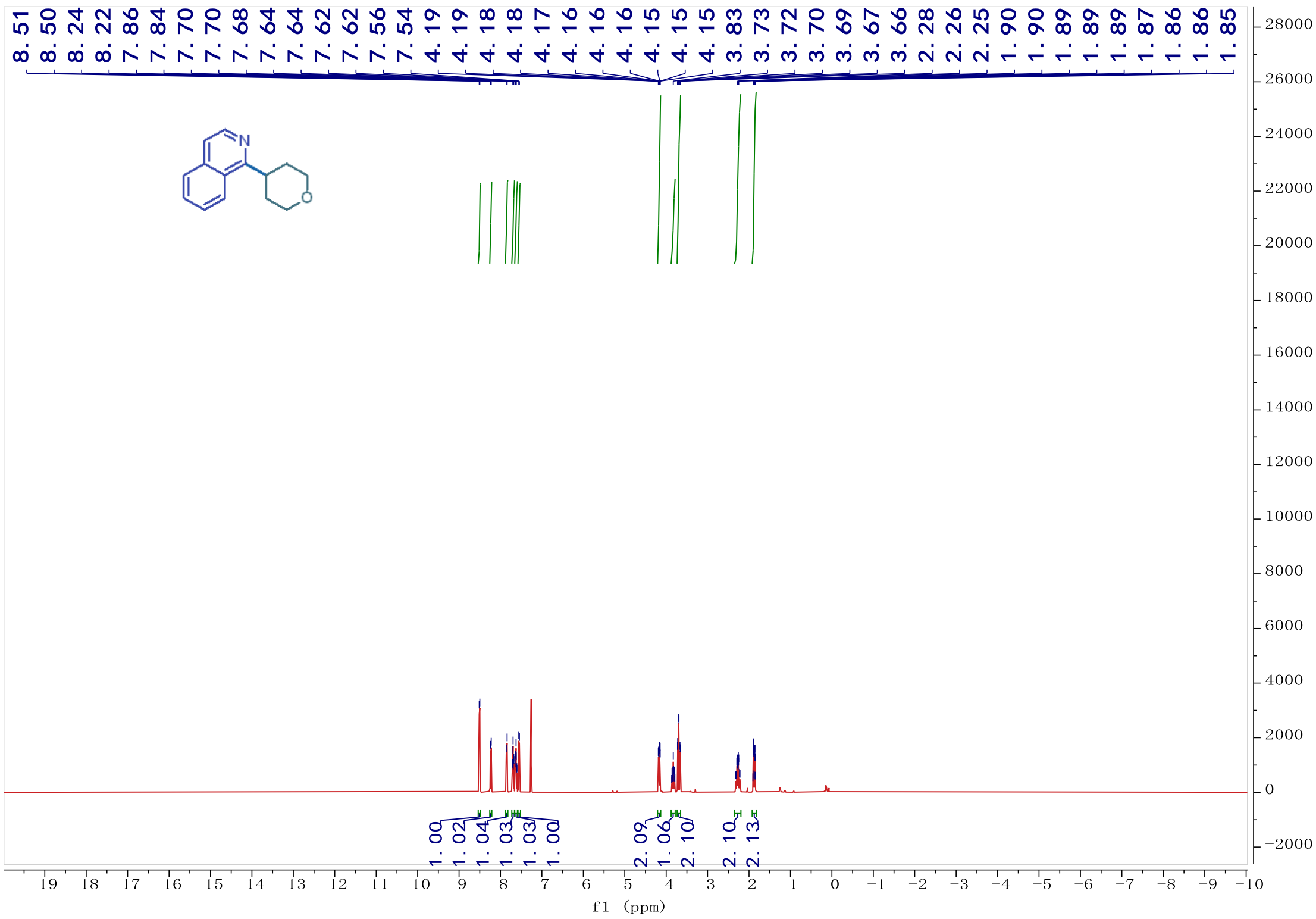

$^{13}C\{^{1}H\}$ NMR spectrum of **4-13** (101 MHz, $CDCl_3$).

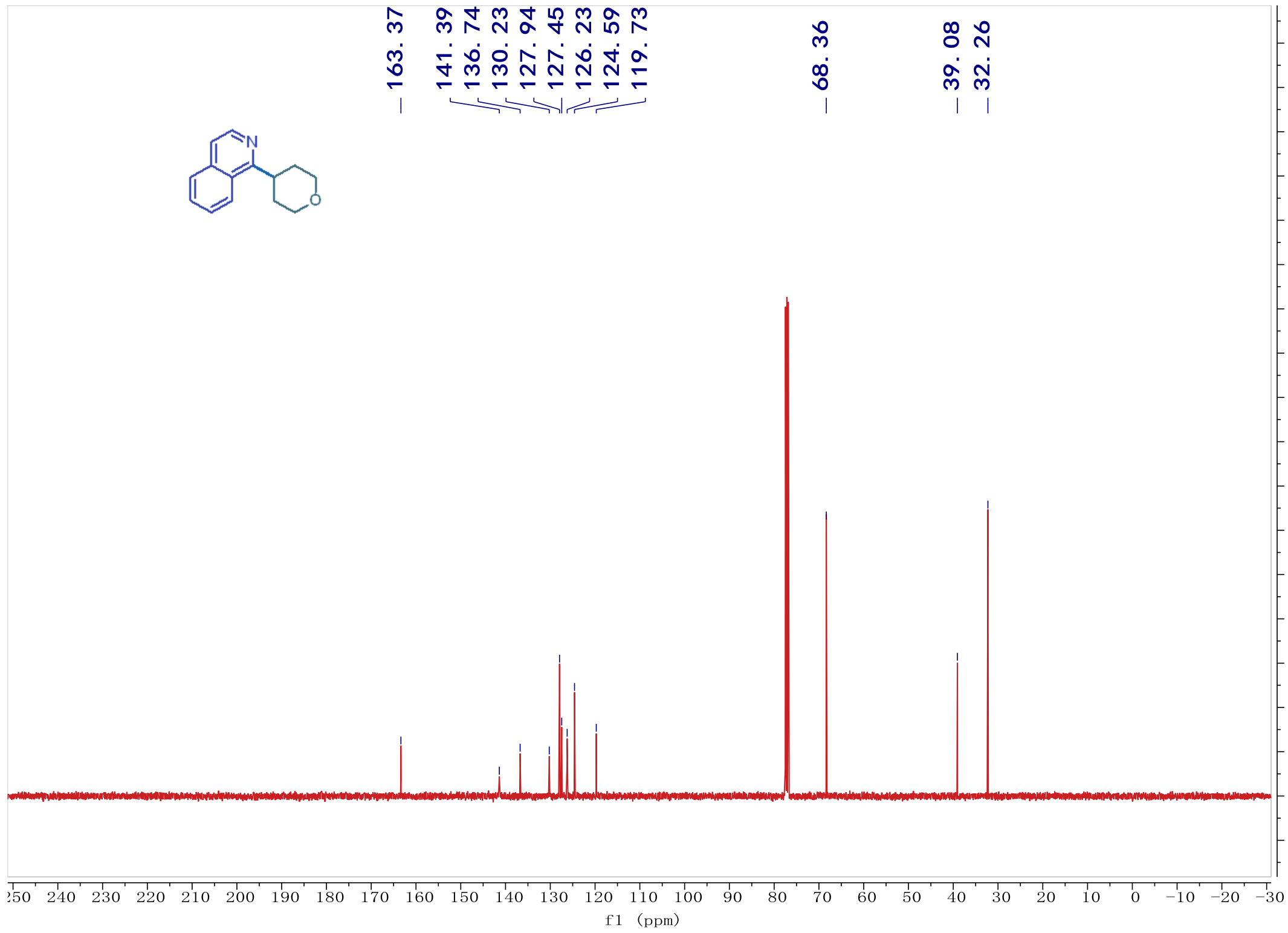

^{1}H NMR spectrum of **4-14** (400 MHz, $CDCl_3$).

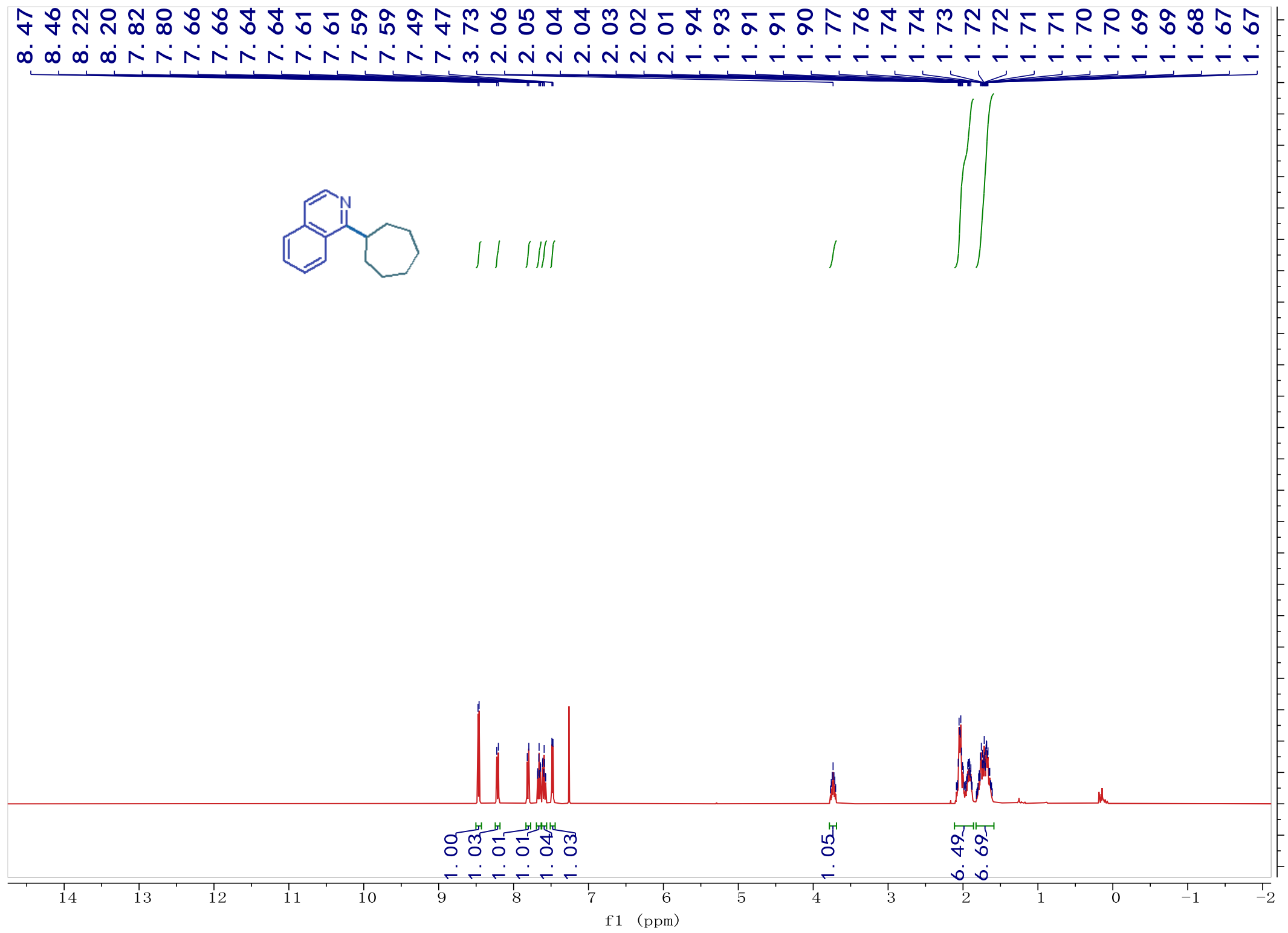

$^{13}C\{^{1}H\}$ NMR spectrum of **4-14** (101 MHz, $CDCl_3$).

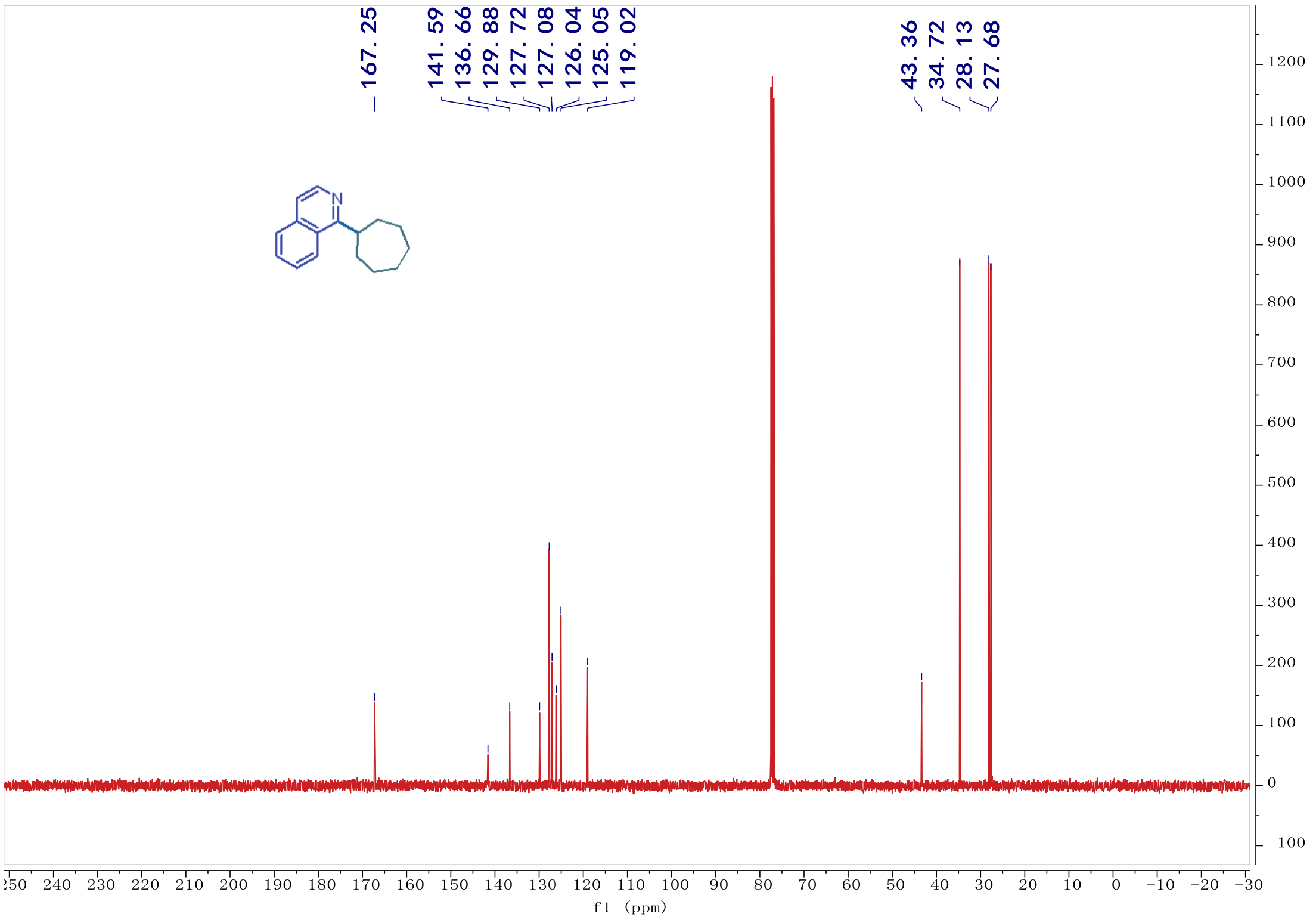

^{1}H NMR spectrum of **4-15** (400 MHz, $CDCl_3$).

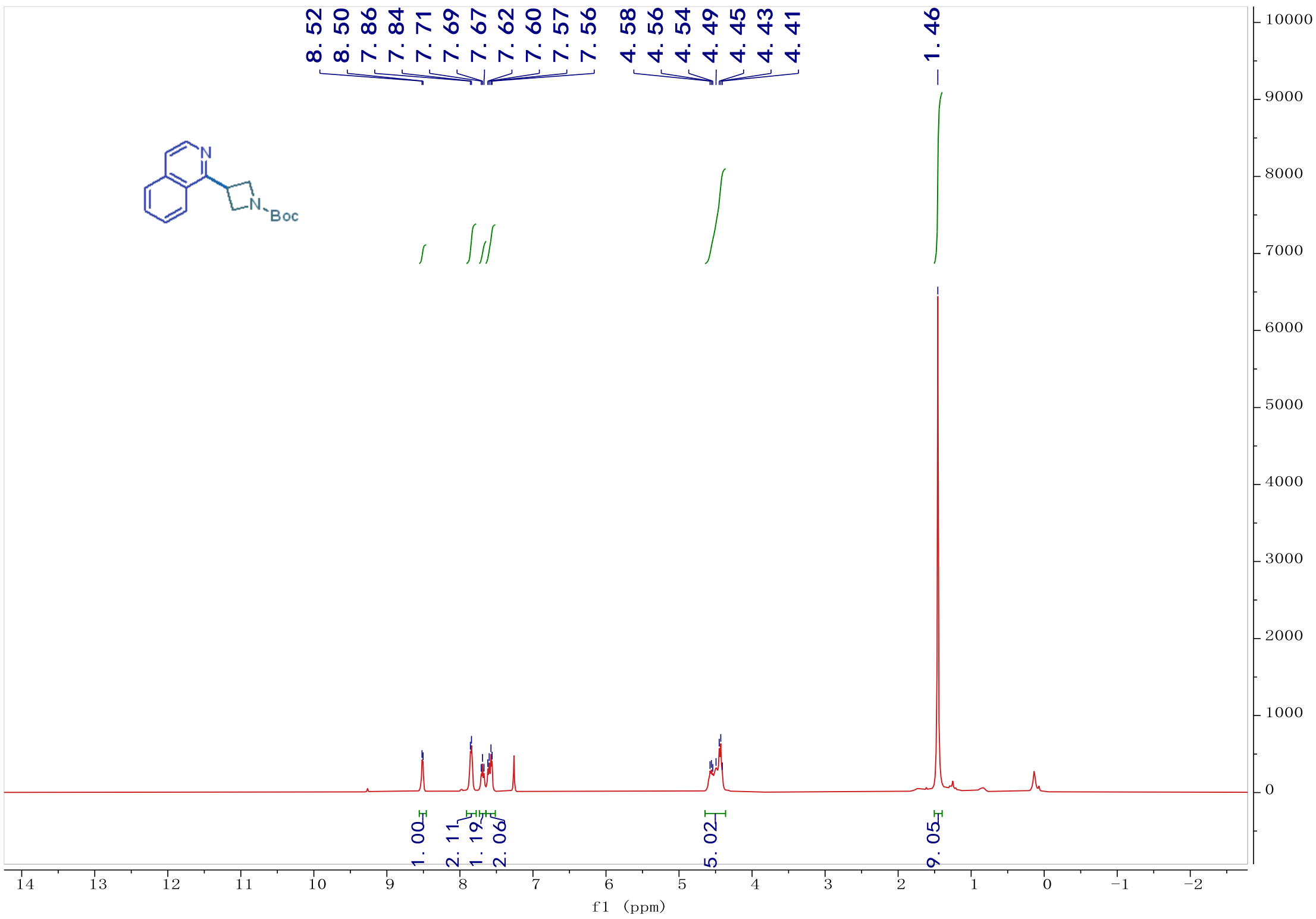

$^{13}C\{^{1}H\}$ NMR spectrum of **4-15** (101 MHz, $CDCl_3$).

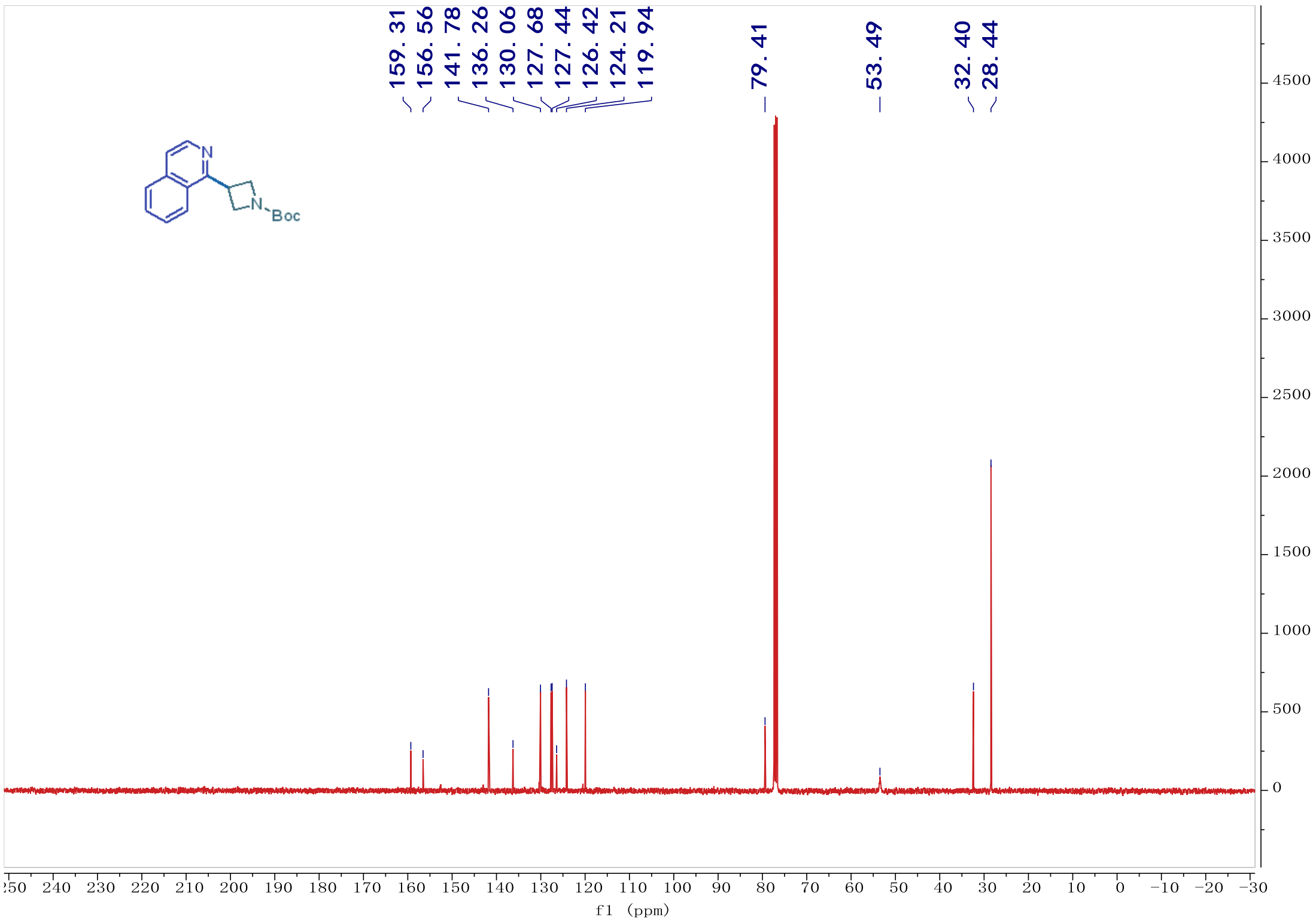

^{1}H NMR spectrum of **4-16** (400 MHz, DMSO-d_6).

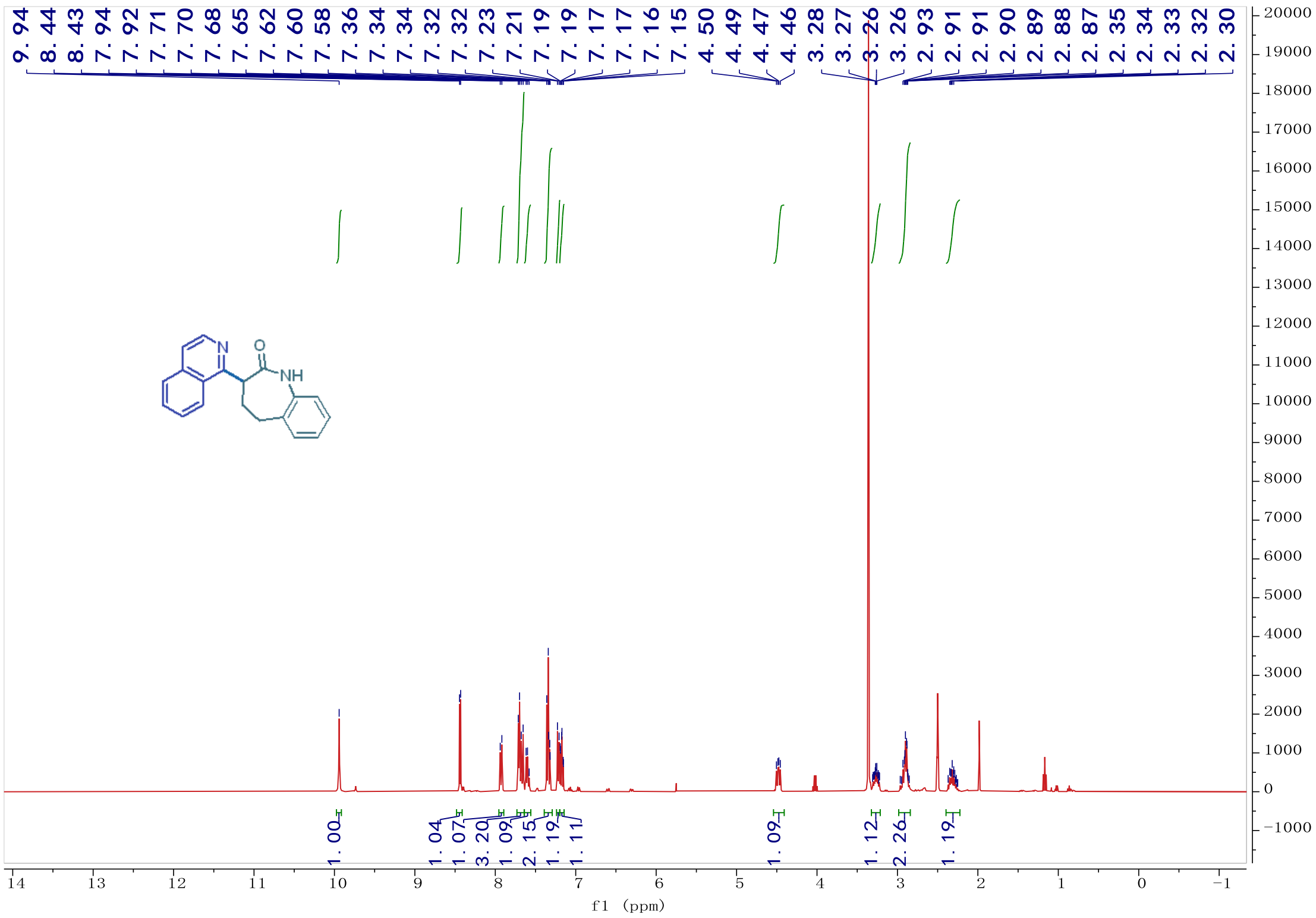

$^{13}C\{^{1}H\}$ NMR spectrum of **4-16** (101 MHz, DMSO-d$_6$).

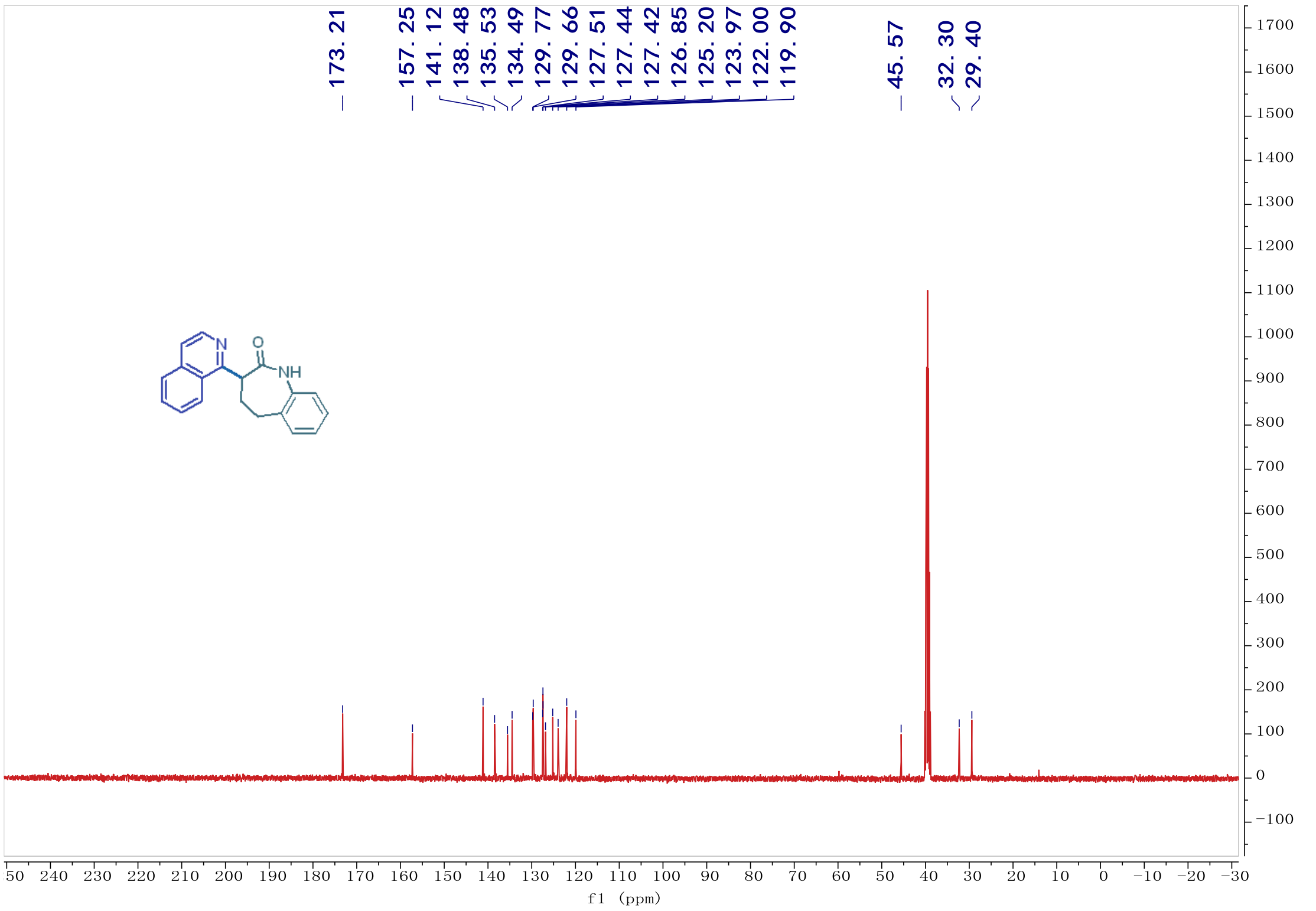

^{1}H NMR spectrum of **4-17** (400 MHz, $CDCl_3$).

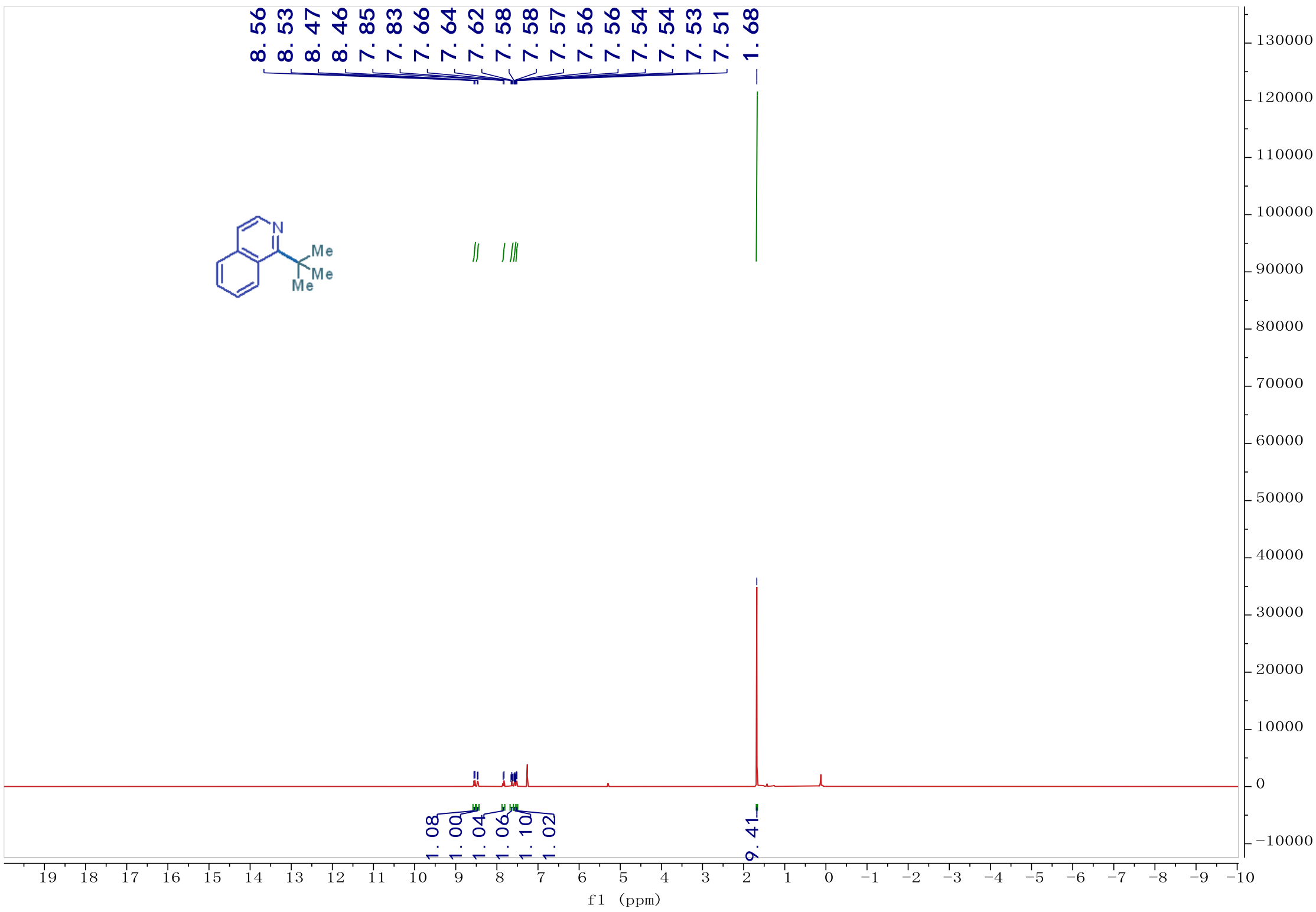

$^{13}C\{^1H\}$ NMR spectrum of **4-17** (101 MHz, $CDCl_3$).

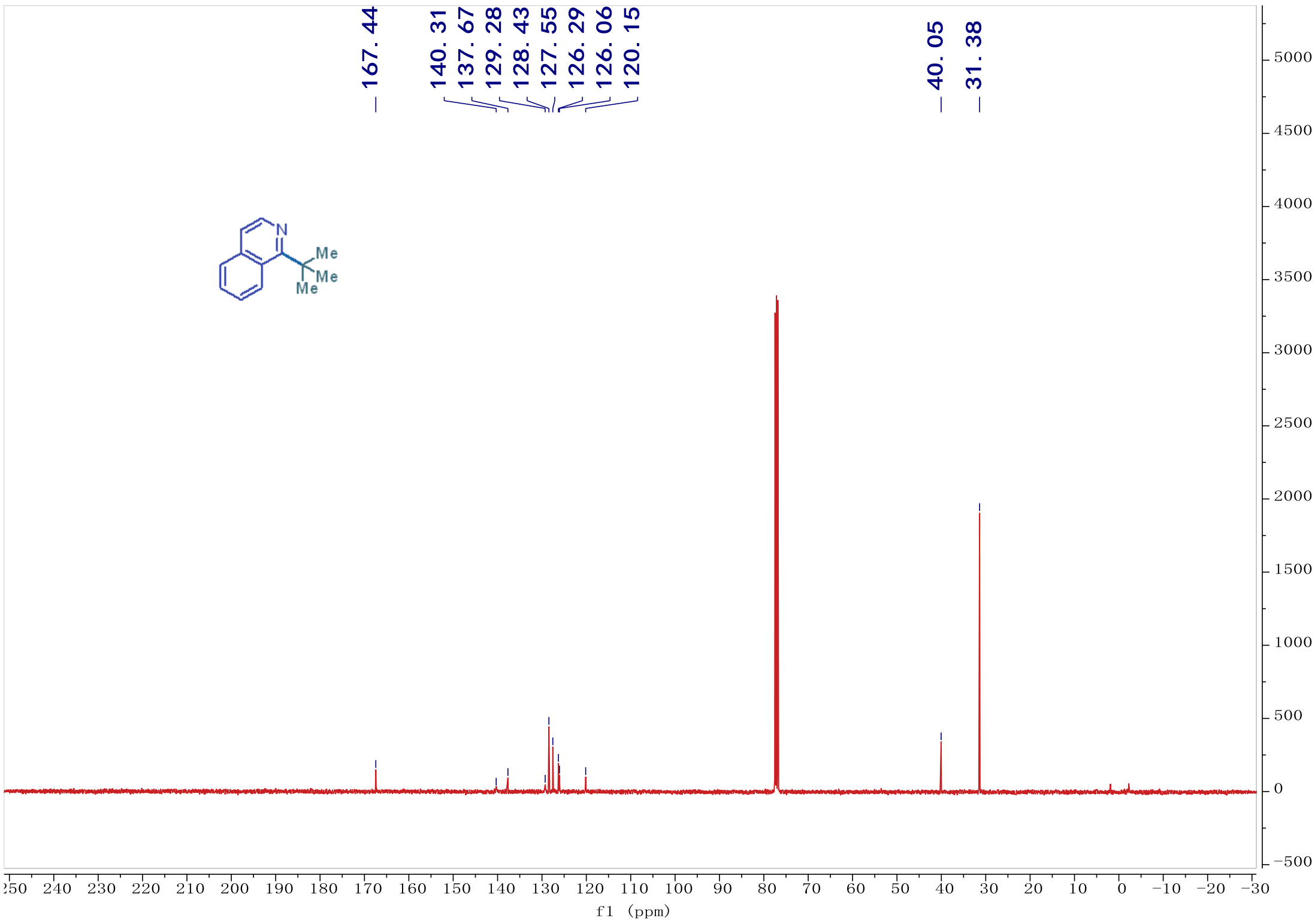

^1H NMR spectrum of **4-18** (400 MHz, $CDCl_3$).

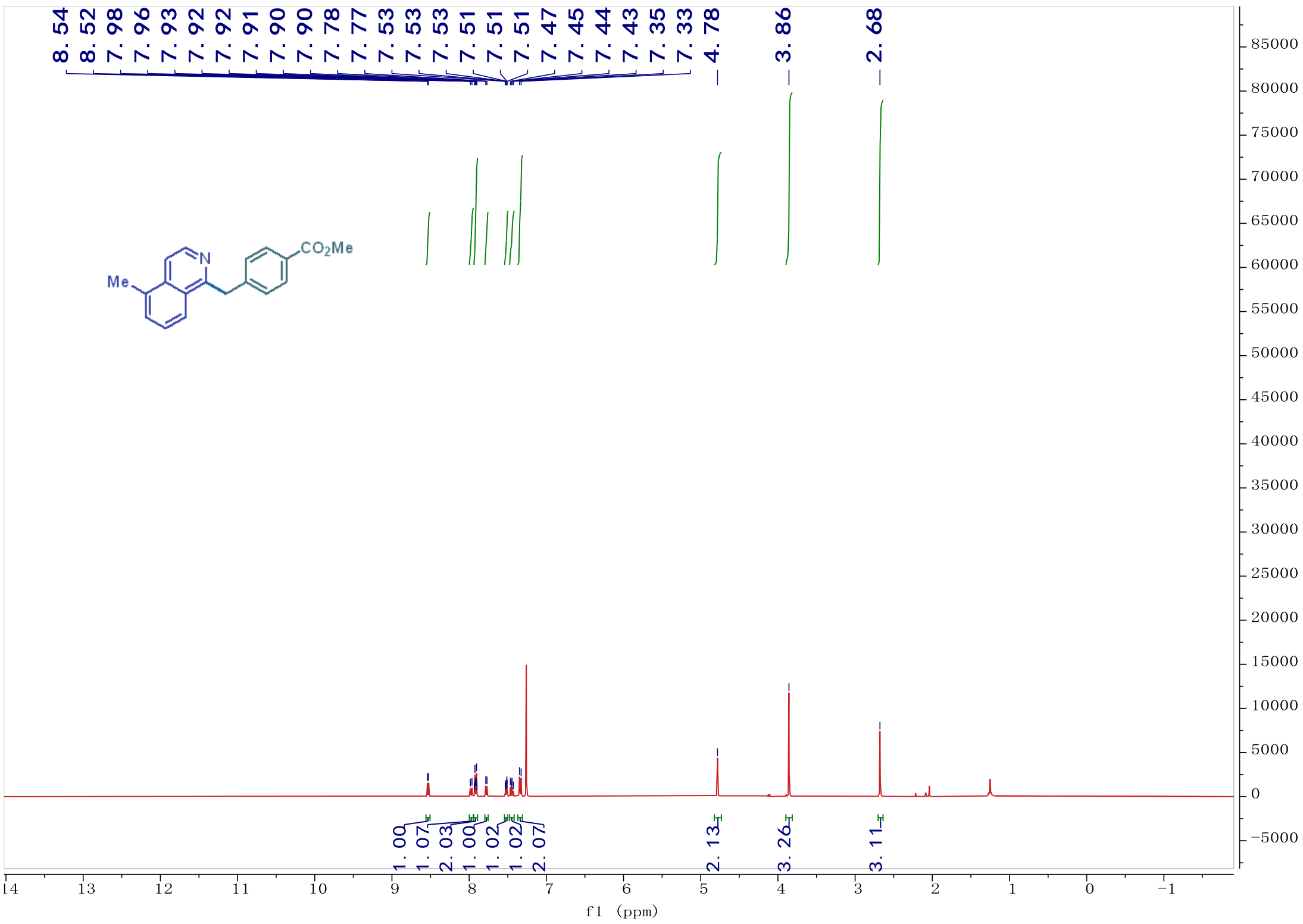

$^{13}C\{^{1}H\}$ NMR spectrum of **4-18** (101 MHz, $CDCl_3$).

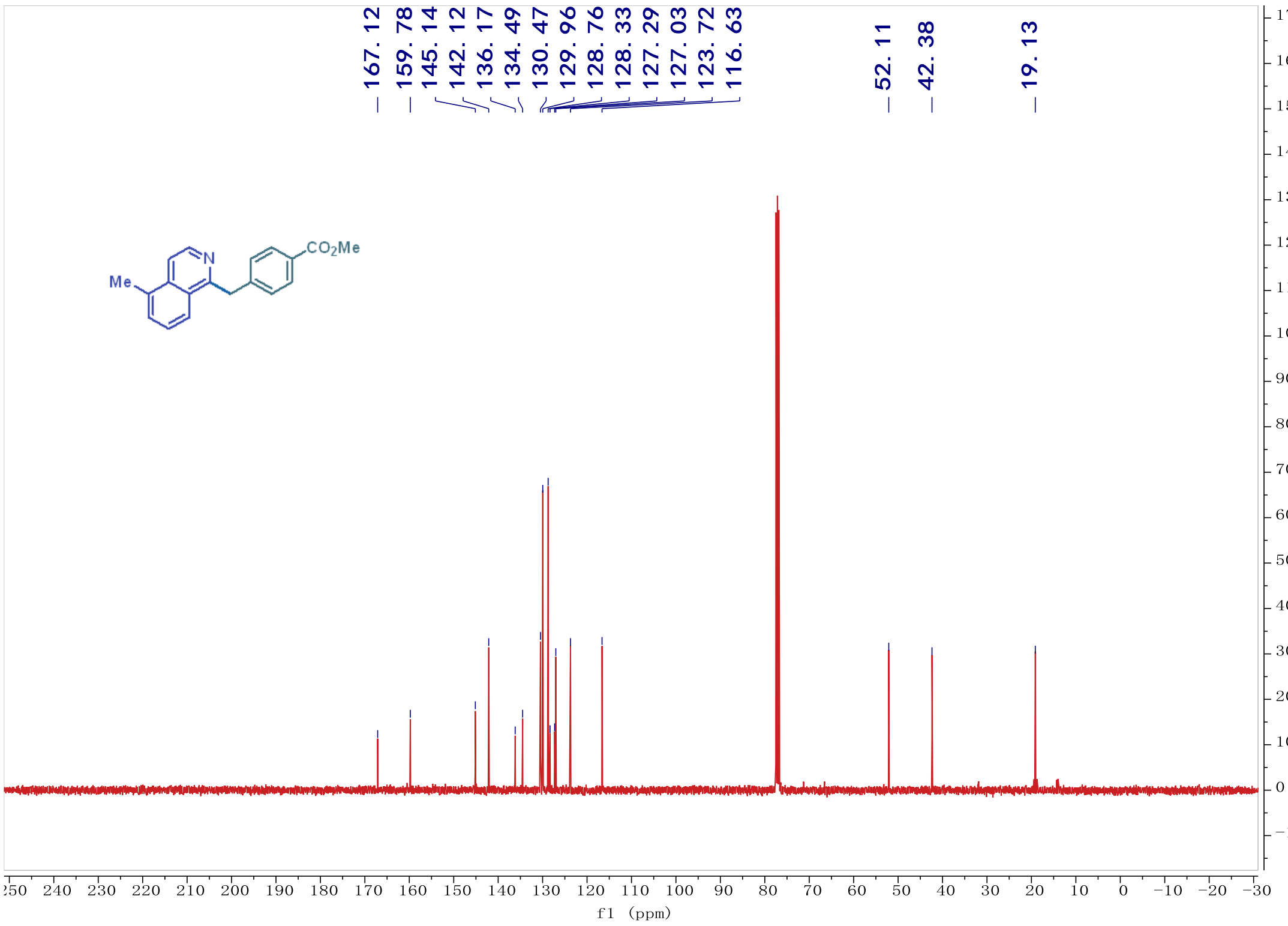

^{1}H NMR spectrum of **4-19** (400 MHz, $CDCl_3$).

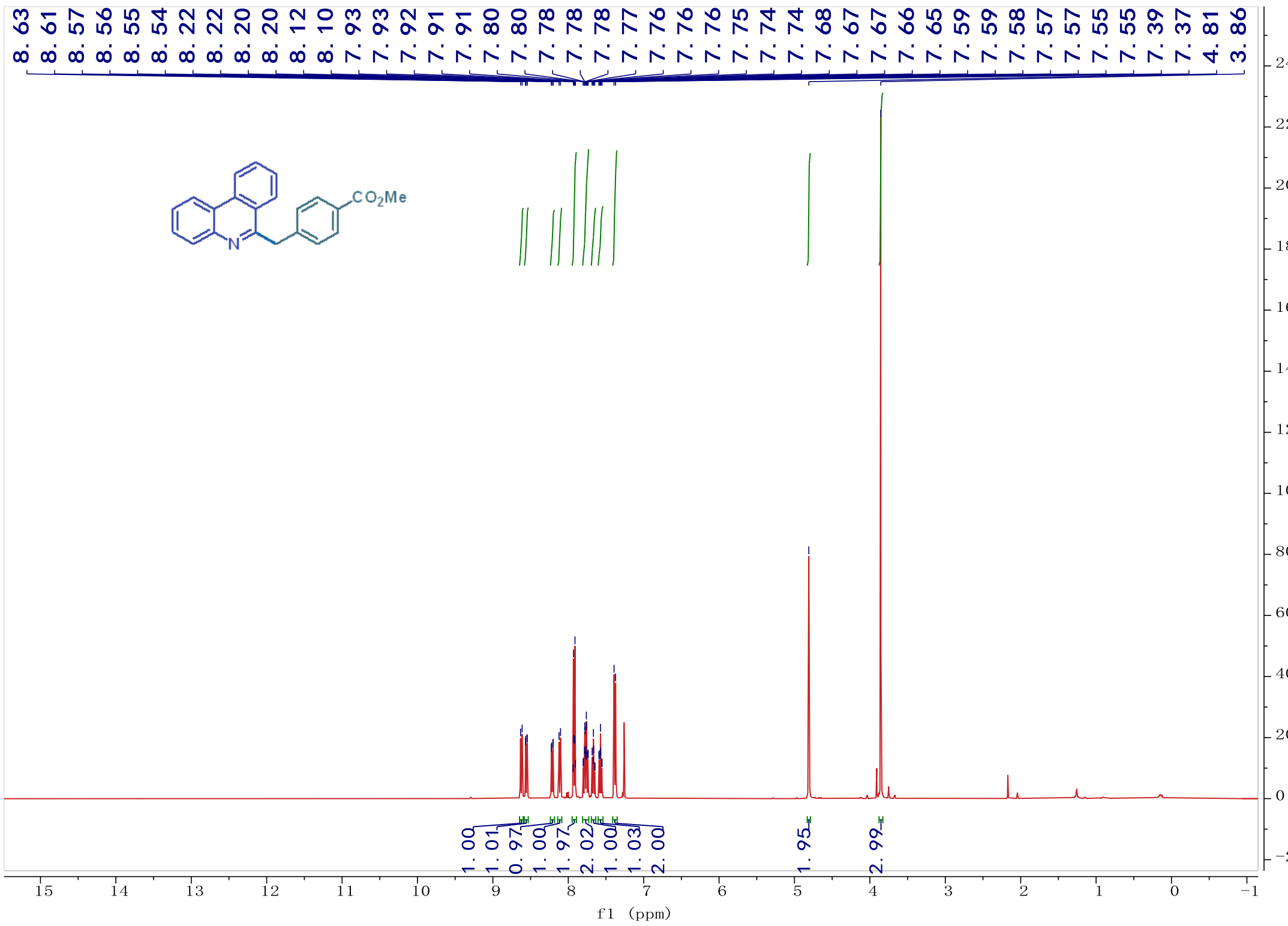

$^{13}C\{^1H\}$ NMR spectrum of **4-19** (101 MHz, $CDCl_3$).

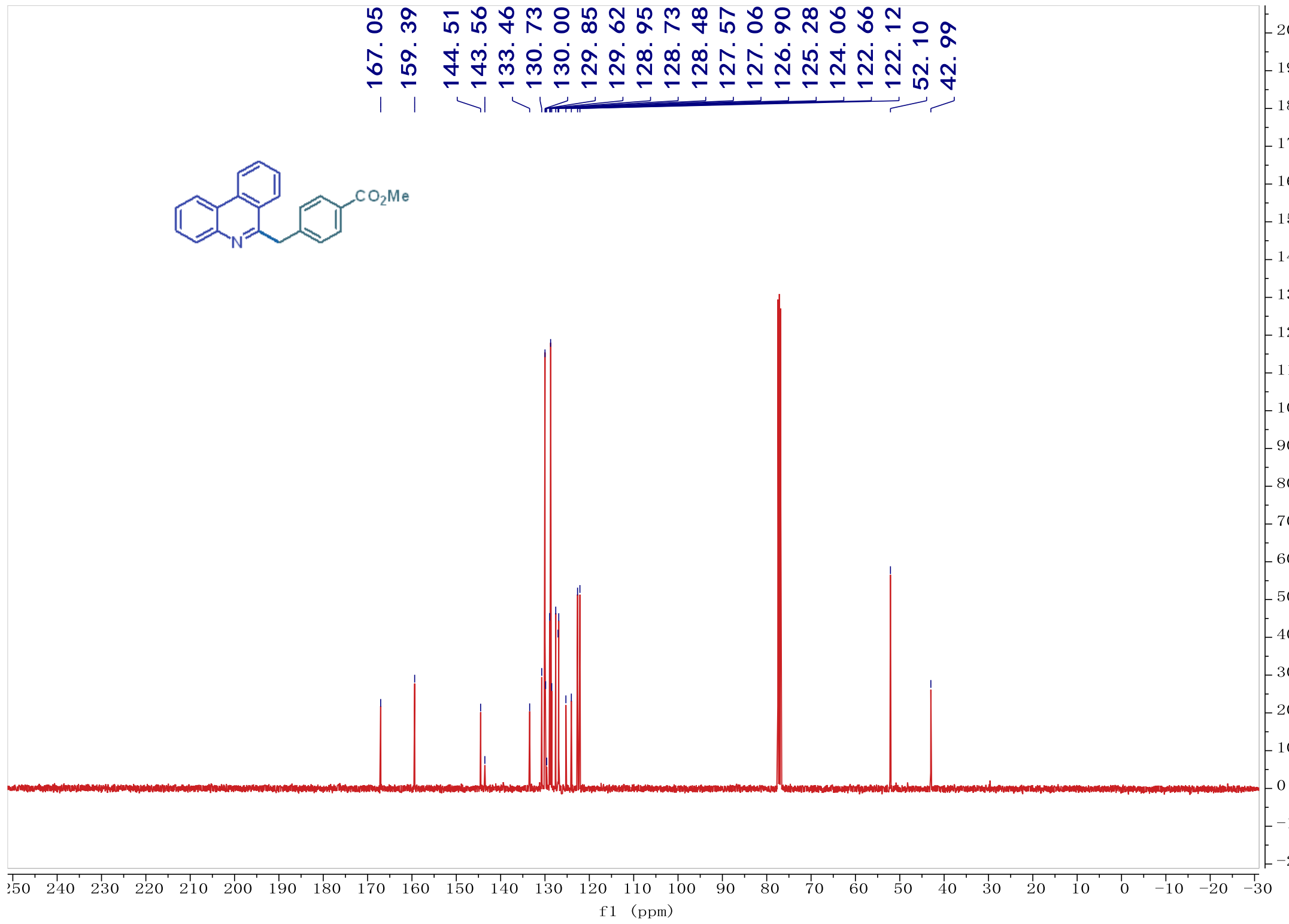

^1H NMR spectrum of **4-20** (400 MHz, $CDCl_3$).

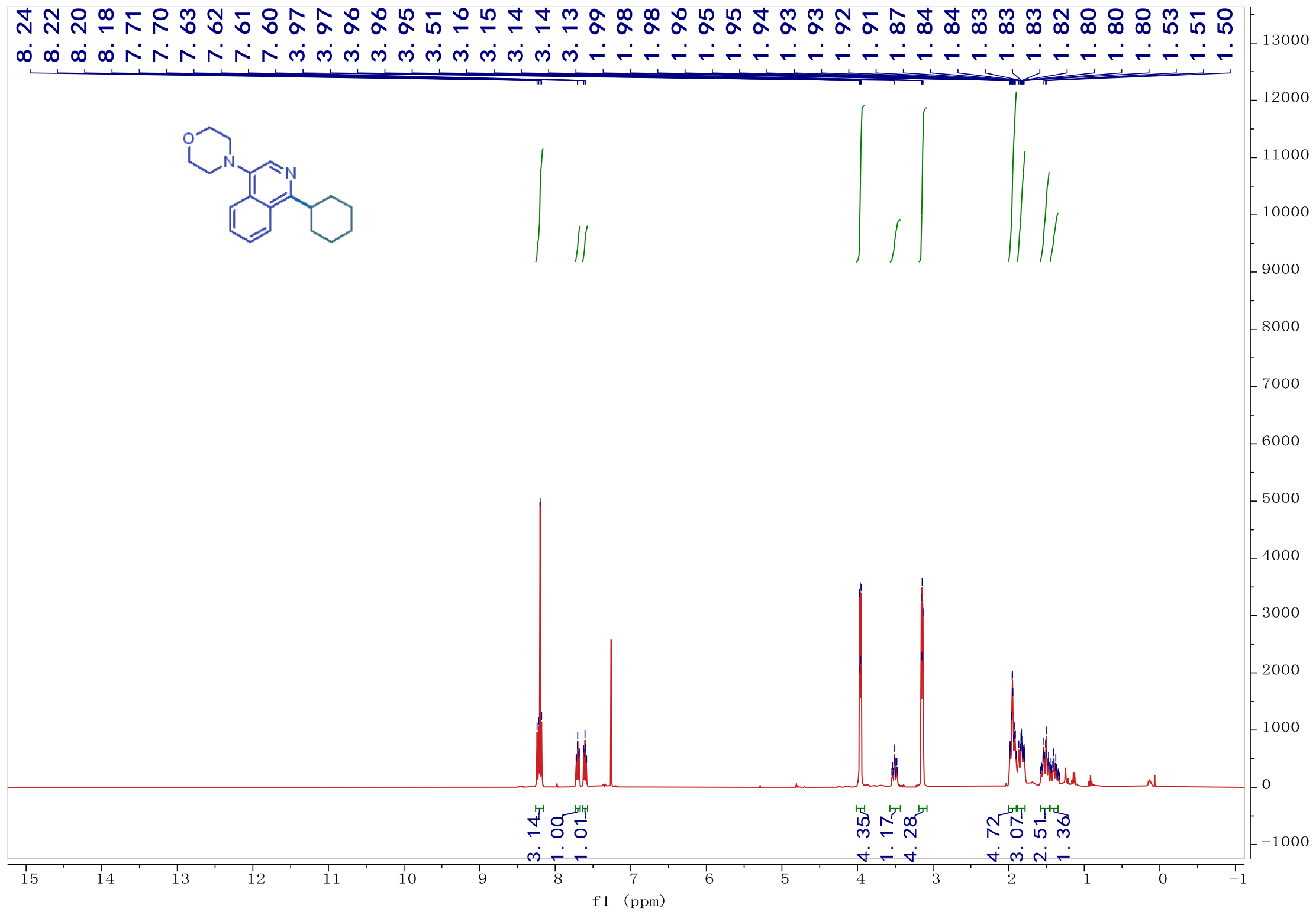

$^{13}C\{^{1}H\}$ NMR spectrum of **4-20** (101 MHz, $CDCl_3$).

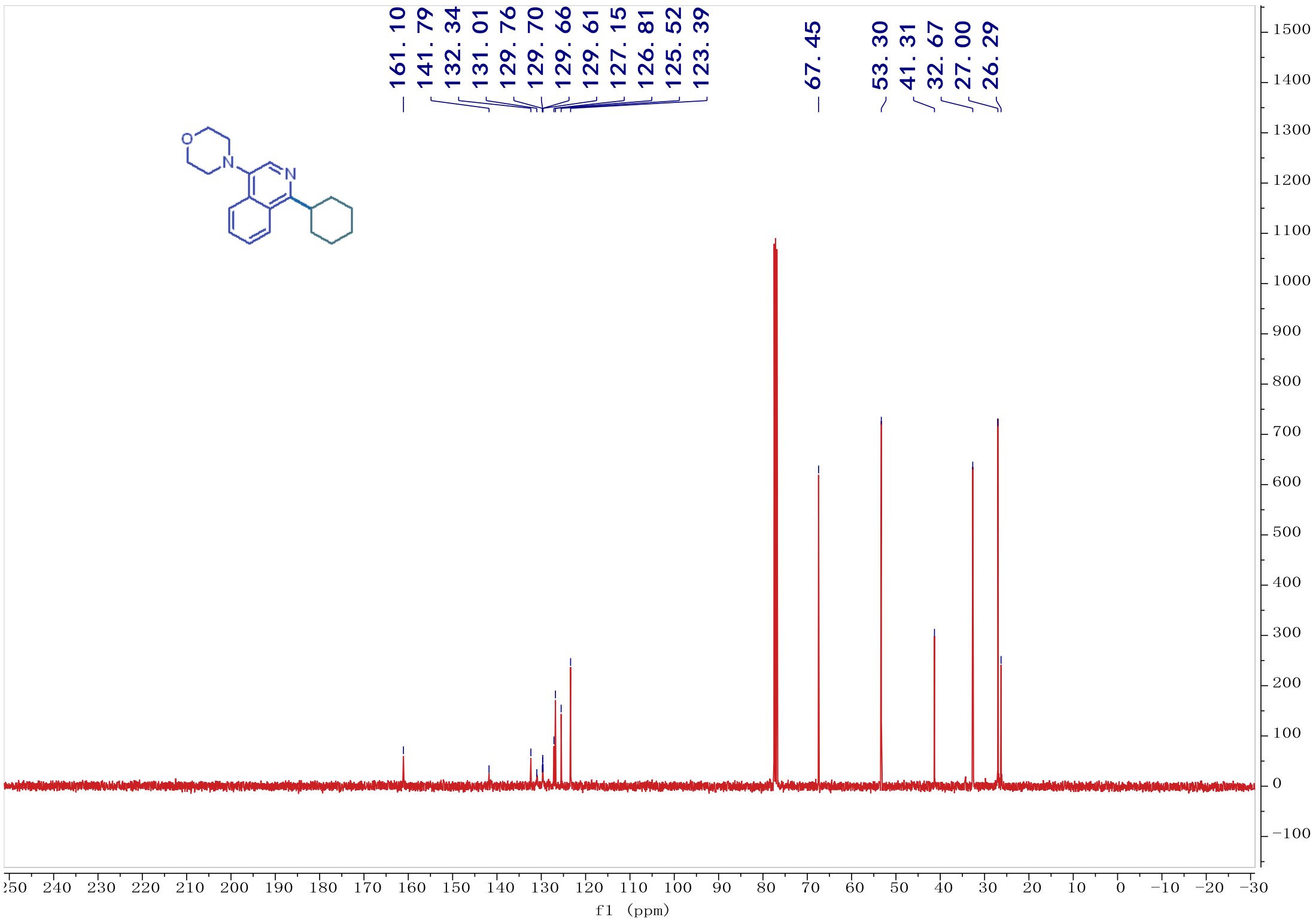

^{1}H NMR spectrum of **4-21** (400 MHz, $CDCl_3$).

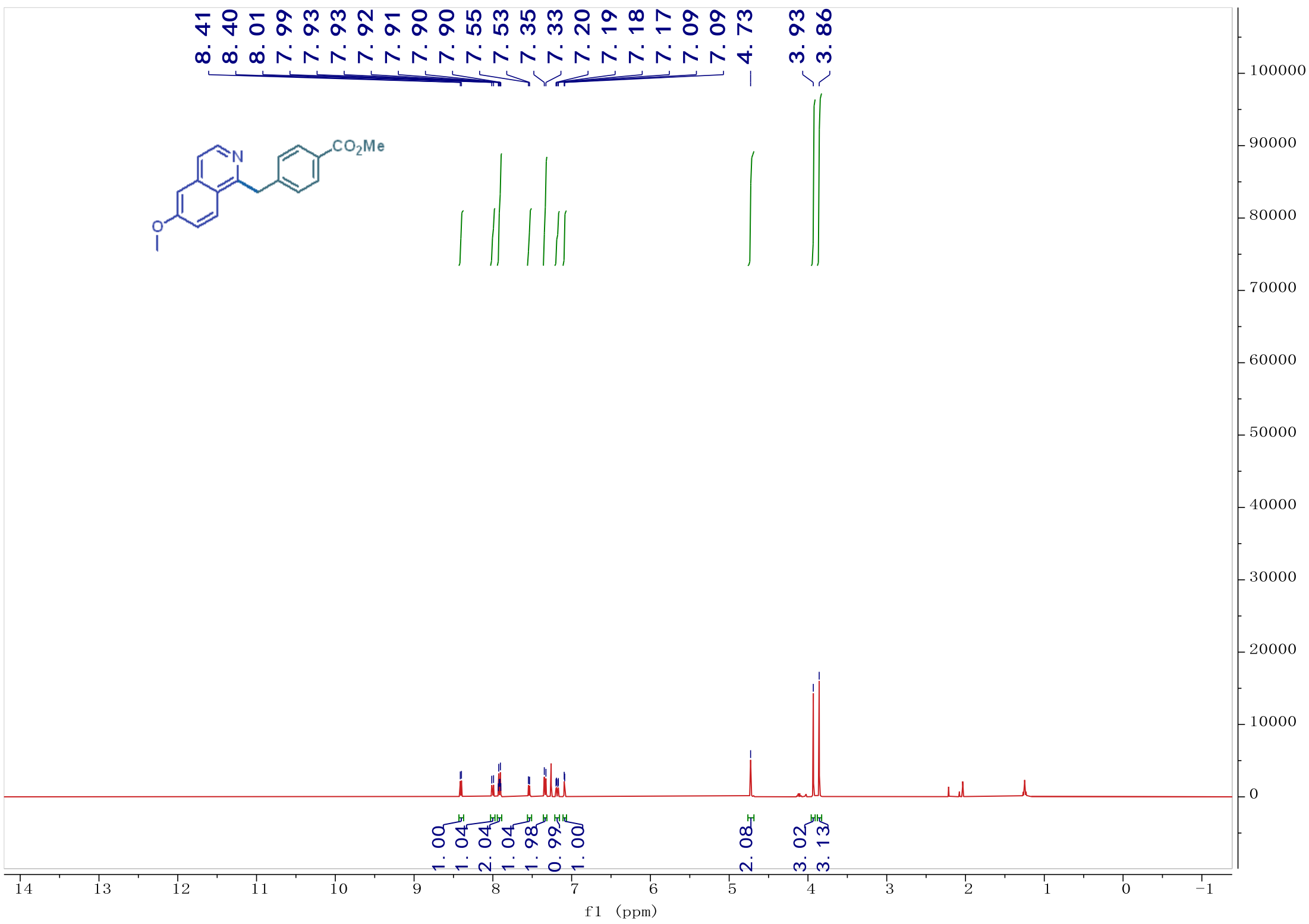

$^{13}C\{^{1}H\}$ NMR spectrum of **4-21** (101 MHz, $CDCl_3$).

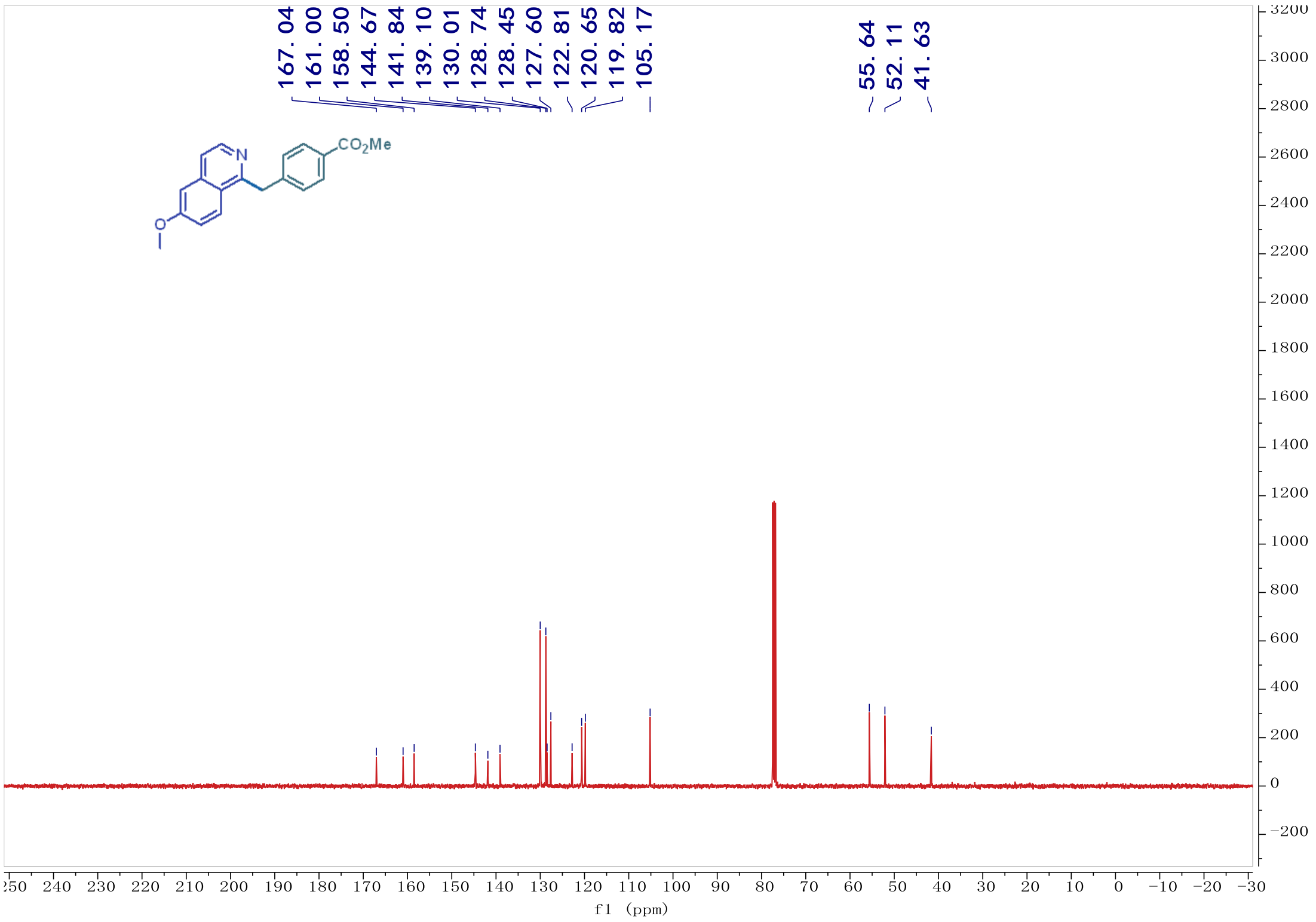

^{1}H NMR spectrum of **4-22** (400 MHz, $CDCl_3$).

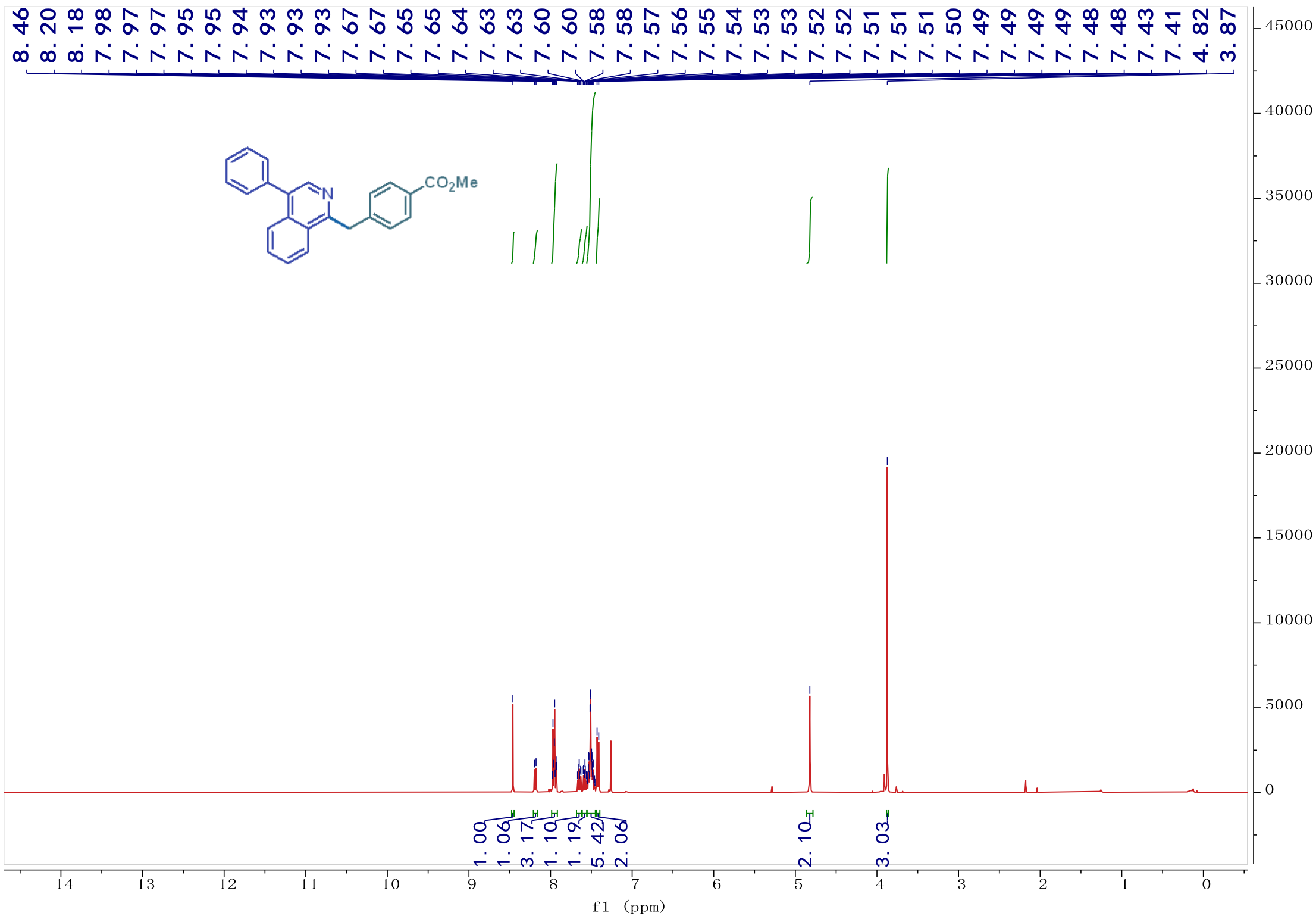

$^{13}C\{^{1}H\}$ NMR spectrum of **4-22** (101 MHz, $CDCl_3$).

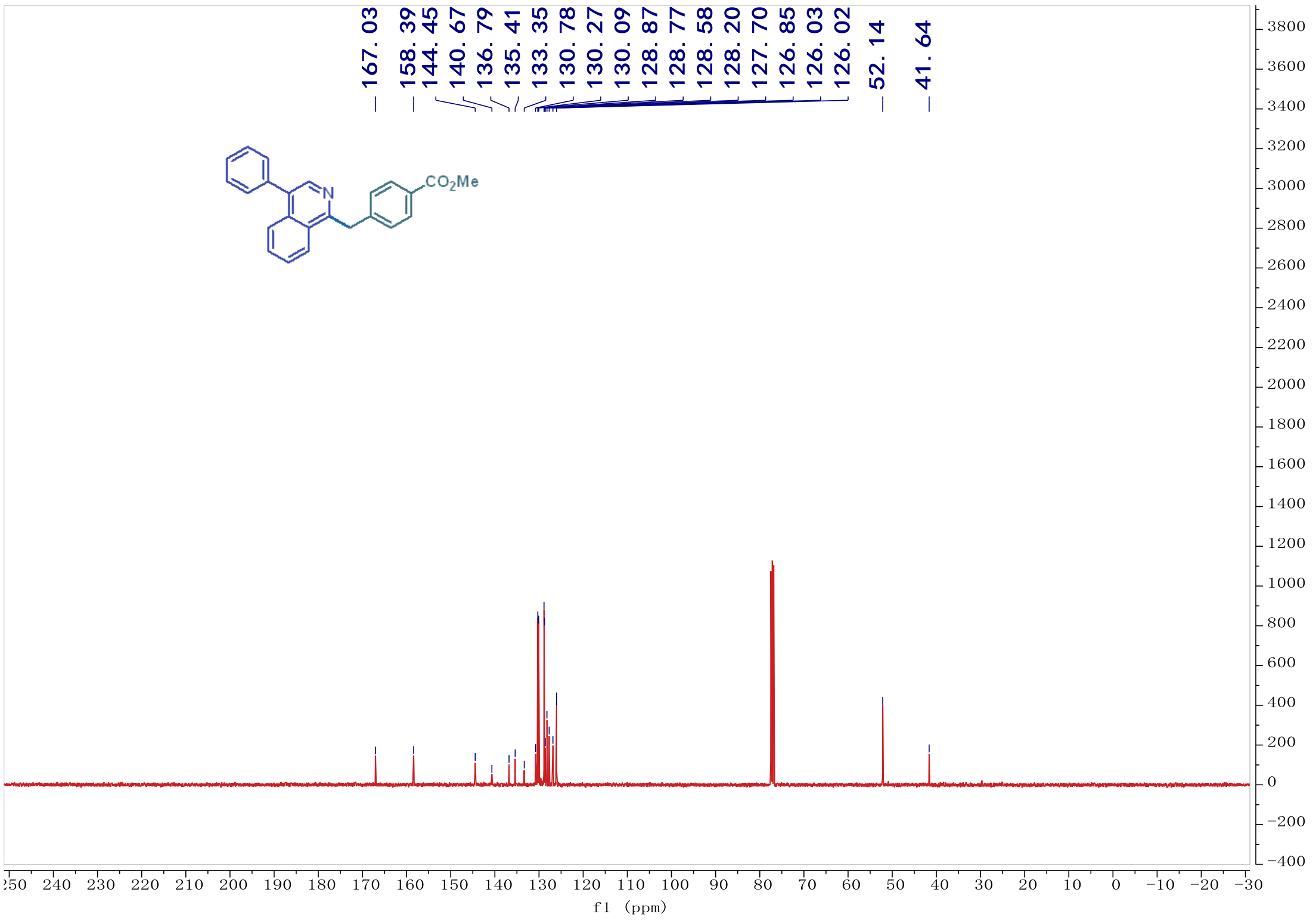

^{1}H NMR spectrum of **4-23** (400 MHz, $CDCl_3$).

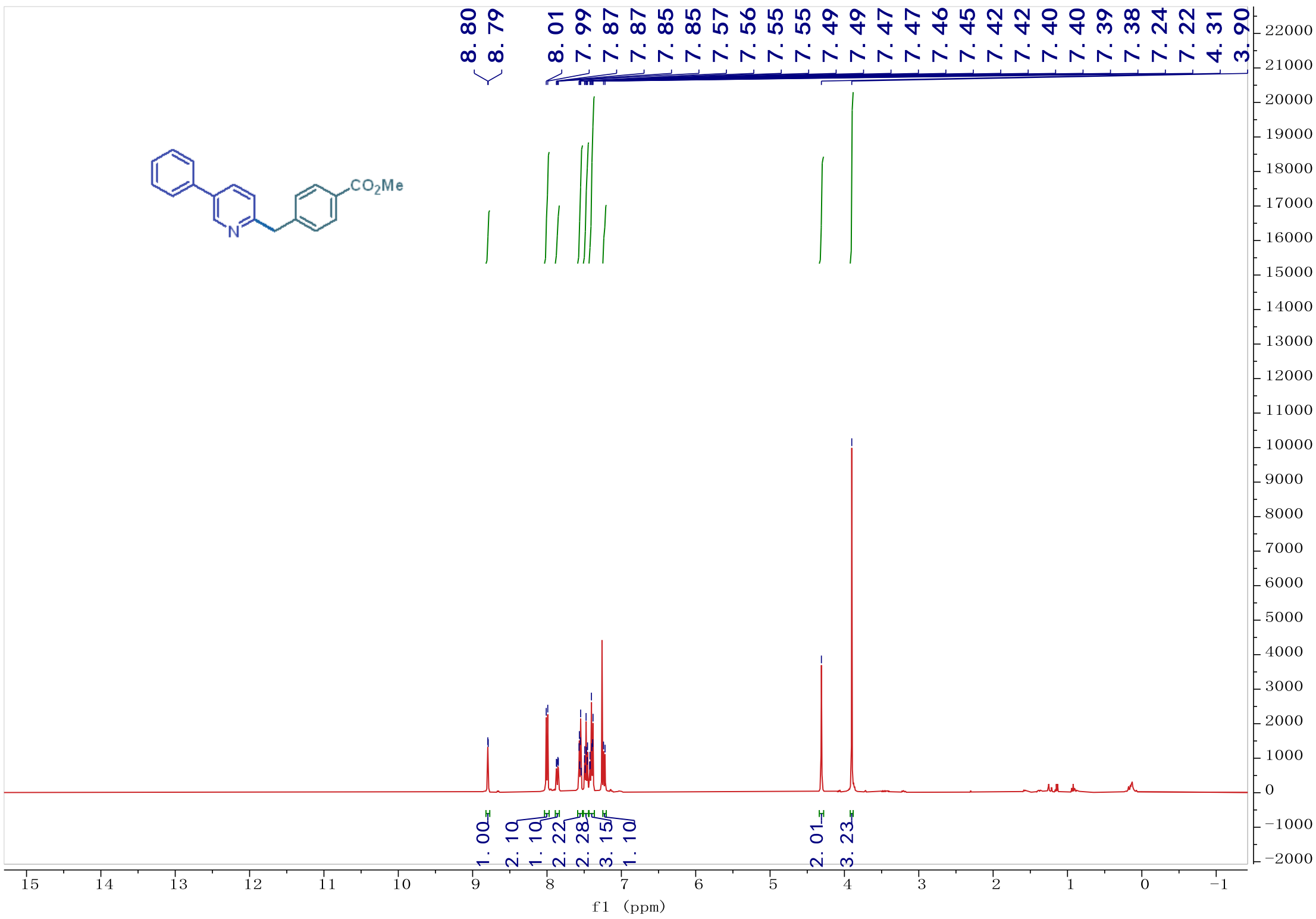

$^{13}C\{^1H\}$ NMR spectrum of **4-23** (101 MHz, $CDCl_3$).

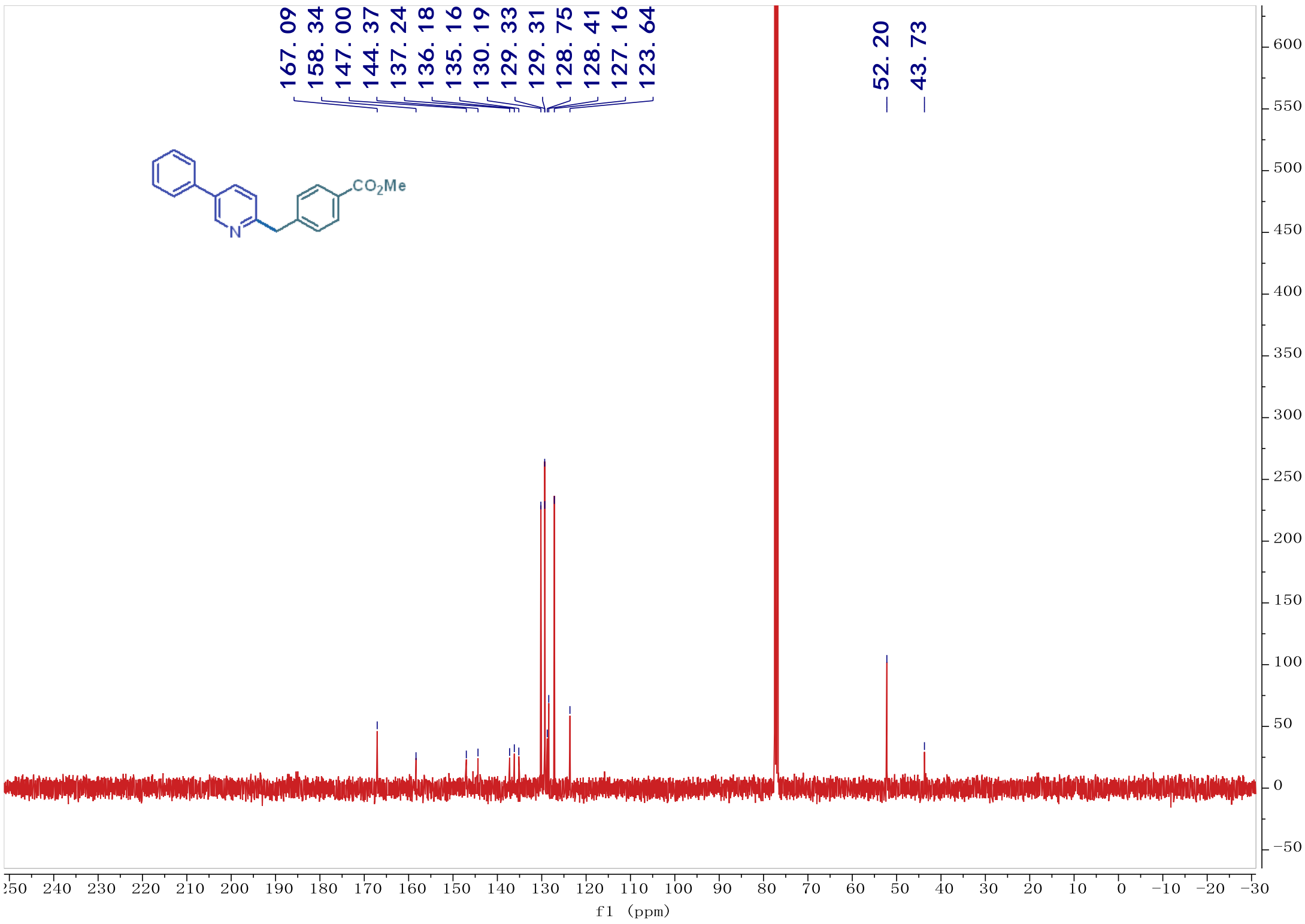

^1H NMR spectrum of **4-24** (400 MHz, $CDCl_3$).

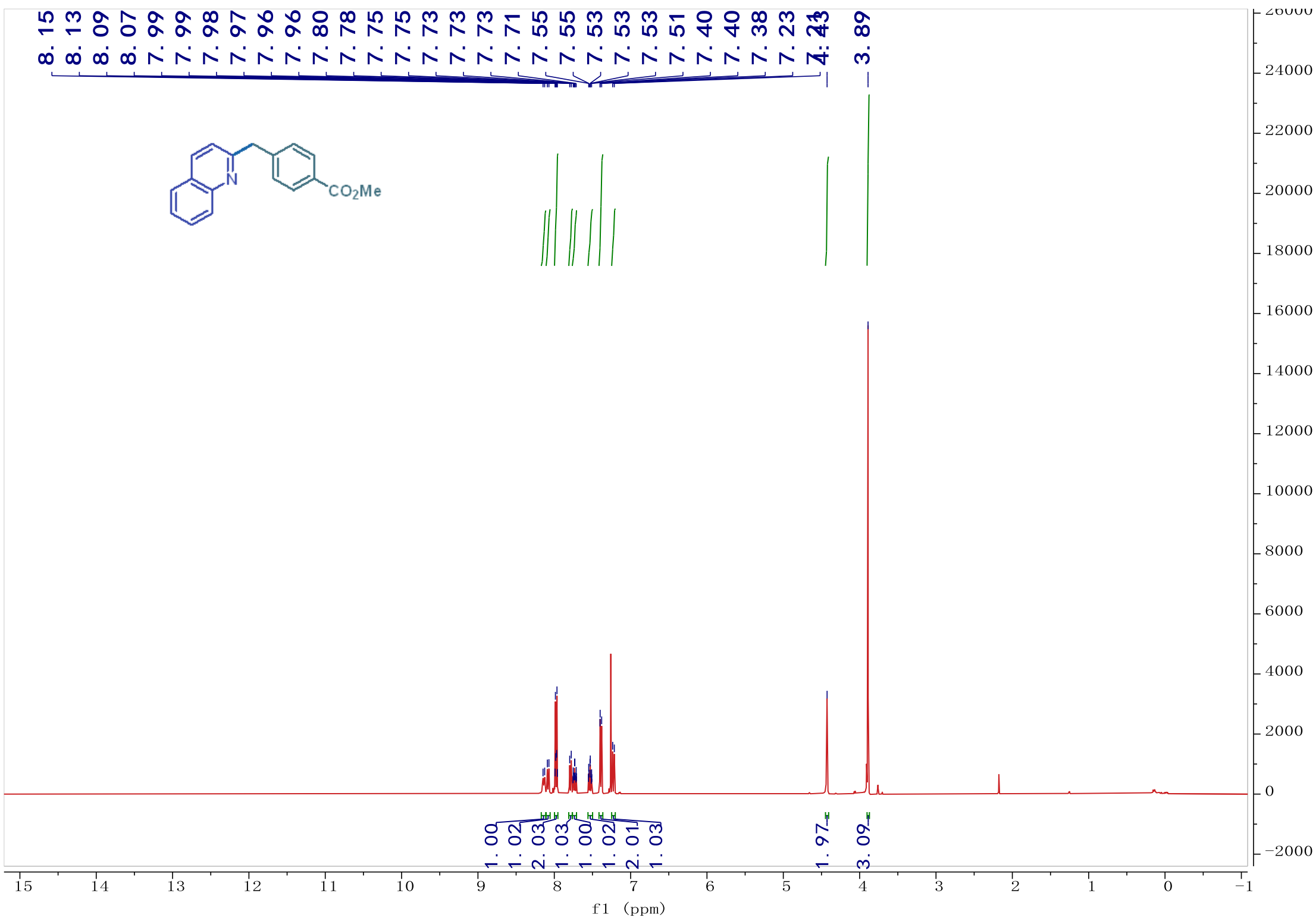

$^{13}C\{^{1}H\}$ NMR spectrum of **4-24** (101 MHz, $CDCl_3$).

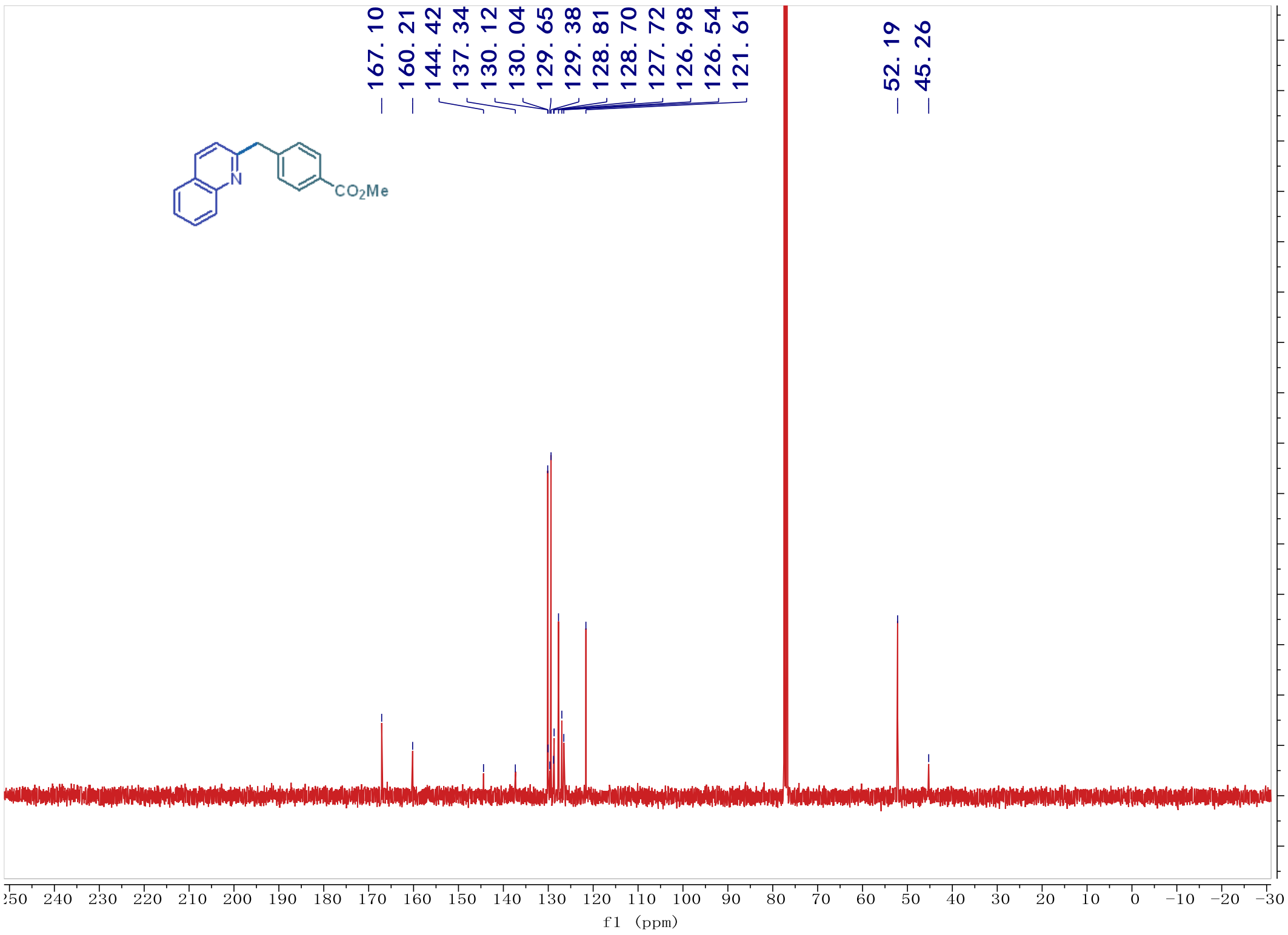

^{1}H NMR spectrum of **4-25** (400 MHz, $CDCl_3$).

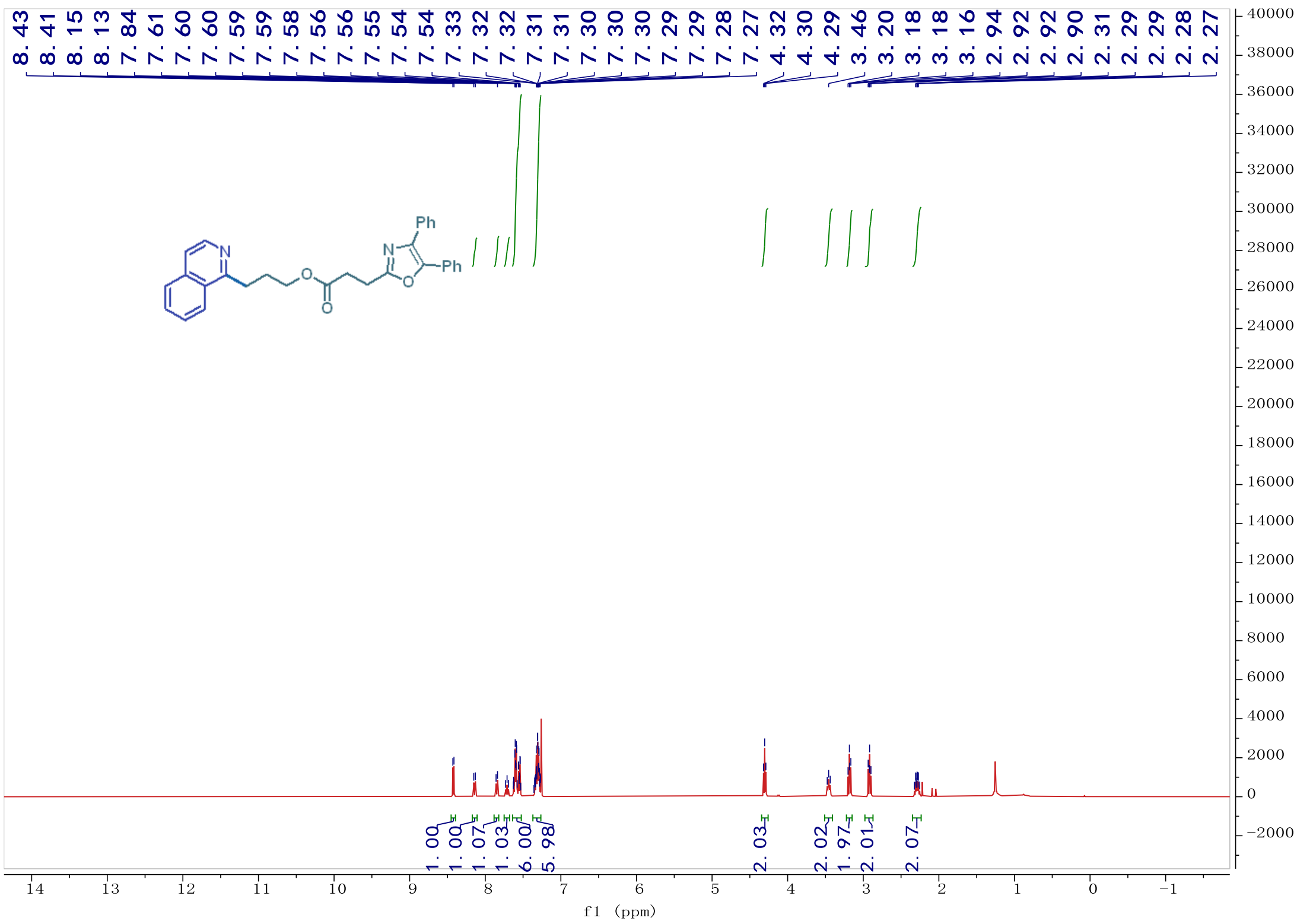

$^{13}C\{^{1}H\}$ NMR spectrum of **4-25** (101 MHz, $CDCl_3$).

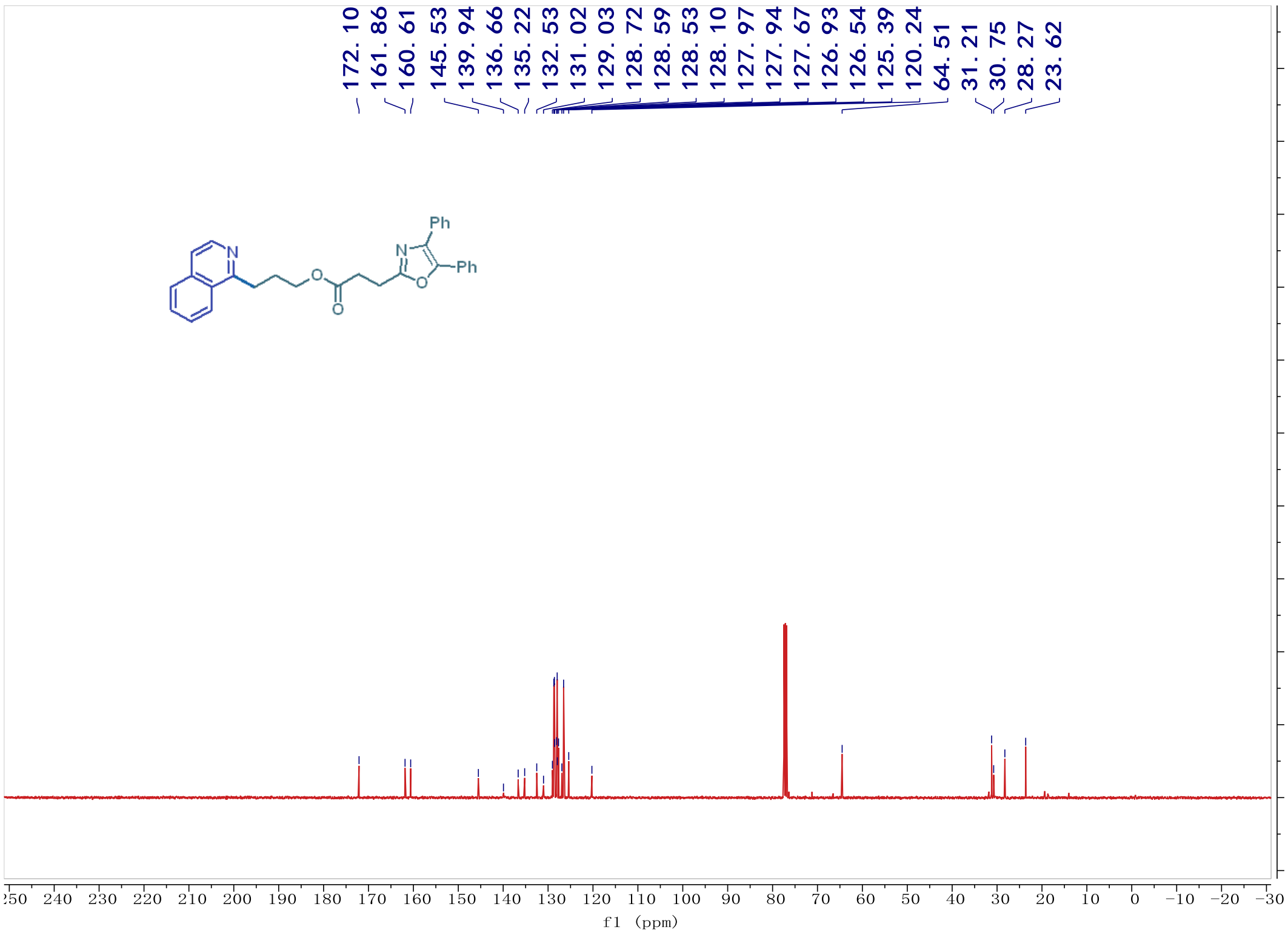

^{1}H NMR spectrum of **4-26** (400 MHz, $CDCl_3$).

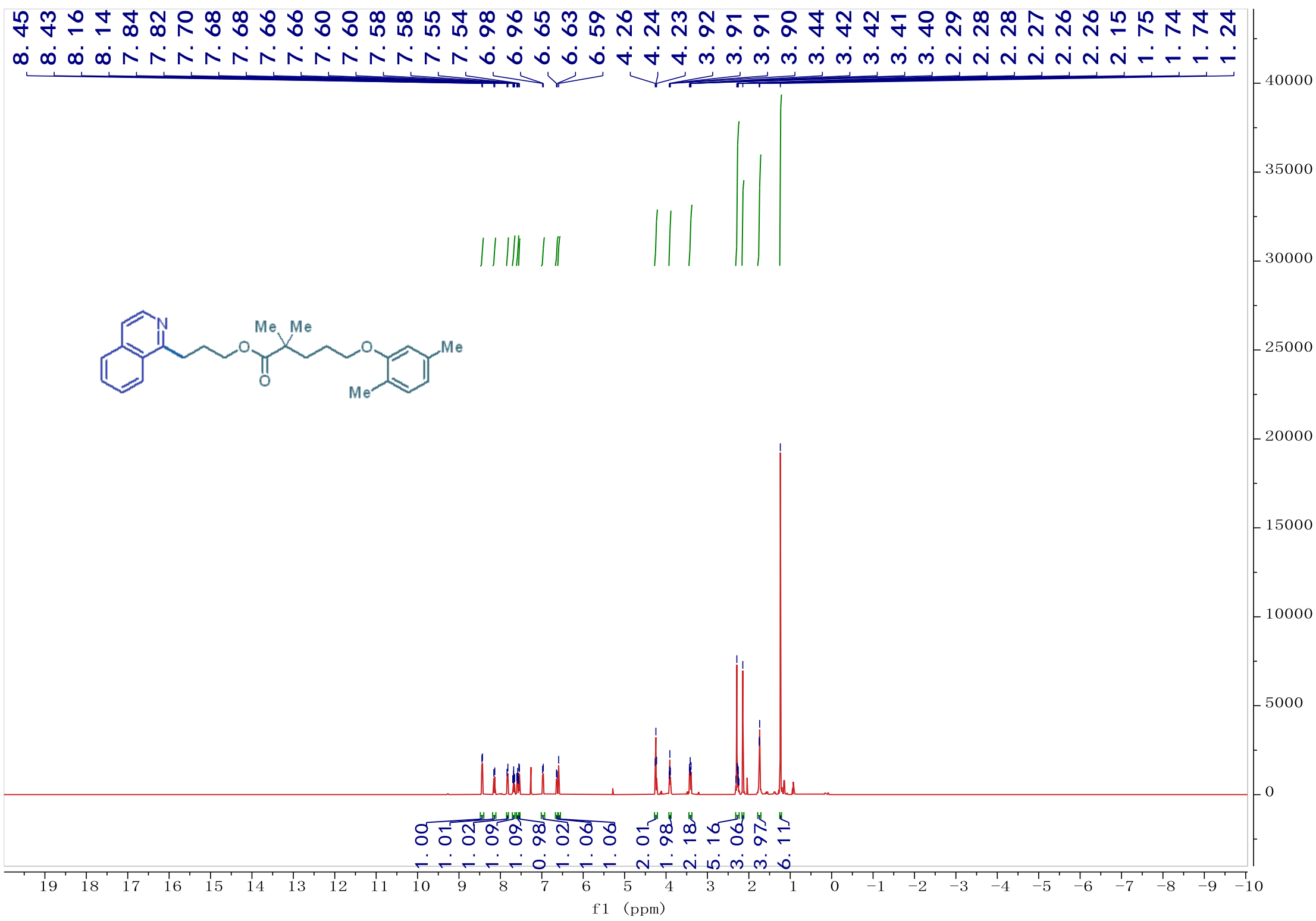

$^{13}C\{^{1}H\}$ NMR spectrum of **4-26** (101 MHz, $CDCl_3$).

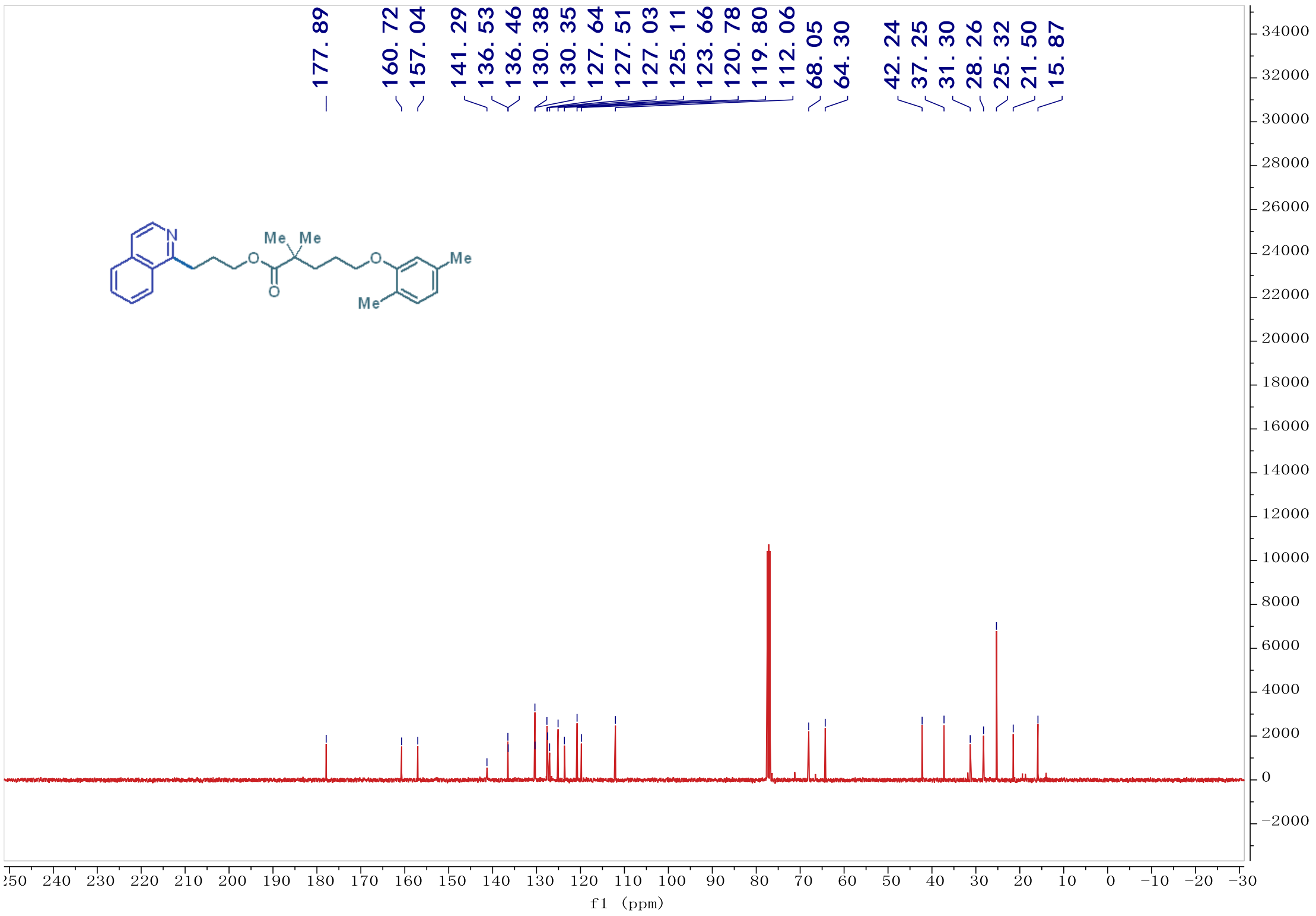

^{1}H NMR spectrum of **4-27** (400 MHz, $CDCl_3$).

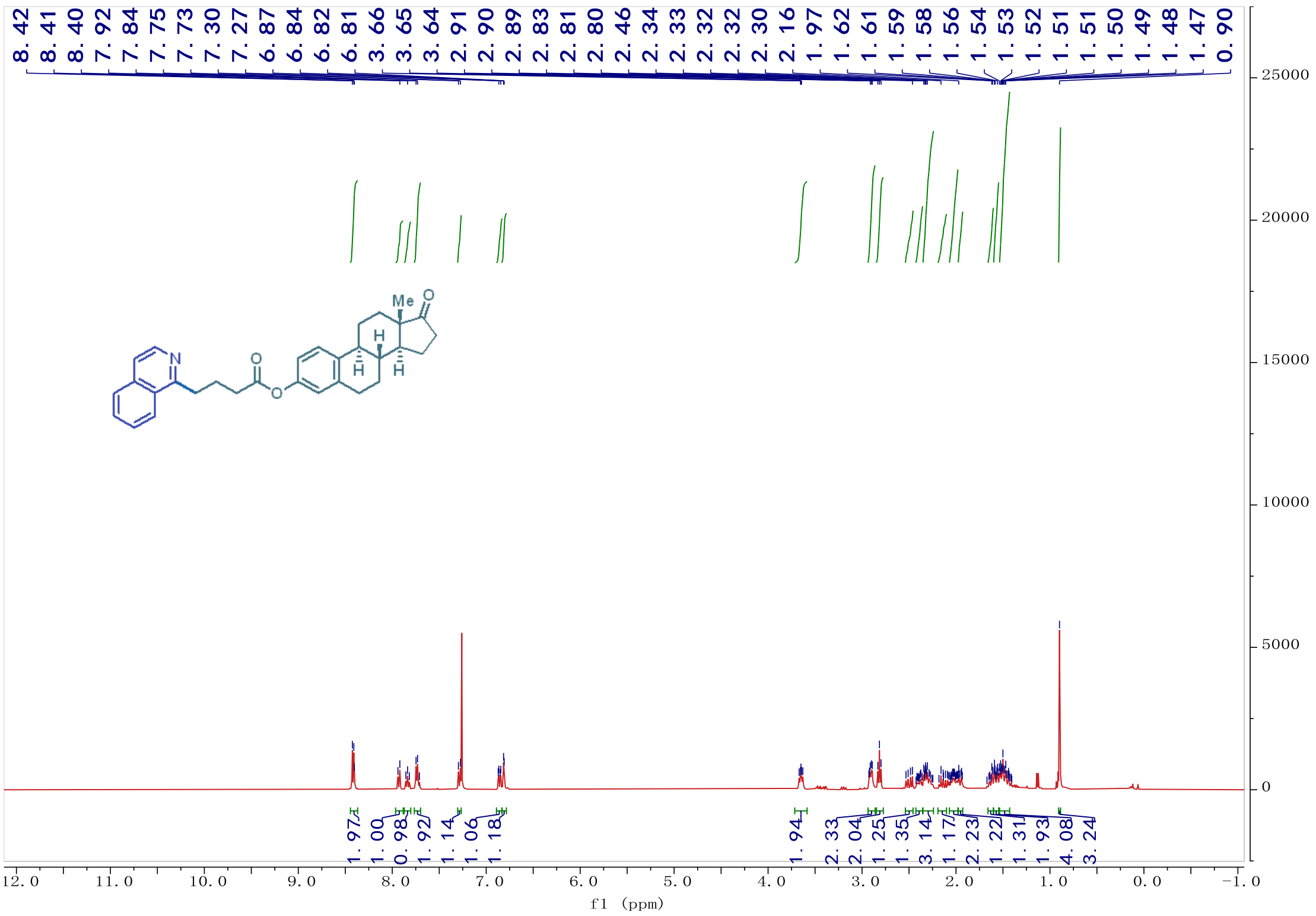

$^{13}C\{^{1}H\}$ NMR spectrum of **4-27** (101 MHz, $CDCl_3$).

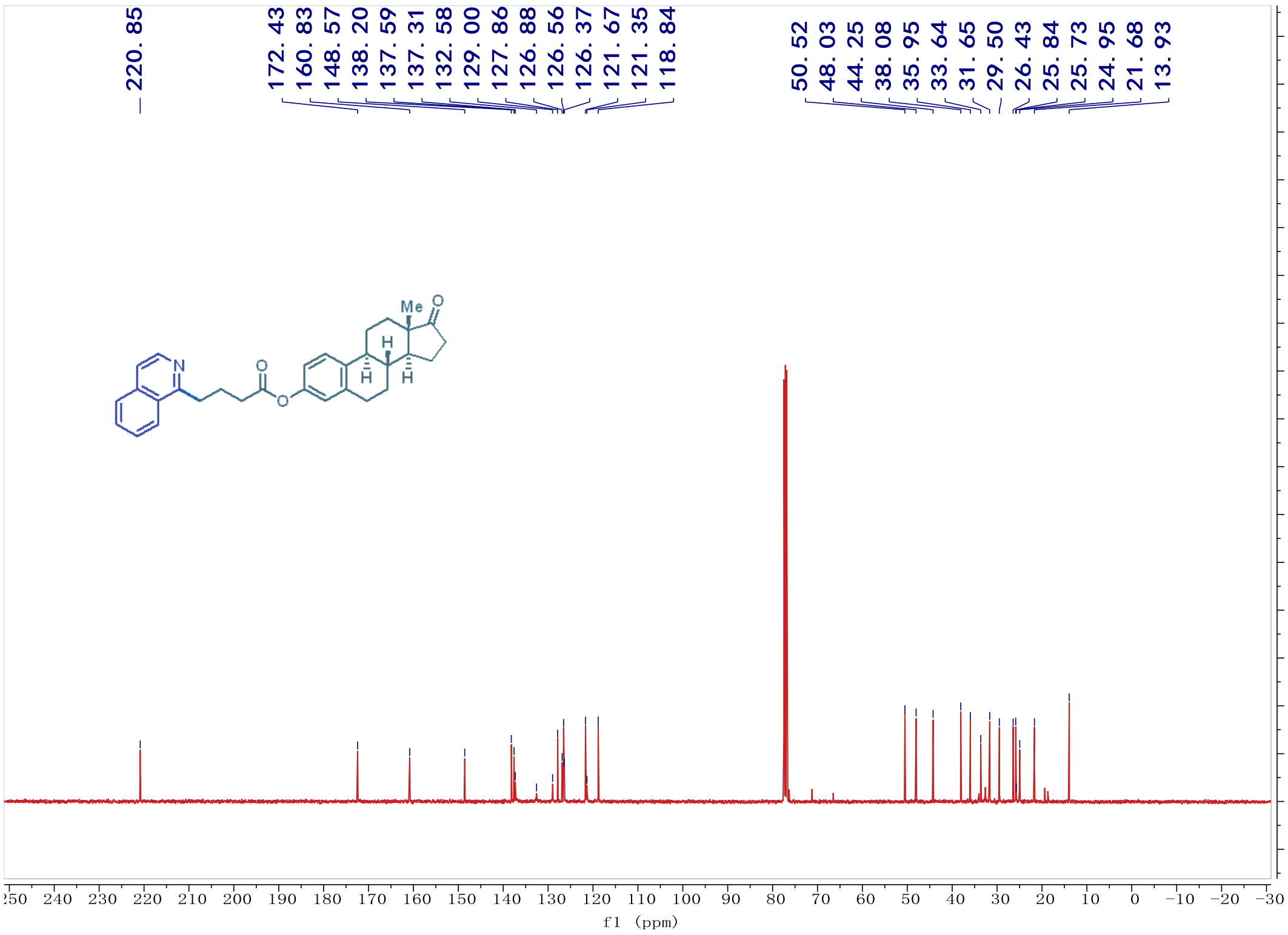

^{1}H NMR spectrum of **4-28** (400 MHz, $CDCl_3$).

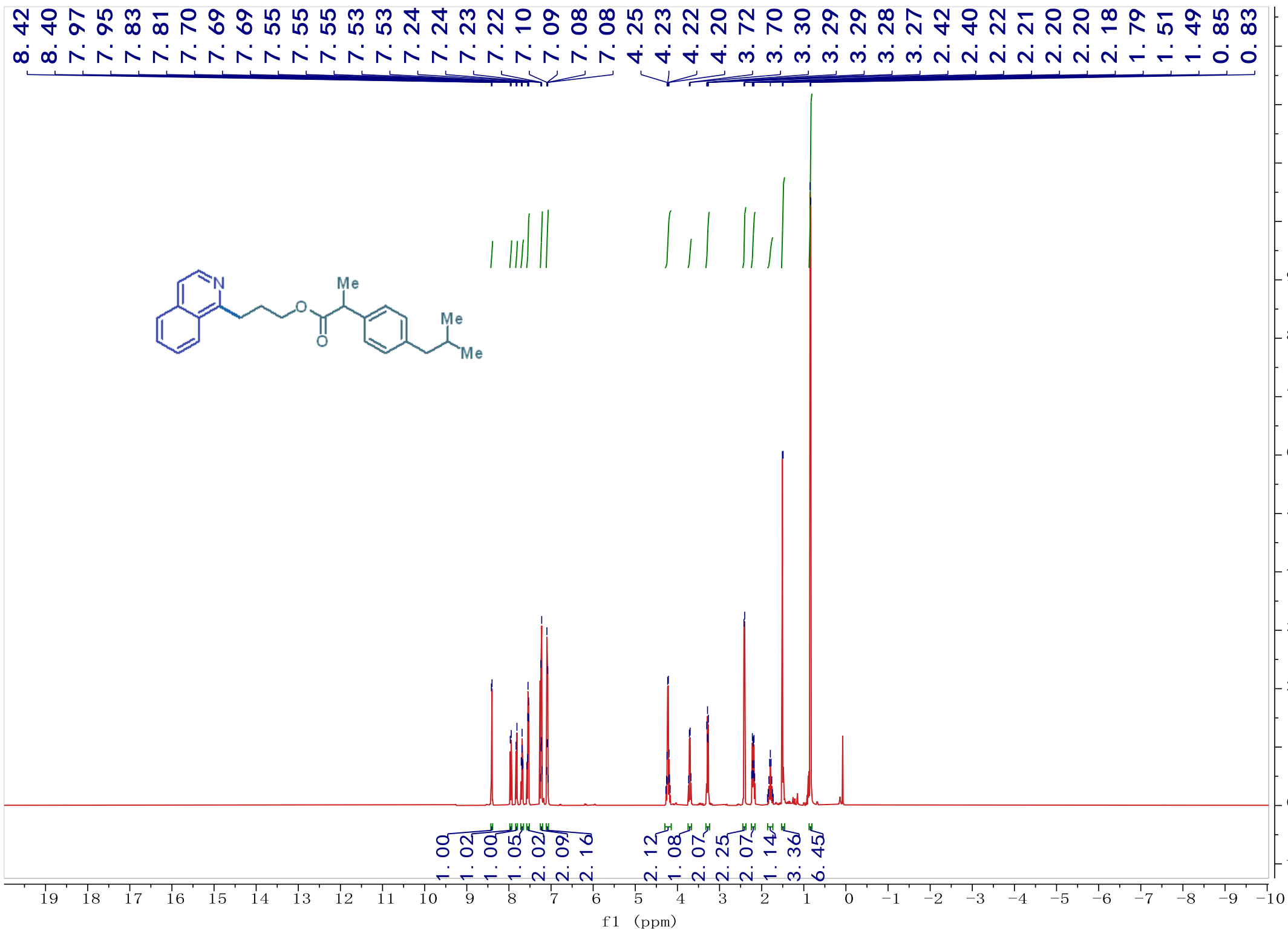

$^{13}C\{^1H\}$ NMR spectrum of **4-28** (101 MHz, $CDCl_3$).

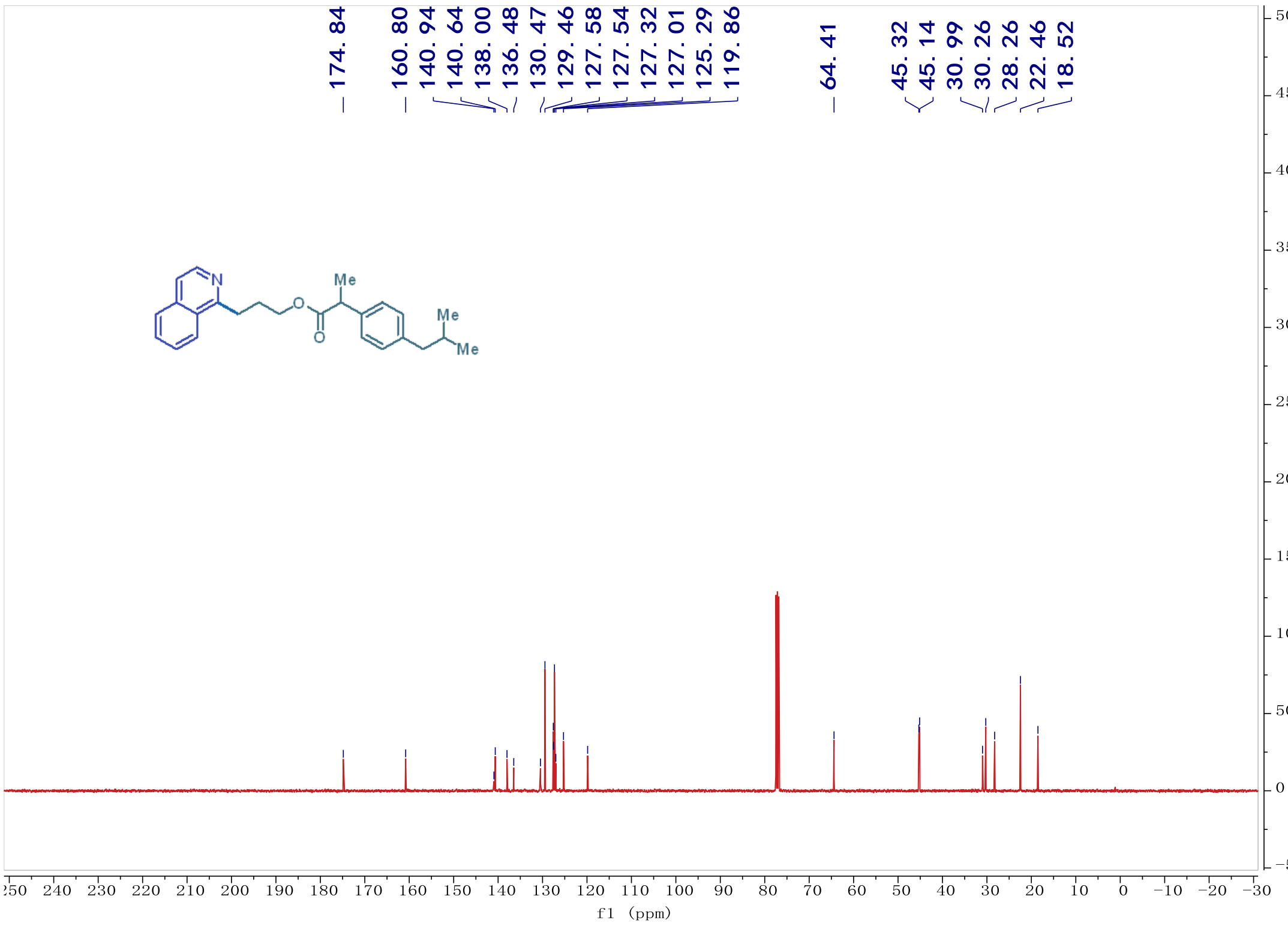

^1H NMR spectrum of **4-29** (400 MHz, $CDCl_3$).

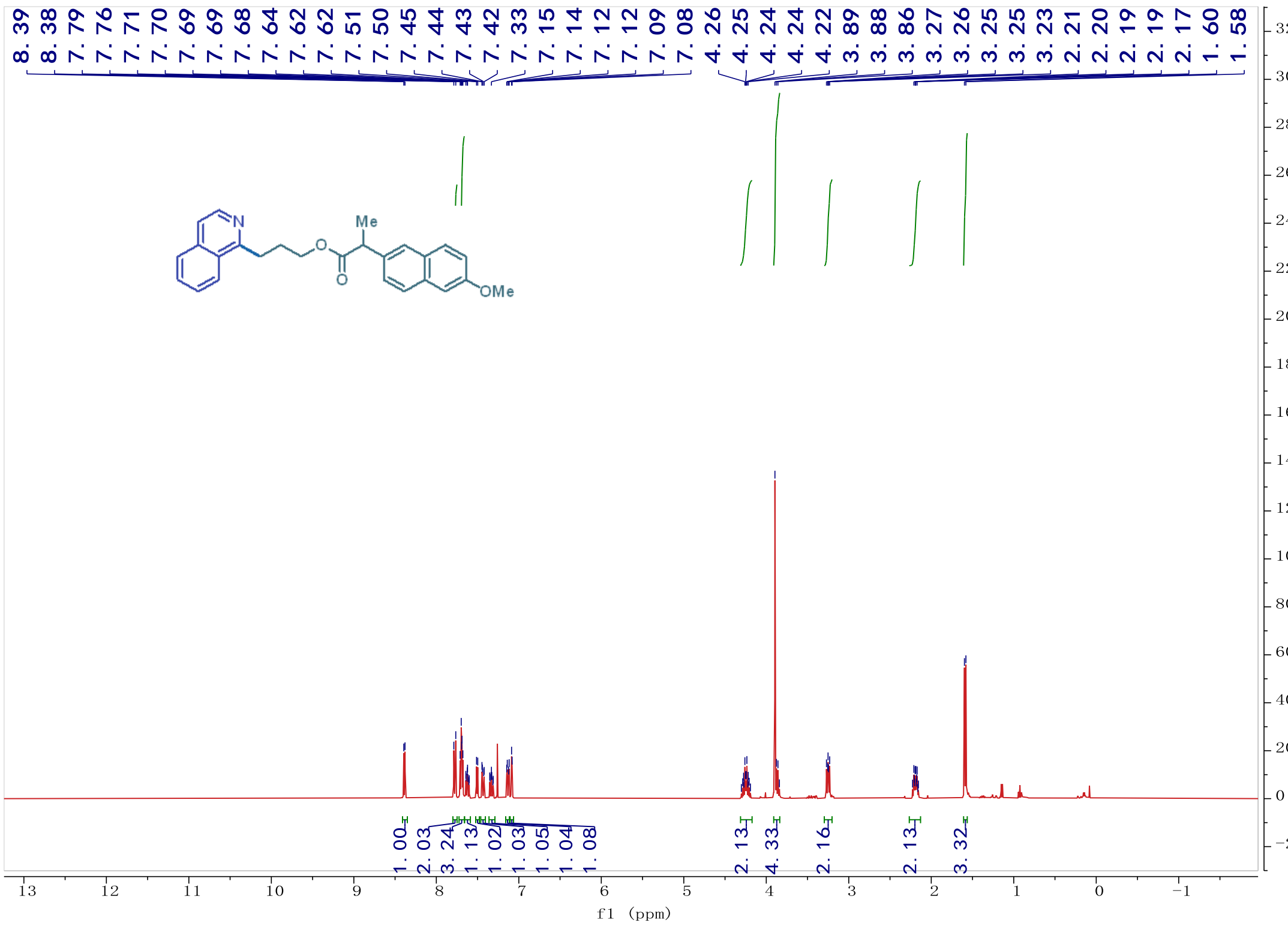

$^{13}C\{^{1}H\}$ NMR spectrum of **4-29** (101 MHz, $CDCl_3$).

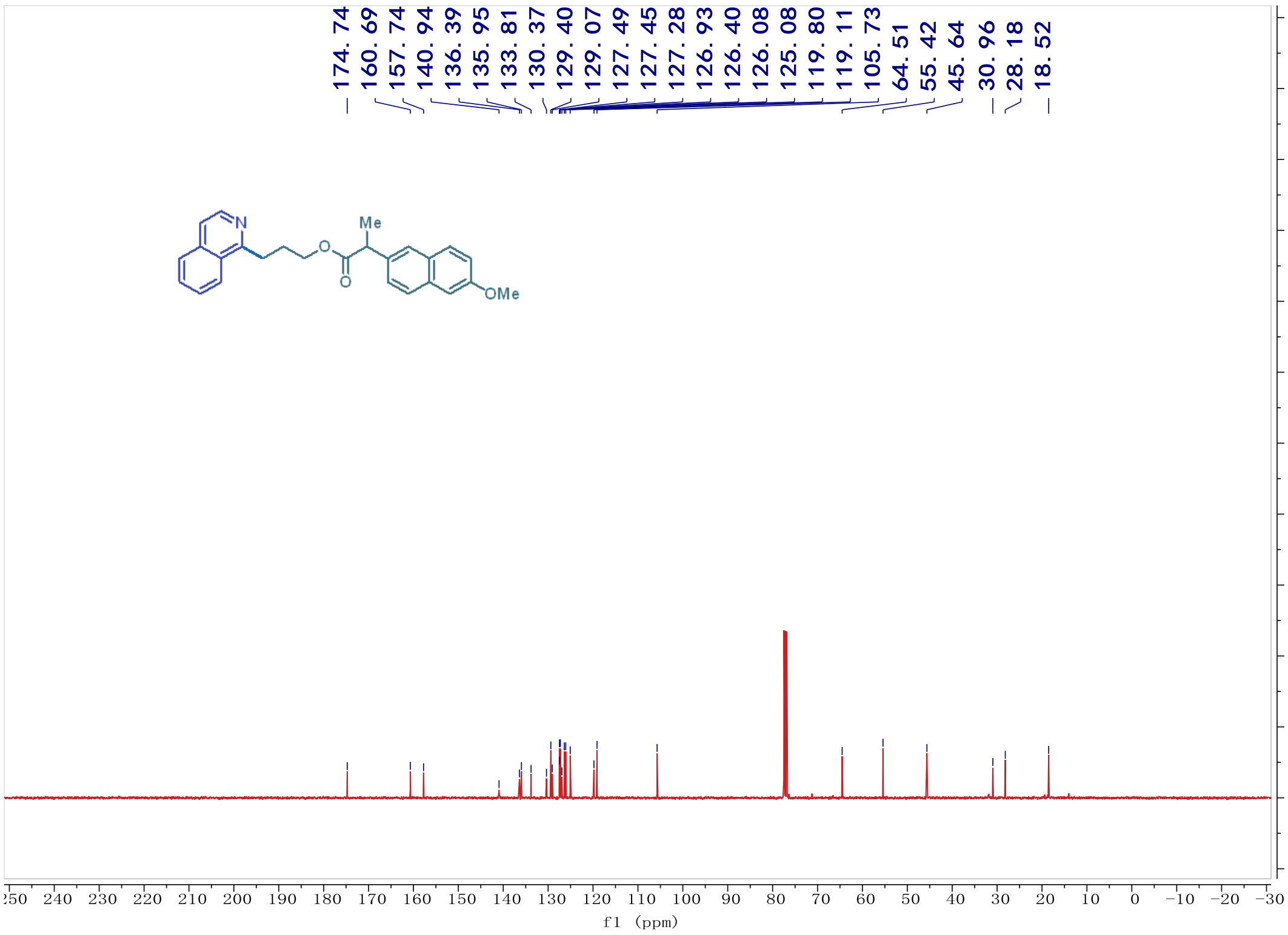

^{1}H NMR spectrum of **4-30** (400 MHz, $CDCl_3$).

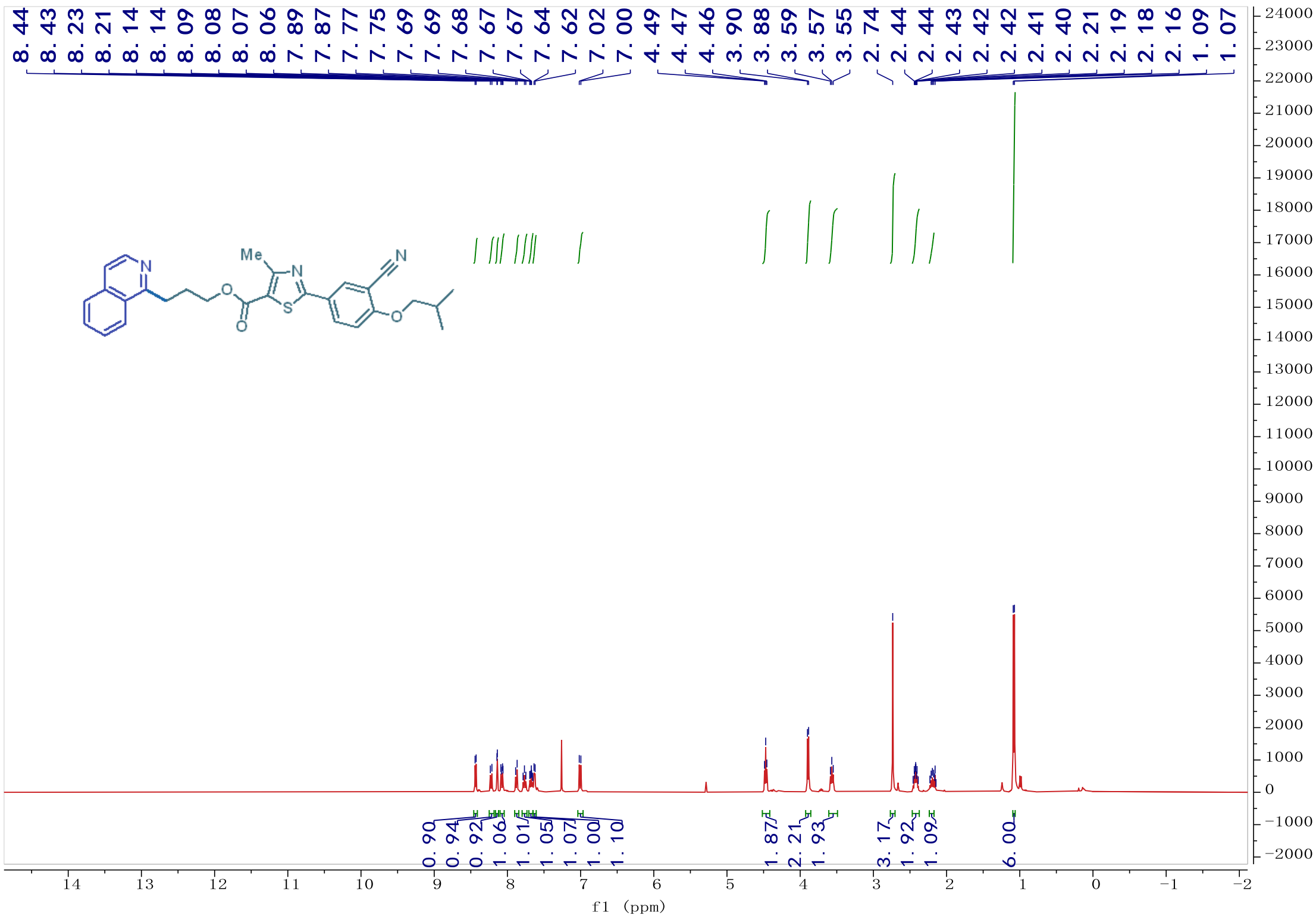

$^{13}C\{^{1}H\}$ NMR spectrum of **4-30** (101 MHz, $CDCl_3$).

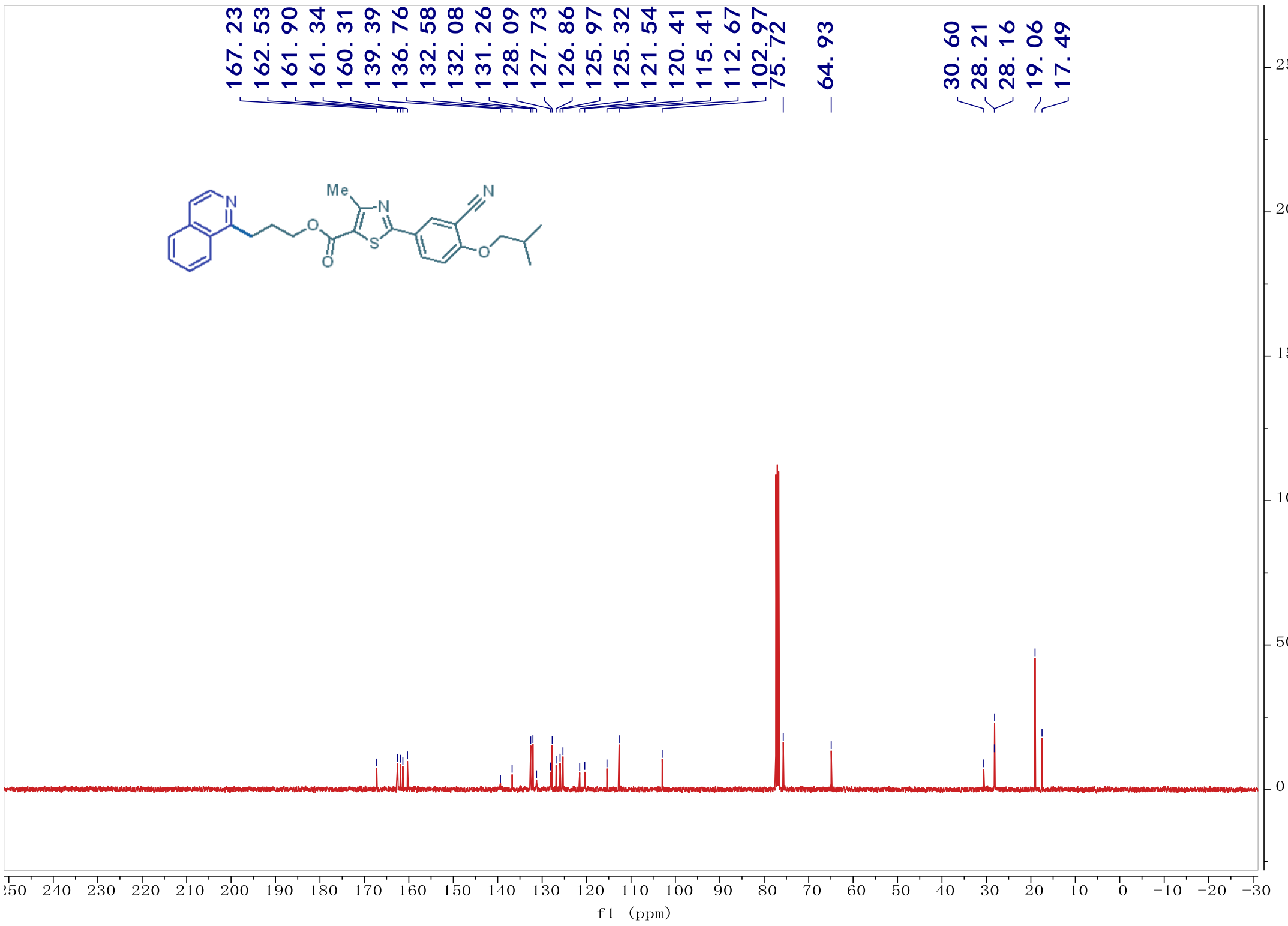

^{1}H NMR spectrum of **4-31** (400 MHz, $CDCl_3$).

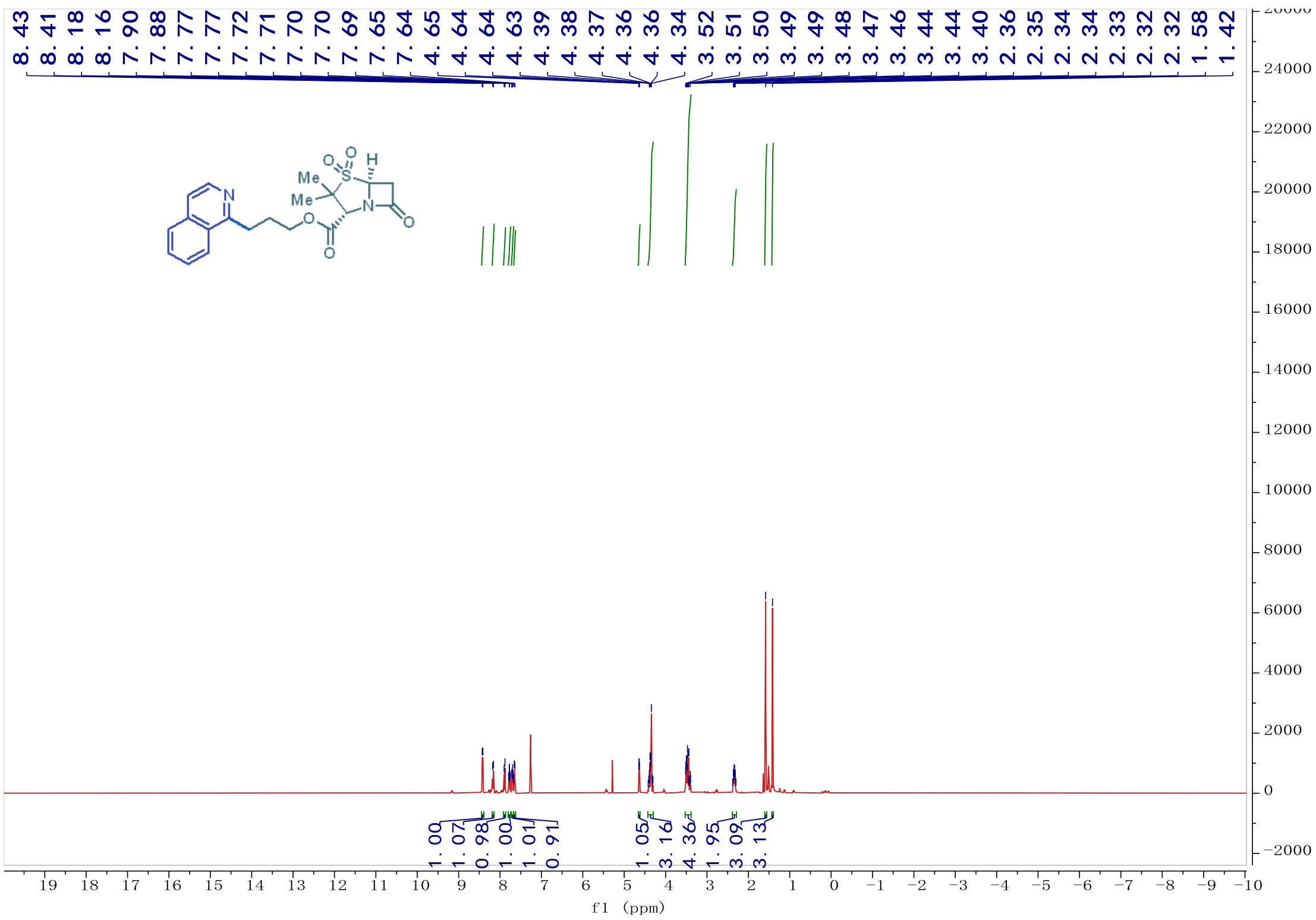

$^{13}C\{^{1}H\}$ NMR spectrum of **4-31** (101 MHz, CDCl₃).

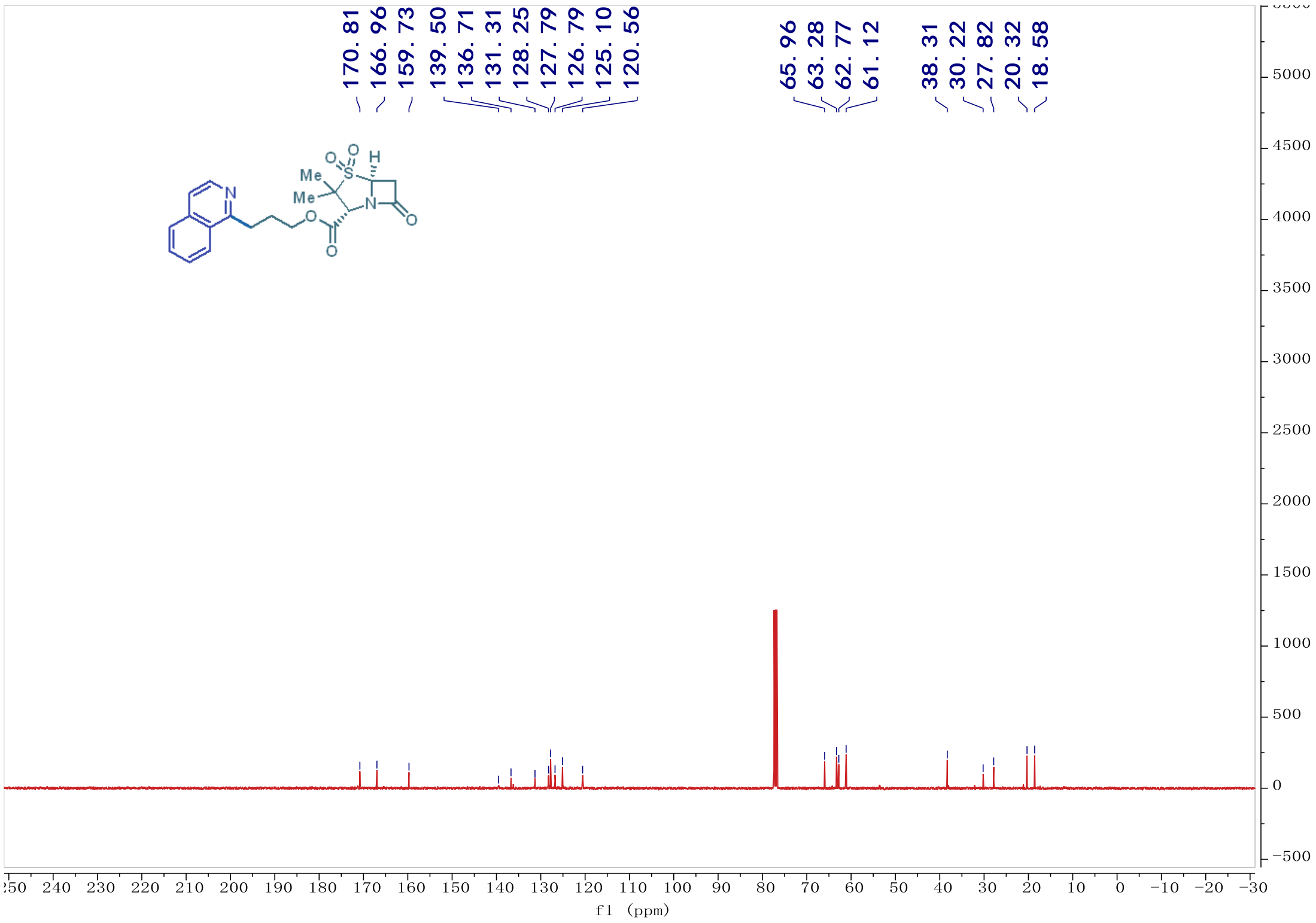

^{1}H NMR spectrum of **6-1** (400 MHz, CDCl₃).

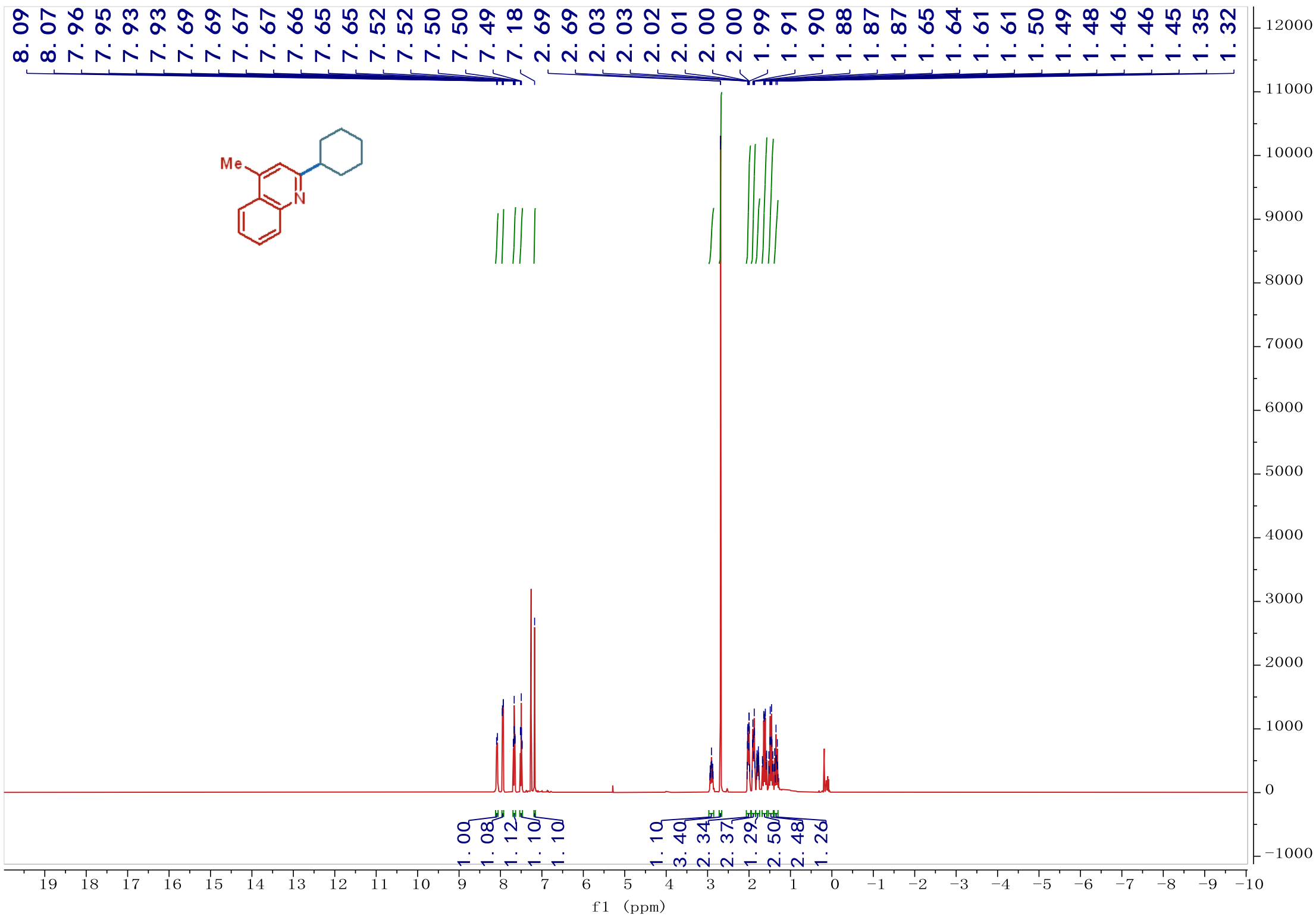

$^{13}C\{^{1}H\}$ NMR spectrum of **6-1** (101 MHz, $CDCl_3$).

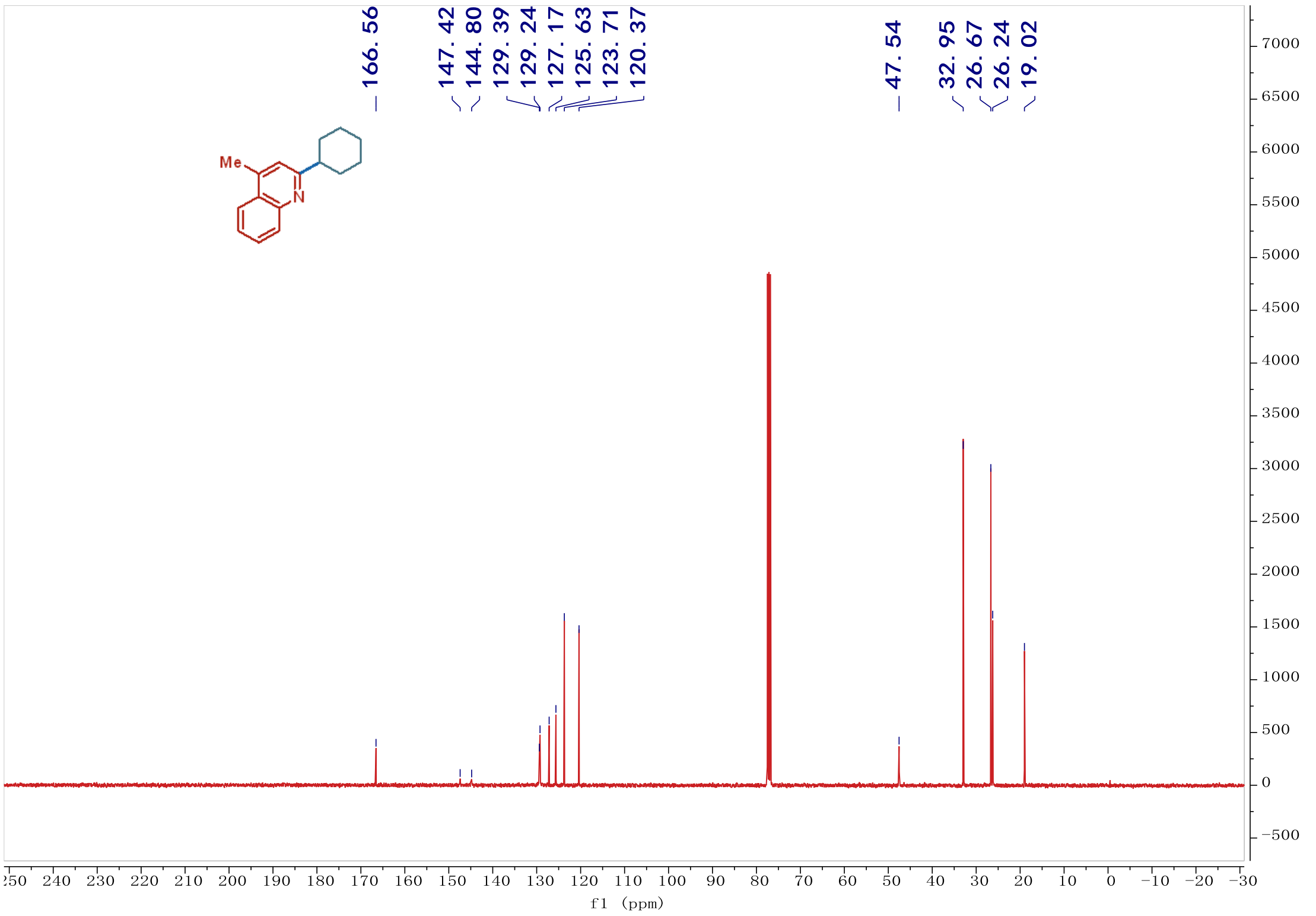

^{1}H NMR spectrum of **6-2** (400 MHz, $CDCl_3$).

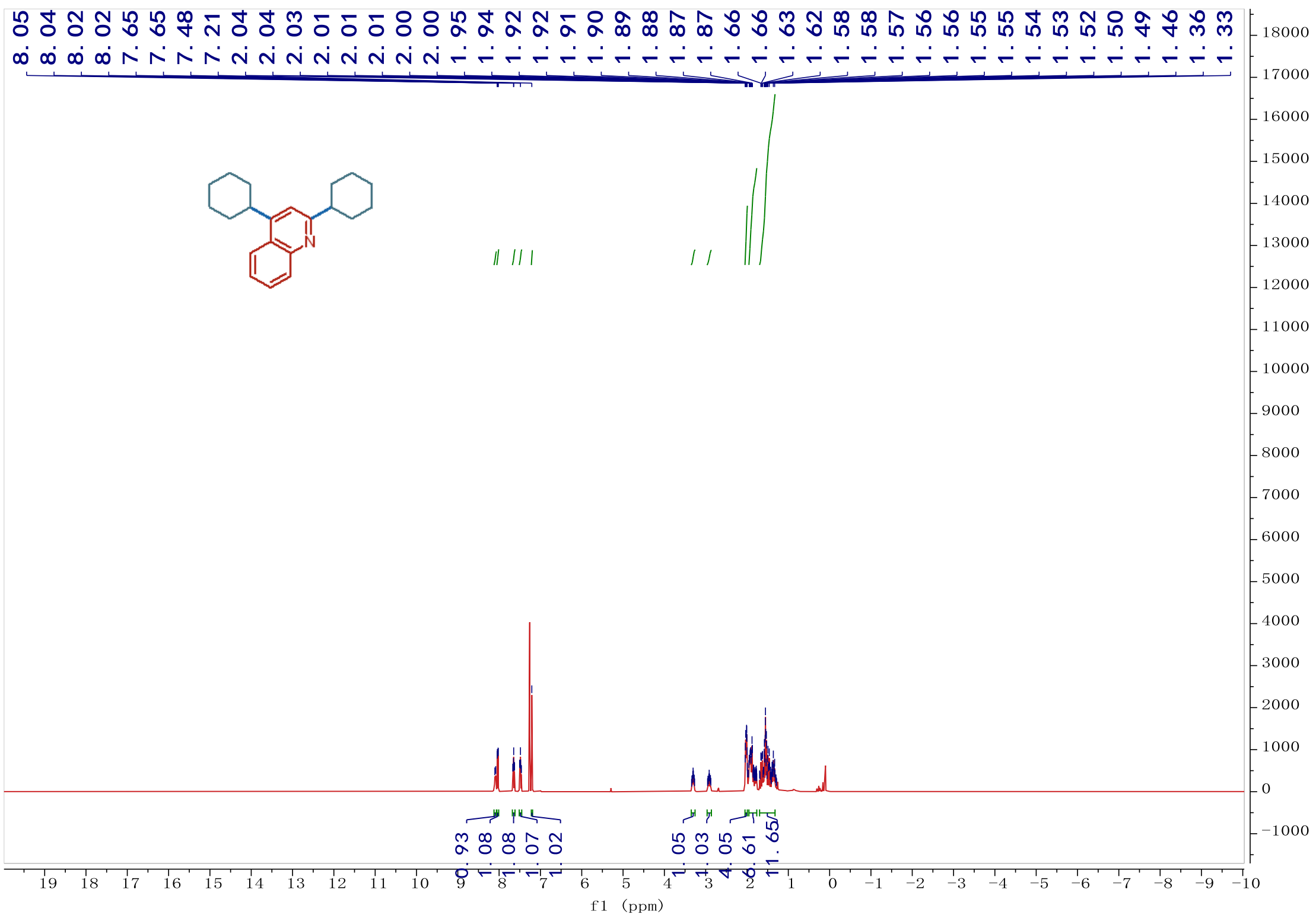

$^{13}C\{^{1}H\}$ NMR spectrum of **6-2** (101 MHz, CDCl3).

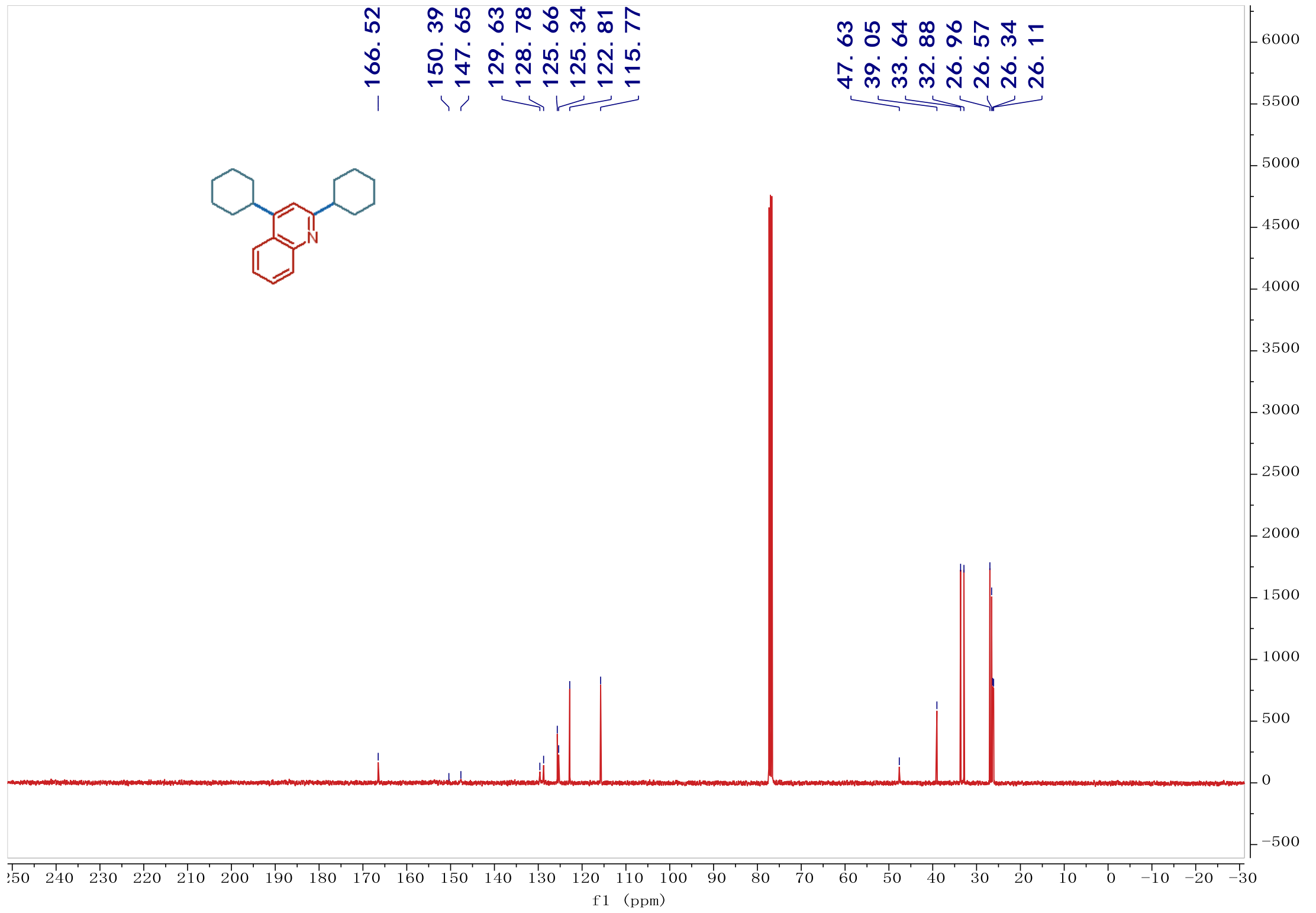

^{1}H NMR spectrum of **6-3** (400 MHz, CDCl3).

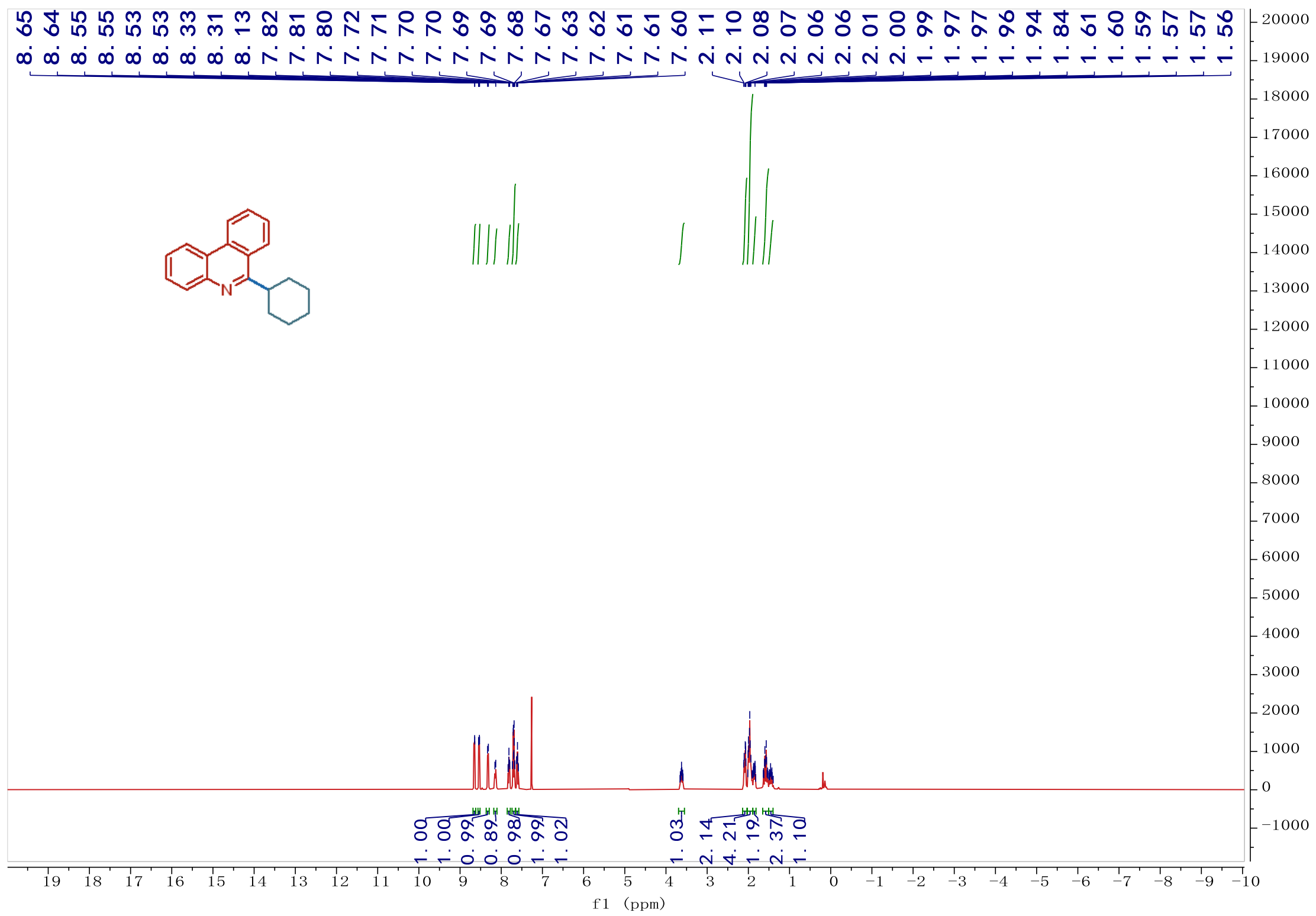

$^{13}C\{^1H\}$ NMR spectrum of **6-3** (101 MHz, $CDCl_3$).

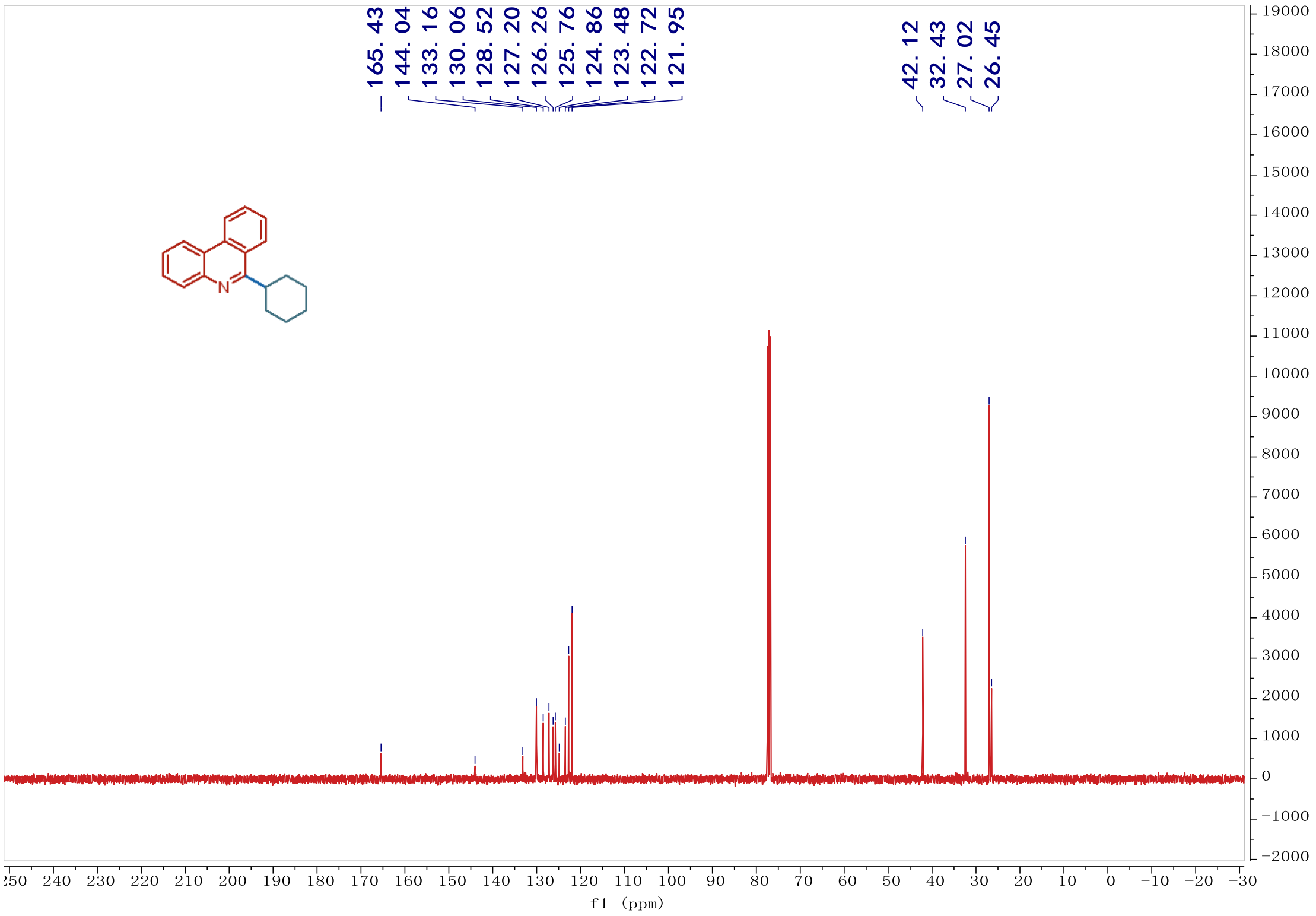

^1H NMR spectrum of **6-4** (400 MHz, $CDCl_3$).

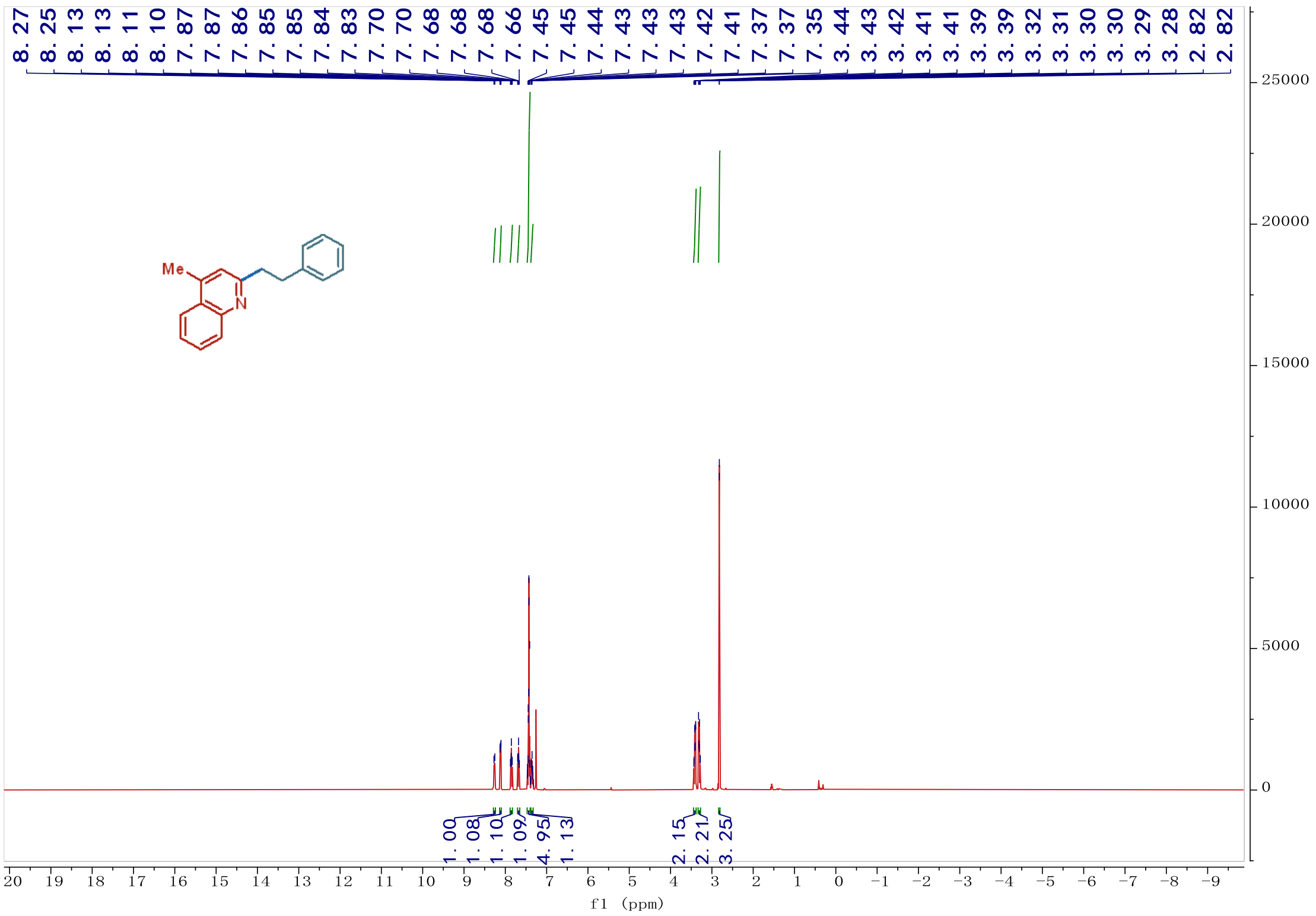

$^{13}C\{^{1}H\}$ NMR spectrum of **6-4** (101 MHz, $CDCl_3$).

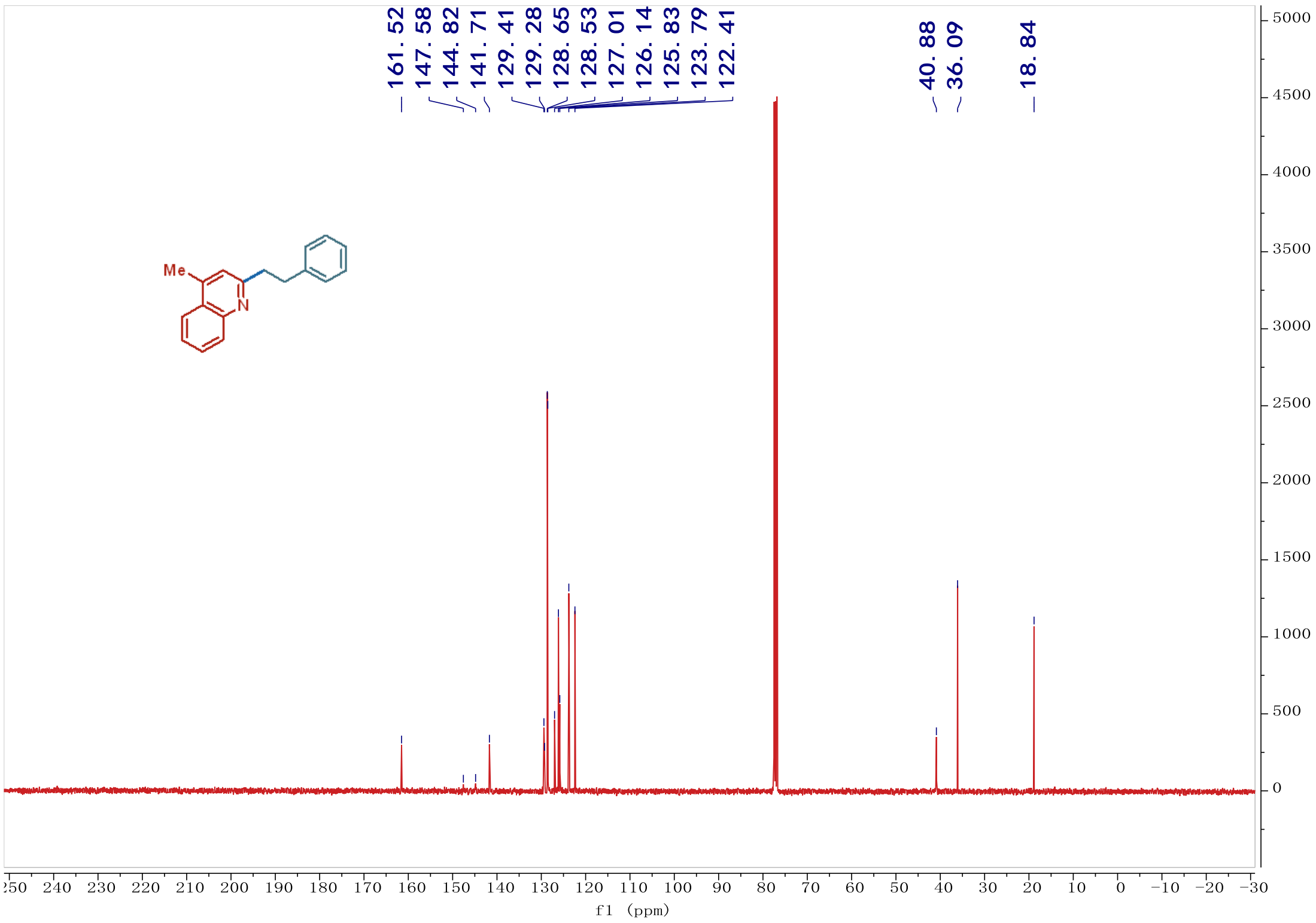

^{1}H NMR spectrum of **6-5** (400 MHz, $CDCl_3$).

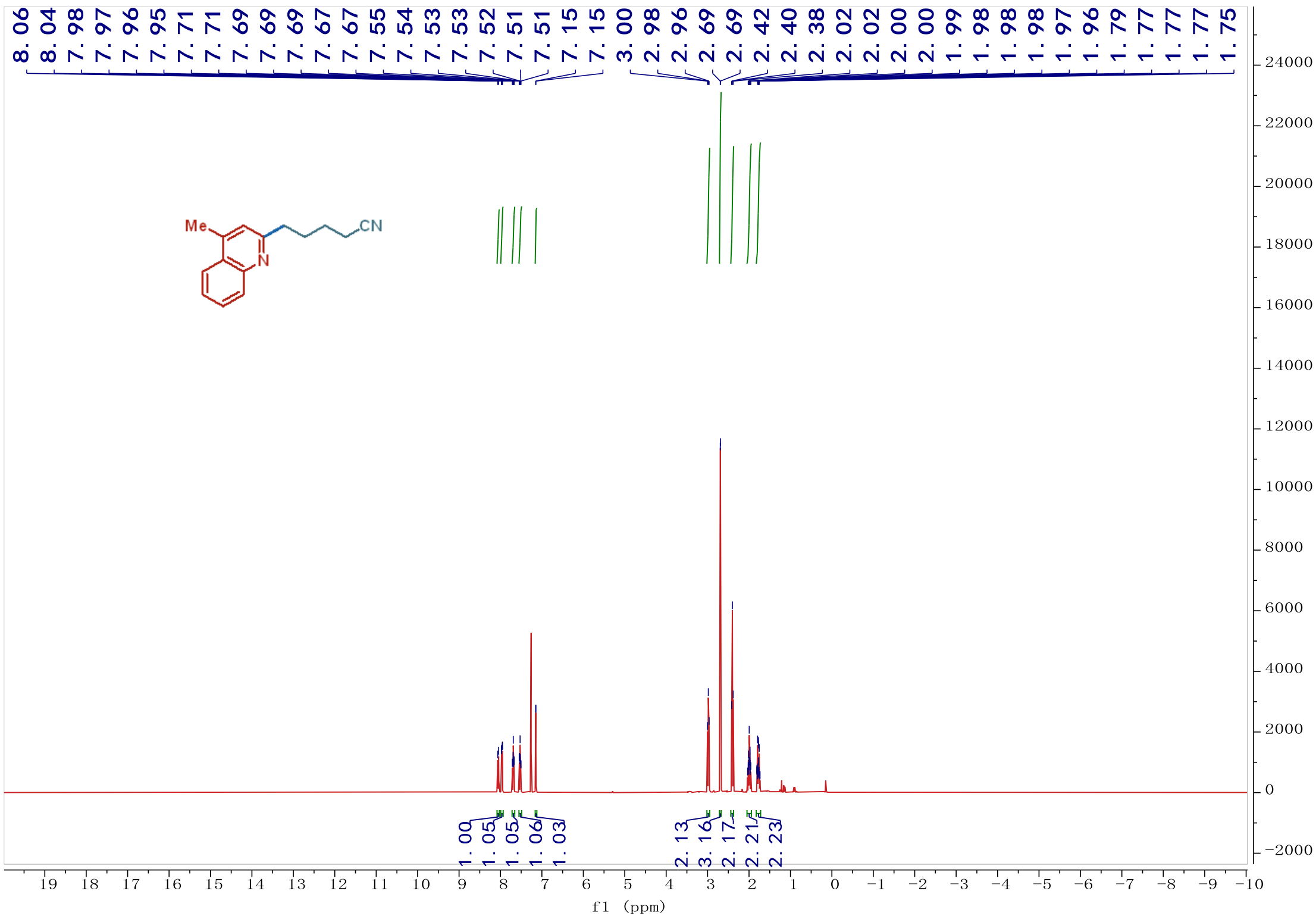

$^{13}C\{^{1}H\}$ NMR spectrum of **6-5** (101 MHz, $CDCl_3$).

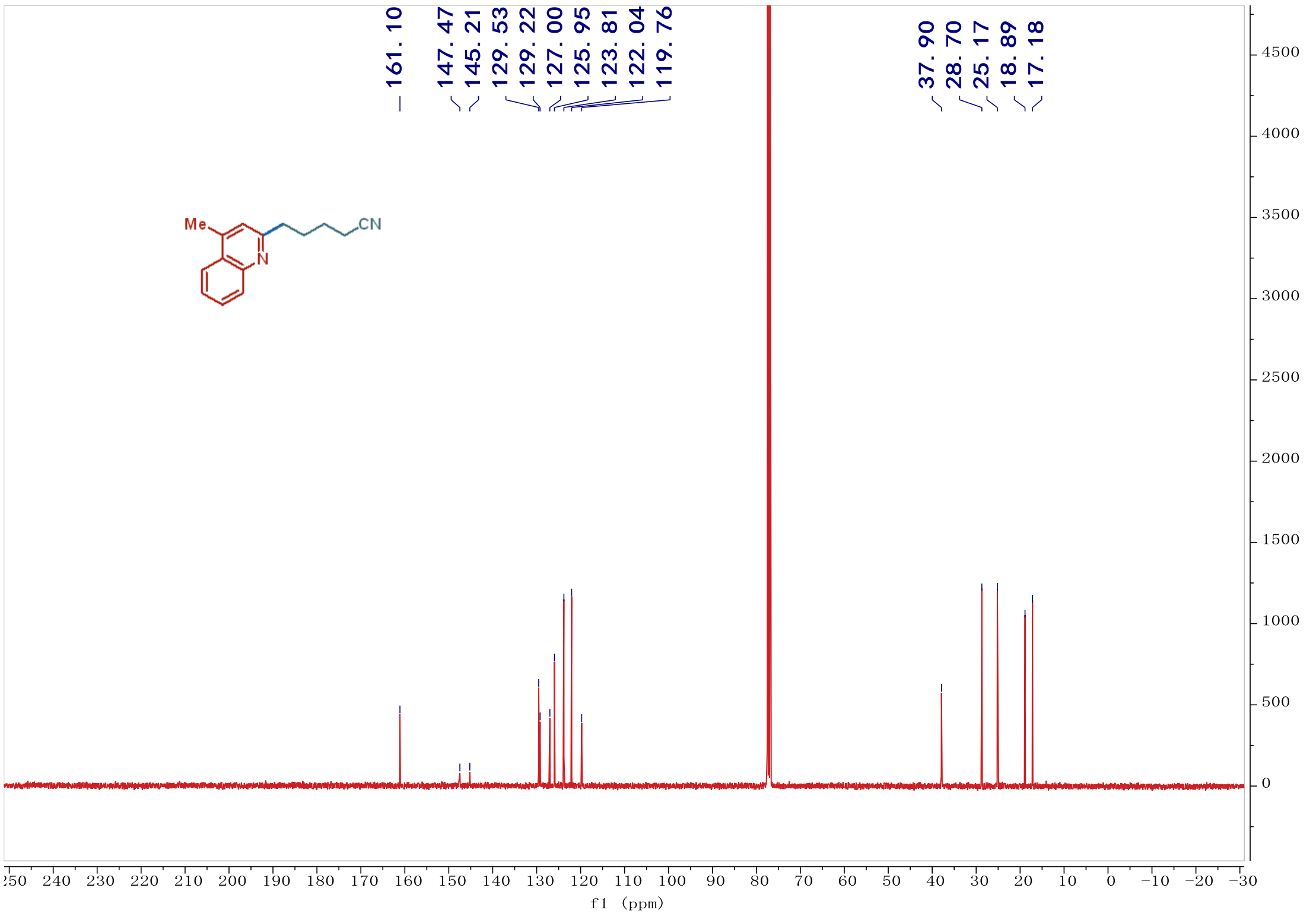

^{1}H NMR spectrum of **7** (400 MHz, $CDCl_3$).

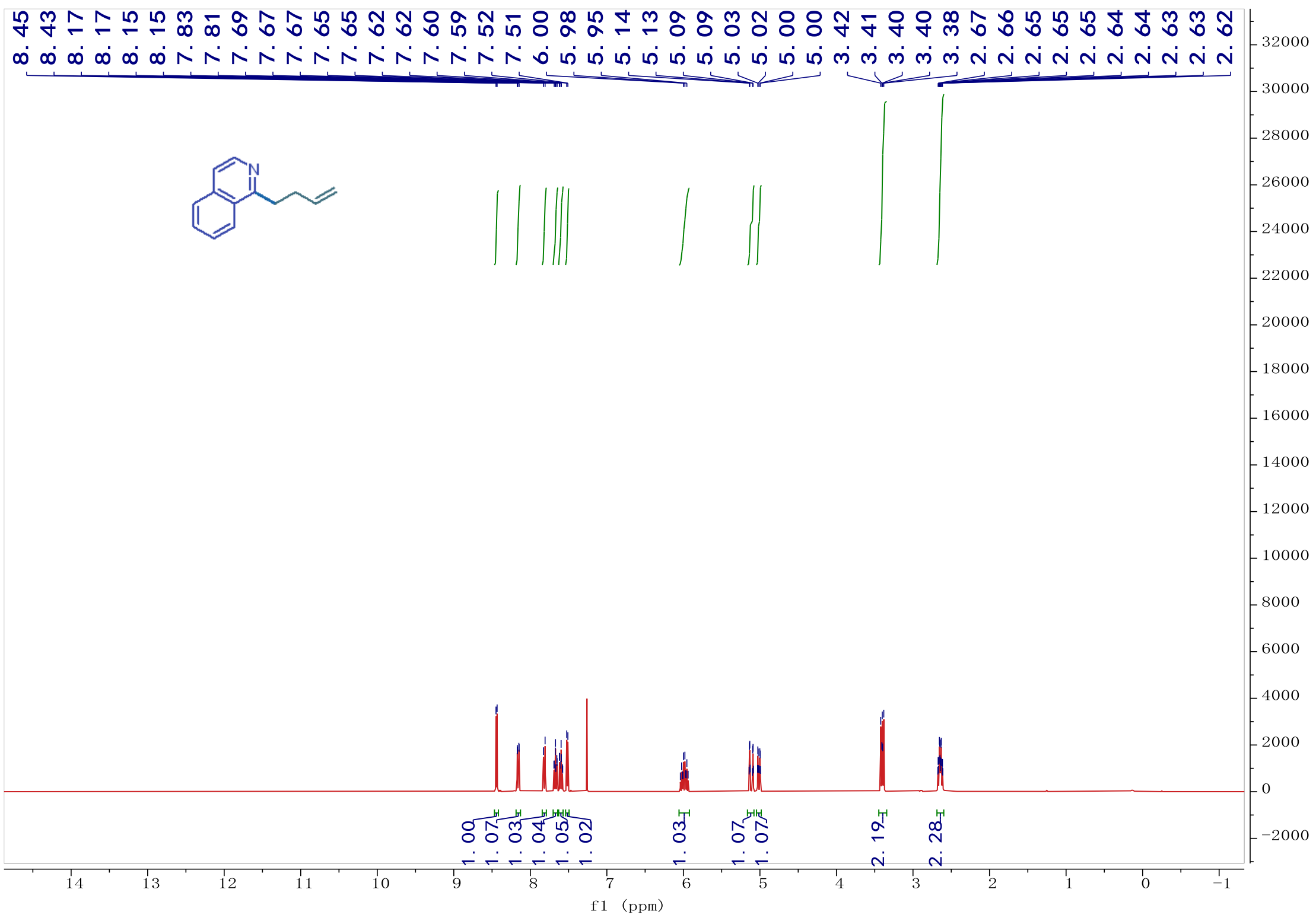

$^{13}C\{^{1}H\}$ NMR spectrum of **7** (101 MHz, $CDCl_3$).

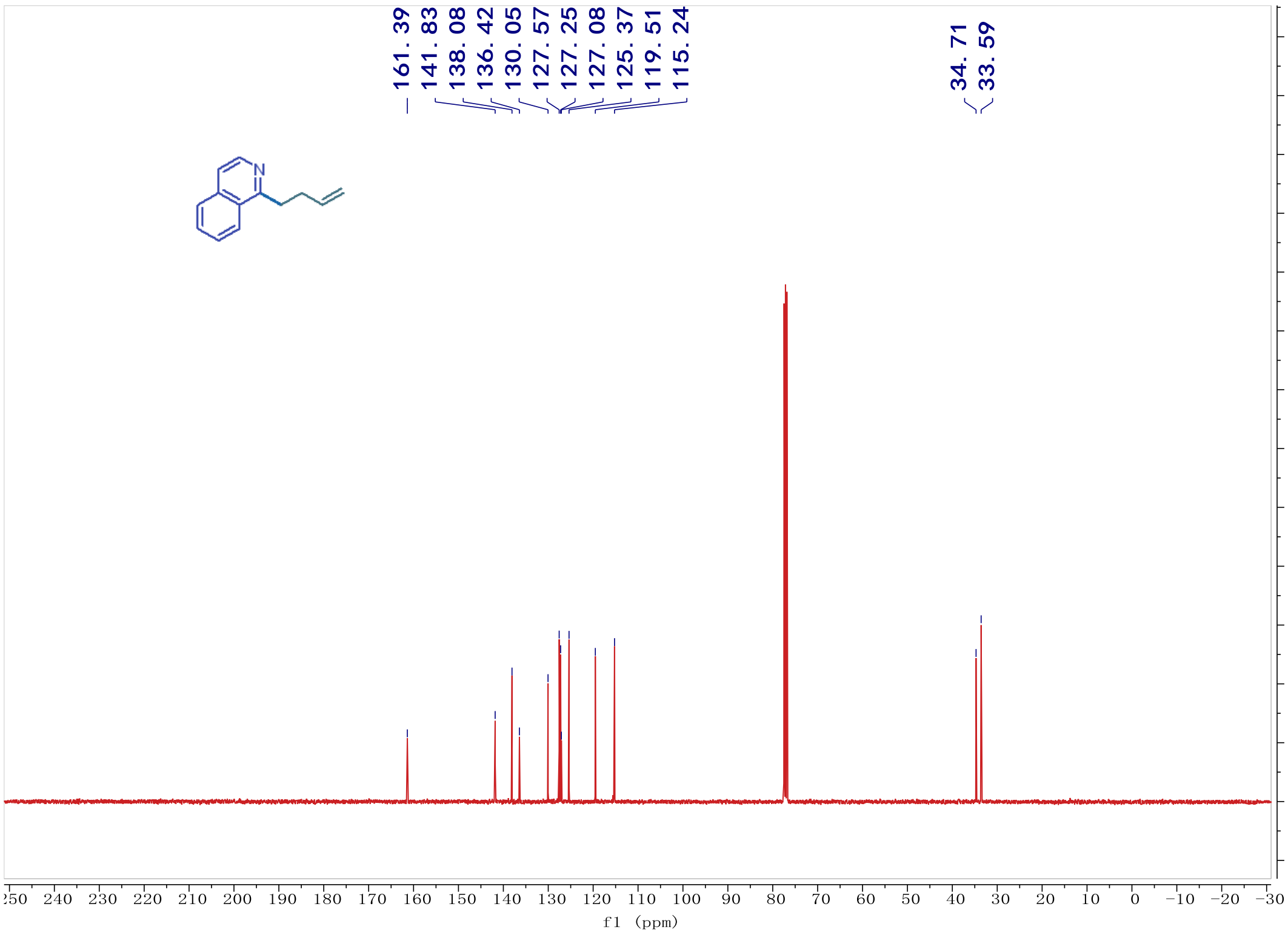

7. References

(1) Elbert, B. L.; Farley, A. J. M.; Gorman, T. W.; Johnson, T. C.; Genicot, C.; Lallemand, B.; Pasau, P.; Flasz, J.; Castro, J. L.; MacCoss, M.; et al. C−H Cyanation of 6-Ring N-Containing Heteroaromatics. *Chem. Eur. J.* **2017**, *23* (59), 14733-14737. DOI: 10.1002/chem.201703931.

(2) Peng, J.; Wang, A.; Liu, Y.; Chen, F.; Tang, G.; Zhao, Y. Selective Functionalization of White Phosphorus with Alkyl Bromides under Photocatalytic Conditions: A Chlorine-Free Protocol to Dialkyl and Trialkyl Phosphine Oxides. *Org. Lett.* **2024**, *26* (43), 9316-9321. DOI: 10.1021/acs.orglett.4c03494.

(3) Huang, X.; Shen, B.; Yao, M.; Duan, A.; Ma, Y. Nickel-Catalyzed Chemo- and Diastereoselective Dearomative Dialkylation of Indoles with Two Different Alkyl Halides. *Angew. Chem. Int. Ed.* **2026**, *65* (6), e20330. DOI: 10.1002/anie.202520330 (acccessed 2026/03/30).

(4) Li, J.-L.; Zhu, C.-M.; Li, H.-G.; Yuan, P.-F.; Meng, Q.-Y. Water as an Electron Donor for Cross-Electrophile Coupling Reactions. *J. Am. Chem. Soc.* **2025**, *147* (37), 33372-33378. DOI: 10.1021/jacs.5c12176.

(5) Ye, B.; Su, L.; Zheng, K.; Gao, S.; Liu, J. Synergistic Photoredox/Palladium Catalyzed Enantioconvergent Carboxylation of Racemic Heterobiaryl (Pseudo)Halides with CO_2. *Angew. Chem. Int. Ed.* **2025**, *64* (1), e202413949. DOI: 10.1002/anie.202413949 (acccessed 2026/03/30).

(6) Tian, Y.; Fu, X. X.; Cheng, R. M.; Feng, J.; Shen, M. H.; Zhu, C. F.; Xu, H. D. Visible-Light-Mediated Alkylation of N-Heteroarenes Using 2-Mercaptothiazolinium Salts as Alkyl Radical Source. *Eur. J. Org. Chem.* **2024**, *27* (26). DOI: 10.1002/ejoc.202400344.

(7) Boaz, H.; Rollefson, G. K. The Quenching of Fluorescence. Deviations from the Stern-Volmer Law. *J. Am. Chem. Soc.* **1950**, *72* (8), 3435-3443. DOI: 10.1021/ja01164a032.

(8) Megerle, U.; Lechner, R.; König, B.; Riedle, E.; Megerle, U. Laboratory apparatus for the accurate, facile and rapid determination of visible light photoreaction quantum yields. *Photochem. Photobiol. Sci.* **2010**, *9* (10), 1400-1406, 10.1039/C0PP00195C. DOI: 10.1039/C0PP00195C.

(9) Thapa, S.; Kafle, A.; Gurung, S. K.; Montoya, A.; Riedel, P.; Giri, R. Ligand-Free Copper-Catalyzed Negishi Coupling of Alkyl-, Aryl-, and Alkynylzinc Reagents with Heteroaryl Iodides. *Angew. Chem. Int. Ed.* **2015**, *54* (28), 8236-8240. DOI: 10.1002/anie.201502379 (acccessed 2026/03/30).

(10) Onoda, M.; Fujita, K.-i. Iridium-Catalyzed C-Alkylation of Methyl Group on *N*-Heteroaromatic Compounds using Alcohols. *Org. Lett.* **2020**, *22* (18), 7295-7299. DOI: 10.1021/acs.orglett.0c02635.

(11) Jian, Y.; Chen, M.; Yang, C.; Xia, W. j. Minisci-Type C–H Cyanoalkylation of Heteroarenes Through N–O/C–C Bonds Cleavage. *Eur. J. Org. Chem.* **2019**, *2020* (10), 1439-1442. DOI: 10.1002/ejoc.201900406.

(12) Yang, L.; Shi, X.; Zhang, F.; Luo, W.-K. Oxidant-Triggered C1-Benzylation of Isoquinoline by Iodine--Catalyzed Cross-Dehydrogenative-Coupling with Methylarenes. *Synlett* **2016**, *28* (04), 494-498. DOI: 10.1055/s-0036-1588331.

(13) Lyu, X.-L.; Huang, S.-S.; Song, H.-J.; Liu, Y.-X.; Wang, Q.-M. Visible-Light-Induced Copper-Catalyzed Decarboxylative Coupling of Redox-Active Esters with *N*-Heteroarenes. *Org. Lett.* **2019**, *21* (14), 5728-5732. DOI: 10.1021/acs.orglett.9b02105.

(14) Huang, J.-S.; Wang, Z.-H.; Li, X.-T.; Guo, S.-Y.; Jiang, X.-L.; Chen, Q.-A. Traceless Aminoalkyl Radical-Induced Halogen-Atom Transfer for Minisci Reactions. *Org. Lett.* **2025**, *27* (23), 6227-6232. DOI: 10.1021/acs.orglett.5c01794.

(15) Klauck, F. J. R.; James, M. J.; Glorius, F. Deaminative Strategy for the Visible-Light-Mediated Generation of Alkyl Radicals. *Angew. Chem. Int. Ed.* **2017**, *56* (40), 12336-12339. DOI: 10.1002/anie.201706896 (acccessed 2026/03/30).
(16) Zhao, Z.; Jing, Q.; Liu, H.; Liu, N.; Young, D. J.; Ren, Z.-G.; Li, H.-X. Decarboxylative and Deoxygenative/Decarbonylative Alkylation of Heterocycles with α-Keto Acids. *Org. Lett.* **2026**, *28* (8), 2801-2806. DOI: 10.1021/acs.orglett.6c00440.
(17) Jaiswal, Y.; Kumar, Y.; Pal, J.; Subramanian, R.; Kumar, A.; Jaiswal, Y. Rapid synthesis of polysubstituted phenanthridines from simple aliphatic/aromatic nitriles and iodo arenes via Pd(ii) catalyzed domino C−C/C−C/C−N bond formation. *Chem. Commun.* **2018**, *54* (52), 7207-7210, 10.1039/C8CC03556C. DOI: 10.1039/C8CC03556C.
(18) Laze, L.; Quevedo-Flores, B.; Bosque, I.; Gonzalez-Gomez, J. C. Alkanes in Minisci-Type Reaction under Photocatalytic Conditions with Hydrogen Evolution. *Org. Lett.* **2023**, *25* (48), 8541-8546. DOI: 10.1021/acs.orglett.3c02619.
(19) Feng, G.; Zhang, C.; Cai, T.; Wang, C.; Wang, X.-H.; Wang, Y.-S.; Yu, L.; Jin, J. Visible-Light-Induced Organobismuth(III)-Initiated Halogen-Atom Transfer for the Alkylation of Heterocycles with Unactivated Alkyl Iodides. *Org. Lett.* **2025**, *27* (45), 12483-12489. DOI: 10.1021/acs.orglett.5c03755.
(20) Price, G. A.; Bogdan, A. R.; Aguirre, A. L.; Iwai, T.; Djuric, S. W.; Organ, M. G.; Price, G. A. Continuous flow Negishi cross-couplings employing silica-supported Pd-PEPPSI−IPr precatalyst. *Catal. Sci. Technol.* **2016**, *6* (13), 4733-4742, 10.1039/C6CY00331A. DOI: 10.1039/C6CY00331A.
